# Deep learning-based structural prediction (AlphaFold 3) of shrimp IMNV RdRp and identification of seaweed-derived metabolites for antiviral intervention in aquaculture

**DOI:** 10.1016/j.virusres.2026.199796

**Published:** 2026-09-08

**Authors:** Mostafizur Rahman, Md Ahad Ali, Tahosin Tasneem Samara, Monish Saha, Pritom Kundu, Hriddhi Sarker, Ankita Dutta, Neeraj Kumar

**Affiliations:** a Department of Biotechnology and Genetic Engineering, Gopalganj Science and Technology University, Gopalganj, Bangladesh; b Department of Computational Chemistry and Drug Design, Panacea Research Center, Bangladesh; c Department of Biochemistry and Molecular Biology, University of Rajshahi, Rajshahi, Bangladesh; d Department of Life Sciences, Independent University, Dhaka, Bangladesh; e Department of Biochemistry and Cell Biology, Bangladesh University of Health Sciences, Dhaka, Bangladesh; f Department of Pharmacy, University of Rajshahi, Rajshahi, Bangladesh; g Department of Pharmacy, University of Science and Technology Chittagong, Chittagong, Bangladesh; h Department of Pharmaceutical Chemistry, Bhupal Nobles' College of Pharmacy, Udaipur, Rajasthan, 313001, India

**Keywords:** RNA-dependent RNA polymerase, Deep learning, AlphaFold 3, Seaweed metabolites, Infectious myonecrosis virus

## Abstract

Infectious Myonecrosis Virus (IMNV) remains a major global threat to shrimp aquaculture, causing severe economic losses due to the lack of approved antiviral therapeutics. In this study, an advanced in silico approach was employed, utilizing a deep learning-based prediction of AlphaFold 3 protein structures and large-scale virtual screening of seaweed-derived metabolites to identify potential inhibitors targeting RNA-dependent RNA polymerase (RdRp). To identify potential seaweed-derived candidate compounds against IMNV, a total of 1077 compounds were screened against the RdRp_M01 protein. The docking results suggest the top-ranked three bioactive compounds, such as GA002 (−13.0 kcal/mol), RC003 (-10.9 kcal/mol), and BE012 (-10.8 kcal/mol), based on their highest binding energy score and molecular interactions, for further analysis. The 200 ns molecular dynamics simulations showed that the selected complexes exhibited stable behavior throughout the simulation time, with relatively low standard deviation values. Post-simulation MMGBSA analysis revealed total binding free energies (ΔG_total) of −24.726, −26.722, and -34.850 kcal/mol for RdRp_M01_GA002, RdRp_M01_RC003, and RdRp_M01_BE012, respectively, indicating favorable binding affinity and stable protein-ligand interactions. The drug-likeness and physicochemical profiles of the selected compounds were acceptable, supporting their further investigation. Therefore, further validation through *in vitro* and *in vivo* studies is necessary to confirm their applicability in shrimp systems and to explore the potential of seaweed metabolites as candidate compounds targeting the IMNV RdRp and alternatives to synthetic drugs.

## Highlights

• Deep learning enabled structural prediction of the IMNV RdRp target protein structure.
• Virtual screening of seaweed metabolites identified 1077 promising antiviral candidates against RdRp.
• Computationally prioritized candidates (GA002, RC003, and BE012) showed favorable docking scores and diverse predicted protein-ligand interactions.
• 200 ns molecular dynamics simulations demonstrated stable performance of the selected Protein-ligand complexes.
• Seaweed-derived metabolites show potential as antiviral candidates for shrimp aquaculture.

## 1 Introduction

In today’s rapidly growing and densely populated world, food is essential for sustaining human life. Several sectors, such as meat, fish, and livestock, play a crucial role in meeting these nutritional needs. Among them, the fish sector plays a significant role in feeding the global population and accounts for a major share worldwide. Within the fish industry, shrimp contributes substantially to global aquaculture, with production volumes exceeding several million tons annually (International Markets for Fisheries and Aquaculture Products; 2025). However, annual outbreaks of known and unknown diseases reduce production, among which Infectious Myonecrosis Virus (IMNV) is one of the most critical diseases (Andrade et al., 2022).

Infectious Myonecrosis (IMN) was first reported in *Penaeus vannamei* in Brazil in 2002 and has since spread to several countries, including Indonesia, India, and Malaysia, largely due to the cross-border movement of farmed shrimp (Wan et al., 2023; Lightner et al., 2004; Poulos et al., 2006). IMNV is a non-enveloped, double-stranded RNA virus belonging to the family *Totiviridae*, which has been reported to infect several shrimp species, including *P. vannamei* (Pacific white shrimp), *P. monodon* (Black tiger shrimp), *P. merguiensis* (Banana prawn), and *P. esculentus* (Brown tiger prawn) (Arulmoorthy et al., 2020; Tang et al., 2019). IMNV infects shrimp at all life stages, including post-larvae, juveniles, and adults, but mortality is mainly observed in juveniles and adults (Mai et al., 2019; Coelho et al., 2009). IMNV-infected shrimp often exhibited a characteristic cooked appearance, along with whitish, opaque abdominal muscle tissue and whitish to reddish necrotic areas in the striated muscles, particularly in the distal abdominal segments and tail fan, which may become extensively necrotic and reddened (Poulos et al., 2006). In some cases, the tail and last abdominal segment turn red, and infected shrimp appear weak with reduced appetite (Jha et al., 2021; Lee et al., 2022). IMNV is known to cause significant losses, with mortality rates ranging between 40% and 70% (Tang et al., 2019). IMNV spreads efficiently among shrimp via infected tissue and water, as confirmed by experimental infections, and although no specific vectors are known, it may be transmitted between farms through seabirds via feces or expelled infected shrimp (Poulos and Lightner, 2006; Vanpatten et al., 2004).

IMNV is a double-stranded RNA (dsRNA) virus that infects shrimp, especially *P. vannamei*. IMNV possesses a double-stranded RNA genome that was initially reported to be 7561 bp in length but later revised to approximately 8226–8230 bp, comprising two open reading frames, with ORF1 encoding an RNA-binding and capsid protein and ORF2 encoding an RNA-dependent RNA polymerase (RdRp) (Wan et al., 2023; Tang et al., 2008; Senapin et al., 2007; Pathania et al., 2022). As like other RNA viruses, IMNV depends on an enzyme called RNA-dependent RNA polymerase (RdRp) for its replication cycle. RdRp is important in the transcription and replication of viral RNA, catalyzing the synthesis of RNA from an RNA template (Pathania et al., 2022). The host cells do not contain RdRp enzyme, which usually important for the viral RNA genome replication, RdRp is essential for viral propagation and is a promising target for antiviral drug development (Feijó et al., 2025). Despite causing major losses in shrimp farming, IMNV still has no approved vaccines or therapeutics, highlighting the urgent need to find effective ways to control and manage this virus.

Seaweed-derived metabolites represent a diverse and valuable source of bioactive compounds with significant potential in drug discovery, owing to their wide range of biological activities. Compounds isolated from green, brown, and red algae have shown activity against bacteria, viruses, fungi, and other epibionts, with properties including cytostatic, antiviral, antihelminthic, antibacterial, antifungal, and other bioactive effects (Pereira and Valado, 2023; Besednova et al., 2019; Sruthy and Baiju, 2024). In addition, both crude extracts and purified fractions from marine algae have been reported to show notable pharmacological effects, including anticoagulant, antiviral, antioxidant, anticancer, and anti-inflammatory activities (Kazłowska et al., 2010; Namvar et al., 2013; Cox et al., 2010; Damonte et al., 2004; Wijesinghe et al., 2011). These statements indicate that seaweed metabolites may provide potential candidate compounds for further investigation against aquatic pathogens.

An *in silico* study reported that *Nasturtium officinale* bioactive compounds exhibited antiviral potential against IMNV RdRp, with Rhamnetin 3-sophoroside showing the strongest binding affinities in the Indonesia and India_QDN RdRp variants (−7.8 and −8.7 kcal/mol, respectively), while Rhamnazin 3-sophoroside showed the highest affinity for the Mexico and India_QIL variants (−8.2 kcal/mol) (A'yunin et al., 2024). Previous *in vitro* studies demonstrated that the cathelicidin-derived eicosapeptide Ctn[15–34] exhibited antiviral activity against IMNV by neutralizing virus-induced cytotoxicity in *Litopenaeus vannamei* primary hemocyte cultures at concentrations below 12.5 μM (Vieira-Girão et al., 2017). Previous studies have explored antiviral strategies against IMNV in *L. vannamei* using RNA interference (RNAi), in which dsRNAs targeting the viral ORF1a and ORF1b regions suppressed viral replication and improved shrimp survival (Feijó et al., 2015). Previous *in vivo* studies investigated RNAi as an antiviral strategy against IMNV in *L. vannamei*, demonstrating that dsRNAs targeting specific viral genomic regions protected shrimp against IMNV infection, whereas dsRNAs targeting the polymerase or structural genes conferred limited or no protection (Loy et al., 2012). However, effective small-molecule inhibitors and sea-weed metabolites targeting the IMNV RNA-dependent RNA polymerase (RdRp) remain largely unexplored. Previous studies have demonstrated that seaweed-derived metabolites possess antiviral activity against shrimp viruses. Fucoidan isolated from the brown seaweed *Sargassum wightii* reduced mortality and WSSV infection in *Penaeus monodon* (Sivagnanavelmurugan et al., 2012). Sulfated galactans isolated from the red seaweed *Gracilaria fisheri* exhibited antiviral activity against WSSV by enhancing immune responses, reducing viral load, and lowering shrimp mortality (Wongprasert et al., 2014). Hot-water extracts of the brown seaweeds *Sargassum wightii* and *Sargassum duplicatum* enhanced resistance to WSSV infection in *Penaeus monodon*, reducing shrimp mortality and viral infectivity in a concentration-dependent manner (Immanuel et al., 2010). Collectively, these findings highlight the therapeutic potential of marine algal metabolites in shrimp aquaculture and support their application as promising antiviral agents against shrimp viral diseases.

Although RdRp is an important drug target of IMNV, there is no experimentally determined structure of IMNV RdRp available in the database. Seaweed-derived metabolites are well-recognized as a source of potentially bioactive/therapeutic compounds. Additionally, there is no such previous study that systematically evaluated the potential of seaweeds against IMN infection in shrimp (Chaube et al., 2023; Rudtanatip et al., 2014; Saravanan et al., 2025). This lack of targeted, structure-based evaluation of seaweed-derived metabolites to inhibit RdRp for IMNV infection is one of the knowledge gaps that should be addressed to develop sustainable antiviral approaches for shrimp farms.

Therefore, this study aims to identify potential seaweed metabolites targeting RdRp for further investigation against IMN infections in shrimps through bioinformatics approaches. The structure-based design procedure highly depends on the structural availability of the protein’s crystal structure (X-ray or NMR) and as there were no available experimental structure of RdRp of IMNV in the Protein Data Bank (PDB). Thus, we can build the protein structure hypothetically using protein modeling tools. AlphaFold 3, an AI system developed by Google DeepMind and Isomorphic Labs, predicts protein structures through integrating deep learning (primarily convolutional neural networks) with physical and biological knowledge (Albawi et al., 2017; Senior et al., 2020). AlphaFold 3 is an AI-driven, deep learning-based model that integrates protein sequence and evolutionary information using a Pairformer architecture and diffusion-based generation to accurately predict 3D biomolecular structures (Krokidis et al., 2025; Fang et al., 2025). The selected potential leads were further validated through molecular docking, molecular dynamics (MD) simulations, MM-GBSA-based binding free energy calculations, and drug-likeness properties predictions to determine their stability, binding affinity, and pharmacokinetic properties. Thus, this study offers valuable insights into the design of sustainable therapeutic plans to control IMNV in shrimp aquaculture coupled with a comprehensive computational framework for the discovery of novel candidate compounds.

## 2 Methods and materials

### 2.1 Data collection and preprocessing

#### 2.1.1 Sequence retrieval, protein structure prediction, and model evaluation

The amino acid sequence of RNA-dependent RNA polymerase (RdRp) from Penaeid shrimp infectious myonecrosis virus (IMNV) was retrieved in FASTA format using the accession number QDN53938.1 (Isolate IMNV_IndAP17) from the National Center for Biotechnology Information (NCBI) protein database (https://www.ncbi.nlm.nih.gov/), and submitted to the AlphaFold server (https://alphafoldserver.com/) powered by AlphaFold 3 for protein structure prediction, as there were no available experimental structures (X-ray or NMR) of RdRp of IMNV in the Protein Data Bank (PDB). The AlphaFold Server is a free, web-based platform from Google DeepMind and Isomorphic Labs that utilizes the AlphaFold 3 (AF3) AI model to predict 3D structures of biomolecular complexes, including proteins, DNA, RNA, and ligands (Gomez and Baylink, 2025). Furthermore, predicted local distance difference test (pLDDT) and predicted aligned error (PAE) analyses were used to evaluate the overall confidence and reliability of the predicted structure (Carugo, 2023; Elfmann and Stülke, 2023). Afterward, the predicted models were evaluated using SAVES v6.1 (https://saves.mbi.ucla.edu/) and ProSA-web (https://prosa.services.came.sbg.ac.at/prosa.php) servers. Then, the Swiss-PdbViewer (SPDBV) v4.10 (Guex and Peitsch, 1997) was appointed to minimize the selected model energy. The predicted IMNV RdRp structures were structurally aligned using PyMOL, and pairwise structural RMSD values were calculated based on the aligned atomic coordinates.

#### 2.1.2 Conserved RdRp motif identification and structural mapping

Based on the structural evaluation, model_1 was selected for further structural annotation and computational analyses. The IMNV RdRp sequence corresponding to ORF2 was analyzed using the NCBI Conserved Domain Database (CDD) (https://www.ncbi.nlm.nih.gov/Structure/cdd/cdd.shtml) through the CD-Search tool to confirm the presence and extent of the conserved viral RNA-dependent RNA polymerase domain (Marchler-Bauer and Bryant, 2004; Wang et al., 2023). Sequence similarity of the IMNV ORF2 protein to *Totiviridae* RdRps was further assessed using BLASTP (https://blast.ncbi.nlm.nih.gov/Blast.cgi?PROGRAM=blastp&PAGE_TYPE=BlastSearch&LINK_LOC=blasthome) against the NCBI protein database, with the search restricted to *Totiviridae* sequences. IMNV ORF2 was previously reported to encode a putative RdRp containing eight conserved motifs characteristic of totiviruses, as demonstrated by alignment with RNA polymerases from members of the family *Totiviridae* (Poulos et al., 2006). The correspondence between the IMNV/Totiviridae motif numbering and canonical viral RdRp motif nomenclature was established based on published structural and sequence comparisons (Bruenn, 2003). The conserved motif sequences were mapped to the amino-acid sequence used for AlphaFold 3 structure prediction, and the corresponding residue positions were recorded. The identified residues were subsequently mapped onto the selected AlphaFold 3 model_1 structure and visualized using BIOVIA Discovery Studio Visualizer. The conserved RdRp motifs were mapped onto the model_1 structure and visualized using BIOVIA Discovery Studio Visualizer.

#### 2.1.3 Marine-derived compounds collection

The Seaweed Metabolite Database (SWMD) is a free, open-source online resource (https://www.swmd.co.in/) that provides free, searchable information on marine seaweed compounds and their biological activities (Davis and Vasanthi, 2011; Al Sharie et al., 2020). It focuses on bioactive secondary metabolites, mainly from Laurencia red algae, to support pharmaceutical drug discovery and cheminformatics analysis, covering 25 structural and pharmacological fields. It offers information on the structural diversity of seaweed metabolites, allowing researchers to analyze the chemical space of seaweed metabolites (*e.g.*, using scaffold diversity analysis). Structural information is available by searching by accession number, chemical name, seaweed name (*e.g.*, Laurencia), SMILES, or InChI. Finally, the all the secondary metabolites were retrieved form the ‘Download’ module of the SWMD database in PDB format (Davis and Vasanthi, 2011).

### 2.2 Molecular docking and protein-ligand interaction

Molecular docking analyses were carried out using the AutoDock Vina suite of tools integrated in PyRx v0.8 (Trott and Olson, 2010; Alkubaysi et al., 2025) to estimate docking scores and characterize protein-ligand interactions. The downloaded ligand structures were energy-minimized in PyRx and prepared as a ligand library. The database-provided stereochemical structures were retained, and alternative stereoisomers or tautomers were not separately evaluated. The AlphaFold 3 Model 1 structure of the IMNV RdRp was imported into PyRx and prepared as the macromolecule. Blind docking was conducted using AutoDock Vina with a grid box of dimensions X = 73.0260 Å, Y = 78.3107 Å, and Z = 86.1746 Å, centered at X = 0.8456, Y = −0.5980, and Z = 0.8598, encompassing the whole protein surface and allowing the ligands to search across all potential binding sites. Multiple docking poses were generated for each ligand, and the final pose was selected primarily according to the most favorable (most negative) docking score, with RMSD upper-bound and lower-bound values of 0.0 Å used as additional pose-selection criteria. Following docking, protein-ligand interactions were visualized and analyzed using BIOVIA Discovery Studio 2021 Client (Discovery Studio, 2021).

### 2.3 Local pLDDT and PAE analysis and structural confidence of predicted ligand-binding regions

Residue-level pLDDT values were obtained from the model_1 CIF structure file, whereas the PAE matrix was obtained from the corresponding AlphaFold 3 full-data JSON output. Mean pLDDT values were calculated for the residues comprising each of the eight conserved RdRp motif regions. For each motif, the mean intra-region PAE was calculated from the PAE values for residue pairs within that motif. The same procedure was subsequently applied to the sets of residues identified as interacting with the selected ligands in the docking analysis to assess the local structural confidence of the predicted ligand-binding regions.

### 2.4 Predicted catalytic and putative entry-associated regions

Conserved acidic residues and the GDD motif were mapped onto the AlphaFold 3 model_1 structure and examined as candidate components of the polymerase catalytic region. In viral RdRps, Motif A contains the conserved acidic residues of the d-X(2–4)-D pattern, whereas Motif C contains the conserved GDD motif, and these acidic residues contribute to the conserved metal-dependent catalytic mechanism (Shu and Gong, 2016; Venkataraman et al., 2018). Accordingly, IMNV residues D354 and D359 in Motif 4 (canonical Motif A), together with G466-D467-D468 in Motif 6 (canonical Motif C), were annotated as a putative catalytic region, with D354, D467, and D468 considered candidate metal-coordinating residues. The residues involved in the GA002, RC003, and BE012 docking interactions were obtained from the corresponding docking interaction analyses and used as the residue sets for ligand-binding-region confidence assessment. Motif F is located in the fingers subdomain and is associated with interactions with incoming NTPs, whereas Motif G is associated with the template-entry route (Venkataraman et al., 2018). Accordingly, IMNV Motif 3 (residues 291–297), corresponding to canonical Motif F, was annotated as a putative NTP-entry-associated region. Motif 2 (residues 231–237) was located within the finger’s region of the predicted structure, and structural modeling of totivirus polymerases showed that this additional motif is positioned in the fingers domain and interacts canonical Motif G (Lee et al., 2022). Based on this conserved structural organization, the surrounding region was annotated as a putative RNA-template entry-associated region. Because these annotations were derived from a predicted structure rather than an experimentally resolved polymerase-RNA complex, they were considered putative structural annotations rather than experimentally confirmed channels.

### 2.5 Molecular dynamics simulation study

Molecular dynamics (MD) simulations were conducted under defined conditions chosen to represent a saline shrimp aquaculture environment. MD simulations were conducted to assess the structural stability of protein-ligand complexes under varying physiological conditions, utilizing *in silico* approaches to analyze biomolecular behavior through the simulation of atomic interactions over defined time scales (Fu et al., 2018; Badar et al., 2022). MD simulations of the protein-ligand complexes were performed using Desmond v2020.4 (Schrödinger, Academic) on a Linux platform. The simulations employed the OPLS3e force field and the SPC water model within an orthorhombic periodic boundary box, maintaining a minimum solvent buffer distance of 10 Å in all directions. The systems were first neutralized by adding counterions to remove any net charge, followed by the addition of Na⁺ and Cl⁻ ions to achieve a total NaCl concentration of 0.15 M. Also, the total intracellular ionic concentration of a shrimp cell typically sits between 0.12 M and 0.20 M (which is 120 to 200 mM) (Deris et al., 2024; Huang et al., 2026).Thus, this ionic condition was used as a defined simulation condition to represent both of aquaculture environment and the intracellular ionic environment of infected shrimp cells. Subsequently, energy minimization was performed to resolve steric clashes. The system was gradually heated to 300 K under NVT conditions and subsequently equilibrated under NPT conditions at 300 K and 1 atm to stabilize density and pressure, aligning with typical shrimp habitat temperatures (Kır et al., 2023). Long-range electrostatic interactions were treated using the particle mesh Ewald (PME) method, and all bonds involving hydrogen atoms were constrained, enabling a 2-femtosecond (fs) integration time step. Production simulations were then performed under NPT conditions (300 K, 1 atm) for 200 ns, yielding 5000 frames per trajectory. A 200 ns molecular dynamics (MD) simulation was performed for each of the four systems, comprising one apo protein and three protein-ligand complexes. A single trajectory was generated for each system. The mean ± SD values were calculated from the values obtained at each frame throughout the 200 ns simulation. The OPLS3e force field was selected due to its suitability for drug-like molecules and its compatibility with protein-ligand simulations (Roos et al., 2019). Trajectory analysis was performed to assess the stability and flexibility of the protein-ligand complexes, employing metrics such as root mean square deviation (RMSD), root mean square fluctuation (RMSF), radius of gyration (Rg), and hydrogen bond (H-bond).

### 2.6 Post-simulation MM-GBSA analysis

Then, the MM-GBSA-based binding free energy (ΔG_bind) of the selected complex was calculated with the gmx_MMPBSA package (Valdés-Tresanco et al., 2021; Miller et al., 2012). This approach integrates van der Waals, electrostatic, and solvation energy components, along with solvent-accessible surface area (SASA) contributions, providing a thermodynamic estimate of ligand affinity. The Desmond trajectory files were converted to GROMACS-compatible formats using Schrödinger utilities, while the corresponding topology files were generated through InterMol conversion of *.cms files to *.gro and *.top formats (Shirts et al., 2017). The MM-GBSA calculations were performed using trajectory snapshots obtained throughout the entire 200 ns MD simulation for each protein-ligand complex. The same frame-selection protocol was applied to all complexes.

### 2.7 Pharmacokinetics analysis

ADMET refers to absorption, distribution, metabolism, excretion, and toxicity, which are critical parameters in the drug development process (Chibuye et al., 2024; Tsaioun et al., 2016). The ADME profiles were evaluated using the SwissADME (http://www.swissadme.ch/), and toxicity profiles were evaluated from ADMETlab 3.0 (https://admetlab3.scbdd.com/), ProTox-3.0 (https://tox.charite.de/protox3), and Deep-PK (https://biosig.lab.uq.edu.au/deeppk/) web-based platforms (Myung et al., 2024; Fu et al., 2024; Banerjee et al., 2024; Daina et al., 2017). The SMILES representations of the compounds were obtained and used as input for ADME and toxicity prediction. In this study, ADME-related parameters obtained from SwissADME included Consensus Log *P_o/w_*, water solubility (Log S) (ESOL), hydrogen bond acceptors and donors, topological polar surface area (TPSA), Lipinski rule violations, and bioavailability score. Toxicity parameters were predicted using ADMETlab 3.0, including bioconcentration factor (BCF), LC_50_ for Fathead minnow (LC_50_ FM), LC_50_ for *Daphnia magna* (LC_50_ DM), aquatic toxicity rule, and half-life of drugs (T_1/2_). Additionally, ecotoxicity was evaluated using ProTox-3.0, and toxicity to crustaceans was predicted using Deep-PK.

## 3 Results

### 3.1 Data collection and preprocessing

#### 3.1.1 Sequence retrieval, protein structure prediction, and model evaluation

**Fig. 1:**
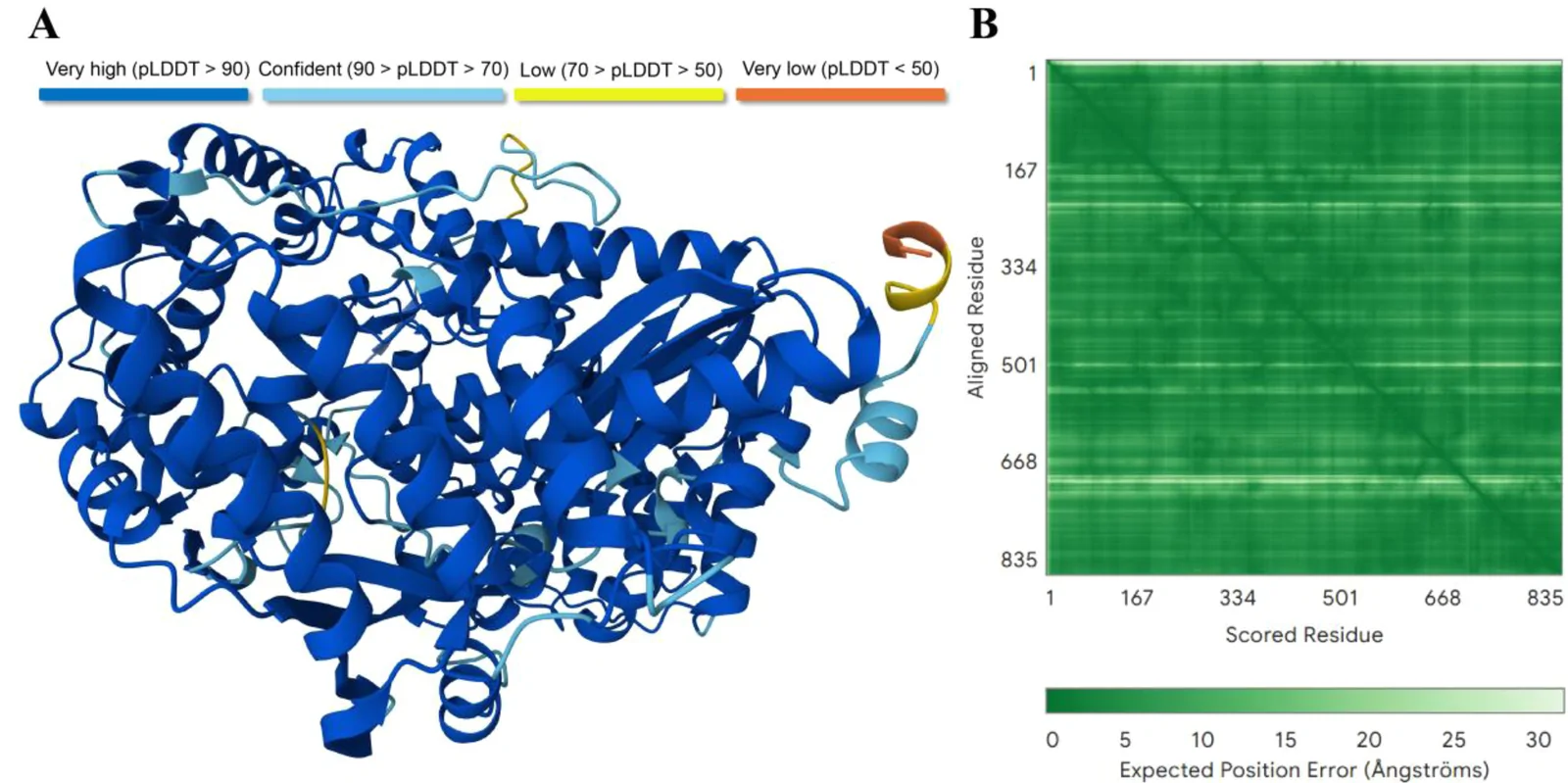
Overall confidence assessment of the predicted structure using (A) pLDDT and (B) PAE analyses.

The pLDDT and PAE analyses of the predicted structure were illustrated (Fig. 1). The predicted structure exhibited predominantly very high pLDDT scores (pLDDT > 90) and high-confidence regions (90 > pLDDT > 70), with only a few residues showing low confidence, indicating high overall prediction accuracy (Fig. 1**A**). The PAE plot showed overall low error values, suggesting reliable residue positioning and high confidence in the predicted structure (Fig. 1**B**).

**Fig. 2:**
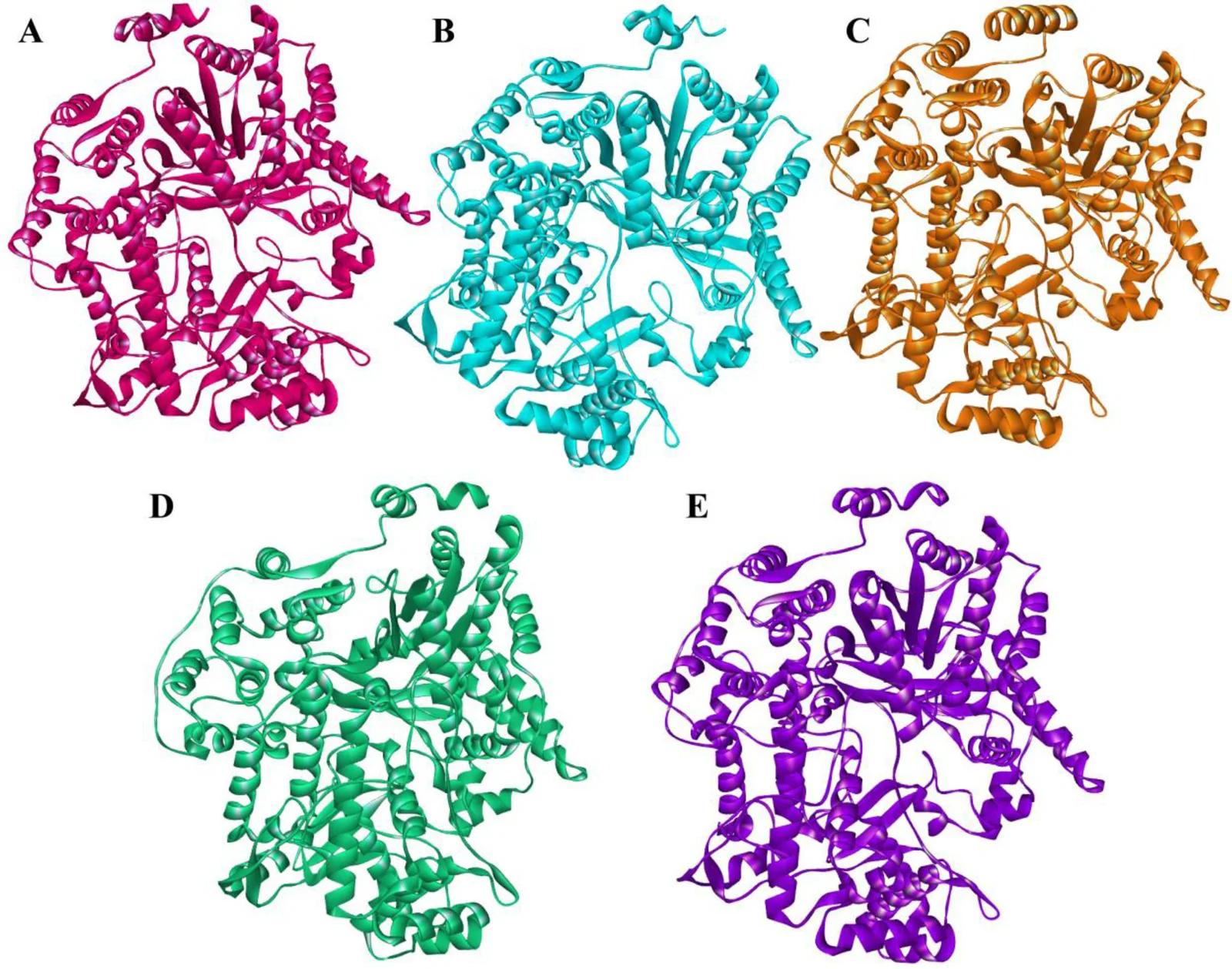
Structural illustrations of the five predicted models: (A) model_0 (Red), (B) model_1 (Cyan), (C) model_2 (Orange), (D) model_3 (Green), and (E) model_4 (Purple), displayed in cartoon representation with different color schemes.

The AlphaFold server predicted five models (model_0, model_1, model_2, model_3, and model_ 4) (Fig. 2). Among the five predicted models, structural quality was evaluated using model ranking score, ERRAT, VERIFY3D, and Z-score analyses (Table 1). In addition, Ramachandran plot assessment was also performed (Table 1 and Fig. 4). All predicted models exhibited an identical predicted template modeling (pTM) value of 0.93.

**Table 1:** Evaluation of the five predicted models using model ranking score, pTM, ERRAT, VERIFY3D, Ramachandran score, and Z-score analyses.

Model	Given Name	Model Ranking Score	pTM Value	ERRAT	Ramachandran Score	VERIFY3D	Z-score
model_0	RdRp_M00	0.94	0.93	94.9878	95.6	82.34	−12.54
model_1	RdRp_M01	0.94	0.93	96.0543	95.6	83.41	−12.58
model_2	RdRp_M02	0.94	0.93	94.9939	95.8	83.17	−12.79
model_3	RdRp_M03	0.94	0.93	95.7002	96.2	81.50	−12.41
model_4	RdRp_M04	0.93	0.93	95.116	96	80.91	−12.51

**Fig. 3:**
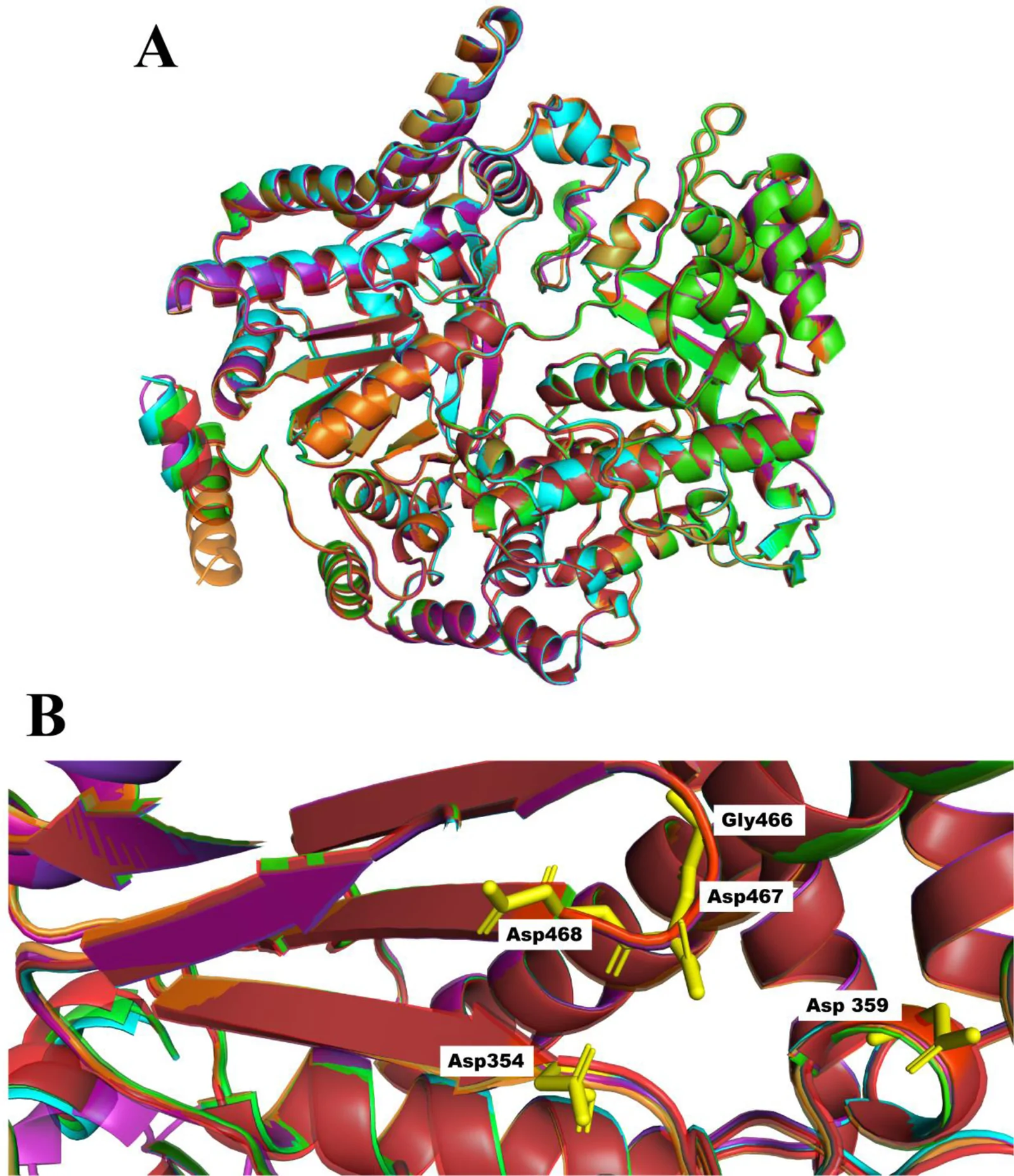
(A) Structural superposition of model_0 (Red), model_1 (Cyan), model_2 (Orange), model_3 (Green), and model_4 (Purple). (B) Close-up comparison of the predicted catalytic region, including Asp354, Asp359, Gly466, Asp467, and Asp468, shown as yellow sticks.

However, the ranking scores of the model_0, model_1, model_2, and model_3 were 0.94, while the model_4 showed a score of 0.93. The validation results showed that model_0, model_1, model_2, model_3, and model_4 exhibited ERRAT scores of 94.9878, 96.0543, 94.9939, 95.7002, and 95.116; Ramachandran most favored region percentages of 95.6%, 95.6%, 95.8%, 96.2%, and 96.0%; VERIFY3D scores of 82.34%, 83.41%, 83.17%, 81.50%, and 80.91%; and Z-scores of −12.54, −12.58, −12.79, −12.41, and −12.51, respectively. Using model_1 as the reference structure, the five predicted IMNV RdRp models showed close structural agreement, with pairwise structural RMSD values ranging from 0.221 to 0.328 Å. The lowest RMSD was observed between model_1 and model_4 (0.221 Å), whereas the highest was observed between model_0 and model_3 (0.328 Å). Overall, the low RMSD values indicated a high degree of structural similarity among the predicted models (Fig. 3). In this study, model_1′s energy was minimized using SPDBV, and the energy-minimized model was named RdRp_model_1 or RdRp_M01. Finally, the SWMD was used for the retrieval of all 1077 compounds in PDB format.

**Fig. 4:**
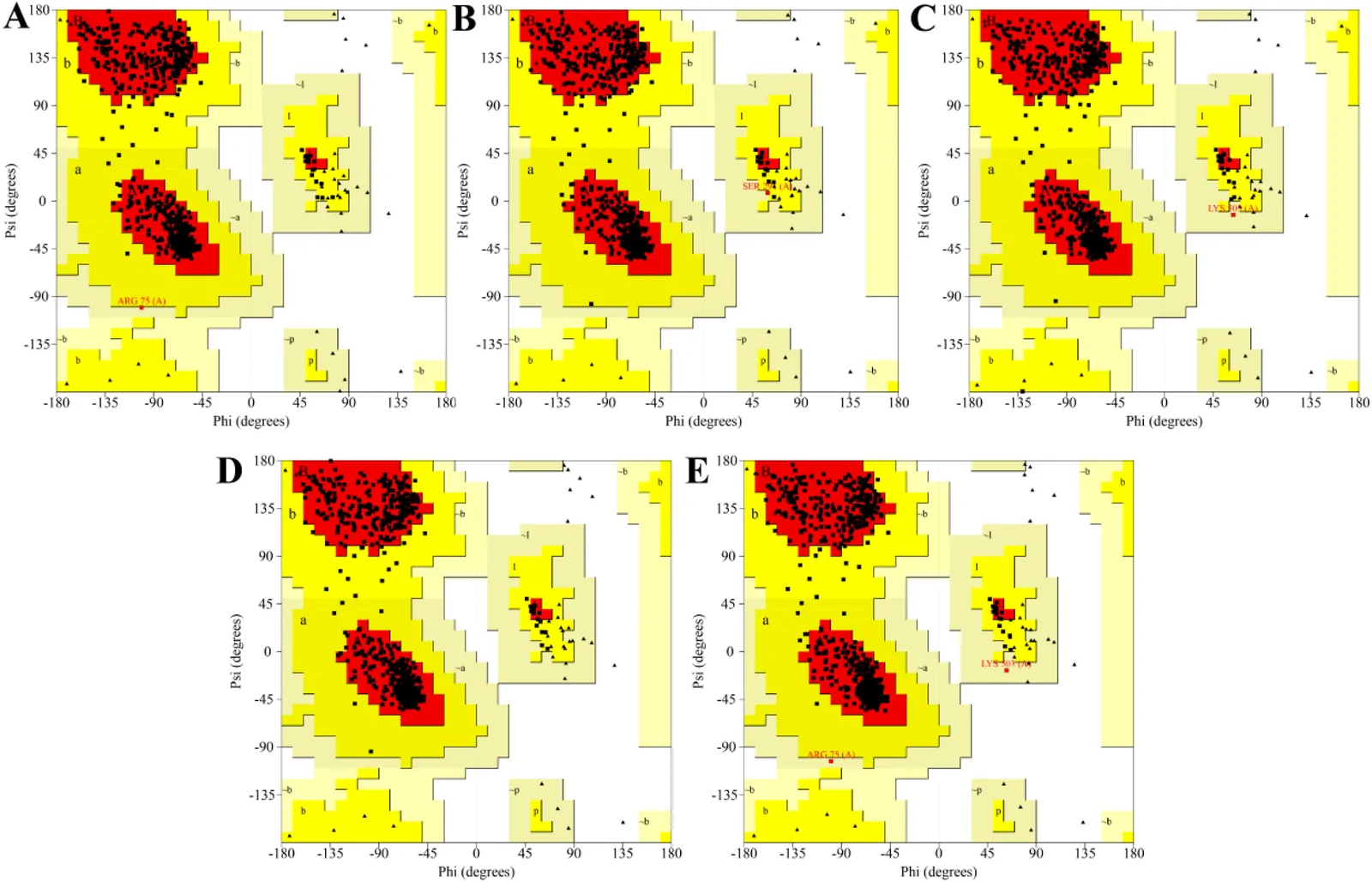
Ramachandran plots and their corresponding statistical analyses for the five predicted models: (A) model_0, (B) model_1, (C) model_2, (D) model_3, and (E) model_4.

#### 3.1.2 Conserved RdRp motif identification and structural mapping

The IMNV ORF2-encoded protein was analyzed using the NCBI Conserved Domain Database through CD-Search. A viral RNA-directed RNA polymerase (RdRP_4) domain was identified spanning residues 157–525, with an E-value of 6.51 × 10^–12^, supporting the presence of a conserved viral RdRp domain within the analyzed protein. In addition, pairwise BLASTP analysis against the top-scoring Totiviridae RdRp hit (UHS72541.1) produced a 1061-bit alignment score with an E-value of 0.0, with 494 of 825 aligned residues identical (59.88%) and 628 of 825 residues showing positive matches (76.12%) (**Table S1**). The alignment contained only one gap and covered approximately 98% of the query sequence, indicating extensive sequence similarity across the protein (**Supplementary 2A and 2B**). These findings were consistent with the previously reported characterization of IMNV ORF2 as a putative RdRp containing motifs characteristic of totiviruses.

### 3.2 Molecular docking and protein-ligand interaction

Molecular docking analyses were conducted between RdRp_M01 and 1077 compounds, resulting in 1032 successful hits, with the highest and lowest docking scores of −13 and −1.0 kcal/mol for GA002 and BD037, respectively (**Table S2**). Among these, the top six ligands: GA002, RL354, RL015, BS067, RC003, and BE012 exhibited binding energies of −13.0, −11.5, −11.0, −10.9, −10.9, and −10.8 kcal/mol, respectively (Table 2). The corresponding 2D structures, including database-defined stereochemical information, and the 3D structures used for docking are provided in the Supplementary Material (**Figures S1** and **S2**, respectively).

**Table 2:** The docking scores of the six chosen compounds, along with their accession number, type, sources, and molecular weight.

Accession Number	Compound Type	Molecular Weight (g/mol)	Docking Score (kcal/mol)	Source Organism	Biological Source
GA002	Avrainvilloside	786.17,358	−13.0	*Avrainvillea nigricans* (Andersen and Taglialatela-Scafati, 2005)	Green Alga
RL354	Dimer of Laurinterol	588.10,819	−11.5	*Laurencia tristicha* (Ji et al., 2008)	Marine Red Alga
RL015	Dimeric sesquiterpene (cyclolaurane-type)	588.41,360	−11.0	*Laurencia microcladia* (Kladi et al., 2006)	Red Algae
BS067	Epitaondiol diacetate	496.67,810	−10.9	*Stypopodium flabelliforme* (Areche et al., 2009)	Marine Brown Alga
RC003	Bromophycolide C	602.39,600	−10.9	*Callophycus serratus* (Kubanek et al., 2006)	Fijian Red Alga
BE012	6,6′-Bieckoll	742.54,908	−10.8	*Ecklonia cava* (Li et al., 2009)	Marine Brown Alga

**Fig. 5:**
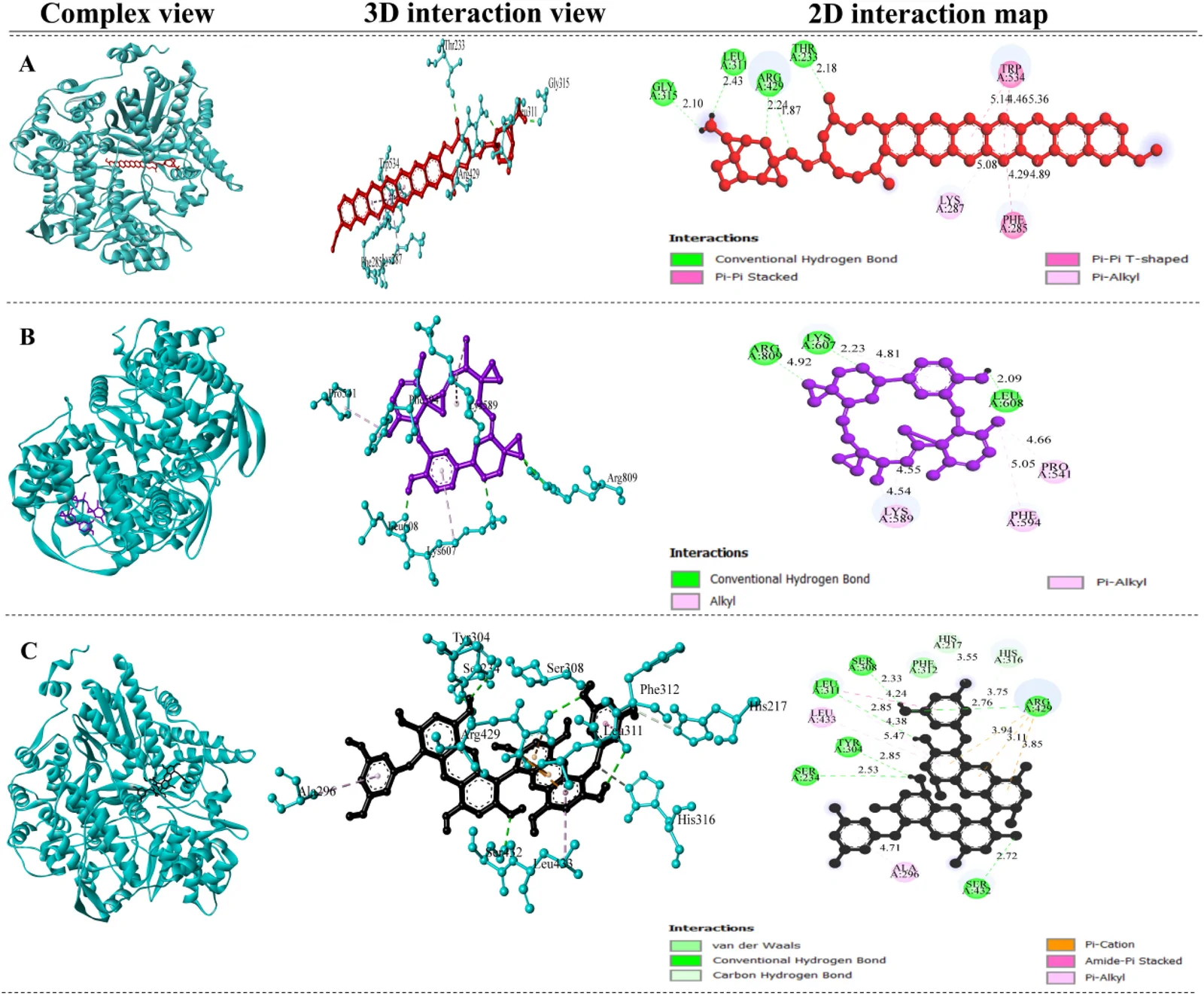
Complex view (Left), 3D interaction view (Middle), and 2D interaction map (Right) of the protein RdRp_M01 interactions with ligands: (A) GA002 (Red), (B) RC003 (Purple), and (C) BE012 (Black).

The molecular interactions between RdRp_M01 and the six selected compounds were visualized using the BIOVIA Discovery Studio Visualizer. The receptor protein and ligands formed various interactions, including hydrogen bonds, hydrophobic interactions, and van der Waals forces, with specific bond distances observed between the amino acid residues of the receptor and the ligands. GA002, RC003, and BE012 exhibited comparatively diverse interaction profiles, including hydrogen-bond and hydrophobic interactions (Fig. 5), whereas RL354, RL015, and BS067 showed predominantly alkyl and π-alkyl interactions (**Figure S3**). Thus, based on the combination of favorable docking scores and interaction profiles, GA002, RC003, and BE012 were selected for further computational studies, including MD simulation and ADMET analysis. GA002 exhibited multiple interactions with the target RdRp_M01 protein, including conventional hydrogen bonds with GLY A:315, LEU A:311, ARG A:429, and THR A:233; π-π T-shaped interaction with PHE A:285; π-π stacked interaction with TRP A:534; and π-alkyl interaction with LYS A:287 (Fig. 5**A**). RC003 formed interactions with conventional hydrogen bonds with ARG A:809, LYS A:607, and LEU A:608; alkyl interactions with LYS A:589 and PRO A:541; and a π-alkyl interaction with PHE A:594 (Fig. 5**B**). BE012 showed conventional hydrogen bonds with SER A:234, TYR A:304, LEU A:311, SER A:308, ARG A:429, and SER A:432; van der Waals interactions with PHE A:312; and carbon-hydrogen bonds with HIS A:217 and HIS A:316 (Fig. 5**C**). The GA002-interacting residues included residue 233 within Motif 2 (231–237) and residue 429 within Motif 5 (426–446). The BE012-interacting residues included residue 234 within Motif 2, residue 296 within Motif 3/canonical motif F (291–297), and residues 429 and 432 within Motif 5. In contrast, none of the RC003-interacting residues overlapped the mapped conserved motif regions. None of the ligand-interacting residue sets directly overlapped the predicted catalytic residues D354, D359, D467, and D468. Therefore, the blind-docking results were interpreted as predicted ligand-binding interactions rather than evidence of direct catalytic-site binding or functional inhibition. A detailed summary of the docking-interacting residues and their relationship to the mapped functional regions is provided in **Table S3**.

### 3.3 Local pLDDT and PAE analysis and structural confidence of predicted ligand-binding regions

The IMNV RdRp sequence contained eight conserved polymerase motifs corresponding to the motif framework reported in the published IMNV/*Totiviridae* RdRp alignment. The correspondence with canonical viral RdRp motifs was summarized (**Table S4**). The corresponding regions in the AlphaFold 3 model_1 sequence were mapped as Motif 1 (165–172), Motif 2 (231–237), Motif 3 (291–297), Motif 4 (354–363), Motif 5 (426–446), Motif 6 (466–468), Motif 7 (506–509), and Motif 8 (516–523). Local structural confidence varied among the motifs, with mean pLDDT values ranging from 72.61 to 93.98 and mean intra-region PAE values ranging from 0.88 to 3.21 Å (**Table S5**). Most motifs showed high local confidence, with mean pLDDT values above 87, whereas Motif 2 displayed comparatively lower confidence (pLDDT 72.61; mean intra-region PAE 3.21 Å). In contrast, Motif 6 (G466-D467-D468) showed high local confidence (pLDDT 91.05; mean intra-region PAE 0.88 Å), indicating high local structural confidence for this conserved polymerase region.

The local structural confidence of the predicted ligand-binding regions was assessed for the three docked complexes GA002, RC003, and BE012, respectively. The GA002-interacting residues showed a mean pLDDT of 84.53 and a mean pocket PAE of 3.43 Å, whereas the RC003 and BE012 interacting residue sets showed mean pLDDT values of 87.15 and 87.51, with corresponding mean pocket PAE values of 2.55 Å and 2.51 Å, respectively (**Table S6**). These results indicated moderate to high local structural confidence across the predicted ligand-interacting regions, with the GA002 binding region showing the comparatively lowest confidence among the three complexes.

### 3.4 Predicted catalytic and putative entry-associated regions

The conserved acidic residues within the predicted catalytic region were mapped onto the AlphaFold 3 model_1 structure, including Asp354, Asp359, Asp467, and Asp468 (**Figure S4**). The sequence region containing Asp354 and Asp359 conforms to the conserved d-X(2–4)-D acidic-residue pattern of RdRp motif A. Based on the conserved RdRp catalytic architecture, Asp354, Asp467, and Asp468 were considered candidate metal-coordinating acidic residues. In addition, Gly466-Asp467-Asp468 (G466-D467-D468) constitutes the conserved GDD motif (Motif 6) of the polymerase. Based on the conserved RdRp catalytic architecture, Asp354, Asp467, and Asp468 were considered candidate metal-coordinating acidic residues. The GDD-containing region showed high local structural confidence, with a mean pLDDT of 91.05 and a mean intra-region PAE of 0.88 Å (**Table S4**). Together, the conserved acidic motif architecture and high local structural confidence support the annotation of this region as a predicted RdRp catalytic center.

The IMNV Motif 3 region (residues 291–297), corresponding to canonical motif F, was annotated as a putative NTP-entry-associated region based on the conserved structural architecture of viral RdRps (**Figure S5A**). A second region, corresponding to IMNV Motif 2 (residues 231–237), was annotated as a putative RNA-template entry-associated region based on the conserved structural organization of the predicted polymerase (**Figure S5B**). Because the AlphaFold 3 model does not contain experimentally resolved RNA or nucleotide ligands, these annotations were considered putative structural inferences rather than experimentally confirmed channels.

### 3.5 Molecular dynamics (MD) simulation

**Fig. 6:**
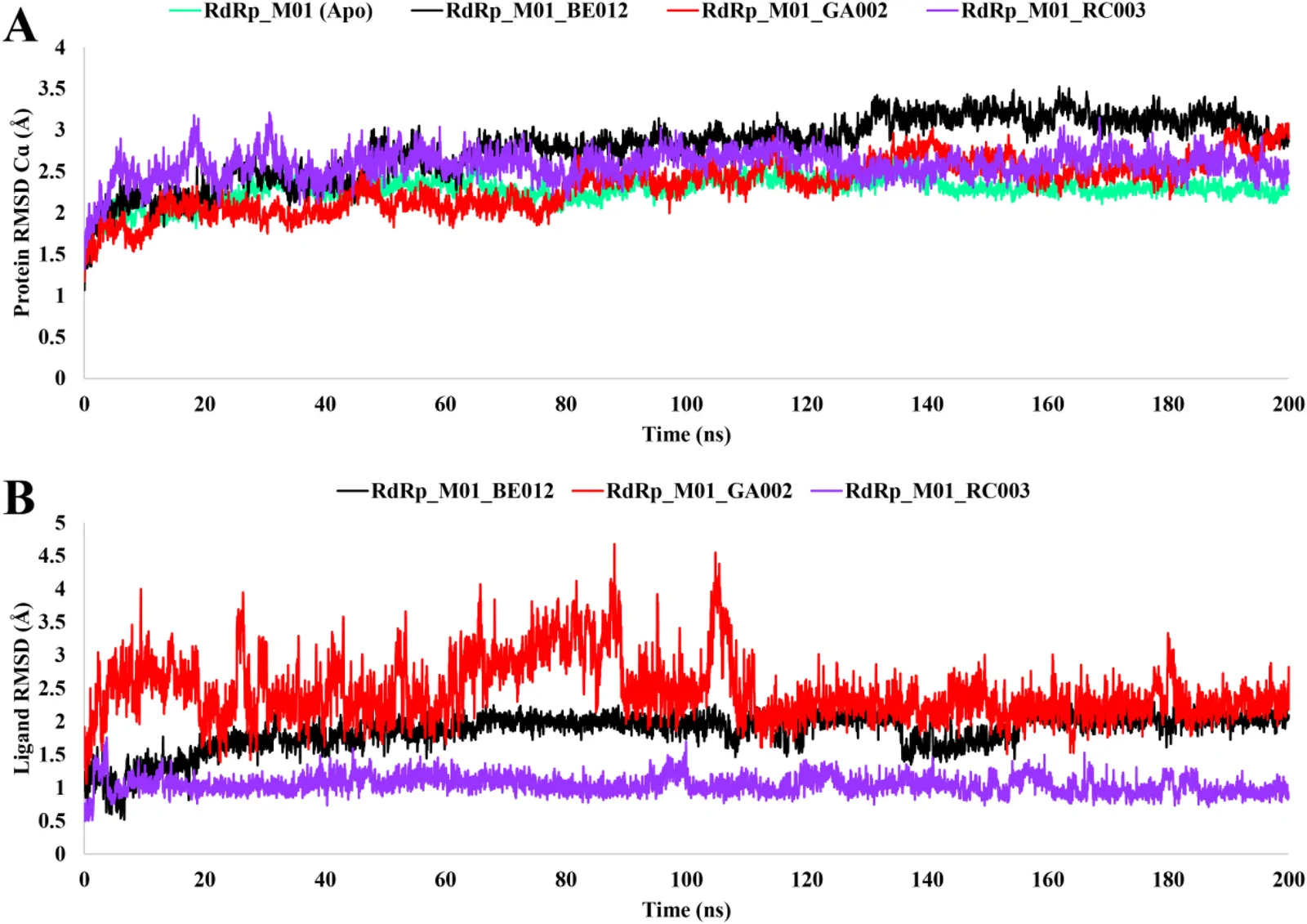
Molecular dynamics simulations of the (A) Protein RMSD and (B) Ligand RMSD, where the unbound protein Apo (RdRp_M01) (Light green), and the complexes GA002 (Light red), RC003 (Purple), and BE012 (Black) were illustrated.

**Fig. 7:**
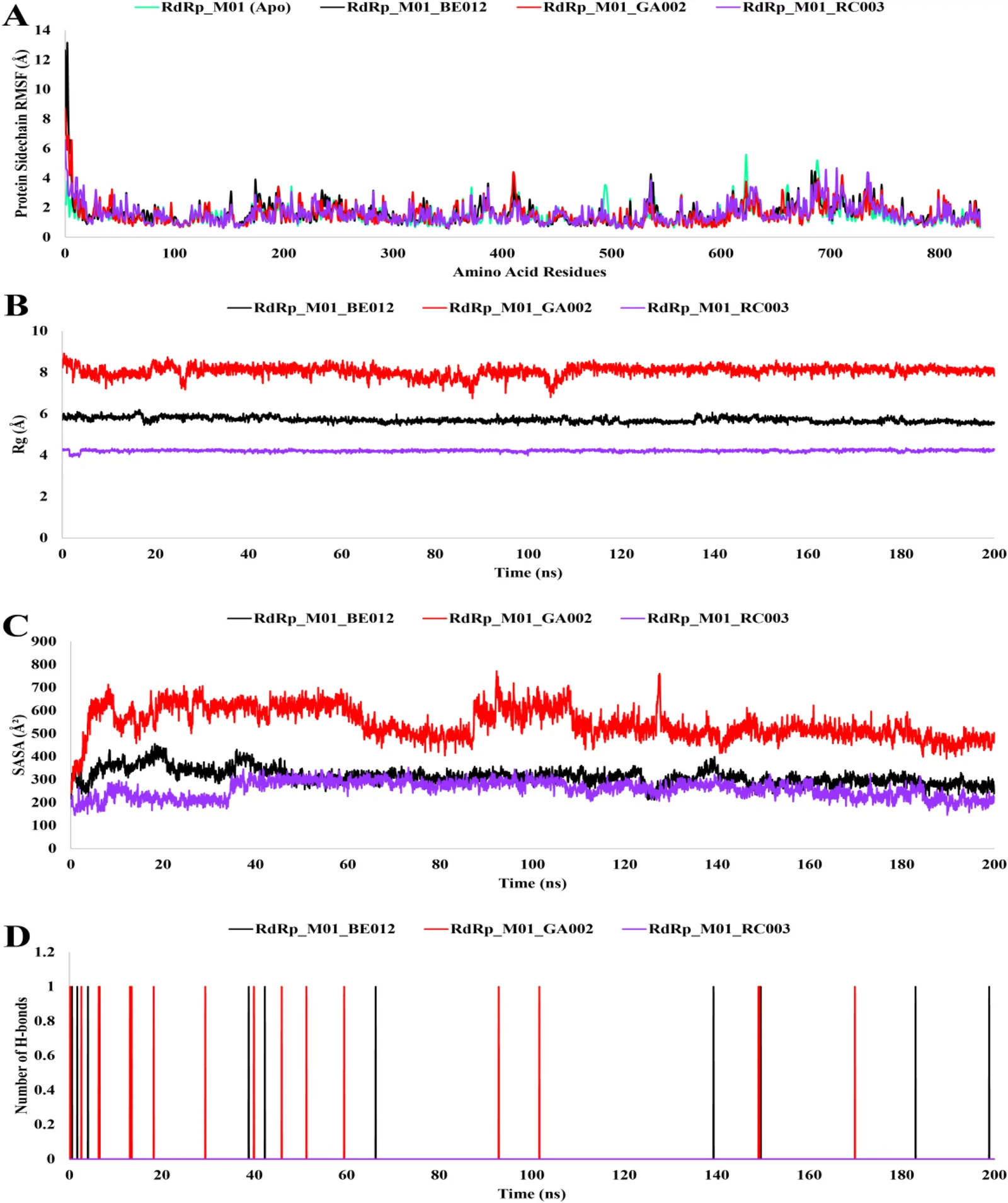
Molecular dynamics simulations of the (A) Protein Sidechain RMSF, (B) Rg, (C) SASA, and (D) Number of H-bonds, where the unbound protein Apo (RdRp_M01) (Light green), and the complexes GA002 (Light red), RC003 (Purple), and BE012 (Black) were depicted.

**Fig. 8:**
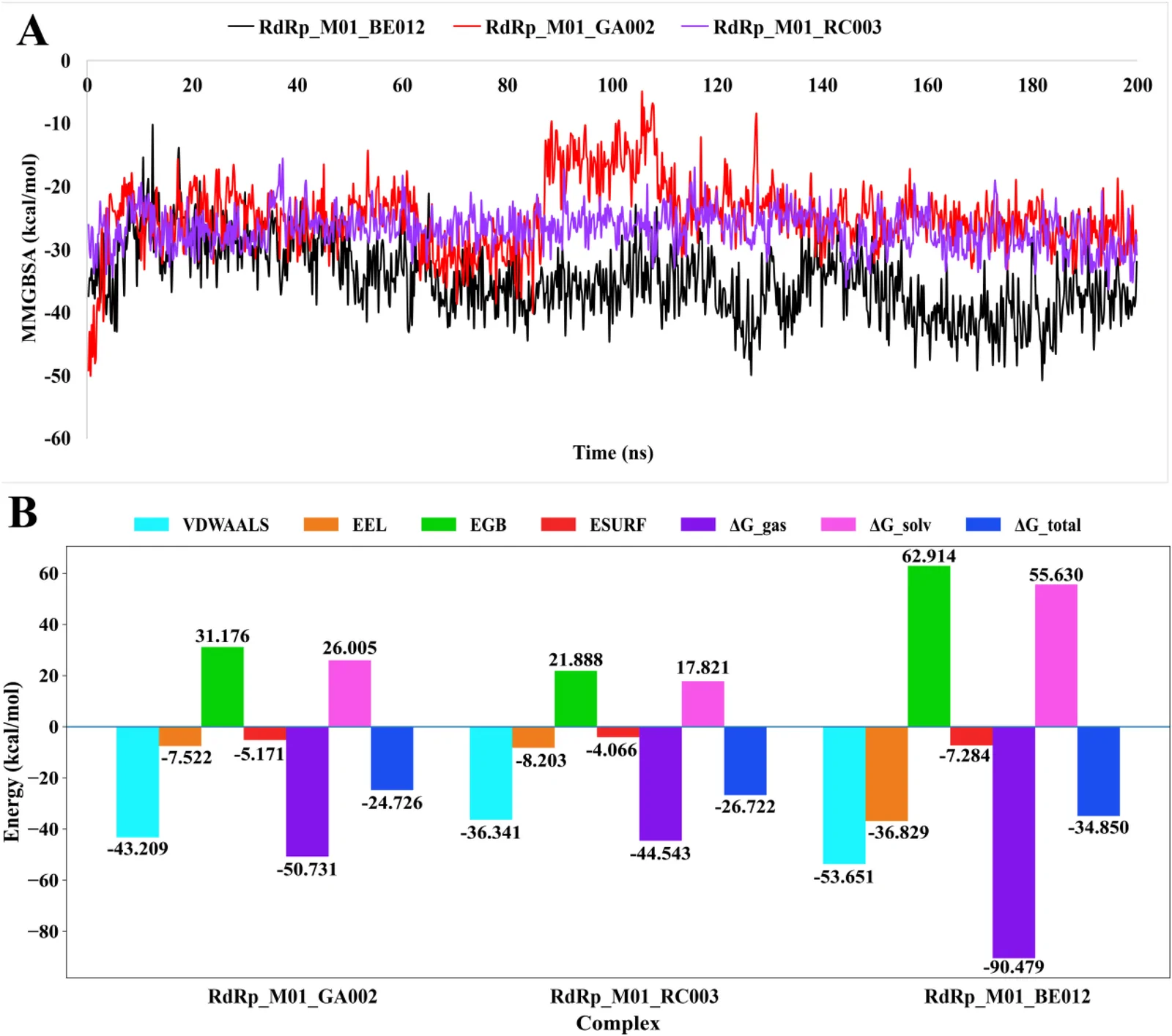
Post-simulation MMGBSA analysis of the RdRp_M01_GA002 (Light red), RdRp_M01_RC003 (Purple), and RdRp_M01_BE012 (Black) complexes. (A) MMGBSA binding free energy profile. (B) Energy decomposition bar chart showing the contributions of VDWAALS (Light blue), EEL (Orange), EGB (Green), ESURF (Red), ΔG_gas (Purple), ΔG_solv (Pink), and ΔG_total (Dark blue).

In this study, a 200 ns MD simulation was conducted to evaluate the conformational stability and steady-state behavior of the protein-ligand complexes. The simulations were performed on the three selected complexes: RdRp_M01_GA002, RdRp_M01_RC003, and RdRp_M01_BE012 (Figs. 6, 7, and 8). The results of the MD simulations were analyzed based on RMSD, RMSF, Rg, SASA, H-bond, and post-simulation MMGBSA. The summarized data of MD simulations were represented (Table 3), where all values were expressed as mean ± standard deviation (SD).

**Table 3:** The average values with standard deviation (SD) for different MD simulation parameters, calculated from the MD simulation trajectories.

Analysis	Protein-ligand Complex	Mean ± SD
Protein RMSD (Å)	Apo (RdRp_M01)	2.28 ± 0.15
	RdRp_M01_GA002	2.34 ± 0.31
	RdRp_M01_RC003	2.58 ± 0.18
	RdRp_M01_BE012	2.80 ± 0.37
Ligand RMSD (Å)	RdRp_M01_GA002	2.46 ± 0.45
	RdRp_M01_RC003	1.05 ± 0.14
	RdRp_M01_BE012	1.85 ± 0.28
RMSF (Å)	Apo (RdRp_M01)	1.50 ± 0.66
	RdRp_M01_GA002	1.56 ± 0.78
	RdRp_M01_RC003	1.58 ± 0.70
	RdRp_M01_BE012	1.67 ± 0.93
Rg (Å)	RdRp_M01_GA002	8.07 ± 0.24
	RdRp_M01_RC003	4.21 ± 0.05
	RdRp_M01_BE012	5.70 ± 0.12
SASA (Å²)	RdRp_M01_GA002	544.92 ± 69.04
	RdRp_M01_RC003	256.71 ± 37.31
	RdRp_M01_BE012	312.29 ± 34.32
H-bond	RdRp_M01_GA002	0.0044 ± 0.0662
	RdRp_M01_RC003	0.0000 ± 0.0000
	RdRp_M01_BE012	0.0020 ± 0.0447
Post-simulation MMGBSA (kcal/mol)	RdRp_M01_GA002	−24.70 ± 5.58
	RdRp_M01_RC003	−26.72 ± 3.06
	RdRp_M01_BE012	−34.85 ± 5.91

#### 3.5.1 Protein’s backbone RMSD

RMSD measures the average displacement of atomic positions relative to a reference structure, providing insight into structural variations, which is widely used to monitor conformational changes of proteins or ligands throughout simulations or docking studies in protein-ligand complexes (Isa and Kappo, 2025; Puthanveedu and Muraleedharan, 2022). The structural departure from the unbound or Apo (RdRp_M01) protein was evaluated by calculating RMSD values for the three selected ligands (Fig. 6**A**). The RMSD values for the Apo (RdRp_M01) protein and its complexes were 2.28 ± 0.15 Å (Apo), 2.34 ± 0.31 Å (RdRp_M01_GA002), 2.58 ± 0.18 Å (RdRp_M01_RC003), and 2.80 ± 0.37 Å (RdRp_M01_BE012). The RMSD profiles showed consistent values across all systems throughout the simulation.

#### 3.5.2 Ligand RMSD

Ligand RMSD represents the average positional differences between ligand atoms across conformations and is commonly used to assess the similarity or variation between binding poses (Velázquez-Libera et al., 2020). Ligand RMSD was calculated using the Desmond SID Lig fit Prot metric after protein-backbone fitting to the initial trajectory frame, using ligand heavy atoms. The RMSD of the three selected ligands was analyzed (Fig. 6**B**). The ligand RMSD values for GA002, RC003, and BE012 were 2.46 ± 0.45 Å, 1.05 ± 0.14 Å, and 1.85 ± 0.28 Å, respectively. The RMSD profiles of all ligands remained relatively consistent throughout the simulation, with differences observed in the magnitude of deviation among the three ligands.

#### 3.5.3 Root mean square fluctuation (RMSF)

RMSF is a vital metric in MD simulation used to evaluate the flexibility and local motion of amino acid residues by quantifying how much each residue deviates from its average position over time (Surti et al., 2020; Shahraki et al., 2016). The RMSF analysis was performed to evaluate the flexibility of individual amino acid residues of protein RdRp_M01 (Apo) upon binding with the selected ligands GA002, RC003, and BE012 (Fig. 7**A**). The RMSF values for the Apo protein, RdRp_M01_GA002, RdRp_M01_RC003, and RdRp_M01_BE012 were 1.50 ± 0.66 Å, 1.56 ± 0.78 Å, 1.58 ± 0.70 Å, and 1.67 ± 0.93 Å, respectively. The RMSF profiles showed comparable fluctuation patterns across all systems, with slight variations in the magnitude of residue mobility. Residue-level RMSF analysis revealed distinct fluctuation patterns at several positions, including residues Ile410, Pro536, Pro623, Thr688, Glu696, and Glu706. Notable differences between the apo and ligand-bound systems were observed in the 620–710 region, particularly at residues Glu696 and Glu706 in the RC003 complex.

#### 3.5.4 Radius of gyration (Rg)

Rg is a pivotal structural parameter in MD simulations that quantifies the compactness of a protein-ligand complex over time (Rampogu et al., 2022; He et al., 2025). The Rg profiles of the three complexes were analyzed to evaluate structural compactness (Fig. 7**B**). The Rg values for RdRp_M01_GA002, RdRp_M01_RC003, and RdRp_M01_BE012 were 8.07 ± 0.24 Å, 4.21 ± 0.05 Å, and 5.70 ± 0.12 Å, respectively. The Rg profiles remained relatively stable throughout the simulation, with observable differences in the magnitude of compactness among the complexes.

#### 3.5.5 Solvent accessible surface area (SASA)

SASA provides fundamental insights into protein-ligand interactions by quantifying residue exposure, identifying potential binding sites, and monitoring structural changes upon ligand binding (Gromiha and Ahmad, 2005; Sraphet and Javadi, 2022). The SASA of the protein in complex with ligands GA002, RC003, and BE012 was analyzed (Fig. 7**C**). The SASA values for the RdRp_M01_GA002, RdRp_M01_RC003, and RdRp_M01_BE012 complexes were 544.92 ± 69.04 Å², 256.71 ± 37.31 Å², and 312.29 ± 34.32 Å², respectively. The SASA profiles remained relatively consistent throughout the simulation, with noticeable differences in surface exposure among the complexes.

#### 3.5.6 Hydrogen bond (H-bond)

H-bonds contribute to stability, but overall structural stability is governed by multiple non-covalent interactions, including hydrophobic and van der Waals forces (Lins and Brasseur, 1995; Pace et al., 2014). H-bonding interactions in the RdRp_M01_GA002, RdRp_M01_RC003, and RdRp_M01_BE012 complexes were analyzed (Fig. 7**D**). The H-bond values for the RdRp_M01_GA002, RdRp_M01_RC003, and RdRp_M01_BE012 complexes were 0.0044 ± 0.0662, 0.0000 ± 0.0000, and 0.0020 ± 0.0447, respectively. The H-bond profiles showed very low average values across all protein-ligand complexes, with noticeable differences in the extent of interaction among the complexes.

#### 3.5.7 Post-simulation MM-GBSA analysis

Binding free energies were estimated using gmx_MMPBSA based on GROMACS-compatible trajectories obtained from Desmond simulations. The post-simulation MMGBSA was employed to estimate the binding free energies of the three selected RdRp_M01_GA002, RdRp_M01_RC003, and RdRp_M01_BE012 complexes from the 200 ns simulation trajectory (Fig. 8). The simulation showed stable behavior with no major fluctuations during 0–87 ns, minor fluctuations between 88–137 ns, renewed stability from 137–155 ns, brief increased fluctuations between 156–184 ns, and the complexes remained stable again until 200 ns (Fig. 8**A**). The post-simulation MMGBSA analysis revealed average binding free energy values of −24.70 ± 5.58 kcal/mol for RdRp_M01_GA002, −26.72 ± 3.06 kcal/mol for RdRp_M01_RC003, and −34.85 ± 5.91 kcal/mol for RdRp_M01_BE012, calculated over the simulation trajectory. The ΔG_total values of RdRp_M01_GA002, RdRp_M01_RC003, and RdRp_M01_BE012 complexes were −24.726, −26.722, and −34.850 kcal/mol, respectively (Fig. 8**B**).

### 3.6 Pharmacokinetics analysis

The ADMET properties of the three selected ligands were predicted using SwissADME, ADMETlab 3.0, ProTox-3.0, and Deep-PK, and the results were summarized (Table 4). The consensus Log *P_o/w_* values were 9.91 for GA002, 5.05 for RC003, and 3.20 for BE012. Water solubility (Log S) (ESOL) indicated that GA002 was insoluble (−11.06), while RC003 (−7.03) and BE012 (−7.52) were classified as poorly soluble. Hydrogen-bond acceptors were 10, 5, and 18 for GA002, RC003, and BE012, respectively, while hydrogen-bond donors were 4, 3, and 12. The topological polar surface area (TPSA) values were 157.77 Å² (GA002), 86.99 Å² (RC003), and 298.14 Å² (BE012). Lipinski rule violations were observed as follows: GA002 showed one violation (molecular weight > 500), RC003 showed two violations (molecular weight > 500 and MLOGP > 4.15), and BE012 showed three violations (molecular weight > 500, NorO > 10, and NHorOH > 5). The bioavailability scores were 0.55 for GA002 and 0.17 for both RC003 and BE012. The predicted half-life (T_1/2_) values were 6.509 h (intermediate-short) for GA002, 2.001 h (short) for RC003, and 4.763 h (intermediate-short) for BE012. The bioconcentration factor (BCF) values were −0.415 for GA002, 0.902 for RC003, and 0.886 for BE012. For aquatic toxicity, LC_50_ values for fathead minnow (LC_50_ FM) were 3.413 (GA002), 4.673 (RC003), and 3.942 (BE012). LC_50_ values for *Daphnia magna* (LC_50_ DM) were 7.21 for GA002, 5.314 for RC003, and 4.61 for BE012. Aquatic toxicity rule assessment showed 0 alerts for GA002, 3 alerts for RC003, and 0 alerts for BE012. Ecotoxicity predictions indicated inactive profiles for all three compounds, with values of 0.61 (GA002), 0.53 (RC003), and 0.50 (BE012). The crustacean-specific model predicted GA002 and BE012 as toxic with high confidence, whereas RC003 was predicted as safe with low confidence.

**Table 4:** ADME, pharmacokinetic, drug-likeness, and toxicity profiles of the three selected compounds.

Parameter	GA002	RC003	BE012
Consensus Log *P*_o/w_	9.91	5.05	3.20
Water Solubility Log S (ESOL)	−11.06; Insoluble	−7.03; Poorly Soluble	−7.52; Poorly Soluble
Hydrogen Bond Acceptors	10	5	18
Hydrogen Bond donors	4	3	12
TPSA (Å²)	157.77	86.99	298.14
Lipinski Violations	1	2	3
Bioavailability Score	0.55	0.17	0.17
Half-life of Drugs (T_1/2_) (h)	6.509; Intermediate Short	2.001; Short	4.763; Intermediate Short
Bioconcentration Factors (BCF)	−0.415	0.902	0.886
LC_50_ FM (Fathead minnow)	3.413	4.673	3.942
LC_50_ DM (*Daphnia magna*)	7.21	5.314	4.61
Aquatic Toxicity Rule	0 Alert	3 Alerts	0 Alert
Ecotoxicity	0.61 (Inactive)	0.53 (Inactive)	0.50 (Inactive)
Crustacean Toxicity	1 (Toxic; High Confidence)	0.496 (Safe; Low Confidence)	1 (Toxic; High Confidence)

## 4 Discussion

Infectious Myonecrosis Virus (IMNV) remains one of the major viral threat to shrimp aquaculture, causing severe tissue damage, high mortality rates, and considerable economic losses worldwide (Nolasco-Alzaga et al., 2025; Sunarto and Naim, 2016). Currently, there is no approved therapeutic against IMNV in shrimp by the FDA or other major regulatory bodies, highlighting the urgent need for the development of effective antiviral interventions. Marine-derived compounds, particularly seaweed metabolites, represent a rich source of bioactive molecules that have been widely explored for various diseases and drug discovery applications (Pereira, 2026; Rosa et al., 2020; Gajjar et al., 2025). Fucoidan from *Sargassum wightii*, sulfated galactans from *Gracilaria fisheri*, and hot-water extracts of *Sargassum* spp. showed antiviral effects against WSSV in shrimp, including reduced viral load, infectivity, and mortality (Sivagnanavelmurugan et al., 2012; Wongprasert et al., 2014; Immanuel et al., 2010). This study integrates an *in silico* drug design approach incorporating deep learning-based (Alphafold 3) structural prediction along with advanced computational methods was applied to identify potential therapeutic candidates against RdRp of IMNV. RdRp is a promising and selective antiviral target as host cells do not have RNA-dependent RNA polymerases that enable viral replication of RNA. The same approach towards RdRp has demonstrated therapeutic efficacy against a number of RNA viruses, such as SARS-CoV-2, hepatitis C virus, and dengue virus (Kumar et al., 2021; Sesmero and Thorpe, 2015; Ao et al., 2020; Ali et al., 2026). Thus, blocking IMNV RdRp can disrupt viral replication and propagation, and eventually, decrease viral infection in shrimp aquaculture systems.

Since there is no experimental crystal or NMR structure of IMNV RdRp has been solved so far in the RCSB Protein Data Bank, AlphaFold 3 was used to create the three-dimensional structure of the target protein. Recently, deep learning-based AlphaFold 3 has shown impressive accuracy in biomolecular structure prediction by modeling with deep learning and structural generation with diffusion models (Albawi et al., 2017; Senior et al., 2020). The five predicted models of this study exhibited an identical pTM value of 0.93, indicating high global structural confidence. Generally, a pTM score greater than 0.5 indicates that the predicted structure likely adopts a fold similar to the native conformation (Jiménez-Osés and Peccati, 2025; Abramson et al., 2024). The quality and structural reliability of the predicted models were evaluated by high ERRAT and VERIFY3D scores along with Ramachandran plot statistics (> 90% residues in favored regions). The structural confidence of the AlphaFold 3-predicted RdRp model was assessed using pLDDT and PAE analyses. The majority of the predicted structure exhibited very high confidence (pLDDT > 90), whereas a few localized loop regions showed lower confidence, including regions with pLDDT < 70. The PAE plot generally indicated low predicted positional errors across the major structural regions. Although the presence of lower-confidence regions represents a limitation of the predicted structure and may introduce uncertainty in local structural geometry, the overall high-confidence structural model provided a suitable basis for subsequent computational analyses. Therefore, the findings of the subsequent computational analyses should be interpreted with consideration of the potential uncertainty associated with the lower-confidence regions.

The present study provides a sequence and structure-based characterization of the putative IMNV RdRp encoded by ORF2. CDD analysis identified a conserved viral RdRp domain, while BLASTP showed strong similarity to a Totiviridae RdRp, supporting the previously reported polymerase characterization. The eight conserved IMNV/Totiviridae RdRp motifs were successfully mapped onto the AlphaFold 3 model_1 structure, with most motifs showing high local structural confidence. The predicted catalytic region contained the conserved D354-X4-D359 acidic pattern and G466-D467-D468 GDD motif, with high confidence in the GDD-containing region, supporting its annotation as a putative catalytic region. The region corresponding to canonical motif F was further annotated as a putative NTP-entry-associated region, while a motif 2 was annotated as a putative RNA-template entry associated regions based on conserved RdRp structural organization. The predicted ligand-interacting regions also showed moderate to high local structural confidence. The conserved acidic residues D354, D467, and D468 were also considered candidate metal-coordinating residues based on the established RdRp catalytic architecture. However, the catalytic region, ligand-binding regions, and entry associated regions remain computational predictions and require experimental structural or biochemical validation.

The molecular docking study revealed several potential seaweed-derived metabolites with favorable predicted binding affinities towards IMNV RdRp. In molecular docking, a more negative (lower) binding score generally indicates a more favorable, stable, and strong binding affinity between a ligand and a receptor (protein) (Alsedfy et al., 2024). Among the screened compounds, GA002 exhibited the highest docking score of −13.0 kcal/mol, whereas RC003 and BE012 also exhibited favorable docking scores of −10.9 and −10.8 kcal/mol, respectively. The presence of multiple hydrogen bonds between the ligands and the target protein suggests stronger binding affinity and enhanced stability of the protein-ligand complex, which may contribute to improved biological activity (Herschlag and Pinney, 2018; Williams and Ladbury, 2005). The molecular docking study revealed several potential seaweed-derived metabolites with favorable predicted binding affinities towards IMNV RdRp_M01. GA002 showed the most favorable docking score (−13.0 kcal/mol), while RC003 and BE012 also showed favorable scores of −10.9 and −10.8 kcal/mol, respectively. Their selection for subsequent MD simulation and ADMET analysis was based on the combination of favorable docking scores and comparatively diverse interaction profiles observed in the docked complexes. However, the MD simulations showed very low mean hydrogen-bond counts for GA002, RC003, and BE012, indicating that the hydrogen-bond interactions observed in the initial docking poses were not persistent throughout the 200 ns simulations. Therefore, the docking interactions represent initial pose-specific interactions rather than persistent hydrogen-bond-mediated binding. The protein RMSD analysis indicates that all complexes maintained overall structural stability during the simulation. The Apo (RdRp_M01) protein exhibited the lowest deviation (2.28 ± 0.15 Å), suggesting minimal structural fluctuation in the absence of ligand. Among the complexes, RdRp_M01_GA002 (2.34 ± 0.31) and RdRp_M01_RC003 (2.58 ± 0.18) showed comparable RMSD values, reflecting stable protein behavior upon ligand binding. In contrast, the RdRp_M01_BE012 complex exhibited slightly higher RMSD (2.80 ± 0.37 Å), indicating transient deviations from the average RMSD. Overall, the results suggest that ligand binding does not significantly disrupt the structural integrity of the protein. The ligand RMSD analysis revealed differences in conformational stability among the studied compounds. RC003 exhibited the lowest RMSD value with minimal deviation (1.05 ± 0.14 Å), indicating a stable binding conformation within the protein binding pocket. In contrast, GA002 showed the highest RMSD and variability (2.46 ± 0.45 Å), suggesting greater conformational flexibility during the simulation. However, BE012 displayed intermediate behavior (1.85 ± 0.28 Å), reflecting moderate stability compared to the other ligands.

Residue-level flexibility analysis using RMSF exhibited moderate fluctuations across all protein-ligand complexes, demonstrating that ligand binding caused minimal perturbation to the overall structural dynamics of RdRp. Higher RMSF values indicate greater flexibility, while lower values correspond to more stable regions (Song et al., 2024; Enayatkhani et al., 2020). The Apo protein showed the lowest fluctuation, while the RdRp_M01_BE012 complex exhibited slightly higher RMSF values, suggesting increased local flexibility. The RdRp_M01_GA002 and RdRp_M01_RC003 complexes showed closely comparable mean RMSF values, although slight differences in their standard deviations indicate variation in residue mobility. Overall, the observed variations reflect minor differences in residue flexibility rather than substantial structural changes.

The higher radius of gyration (Rg) values reflect a more expanded and less compact protein structure, whereas lower values indicate a more compact and stable conformation (Rahimi et al., 2023; Khan et al., 2021). In our studied protein-ligand complexes, the Rg analysis indicates that the protein maintained a stable structural arrangement throughout the simulation. In addition, the SASA analysis provides insight into the solvent exposure and conformational behavior of the protein-ligand complexes. The relatively stable SASA values were observed across all complexes, suggesting that no major conformational changes occurred during the simulation. Although the RdRp_M01_GA002 complex exhibited comparatively higher Rg and SASA values, suggesting increased solvent exposure and reduced compactness, the overall structural arrangement remained stable throughout the trajectory. On the other hand, RdRp_M01_RC003 demonstrated lower Rg and SASA profiles, indicating a more compact and less solvent-accessible conformation. The H-bond analysis indicates minimal intermolecular interactions between the protein and ligands during the simulation. The RdRp_M01_GA002 and RdRp_M01_BE012 complexes form only one hydrogen bond, suggesting transient interactions rather than stable bonding. In contrast, the RdRp_M01_RC003 complex did not form any hydrogen bonds throughout the 200 ns simulation. However, hydrogen bonding alone is not the sole determinant of complex stability, as other multiple non-covalent interactions, including van der Waals forces, may play a significant role, particularly in the RdRp_M01_RC003 complex.

Post-simulation MM-GBSA analysis further supported the dynamic simulation findings. Post-simulation MMGBSA analysis revealed that all complexes remained relatively stable over the 200 ns simulation trajectory, with only minor fluctuations observed during 88–137 ns and 156–184 ns. Outside these intervals, the complexes exhibited minimal variation, reflecting overall stable behavior. Among the studied complexes, RdRp_M01_BE012 showed the most favorable binding free energy (−34.850 kcal/mol), compared to RdRp_M01_GA002 (−24.726 kcal/mol) and RdRp_M01_RC003 (−26.722 kcal/mol), indicating stronger energetic stability and enhanced intermolecular interactions with RdRp_M01. Furthermore, RdRp_M01_BE012 exhibited the most favorable van der Waals interaction energy (−53.651 kcal/mol), reflecting stronger non-covalent interactions. Although the docking score of GA002 was higher than others, its comparatively less favorable MM-GBSA value and higher conformational fluctuations suggest that only the docking scores alone may not reliably predict long-term dynamic stability. These findings collectively demonstrate the importance of combining docking, MD simulation, and free-energy calculations for identifying reliable antiviral candidates. Overall, these findings suggest that RdRp_M01_BE012 forms the most energetically favorable protein-ligand complex among the studied complexes.

The pharmacokinetics results revealed notable differences among selected compounds regarding structural, biological, and environmental safety profiles. The lipophilicity index varied largely, particularly, the GA002 exhibited the highest Consensus Log *P*_o/w_ value, followed by RC003 and BE012, which may influence solubility and distribution behavior. The solubility results (Log S) of RC003 and BE012 showed poor water solubility, whereas GA002 showed insoluble properties. Although low solubility may limit dissolution in aqueous environments, it can also favor accumulation in lipid-rich compartments. However, lipid-based nanoparticle systems can enhance the solubility, stability, and bioavailability of poorly water-soluble compounds by encapsulating them within a lipid matrix, thereby improving their delivery in aqueous environments (Rehman et al., 2024; Mehnert and Mäder, 2001). Due to the low aqueous solubility of the studied compounds, their effective delivery in aquatic systems may be limited. Therefore, lipid-based nanoparticle delivery systems could be considered to enhance solubility, stability, and bioavailability, thereby improving their potential as candidate compounds targeting the IMNV RdRp for further investigation. Drug-likeness assessment showed several Lipinski violations, particularly for BE012, suggesting potential limitations in oral bioavailability. Nevertheless, computational drug-likeness rules should be interpreted cautiously for marine natural products, as many clinically valuable natural compounds exhibit physicochemical properties outside conventional drug-likeness boundaries. The lower bioavailability scores of RC003 and BE012 compared to GA002 suggest a reduced likelihood of efficient absorption.

Environmental toxicity assessment showed variation in bioaccumulation potential, with RC003 and BE012 exhibiting relatively higher log_10_ BCF values compared to GA002. Nevertheless, all values were well below the threshold of log_10_ BCF < 3, suggesting a low likelihood of bioaccumulation in aquatic organisms. However, the aquatic toxicity assessment predicted toxicity toward both the fish Fathead minnow and the small planktonic crustacean *Daphnia magna* for all three compounds. The presence of multiple aquatic toxicity alerts in RC003 indicated potential environmental concern, whereas no aquatic toxicity alerts were identified for GA002 and BE012. Despite all compounds being predicted as inactive in ecotoxicity classification, crustacean-specific toxicity predictions provided additional insights. Although shrimp belong to crustaceans, no shrimp-specific models are currently available for ADMET prediction, and the crustacean model does not specify the exact species used. Therefore, these results should be interpreted as indicative rather than definitive for shrimp ADME and toxicity, and further experimental validations are needed. Overall, the three candidates showed distinct computational profiles. GA002 exhibited the most favorable docking score but comparatively weaker ligand stability during MD simulation, the least favorable MM-GBSA binding energy, and an unfavorable physicochemical profile characterized by very high lipophilicity and poor aqueous solubility. RC003 demonstrated the most favorable MD stability, particularly in terms of ligand RMSD, radius of gyration, and SASA, together with the second-most favorable MM-GBSA binding energy. BE012, despite showing relatively greater structural fluctuations during MD simulation, exhibited the most favorable MM-GBSA binding energy and a comparatively more favorable physicochemical profile. Therefore, based on the integrated assessment of docking, MD simulation, MM-GBSA, and ADMET properties, BE012 was prioritized as the most promising candidate, followed by RC003 and GA002.

Existing research has shown that the metabolites from the sea have a wide range of antiviral effects against several types of RNA viruses by blocking virus enzymes and replication pathways (Lomartire and Gonçalves, 2022; Riccio et al., 2020). The antiviral, antioxidant, and immunomodulatory properties of the seaweed-derived sulfated polysaccharides, terpenoids, and phenolic compounds have gained special interest (Lomartire and Gonçalves, 2022; Hans et al., 2021). Based on these findings, the identified seaweed metabolites could be considered computationally prioritized candidate compounds for further investigation against IMNV. Moreover, this computation study systematically explores the seaweed metabolites against IMNV RdRp, which offers a valuable contribution to the field of antiviral drug discovery for shrimp aquaculture diseases.

Although IMNV infection triggers RNA interference (RNAi)-related gene expression in shrimp, this response does not significantly reduce viral load, indicating that innate defense mechanisms alone are inadequate and emphasizing the necessity for novel antiviral drug development for sustainable shrimp aquaculture (Feijó et al., 2025). Overall, this study employed an *in silico* drug design approach integrating deep learning-based tools during different stages of analysis. The identified hit compounds showed promising binding affinity, dynamic stability, favorable energetic profiles, and acceptable pharmacokinetic features, supporting their prioritization as candidate compounds for further experimental evaluation against IMNV in shrimp aquaculture. However, further experimental validation through *in vitro* and *in vivo* studies is required to confirm the predicted efficacy and safety of the identified compounds.

## 5 Limitations

This study employed multiple computational approaches to identify potential IMNV RdRp inhibitors; however, several limitations should be acknowledged. The predicted protein structure and ligand-binding regions remain computational models and lack validation against experimentally determined structures. Molecular docking, MD simulations, MM-GBSA, and local pLDDT/PAE analyses provide valuable estimates of binding and structural confidence but cannot fully reproduce complex biological interactions. Furthermore, pharmacokinetic predictions were based on in silico models developed for humans, fathead minnow, and Daphnia magna because shrimp-specific models were unavailable, limiting their direct applicability to shrimp. Although GA002, RC003, and BE012 were prioritized as promising IMNV RdRp inhibitors, their binding affinity, antiviral efficacy, bioavailability, toxicity, and safety remain experimentally unverified. Therefore, further *in vitro* and *in vivo* studies using appropriate shrimp and viral infection models are required to validate these findings.

## 6 Conclusion

In shrimp aquaculture infectious Myonecrosis Virus (IMNV) is a highly virulent pathogen causing severe infection and mortality. It is considered a major global threat to the multi-billion-dollar shrimp aquaculture industry due to its rapid spread among the shrimp. This current study predicted deep learning-based AlphaFold 3 protein structure to identify seaweed-derived candidate compounds for further investigation against IMNV infections. The docking-based virtual screening identified GA002, RC003, and BE012, derived from green algae, Fijian red algae, and marine brown algae, respectively, as the most promising lead compounds. These three selected compounds demonstrated structural stability and favorable binding towards the target protein of IMNV through MD simulation and post-MD MM-GBSA analysis. To the best of current knowledge, no prior studies have reported these compounds in the context of antiviral activity against IMNV, and no previous research has utilized the database to target IMNV, underscoring the novelty of this study. Thus, the findings of this study highlight GA002, RC003, and BE012 as computationally prioritized candidates for further investigation against IMNV infection. Therefore, these results encourage broader exploration of seaweed metabolites as a valuable source of for further investigation against IMNV. However, further experimental validation through *in vitro* and *in vivo* studies is essential to evaluate their real-world efficacy.

## Ethics statement

This study did not involve human or animal subjects, and therefore, an ethics statement is not required.

## Informed consent statement

Not applicable.

## Funding

This research has not received any funding.

## CRediT authorship contribution statement

**Mostafizur Rahman:** Writing – original draft, Visualization, Validation, Methodology, Investigation, Formal analysis, Data curation, Conceptualization. **Md Ahad Ali:** Writing – original draft, Validation, Supervision, Project administration, Formal analysis, Conceptualization. **Tahosin Tasneem Samara:** Visualization, Investigation, Formal analysis, Data curation. **Monish Saha:** Writing – original draft, Visualization, Investigation, Data curation. **Pritom Kundu:** Software, Resources, Investigation. **Hriddhi Sarker:** Writing – review & editing, Validation, Data curation. **Ankita Dutta:** Validation, Software, Resources, Data curation. **Neeraj Kumar:** Writing – original draft, Software, Resources, Methodology.

## Declaration of competing interest

We can confirm that the work entitled “Deep learning-based structural prediction of shrimp IMNV RdRp and identification of seaweed-derived metabolites for antiviral intervention in aquaculture” is an original work and neither this nor any similar manuscript, in whole or in part, is under consideration, in a press, published, or reported elsewhere. All co-authors have seen and agreed with the contents of the manuscript. We would like to declare that we have no any conflict of interest for this work. Anyone can use any information from our manuscript.

## Data Availability

Data will be made available on request.

## Appendix Supplementary materials

**Supporting Information 1: Table S1**. Totiviridae BLASTP hits identified using the IMNV ORF2-encoded putative RdRp protein sequence as the query; **Table S2**. Molecular docking scores of the seaweed metabolites (1032 successful hits); **Table S3**. Docking-interacting residues of GA002, BE012, and RC003 and their relationship to the mapped conserved RdRp motif regions and predicted functional regions; **Table S4**. Correspondence between IMNV/Totiviridae motif numbering and canonical viral RdRp motifs in AlphaFold 3 model_1; **Table S5**. Local structural confidence of conserved RdRp motifs and predicted ligand-binding residues in the AlphaFold 3 model_1 of IMNV RdRp; **Table S6**. Local structural confidence of predicted ligand-binding regions in the AlphaFold 3 model_1 of IMNV RdRp; **Figure S1**. The 2D structures of the six selected ligands: (**A**) GA002, (**B**) RC003, (**C**) BE012, (**D**) RL354, (**E**) RL015, and (**F**) BS067. The structures retain the stereochemical information provided by the Seaweed Metabolite Database; **Figure S2**. The 3D structures of the six selected ligands: (**A**) GA002 (Red), (**B**) RC003 (Purple), (**C**) BE012 (Black), (**D**) RL354 (Blue), (**E**) RL015 (Yellow), and (**F**) BS067 (Pink), corresponding to the structures used for molecular docking; **Figure S3**. Complex view (Left), 3D interaction view (Middle), and 2D interaction map (Right) of the protein RdRp_M01 interactions with ligands: (**A**) RL354 (Blue), (**B**) RL015 (Yellow), and (**C**) BS067 (Pink); **Figure S4**. Putative catalytic region of the IMNV RdRp. (**A**) Overall AlphaFold 3 Model 1 structure and (**B**) Enlarged view showing the conserved acidic residues Asp354 and Asp359 and the G466-D467-D468 GDD motif; **Figure S5**. Putative entry-associated regions of the IMNV RdRp. (A) Putative NTP-entry-associated region corresponding to Motif F (residues 291–297) and (B) putative RNA-template entry-associated region associated with IMNV Motif 2 (residues 231–237); **Supporting Information 2: Supplementary 2A**. Pairwise BLASTP alignment between the IMNV ORF2-encoded putative RdRp protein sequence and the putative RNA-dependent RNA polymerase of Totiviridae sp. (UHS72541.1); **Supplementary 2B** BLASTP analysis of the IMNV ORF2-encoded putative RdRp protein against the NCBI ClusteredNR database, showing significant Totiviridae hits and pairwise sequence alignments with representative hits.
