# Supplementary material for: Deep learning-based structural prediction (AlphaFold 3) of shrimp IMNV RdRp and identification of seaweed-derived metabolites for antiviral intervention in aquaculture
Source: Virus Res. 2026 Sep 8;373:199796. doi: 10.1016/j.virusres.2026.199796 (PMC13595083; doi:10.1016/j.virusres.2026.199796)
Supplement: Supplementary file 1 [file mmc1.docx]

**Suppporting Information 1**

**Table S1**. Totiviridae BLASTP hits identified using the IMNV ORF2-encoded putative RdRp protein sequence as the query.

| Query_4838030 | UHS72541.1 | 59.879 | 825 | 330 | 1 | 14 | 838 | 10 | 833 | 0 | 1061 | 76.12 |
| --- | --- | --- | --- | --- | --- | --- | --- | --- | --- | --- | --- | --- |
| Query_4838030 | UHS72568.1 | 69.811 | 530 | 160 | 0 | 29 | 558 | 5 | 534 | 0 | 782 | 83.02 |
| Query_4838030 | UHS72444.1 | 44.832 | 803 | 438 | 4 | 38 | 838 | 112 | 911 | 0 | 702 | 64.76 |
| Query_4838030 | UHS72493.1 | 44.048 | 756 | 408 | 9 | 90 | 838 | 13 | 760 | 0 | 641 | 63.89 |
| Query_4838030 | UHS72478.1 | 42.191 | 794 | 438 | 11 | 51 | 838 | 29 | 807 | 0 | 628 | 61.84 |
| Query_4838030 | UHS72503.1 | 43.893 | 745 | 407 | 8 | 78 | 817 | 1 | 739 | 0 | 626 | 64.3 |
| Query_4838030 | UHS72552.1 | 42.308 | 780 | 428 | 11 | 66 | 838 | 87 | 851 | 0 | 622 | 62.05 |
| Query_4838030 | UHS72448.1 | 41.804 | 787 | 441 | 12 | 52 | 831 | 17 | 793 | 0 | 603 | 60.61 |
| Query_4838030 | UHS72466.1 | 42.995 | 728 | 395 | 8 | 54 | 767 | 59 | 780 | 0 | 598 | 61.68 |
| Query_4838030 | UHS72514.1 | 46.037 | 656 | 327 | 6 | 56 | 708 | 21 | 652 | 0 | 597 | 65.24 |
| Query_4838030 | UHS72496.1 | 44.379 | 676 | 361 | 6 | 64 | 731 | 132 | 800 | 0 | 580 | 63.17 |
| Query_4838030 | UHS72487.1 | 37.749 | 853 | 504 | 11 | 3 | 838 | 27 | 869 | 0 | 566 | 57.44 |
| Query_4838030 | UHS72509.1 | 68.325 | 382 | 121 | 0 | 9 | 390 | 19 | 400 | 0 | 548 | 82.2 |
| Query_4838030 | UHS71733.1 | 71.105 | 353 | 102 | 0 | 96 | 448 | 4 | 356 | 0 | 538 | 84.42 |
| Query_4838030 | UHS72421.1 | 40.591 | 744 | 416 | 8 | 111 | 838 | 1 | 734 | 0 | 537 | 61.29 |
| Query_4838030 | UHS72429.1 | 40.295 | 747 | 421 | 11 | 106 | 838 | 3 | 738 | ######## | 516 | 59.97 |
| Query_4838030 | UHS72490.1 | 37.736 | 795 | 462 | 15 | 52 | 838 | 18 | 787 | ######## | 510 | 57.48 |
| Query_4838030 | UHS72526.1 | 41.321 | 651 | 365 | 8 | 190 | 836 | 5 | 642 | ######## | 498 | 60.83 |
| Query_4838030 | UHS72476.1 | 37.212 | 782 | 459 | 15 | 66 | 838 | 14 | 772 | ######## | 492 | 57.54 |
| Query_4838030 | UHS72534.1 | 45.455 | 506 | 258 | 5 | 59 | 555 | 1 | 497 | ######## | 457 | 64.62 |
| Query_4838030 | UHS72482.1 | 42.91 | 543 | 301 | 6 | 66 | 604 | 46 | 583 | ######## | 438 | 59.85 |
| Query_4838030 | UHS72491.1 | 41.805 | 543 | 297 | 7 | 16 | 558 | 5 | 528 | ######## | 416 | 60.41 |
| Query_4838030 | UHS72480.1 | 50 | 400 | 192 | 4 | 219 | 614 | 7 | 402 | ######## | 407 | 69.25 |
| Query_4838030 | UHS72532.1 | 40.232 | 517 | 300 | 4 | 65 | 580 | 41 | 549 | ######## | 400 | 59.19 |
| Query_4838030 | UHS72560.1 | 52.507 | 379 | 165 | 4 | 249 | 621 | 1 | 370 | ######## | 391 | 68.07 |
| Query_4838030 | UHS72546.1 | 45.301 | 415 | 222 | 4 | 241 | 653 | 1 | 412 | ######## | 368 | 65.06 |
| Query_4838030 | UHS72488.1 | 44.289 | 429 | 231 | 3 | 242 | 666 | 1 | 425 | ######## | 367 | 62.24 |
| Query_4838030 | UHS72468.1 | 49.102 | 334 | 169 | 1 | 199 | 532 | 1 | 333 | ######## | 343 | 68.86 |
| Query_4838030 | UHS72564.1 | 47.411 | 367 | 190 | 1 | 153 | 519 | 1 | 364 | ######## | 343 | 64.58 |
| Query_4838030 | UHS72558.1 | 41.09 | 477 | 271 | 8 | 54 | 525 | 33 | 504 | ######## | 347 | 59.12 |
| Query_4838030 | UHS72525.1 | 50.151 | 331 | 156 | 4 | 219 | 545 | 14 | 339 | ######## | 334 | 69.79 |
| Query_4838030 | UHS72522.1 | 44.386 | 383 | 204 | 4 | 66 | 446 | 27 | 402 | ######## | 334 | 64.75 |
| Query_4838030 | UHS72417.1 | 39.752 | 483 | 271 | 9 | 51 | 523 | 22 | 494 | ######## | 337 | 58.18 |
| Query_4838030 | UHS72432.1 | 40.984 | 427 | 237 | 4 | 418 | 838 | 9 | 426 | ######## | 326 | 59.48 |
| Query_4838030 | UHS72538.1 | 37.945 | 477 | 273 | 10 | 52 | 521 | 18 | 478 | 4.06E-95 | 302 | 57.86 |
| Query_4838030 | UHS72415.1 | 38.263 | 426 | 252 | 7 | 66 | 486 | 46 | 465 | 7.26E-89 | 285 | 57.51 |
| Query_4838030 | UHS72416.1 | 46 | 300 | 160 | 2 | 64 | 362 | 46 | 344 | 8.63E-88 | 278 | 65 |
| Query_4838030 | UHS72413.1 | 39.108 | 381 | 216 | 5 | 396 | 769 | 7 | 378 | 5.58E-85 | 272 | 57.48 |
| Query_4838030 | UHS72556.1 | 41.896 | 327 | 182 | 3 | 42 | 368 | 12 | 330 | 6.79E-82 | 262 | 61.47 |
| Query_4838030 | UHS72537.1 | 42.075 | 347 | 192 | 5 | 146 | 488 | 5 | 346 | 1.39E-80 | 259 | 59.65 |
| Query_4838030 | UHS72524.1 | 40.653 | 337 | 191 | 4 | 211 | 547 | 4 | 331 | 5.33E-79 | 254 | 62.61 |
| Query_4838030 | UHS72528.1 | 37.304 | 319 | 194 | 4 | 54 | 367 | 33 | 350 | 3.07E-65 | 218 | 58.31 |
| Query_4838030 | WAK77453.1 | 27.431 | 802 | 496 | 29 | 56 | 796 | 136 | 912 | 7.39E-65 | 230 | 45.39 |
| Query_4838030 | UHS72569.1 | 36.782 | 348 | 203 | 8 | 66 | 410 | 29 | 362 | 2.91E-64 | 216 | 56.61 |
| Query_4838030 | WAK77419.1 | 28.06 | 670 | 422 | 22 | 56 | 682 | 136 | 788 | 3.88E-63 | 225 | 48.21 |
| Query_4838030 | UHS72475.1 | 42.797 | 236 | 135 | 0 | 54 | 289 | 59 | 294 | 1.65E-61 | 206 | 63.14 |
| Query_4838030 | WAK77558.1 | 28.786 | 667 | 421 | 24 | 56 | 682 | 115 | 767 | 4.50E-61 | 219 | 47.38 |
| Query_4838030 | WAK77448.1 | 27.622 | 677 | 431 | 22 | 49 | 682 | 3 | 663 | 1.11E-60 | 216 | 46.97 |
| Query_4838030 | WAK77483.1 | 27.488 | 633 | 421 | 17 | 56 | 654 | 185 | 813 | 1.57E-60 | 218 | 47.24 |
| Query_4838030 | UHS72455.1 | 38.435 | 294 | 169 | 4 | 51 | 336 | 22 | 311 | 3.62E-60 | 202 | 56.8 |
| Query_4838030 | WAK77607.1 | 27.259 | 675 | 431 | 21 | 56 | 685 | 106 | 765 | 6.71E-59 | 212 | 47.11 |
| Query_4838030 | WAK77488.1 | 28.92 | 574 | 373 | 18 | 56 | 601 | 212 | 778 | 8.33E-59 | 211 | 48.61 |
| Query_4838030 | WAK77258.1 | 26.997 | 726 | 460 | 27 | 56 | 734 | 115 | 817 | 8.84E-59 | 212 | 46.01 |
| Query_4838030 | UHS72457.1 | 42.727 | 220 | 126 | 0 | 64 | 283 | 132 | 351 | 1.36E-58 | 199 | 64.55 |
| Query_4838030 | WAK77681.1 | 26.754 | 684 | 423 | 23 | 56 | 685 | 106 | 765 | 2.29E-58 | 211 | 46.78 |
| Query_4838030 | WAK77316.1 | 26.244 | 663 | 452 | 15 | 56 | 686 | 7 | 664 | 7.62E-58 | 208 | 45.55 |
| Query_4838030 | WAK77387.1 | 28.595 | 619 | 393 | 21 | 99 | 682 | 27 | 631 | 3.05E-57 | 206 | 47.33 |
| Query_4838030 | WAK77262.1 | 26.172 | 661 | 455 | 16 | 56 | 686 | 21 | 678 | 7.56E-57 | 205 | 46.6 |
| Query_4838030 | WAK77610.1 | 27.174 | 644 | 417 | 21 | 52 | 654 | 41 | 673 | 2.52E-56 | 204 | 46.12 |
| Query_4838030 | UUT42649.1 | 27.273 | 638 | 411 | 22 | 56 | 653 | 230 | 854 | 1.29E-55 | 203 | 47.96 |
| Query_4838030 | WAK77747.1 | 27.215 | 632 | 419 | 17 | 56 | 653 | 230 | 854 | 2.89E-55 | 202 | 46.36 |
| Query_4838030 | WAK77284.1 | 27.488 | 633 | 401 | 21 | 66 | 654 | 472 | 1090 | 4.66E-55 | 202 | 45.97 |
| Query_4838030 | WAK77254.1 | 26.395 | 663 | 443 | 19 | 56 | 682 | 31 | 684 | 8.54E-55 | 199 | 45.85 |
| Query_4838030 | WAK77427.1 | 27.402 | 635 | 414 | 20 | 56 | 653 | 103 | 727 | 2.23E-54 | 199 | 46.14 |
| Query_4838030 | UHS72484.1 | 38.403 | 263 | 161 | 1 | 16 | 278 | 164 | 425 | 2.60E-54 | 190 | 59.7 |
| Query_4838030 | WAK77278.1 | 26.291 | 639 | 416 | 17 | 56 | 653 | 129 | 753 | 3.67E-54 | 198 | 46.01 |
| Query_4838030 | WAK77616.1 | 29.432 | 581 | 358 | 19 | 56 | 601 | 7 | 570 | 5.23E-54 | 197 | 47.85 |
| Query_4838030 | WAK77676.1 | 29.389 | 524 | 307 | 18 | 56 | 538 | 106 | 607 | 5.67E-54 | 194 | 48.47 |
| Query_4838030 | XFO99082.1 | 26.972 | 634 | 409 | 18 | 63 | 654 | 126 | 747 | 1.26E-53 | 197 | 45.74 |
| Query_4838030 | WAK77722.1 | 27.832 | 618 | 399 | 20 | 73 | 653 | 3 | 610 | 5.34E-52 | 191 | 47.09 |
| Query_4838030 | WAK77292.1 | 25.669 | 635 | 416 | 17 | 63 | 654 | 123 | 744 | 1.12E-51 | 191 | 45.51 |
| Query_4838030 | XCG98880.1 | 24.59 | 671 | 433 | 24 | 62 | 686 | 13 | 656 | 2.50E-51 | 187 | 46.8 |
| Query_4838030 | WAK77583.1 | 24.543 | 656 | 450 | 19 | 56 | 674 | 45 | 692 | 6.51E-51 | 188 | 46.49 |
| Query_4838030 | XZG29191.1 | 24.59 | 671 | 433 | 24 | 62 | 686 | 125 | 768 | 2.02E-50 | 187 | 46.8 |
| Query_4838030 | WAK77535.1 | 24.265 | 680 | 457 | 20 | 62 | 700 | 13 | 675 | 3.33E-50 | 186 | 45 |
| Query_4838030 | XCG98881.1 | 23.802 | 668 | 443 | 22 | 62 | 683 | 13 | 660 | 9.08E-50 | 184 | 46.56 |
| Query_4838030 | WAK77658.1 | 25.04 | 627 | 417 | 20 | 93 | 682 | 21 | 631 | 3.73E-49 | 182 | 46.73 |
| Query_4838030 | WAK77245.1 | 24.251 | 668 | 440 | 22 | 62 | 683 | 2014 | 2661 | 2.64E-48 | 182 | 46.56 |
| Query_4838030 | WAK77504.1 | 26.915 | 483 | 319 | 14 | 99 | 558 | 27 | 498 | 2.99E-47 | 172 | 47.2 |
| Query_4838030 | WAK77481.1 | 28.989 | 445 | 284 | 14 | 56 | 474 | 185 | 623 | 1.23E-46 | 172 | 49.21 |
| Query_4838030 | WAK77695.1 | 28.761 | 452 | 295 | 12 | 169 | 601 | 1 | 444 | 1.27E-46 | 173 | 50 |
| Query_4838030 | WWV90225.1 | 31.832 | 333 | 201 | 8 | 466 | 781 | 1 | 324 | 4.57E-43 | 156 | 49.85 |
| Query_4838030 | WAK77298.1 | 28.802 | 434 | 284 | 13 | 56 | 468 | 188 | 617 | 1.25E-40 | 154 | 48.39 |
| Query_4838030 | WAK77652.1 | 28.502 | 414 | 268 | 10 | 56 | 446 | 106 | 514 | 1.39E-40 | 152 | 47.34 |
| Query_4838030 | WAK77755.1 | 28.788 | 330 | 214 | 11 | 56 | 367 | 7 | 333 | 7.50E-32 | 123 | 50 |
| Query_4838030 | WWV90304.1 | 34.911 | 169 | 101 | 5 | 642 | 808 | 6 | 167 | 4.72E-27 | 103 | 59.17 |
| Query_4838030 | WAK77683.1 | 35.333 | 150 | 92 | 2 | 222 | 367 | 61 | 209 | 1.29E-25 | 102 | 58.67 |
| Query_4838030 | QYW06889.1 | 28.358 | 268 | 173 | 7 | 301 | 558 | 1 | 259 | 3.28E-22 | 92.8 | 50.37 |
| Query_4838030 | WAK77592.1 | 33.913 | 115 | 73 | 3 | 426 | 538 | 2 | 115 | 1.43E-11 | 59.3 | 52.17 |
| Query_4838030 | BDC79619.1 | 18.481 | 395 | 259 | 14 | 231 | 605 | 224 | 575 | 2.37E-06 | 46.6 | 39.24 |
| Query_4838030 | BDG62461.1 | 18.694 | 337 | 216 | 13 | 289 | 605 | 1 | 299 | 3.66E-04 | 38.9 | 39.17 |
| Query_4838030 | QKN88741.1 | 26.178 | 191 | 125 | 6 | 420 | 596 | 824 | 1012 | 0.006 | 35.4 | 40.84 |

**Table S2**. Molecular docking scores of the seaweed metabolites (1032 successful hits).

| **Compounds** | **Binding Affinity (kcal/mol)** |
| --- | --- |
| GA002 | -13 |
| RL354 | -11.5 |
| RL015 | -11 |
| BS067 | -10.9 |
| RC003 | -10.9 |
| BE012 | -10.8 |
| BE005 | -10.7 |
| RC034 | -10.6 |
| RL322 | -10.6 |
| RC036 | -10.5 |
| RC037 | -10.5 |
| BD013 | -10.4 |
| BS034 | -10.4 |
| RC005 | -10.4 |
| BE011 | -10.3 |
| BS068 | -10.3 |
| RC006 | -10.3 |
| RC035 | -10.3 |
| RC038 | -10.3 |
| BS032 | -10.2 |
| BS033 | -10.2 |
| RC016 | -10.2 |
| RL283 | -10.2 |
| BE016 | -10.1 |
| BS055 | -10.1 |
| RC007 | -10.1 |
| RC009 | -10.1 |
| RL326 | -10.1 |
| BS024 | -10 |
| BS060 | -10 |
| RC012 | -10 |
| RC013 | -10 |
| RL006 | -10 |
| RL067 | -10 |
| BE015 | -9.9 |
| BS022 | -9.9 |
| BS056 | -9.9 |
| BS057 | -9.9 |
| BS058 | -9.9 |
| RC015 | -9.9 |
| RG007 | -9.9 |
| BS026 | -9.8 |
| BS028 | -9.8 |
| BS037 | -9.8 |
| RL082 | -9.8 |
| RL325 | -9.8 |
| BS035 | -9.7 |
| BS039 | -9.7 |
| GA003 | -9.7 |
| RC004 | -9.7 |
| RC014 | -9.7 |
| RC040 | -9.7 |
| RR020 | -9.7 |
| RL324 | -9.7 |
| BS021 | -9.6 |
| BS031 | -9.6 |
| RC011 | -9.6 |
| RC023 | -9.6 |
| RC024 | -9.6 |
| RC025 | -9.6 |
| RC039 | -9.6 |
| BS029 | -9.5 |
| RC008 | -9.5 |
| BS036 | -9.4 |
| RC010 | -9.4 |
| BS025 | -9.3 |
| RG004 | -9.3 |
| RL376 | -9.3 |
| RL474 | -9.3 |
| BD069 | -9.2 |
| BS023 | -9.2 |
| BS030 | -9.2 |
| RC028 | -9.2 |
| RG019 | -9.2 |
| RR015 | -9.2 |
| RL374 | -9.2 |
| RL485 | -9.2 |
| RL487 | -9.2 |
| BD078 | -9.1 |
| RL426 | -9.1 |
| RL442 | -9.1 |
| RL512 | -9.1 |
| BE008 | -9 |
| BS059 | -9 |
| BT006 | -9 |
| GA012 | -9 |
| RG001 | -9 |
| RG012 | -9 |
| RL132 | -9 |
| RL136 | -9 |
| RL486 | -9 |
| RL514 | -9 |
| BD068 | -8.9 |
| BE004 | -8.9 |
| BE013 | -8.9 |
| RC021 | -8.9 |
| RG003 | -8.9 |
| RG011 | -8.9 |
| RG014 | -8.9 |
| RL066 | -8.9 |
| RL372 | -8.9 |
| RL373 | -8.9 |
| RL381 | -8.9 |
| RL473 | -8.9 |
| RL551 | -8.9 |
| BD066 | -8.8 |
| BD067 | -8.8 |
| BS069 | -8.8 |
| BS070 | -8.8 |
| RG005 | -8.8 |
| RG008 | -8.8 |
| RG009 | -8.8 |
| RG010 | -8.8 |
| RJ006 | -8.8 |
| RJ008 | -8.8 |
| RL083 | -8.8 |
| RL208 | -8.8 |
| RR021 | -8.8 |
| RL377 | -8.8 |
| RL323 | -8.8 |
| RL513 | -8.8 |
| RL550 | -8.8 |
| BD071 | -8.7 |
| BE010 | -8.7 |
| BS038 | -8.7 |
| BS072 | -8.7 |
| BZ004 | -8.7 |
| RL379 | -8.7 |
| RL515 | -8.7 |
| BC007 | -8.6 |
| BT008 | -8.6 |
| RG006 | -8.6 |
| RL466 | -8.6 |
| RL467 | -8.6 |
| RL484 | -8.6 |
| RL516 | -8.6 |
| BC005 | -8.5 |
| BC008 | -8.5 |
| BC011 | -8.5 |
| BC013 | -8.5 |
| BD007 | -8.5 |
| BD070 | -8.5 |
| BS063 | -8.5 |
| BS071 | -8.5 |
| BT005 | -8.5 |
| BT007 | -8.5 |
| GA011 | -8.5 |
| RG002 | -8.5 |
| RG013 | -8.5 |
| RL242 | -8.5 |
| RL243 | -8.5 |
| RR019 | -8.5 |
| RL469 | -8.5 |
| RL470 | -8.5 |
| RL471 | -8.5 |
| BC004 | -8.4 |
| BC014 | -8.4 |
| BS009 | -8.4 |
| BT009 | -8.4 |
| GU003 | -8.4 |
| RC022 | -8.4 |
| RC027 | -8.4 |
| RL048 | -8.4 |
| RL056 | -8.4 |
| RL284 | -8.4 |
| RL375 | -8.4 |
| RL378 | -8.4 |
| BD039 | -8.3 |
| BE002 | -8.3 |
| BE014 | -8.3 |
| BS020 | -8.3 |
| BT010 | -8.3 |
| BT011 | -8.3 |
| RJ004 | -8.3 |
| RJ005 | -8.3 |
| RL050 | -8.3 |
| RL086 | -8.3 |
| RL150 | -8.3 |
| RR033 | -8.3 |
| RS005 | -8.3 |
| RS006 | -8.3 |
| RL368 | -8.3 |
| RL304 | -8.3 |
| RL315 | -8.3 |
| BD025 | -8.2 |
| BD028 | -8.2 |
| BD041 | -8.2 |
| BE003 | -8.2 |
| BS007 | -8.2 |
| BT004 | -8.2 |
| BT012 | -8.2 |
| GU002 | -8.2 |
| GU005 | -8.2 |
| RJ007 | -8.2 |
| RL036 | -8.2 |
| RL110 | -8.2 |
| RL291 | -8.2 |
| RL367 | -8.2 |
| RL394 | -8.2 |
| RL301 | -8.2 |
| RL427 | -8.2 |
| RL460 | -8.2 |
| BD043 | -8.1 |
| BS003 | -8.1 |
| BS010 | -8.1 |
| GU004 | -8.1 |
| RJ003 | -8.1 |
| RL184 | -8.1 |
| RL343 | -8.1 |
| BC009 | -8 |
| BD014 | -8 |
| BD049 | -8 |
| BD064 | -8 |
| BD072 | -8 |
| BD076 | -8 |
| BS005 | -8 |
| BS011 | -8 |
| RJ002 | -8 |
| RL002 | -8 |
| RL037 | -8 |
| RL047 | -8 |
| RL100 | -8 |
| RL111 | -8 |
| RL229 | -8 |
| RL261 | -8 |
| RL262 | -8 |
| RR013 | -8 |
| RR022 | -8 |
| RS008 | -8 |
| RL362 | -8 |
| RL363 | -8 |
| RL306 | -8 |
| RL436 | -8 |
| BC003 | -7.9 |
| BD021 | -7.9 |
| BD031 | -7.9 |
| BD040 | -7.9 |
| BD042 | -7.9 |
| BS002 | -7.9 |
| BS004 | -7.9 |
| BS008 | -7.9 |
| BS041 | -7.9 |
| BS054 | -7.9 |
| GA008 | -7.9 |
| GC001 | -7.9 |
| RC002 | -7.9 |
| RJ001 | -7.9 |
| RL024 | -7.9 |
| RL102 | -7.9 |
| RL247 | -7.9 |
| RL275 | -7.9 |
| RL286 | -7.9 |
| RL287 | -7.9 |
| RR018 | -7.9 |
| RS007 | -7.9 |
| RL361 | -7.9 |
| RL370 | -7.9 |
| RL371 | -7.9 |
| RL380 | -7.9 |
| RL443 | -7.9 |
| RL476 | -7.9 |
| RL505 | -7.9 |
| RL524 | -7.9 |
| BD027 | -7.8 |
| BD044 | -7.8 |
| BD046 | -7.8 |
| BD082 | -7.8 |
| BS013 | -7.8 |
| BS019 | -7.8 |
| GA006 | -7.8 |
| RL029 | -7.8 |
| RL040 | -7.8 |
| RL041 | -7.8 |
| RL045 | -7.8 |
| RL103 | -7.8 |
| RL139 | -7.8 |
| RL140 | -7.8 |
| RL141 | -7.8 |
| RL181 | -7.8 |
| RL182 | -7.8 |
| RL206 | -7.8 |
| RL207 | -7.8 |
| RL246 | -7.8 |
| RL285 | -7.8 |
| RO006 | -7.8 |
| RR005 | -7.8 |
| RL365 | -7.8 |
| RL369 | -7.8 |
| RL321 | -7.8 |
| RL406 | -7.8 |
| RL468 | -7.8 |
| RL535 | -7.8 |
| BC015 | -7.7 |
| BD009 | -7.7 |
| BD034 | -7.7 |
| BD047 | -7.7 |
| BD073 | -7.7 |
| BE007 | -7.7 |
| BS012 | -7.7 |
| BS061 | -7.7 |
| BS062 | -7.7 |
| GA007 | -7.7 |
| GC003 | -7.7 |
| RC030 | -7.7 |
| RL001 | -7.7 |
| RL003 | -7.7 |
| RL004 | -7.7 |
| RL033 | -7.7 |
| RL046 | -7.7 |
| RL073 | -7.7 |
| RL093 | -7.7 |
| RL179 | -7.7 |
| RL186 | -7.7 |
| RL228 | -7.7 |
| RL234 | -7.7 |
| RL236 | -7.7 |
| RL239 | -7.7 |
| RL300 | -7.7 |
| RL364 | -7.7 |
| RL382 | -7.7 |
| RL383 | -7.7 |
| RL302 | -7.7 |
| RL407 | -7.7 |
| RL422 | -7.7 |
| RL425 | -7.7 |
| RL432 | -7.7 |
| RL440 | -7.7 |
| RL447 | -7.7 |
| BD022 | -7.6 |
| BD032 | -7.6 |
| BD045 | -7.6 |
| BD063 | -7.6 |
| GA009 | -7.6 |
| RC026 | -7.6 |
| RC029 | -7.6 |
| RL005 | -7.6 |
| RL008 | -7.6 |
| RL019 | -7.6 |
| RL020 | -7.6 |
| RL042 | -7.6 |
| RL044 | -7.6 |
| RL095 | -7.6 |
| RL098 | -7.6 |
| RL151 | -7.6 |
| RL152 | -7.6 |
| RL185 | -7.6 |
| RL203 | -7.6 |
| RL204 | -7.6 |
| RL218 | -7.6 |
| RL230 | -7.6 |
| RL233 | -7.6 |
| RL248 | -7.6 |
| RL263 | -7.6 |
| RL277 | -7.6 |
| RL278 | -7.6 |
| RL290 | -7.6 |
| RP065 | -7.6 |
| RR014 | -7.6 |
| RR027 | -7.6 |
| RS002 | -7.6 |
| RS009 | -7.6 |
| RL352 | -7.6 |
| RL353 | -7.6 |
| RL433 | -7.6 |
| RL435 | -7.6 |
| RL472 | -7.6 |
| RL489 | -7.6 |
| RL537 | -7.6 |
| BC010 | -7.5 |
| BD008 | -7.5 |
| BD011 | -7.5 |
| BD016 | -7.5 |
| BD019 | -7.5 |
| BD023 | -7.5 |
| BD024 | -7.5 |
| BD026 | -7.5 |
| BD055 | -7.5 |
| BD075 | -7.5 |
| BD080 | -7.5 |
| BD083 | -7.5 |
| GU001 | -7.5 |
| GU010 | -7.5 |
| RL009 | -7.5 |
| RL010 | -7.5 |
| RL074 | -7.5 |
| RL097 | -7.5 |
| RL114 | -7.5 |
| RL115 | -7.5 |
| RL227 | -7.5 |
| RL235 | -7.5 |
| RL280 | -7.5 |
| RS004 | -7.5 |
| RL384 | -7.5 |
| RL386 | -7.5 |
| RL393 | -7.5 |
| RL398 | -7.5 |
| RL399 | -7.5 |
| RL350 | -7.5 |
| RL402 | -7.5 |
| RL411 | -7.5 |
| RL437 | -7.5 |
| RL438 | -7.5 |
| RL439 | -7.5 |
| RL459 | -7.5 |
| RL499 | -7.5 |
| RL502 | -7.5 |
| BC006 | -7.4 |
| BC012 | -7.4 |
| BD003 | -7.4 |
| BD017 | -7.4 |
| BD053 | -7.4 |
| BD054 | -7.4 |
| BD056 | -7.4 |
| BD081 | -7.4 |
| BL006 | -7.4 |
| BL010 | -7.4 |
| BS006 | -7.4 |
| GA001 | -7.4 |
| RL013 | -7.4 |
| RL014 | -7.4 |
| RL023 | -7.4 |
| RL034 | -7.4 |
| RL043 | -7.4 |
| RL071 | -7.4 |
| RL088 | -7.4 |
| RL104 | -7.4 |
| RL108 | -7.4 |
| RL142 | -7.4 |
| RL144 | -7.4 |
| RL165 | -7.4 |
| RL257 | -7.4 |
| RL265 | -7.4 |
| RL282 | -7.4 |
| RL298 | -7.4 |
| RR034 | -7.4 |
| RR044 | -7.4 |
| RR067 | -7.4 |
| RS003 | -7.4 |
| RL341 | -7.4 |
| RL412 | -7.4 |
| RL415 | -7.4 |
| RL423 | -7.4 |
| RL424 | -7.4 |
| RL456 | -7.4 |
| RL493 | -7.4 |
| RL528 | -7.4 |
| RL529 | -7.4 |
| RL530 | -7.4 |
| RL532 | -7.4 |
| BD062 | -7.3 |
| BD077 | -7.3 |
| BL003 | -7.3 |
| BL004 | -7.3 |
| BS053 | -7.3 |
| GC005 | -7.3 |
| GU006 | -7.3 |
| RC001 | -7.3 |
| RG015 | -7.3 |
| RL007 | -7.3 |
| RL012 | -7.3 |
| RL017 | -7.3 |
| RL021 | -7.3 |
| RL022 | -7.3 |
| RL026 | -7.3 |
| RL055 | -7.3 |
| RL075 | -7.3 |
| RL090 | -7.3 |
| RL101 | -7.3 |
| RL106 | -7.3 |
| RL137 | -7.3 |
| RL178 | -7.3 |
| RL205 | -7.3 |
| RL281 | -7.3 |
| RP001 | -7.3 |
| RP002 | -7.3 |
| RR016 | -7.3 |
| RR046 | -7.3 |
| RR049 | -7.3 |
| RL388 | -7.3 |
| RL389 | -7.3 |
| RL314 | -7.3 |
| RL329 | -7.3 |
| RL334 | -7.3 |
| RL351 | -7.3 |
| RL448 | -7.3 |
| RL453 | -7.3 |
| RL477 | -7.3 |
| RL501 | -7.3 |
| RL503 | -7.3 |
| RL504 | -7.3 |
| RL527 | -7.3 |
| RL533 | -7.3 |
| BD035 | -7.2 |
| BD052 | -7.2 |
| BD065 | -7.2 |
| BD074 | -7.2 |
| BD079 | -7.2 |
| BL005 | -7.2 |
| BS040 | -7.2 |
| BU002 | -7.2 |
| GC004 | -7.2 |
| RH001 | -7.2 |
| RL016 | -7.2 |
| RL025 | -7.2 |
| RL027 | -7.2 |
| RL030 | -7.2 |
| RL035 | -7.2 |
| RL063 | -7.2 |
| RL077 | -7.2 |
| RL153 | -7.2 |
| RL154 | -7.2 |
| RL200 | -7.2 |
| RL202 | -7.2 |
| RL216 | -7.2 |
| RL217 | -7.2 |
| RL240 | -7.2 |
| RL253 | -7.2 |
| RL258 | -7.2 |
| RL260 | -7.2 |
| RL279 | -7.2 |
| RL292 | -7.2 |
| RO001 | -7.2 |
| RR017 | -7.2 |
| RL397 | -7.2 |
| RL308 | -7.2 |
| RL320 | -7.2 |
| RL410 | -7.2 |
| RL417 | -7.2 |
| RL452 | -7.2 |
| RL510 | -7.2 |
| RL531 | -7.2 |
| RL539 | -7.2 |
| BD002 | -7.1 |
| BD038 | -7.1 |
| BD051 | -7.1 |
| BD060 | -7.1 |
| BP001 | -7.1 |
| BS044 | -7.1 |
| RD016 | -7.1 |
| RD020 | -7.1 |
| RL038 | -7.1 |
| RL039 | -7.1 |
| RL072 | -7.1 |
| RL099 | -7.1 |
| RL105 | -7.1 |
| RL146 | -7.1 |
| RL148 | -7.1 |
| RL176 | -7.1 |
| RL177 | -7.1 |
| RL244 | -7.1 |
| RL276 | -7.1 |
| RR003 | -7.1 |
| RR032 | -7.1 |
| RL395 | -7.1 |
| RL400 | -7.1 |
| RL305 | -7.1 |
| RL328 | -7.1 |
| RL401 | -7.1 |
| RL434 | -7.1 |
| RL444 | -7.1 |
| RL492 | -7.1 |
| RL494 | -7.1 |
| RL506 | -7.1 |
| RL511 | -7.1 |
| RL536 | -7.1 |
| RL538 | -7.1 |
| BD061 | -7 |
| BS051 | -7 |
| GC006 | -7 |
| GU007 | -7 |
| RD005 | -7 |
| RL051 | -7 |
| RL052 | -7 |
| RL054 | -7 |
| RL068 | -7 |
| RL147 | -7 |
| RL149 | -7 |
| RL161 | -7 |
| RL167 | -7 |
| RL175 | -7 |
| RL188 | -7 |
| RL190 | -7 |
| RL193 | -7 |
| RL224 | -7 |
| RL250 | -7 |
| RL266 | -7 |
| RL268 | -7 |
| RL289 | -7 |
| RL295 | -7 |
| RL299 | -7 |
| RR002 | -7 |
| RR006 | -7 |
| RL385 | -7 |
| RL387 | -7 |
| RL391 | -7 |
| RL396 | -7 |
| RL347 | -7 |
| RL348 | -7 |
| RL403 | -7 |
| RL404 | -7 |
| RL428 | -7 |
| RL491 | -7 |
| RL497 | -7 |
| RL500 | -7 |
| RL508 | -7 |
| RL509 | -7 |
| BB011 | -6.9 |
| BC016 | -6.9 |
| BL011 | -6.9 |
| BU005 | -6.9 |
| RD001 | -6.9 |
| RD029 | -6.9 |
| RL069 | -6.9 |
| RL070 | -6.9 |
| RL076 | -6.9 |
| RL120 | -6.9 |
| RL123 | -6.9 |
| RL145 | -6.9 |
| RL155 | -6.9 |
| RL156 | -6.9 |
| RL159 | -6.9 |
| RL210 | -6.9 |
| RL223 | -6.9 |
| RL232 | -6.9 |
| RL293 | -6.9 |
| RL297 | -6.9 |
| RO005 | -6.9 |
| RR035 | -6.9 |
| RR056 | -6.9 |
| RR069 | -6.9 |
| RS001 | -6.9 |
| RL390 | -6.9 |
| RL346 | -6.9 |
| RL408 | -6.9 |
| RL416 | -6.9 |
| RL450 | -6.9 |
| RL455 | -6.9 |
| RL490 | -6.9 |
| RL507 | -6.9 |
| RL525 | -6.9 |
| RL540 | -6.9 |
| BB009 | -6.8 |
| BD010 | -6.8 |
| BD029 | -6.8 |
| BD030 | -6.8 |
| BL007 | -6.8 |
| BP002 | -6.8 |
| BU003 | -6.8 |
| RB001 | -6.8 |
| RD025 | -6.8 |
| RH002 | -6.8 |
| RL057 | -6.8 |
| RL118 | -6.8 |
| RL211 | -6.8 |
| RL212 | -6.8 |
| RL213 | -6.8 |
| RL215 | -6.8 |
| RL249 | -6.8 |
| RL252 | -6.8 |
| RL271 | -6.8 |
| RR004 | -6.8 |
| RL392 | -6.8 |
| RL307 | -6.8 |
| RL313 | -6.8 |
| RL327 | -6.8 |
| RL342 | -6.8 |
| RL344 | -6.8 |
| RL405 | -6.8 |
| RL464 | -6.8 |
| RL547 | -6.8 |
| RL548 | -6.8 |
| BD050 | -6.7 |
| BS045 | -6.7 |
| GC002 | -6.7 |
| GE001 | -6.7 |
| GU009 | -6.7 |
| RD004 | -6.7 |
| RG017 | -6.7 |
| RL011 | -6.7 |
| RL032 | -6.7 |
| RL049 | -6.7 |
| RL053 | -6.7 |
| RL064 | -6.7 |
| RL092 | -6.7 |
| RL096 | -6.7 |
| RL117 | -6.7 |
| RL119 | -6.7 |
| RL158 | -6.7 |
| RL162 | -6.7 |
| RL164 | -6.7 |
| RL187 | -6.7 |
| RL197 | -6.7 |
| RL209 | -6.7 |
| RL219 | -6.7 |
| RL225 | -6.7 |
| RL231 | -6.7 |
| RL259 | -6.7 |
| RL264 | -6.7 |
| RL269 | -6.7 |
| RP068 | -6.7 |
| RR058 | -6.7 |
| RL421 | -6.7 |
| RL462 | -6.7 |
| RL463 | -6.7 |
| RL465 | -6.7 |
| RL526 | -6.7 |
| RL543 | -6.7 |
| RL544 | -6.7 |
| RL545 | -6.7 |
| BD004 | -6.6 |
| BS015 | -6.6 |
| BS046 | -6.6 |
| BU004 | -6.6 |
| GU008 | -6.6 |
| RD009 | -6.6 |
| RL028 | -6.6 |
| RL168 | -6.6 |
| RL170 | -6.6 |
| RL171 | -6.6 |
| RL172 | -6.6 |
| RL174 | -6.6 |
| RL183 | -6.6 |
| RL196 | -6.6 |
| RL214 | -6.6 |
| RL222 | -6.6 |
| RL251 | -6.6 |
| RL255 | -6.6 |
| RL256 | -6.6 |
| RL270 | -6.6 |
| RL272 | -6.6 |
| RP040 | -6.6 |
| RP067 | -6.6 |
| RP070 | -6.6 |
| RR011 | -6.6 |
| RR023 | -6.6 |
| RR024 | -6.6 |
| RR025 | -6.6 |
| RL309 | -6.6 |
| RL310 | -6.6 |
| RL349 | -6.6 |
| RL414 | -6.6 |
| RL446 | -6.6 |
| RL451 | -6.6 |
| RL475 | -6.6 |
| RL496 | -6.6 |
| BB002 | -6.5 |
| BC001 | -6.5 |
| BC002 | -6.5 |
| BD058 | -6.5 |
| BL002 | -6.5 |
| BL018 | -6.5 |
| RD006 | -6.5 |
| RL031 | -6.5 |
| RL081 | -6.5 |
| RL089 | -6.5 |
| RL094 | -6.5 |
| RL112 | -6.5 |
| RL189 | -6.5 |
| RL192 | -6.5 |
| RL221 | -6.5 |
| RL238 | -6.5 |
| RP062 | -6.5 |
| RP069 | -6.5 |
| RR045 | -6.5 |
| RR052 | -6.5 |
| RL356 | -6.5 |
| RL357 | -6.5 |
| RL360 | -6.5 |
| RL345 | -6.5 |
| RL409 | -6.5 |
| RL454 | -6.5 |
| RL461 | -6.5 |
| RL478 | -6.5 |
| RL546 | -6.5 |
| BB012 | -6.4 |
| BD001 | -6.4 |
| BD020 | -6.4 |
| BD059 | -6.4 |
| BL019 | -6.4 |
| RD007 | -6.4 |
| RD027 | -6.4 |
| RL113 | -6.4 |
| RL122 | -6.4 |
| RL138 | -6.4 |
| RL160 | -6.4 |
| RL163 | -6.4 |
| RL237 | -6.4 |
| RL274 | -6.4 |
| RR012 | -6.4 |
| RR051 | -6.4 |
| RL355 | -6.4 |
| RL413 | -6.4 |
| RL419 | -6.4 |
| BD057 | -6.3 |
| BD084 | -6.3 |
| BL009 | -6.3 |
| BS014 | -6.3 |
| BS074 | -6.3 |
| BT001 | -6.3 |
| RD028 | -6.3 |
| RL116 | -6.3 |
| RL143 | -6.3 |
| RL195 | -6.3 |
| RL198 | -6.3 |
| RL226 | -6.3 |
| RL294 | -6.3 |
| RP073 | -6.3 |
| RP078 | -6.3 |
| RL458 | -6.3 |
| RL479 | -6.3 |
| RL549 | -6.3 |
| BB008 | -6.2 |
| BS001 | -6.2 |
| BS018 | -6.2 |
| BS064 | -6.2 |
| BU006 | -6.2 |
| RG016 | -6.2 |
| RL199 | -6.2 |
| RL254 | -6.2 |
| RP014 | -6.2 |
| RP057 | -6.2 |
| RP063 | -6.2 |
| RR042 | -6.2 |
| RR057 | -6.2 |
| RR064 | -6.2 |
| RR065 | -6.2 |
| RL311 | -6.2 |
| RL318 | -6.2 |
| RL441 | -6.2 |
| RL449 | -6.2 |
| RL457 | -6.2 |
| RL495 | -6.2 |
| BD015 | -6.1 |
| BD018 | -6.1 |
| BP003 | -6.1 |
| BS065 | -6.1 |
| BS073 | -6.1 |
| RD015 | -6.1 |
| RD022 | -6.1 |
| RL018 | -6.1 |
| RL085 | -6.1 |
| RL166 | -6.1 |
| RL173 | -6.1 |
| RL194 | -6.1 |
| RL220 | -6.1 |
| RL241 | -6.1 |
| RL273 | -6.1 |
| RP005 | -6.1 |
| RP034 | -6.1 |
| RP037 | -6.1 |
| RP047 | -6.1 |
| RP074 | -6.1 |
| RR001 | -6.1 |
| RR007 | -6.1 |
| RR008 | -6.1 |
| RR026 | -6.1 |
| RR037 | -6.1 |
| RR038 | -6.1 |
| RR054 | -6.1 |
| RR066 | -6.1 |
| RL358 | -6.1 |
| RL316 | -6.1 |
| RL317 | -6.1 |
| RL420 | -6.1 |
| RL429 | -6.1 |
| RL481 | -6.1 |
| BL001 | -6 |
| BL008 | -6 |
| BL015 | -6 |
| BL017 | -6 |
| BZ002 | -6 |
| RD014 | -6 |
| RD018 | -6 |
| RD019 | -6 |
| RG018 | -6 |
| RL169 | -6 |
| RL288 | -6 |
| RP026 | -6 |
| RP051 | -6 |
| RP072 | -6 |
| RP075 | -6 |
| RR047 | -6 |
| RL359 | -6 |
| RL319 | -6 |
| BB006 | -5.9 |
| BL013 | -5.9 |
| BS052 | -5.9 |
| RD002 | -5.9 |
| RD011 | -5.9 |
| RD023 | -5.9 |
| RD026 | -5.9 |
| RL061 | -5.9 |
| RL087 | -5.9 |
| RL124 | -5.9 |
| RL157 | -5.9 |
| RO011 | -5.9 |
| RP029 | -5.9 |
| RP038 | -5.9 |
| RP042 | -5.9 |
| RP066 | -5.9 |
| RP071 | -5.9 |
| RR010 | -5.9 |
| RR059 | -5.9 |
| RR061 | -5.9 |
| RL483 | -5.9 |
| BL012 | -5.8 |
| BS016 | -5.8 |
| BS048 | -5.8 |
| BZ003 | -5.8 |
| RD010 | -5.8 |
| RD012 | -5.8 |
| RD017 | -5.8 |
| RL121 | -5.8 |
| RO004 | -5.8 |
| RP009 | -5.8 |
| RP018 | -5.8 |
| RP027 | -5.8 |
| RP031 | -5.8 |
| RP033 | -5.8 |
| RP041 | -5.8 |
| RP053 | -5.8 |
| RR009 | -5.8 |
| RR036 | -5.8 |
| RR041 | -5.8 |
| RR043 | -5.8 |
| RR050 | -5.8 |
| RR070 | -5.8 |
| RL445 | -5.8 |
| RL482 | -5.8 |
| RL523 | -5.8 |
| BS042 | -5.7 |
| GA010 | -5.7 |
| RO010 | -5.7 |
| RP008 | -5.7 |
| RP028 | -5.7 |
| RP039 | -5.7 |
| RP043 | -5.7 |
| RP077 | -5.7 |
| RL480 | -5.7 |
| BD012 | -5.6 |
| BL016 | -5.6 |
| BL020 | -5.6 |
| BS017 | -5.6 |
| BS027 | -5.6 |
| BS049 | -5.6 |
| RD008 | -5.6 |
| RD024 | -5.6 |
| RL062 | -5.6 |
| RL091 | -5.6 |
| RO002 | -5.6 |
| RP010 | -5.6 |
| RP011 | -5.6 |
| RP015 | -5.6 |
| RP017 | -5.6 |
| RP020 | -5.6 |
| RP052 | -5.6 |
| RR028 | -5.6 |
| RR030 | -5.6 |
| RR040 | -5.6 |
| RR053 | -5.6 |
| RR060 | -5.6 |
| RR063 | -5.6 |
| BE001 | -5.5 |
| BL014 | -5.5 |
| RL107 | -5.5 |
| RO003 | -5.5 |
| RP004 | -5.5 |
| RP006 | -5.5 |
| RP050 | -5.5 |
| RP054 | -5.5 |
| RP056 | -5.5 |
| RP076 | -5.5 |
| RR039 | -5.5 |
| RR055 | -5.5 |
| RR068 | -5.5 |
| BS043 | -5.4 |
| BS047 | -5.4 |
| BS050 | -5.4 |
| BS066 | -5.4 |
| BZ001 | -5.4 |
| RD003 | -5.4 |
| RD021 | -5.4 |
| RO008 | -5.4 |
| RP003 | -5.4 |
| RP012 | -5.4 |
| RP019 | -5.4 |
| RP032 | -5.4 |
| RP060 | -5.4 |
| RP061 | -5.4 |
| BB001 | -5.3 |
| BB010 | -5.3 |
| RD013 | -5.3 |
| RL058 | -5.3 |
| RL060 | -5.3 |
| RP016 | -5.3 |
| RP025 | -5.3 |
| RP036 | -5.3 |
| RP046 | -5.3 |
| RR048 | -5.3 |
| RR062 | -5.3 |
| RL534 | -5.3 |
| BB003 | -5.2 |
| BB004 | -5.2 |
| BB005 | -5.2 |
| RO007 | -5.2 |
| RP024 | -5.2 |
| RP030 | -5.2 |
| RP045 | -5.2 |
| RP049 | -5.2 |
| RP064 | -5.2 |
| BE009 | -5.1 |
| RL059 | -5.1 |
| RO009 | -5.1 |
| RP007 | -5.1 |
| RP013 | -5.1 |
| RP021 | -5.1 |
| RP023 | -5.1 |
| RR029 | -5.1 |
| BE006 | -5 |
| RP022 | -5 |
| RP035 | -5 |
| RP044 | -5 |
| RP055 | -5 |
| BB007 | -4.9 |
| RP059 | -4.9 |
| BD048 | -4.8 |
| RP048 | -4.8 |
| RP058 | -4.8 |
| RL333 | -4.8 |
| BD033 | -1.2 |
| BD036 | -1.2 |
| BD037 | -1 |

**Table S3**. Docking-interacting residues of GA002, BE012, and RC003 and their relationship to the mapped conserved RdRp motif regions and predicted functional regions.

| **Complex** | **Docking-interacting residues** | **Overlap with mapped conserved motifs** | **Relationship to predicted functional regions** |
| --- | --- | --- | --- |
| GA002 | 233, 285, 287, 311, 315, 429, and 534 | 233 and 429 | Motif 2 (231-237) and Motif 5 (426-446) |
| BE012 | 217, 234, 296, 304, 308, 311, 312, 316, 429, 432, and 433 | 234, 296, 429, and 432 | Motif 2 (231-237), Motif 3/canonical Motif F (291-297), and Motif 5 (426-446) |
| RC003 | 541, 589, 594, 607, 608, and 809 | None | No overlap with the eight mapped conserved motif regions |

**Table S4**. Correspondence between IMNV/Totiviridae motif numbering and canonical viral RdRp motifs in AlphaFold 3 model_1.

| **IMNV motif** | **Canonical RdRp motif** | **Residues** | **Motif sequence** |
| --- | --- | --- | --- |
| Motif 1 | Additional Totiviridae motif | 165-172 | IVGYMDPN |
| Motif 2 | Additional Totiviridae motif | 231-237 | WLTSGSS |
| Motif 3 | F | 291-297 | SKLRLAV |
| Motif 4 | A | 354-363 | DYASFDHQPT |
| Motif 5 | B | 426-446 | SGVRITSLLGNMWNAIITKIA |
| Motif 6 | C | 466-468 | GDD |
| Motif 7 | D | 506-509 | EFLR |
| Motif 8 | E | 516-523 | GVRGWTCR |

**Table S5**. Local structural confidence of conserved RdRp motifs and predicted ligand-binding residues in the AlphaFold 3 model_1 of IMNV RdRp.

| **Region** | **Residues** | **Mean pLDDT** | **Mean intra-region PAE (Å)** |
| --- | --- | --- | --- |
| Motif 1 | 165-172 | 91.18 | 1.45 |
| Motif 2 | 231-237 | 72.61 | 3.21 |
| Motif 3 | 291-297 | 87.22 | 1.57 |
| Motif 4 | 354-363 | 88.68 | 1.47 |
| Motif 5 | 426-446 | 92.69 | 1.50 |
| Motif 6 | 466-468 | 91.05 | 0.88 |
| Motif 7 | 506-509 | 91.18 | 0.91 |
| Motif 8 | 516-523 | 93.98 | 1.19 |

**Table S6**. Local structural confidence of predicted ligand-binding regions in the AlphaFold 3 model_1 of IMNV RdRp.

| **Region** | **Interacting Residues** | **Mean pLDDT** | **Mean pocket PAE (Å)** |
| --- | --- | --- | --- |
| GA002 binding residues | 233, 285, 287, 311, 315, 429, 534 | 84.53 | 3.43 |
| RC003 binding residues | 541, 589, 594, 607, 608, 809 | 87.15 | 2.55 |
| BE012 binding residues | 217, 234, 296, 304, 308, 311, 312, 316, 429, 432, 433 | 87.51 | 2.51 |

**Figure S1**. The 2D structures of the six selected ligands: (**A**) GA002, (**B**) RC003, (**C**) BE012, (**D**) RL354, (**E**) RL015, and (**F**) BS067. The structures retain the stereochemical information provided by the Seaweed Metabolite Database.

**Figure S2**. The 3D structures of the six selected ligands: (**A**) GA002 (Red), (**B**) RC003 (Purple), (**C**) BE012 (Black), (**D**) RL354 (Blue), (**E**) RL015 (Yellow), and (**F**) BS067 (Pink), corresponding to the structures used for molecular docking.

**Figure S3**. Complex view (Left), 3D interaction view (Middle), and 2D interaction map (Right) of the protein RdRp_M01 interactions with ligands: (**A**) RL354 (Blue), (**B**) RL015 (Yellow), and (**C**) BS067 (Pink).

**Figure S4**. Putative catalytic region of the IMNV RdRp. (**A**) Overall AlphaFold 3 Model 1 structure and (**B**) Enlarged view showing the conserved acidic residues Asp354 and Asp359 and the G466-D467-D468 GDD motif.

**Figure S5**. Putative entry-associated regions of the IMNV RdRp. (A) Putative NTP-entry-associated region corresponding to Motif F (residues 291-297) and (B) putative RNA-template entry-associated region associated with IMNV Motif 2 (residues 231-237).
