# Supplementary material for: Deep learning-based structural prediction (AlphaFold 3) of shrimp IMNV RdRp and identification of seaweed-derived metabolites for antiviral intervention in aquaculture
Source: Virus Res. 2026 Sep 8;373:199796. doi: 10.1016/j.virusres.2026.199796 (PMC13595083; doi:10.1016/j.virusres.2026.199796)
Supplement: Supplementary file 2 [file mmc2.docx]

**Supporting Information 2**

**Supplementary 2A**. Pairwise BLASTP alignment between the IMNV ORF2-encoded putative RdRp protein sequence and the putative RNA-dependent RNA polymerase of Totiviridae sp. (UHS72541.1).

```
Query: unnamed protein product Query ID: lcl|Query_4838030 Length: 838
>MAG: putative RNA-dependent RNA polymerase [Totiviridae sp.]
Sequence ID: UHS72541.1 Length: 833
Range 1: 10 to 833
Score:1061 bits(2744), Expect:0.0,
Method:Compositional matrix adjust.,
Identities:494/825(60%), Positives:628/825(76%), Gaps:1/825(0%)
Query  14   QELTKGIYNASTLTPKIVDQIIKDEGSGTDKTKYVNVPKIITDKELQTFYVPRSNADLVI  73
            QEL+ G+ N  TLT K V  I K      D++KYV +P   TD +LQ  + PR+  DL +
Sbjct  10   QELSDGVINRKTLTWKSVIHIAKQSVGQNDQSKYVEIPSHTTDIQLQEIFKPRAKVDLSL  69

Query  74   RRIRLIDLWRNLKPDQMDEIRNYTHLDYIFVQNICIYMLVFGINTVKHFRQIGLFNERNE  133
            RR R++DL R+   +Q  E+  YTHLDYIFVQNI +YM+VFG+ T++  R  G+FN R
Sbjct  70   RRFRMVDLLRHSTYEQRLELGRYTHLDYIFVQNIFVYMIVFGVPTLRSLRATGMFNNREL  129

Query  134  FIEIAKQLSTKGKRFVDDVDNMKQKVCEIATIVGYMDPNVDKIDVMEEVNSLAAEGNEHG  193
            F+  AK+LST  KR+VDD DN KQKVCE+ATI+GYM+P +D+++V+EEV+SLAA GN HG
Sbjct  130  FLTAAKELSTLAKRYVDDRDNAKQKVCELATIIGYMNPAIDELNVLEEVHSLAAPGNPHG  189

Query  194  IDRDNWNDLFTKTCKEVMTWYKGHEFISFDDYIKEGMWLTSGSSSIGKVHWTKDGENGKF  253
            ++R  W + F    +E+M +   H+FI F+ Y+KEG WLTSGSSSIG V W  + E G F
Sbjct  190  VNRHCWQNEFDAQVREIMEYTPIHDFIPFELYVKEGKWLTSGSSSIGTVEWFANDEKGHF  249

Query  254  KARKNMLLQIYTPQELANIVYAWDGKLHSRVFIKNEMSKLRLAVASNIEAYIHESYMLFL  313
            KARKNML Q+YTP ++ N+   WDGKL SRVFIKNEMSKLRLAVASNIEAYIHESYML+L
Sbjct  250  KARKNMLTQLYTPDDIYNMCVHWDGKLKSRVFIKNEMSKLRLAVASNIEAYIHESYMLYL  309

Query  314  YGHGFKEYFGVTLDEKPDQQHQREIEMIEKLQAGYFGLPFDYASFDHQPTTFEVKTMVRR  373
            YGHGFK YFG+TLDE P  QH REIEM++ LQ G +G PFDY SFDHQPTT E++++++R
Sbjct  310  YGHGFKNYFGITLDESPTAQHIREIEMVKMLQDGAYGFPFDYKSFDHQPTTSEIQSIIKR  369

Query  374  VGEIVVSQVPKNYYYQTQLLVNKIVNAYDKSYLSGNIKNTKFENIKVKGGVPSGVRITSL  433
            V  I++ Q+P  YY +   L  +I+++Y  SYLSG I +  F +++VKGGVPSGVR TSL
Sbjct  370  VISIIMPQIPSRYYTECNHLFTRILDSYTNSYLSGEINDITFRDVRVKGGVPSGVRSTSL  429

Query  434  LGNMWNAIITKIAINNVIGIIGYDPISQISLRGDDVAILSKDPAALYLLRLSYAAINAIG  493
            +GN+WN+ +T  AI      IG  P   ISLRGDDVAILS +P ALY LR++YAAINAIG
Sbjct  430  IGNLWNSCVTHKAIEITESQIGQKPFKHISLRGDDVAILSHNPMALYALRVNYAAINAIG  489

Query  494  KDSKLGISPKVCEFLRNEISVTGVRGWTCRGIGGISQRKPWNPQPWSPNDEVETNASNIS  553
             DSKLGISPK+CEFLRNEI   G+RGWTCRGIGG+SQRKPWNP PWSPN EV TNA+NI
Sbjct  490  LDSKLGISPKICEFLRNEIRTDGIRGWTCRGIGGLSQRKPWNPSPWSPNAEVTTNAANIG  549

Query  554  LLERRAGIELQQLHHINKVKWSRHVRQSYKYLELPKRLGGFGIYRFQGWLPNGKLPLAKK  613
            LLERR G ++ QLH INK+KWS+H+RQSYK+LELP RLGGFG+Y FQGWLPN KLPLAKK
Sbjct  550  LLERRVGYQIPQLHQINKIKWSKHMRQSYKWLELPIRLGGFGLYPFQGWLPNCKLPLAKK  609

Query  614  PLVNVEDIHPSQELFLPLSEQQKKILAQVEMTNKMQTDDIPGTQKLFSKEWIQKVRAKKI  673
            PLV+V+++HPS   F+ L++QQ  +LA++EMTNKMQTDDIPGTQKLFSKEWI +V+A K
Sbjct  610  PLVDVQNVHPSFTPFITLNQQQTALLAKIEMTNKMQTDDIPGTQKLFSKEWINQVKATKC  669

Query  674  IWSRNQTIPIHTDHTVRIPKWDEKIKFPRYKSEYILNNKINLTMEQVLRQYNLLKEVERH  733
            +W++ Q         + IP+WD   +FP Y  +YI +++INLT EQ+LRQYNLLKEV+R+
Sbjct  670  LWNKTQIFFGRRKPPI-IPEWDCTKQFPSYGGKYITSSRINLTFEQLLRQYNLLKEVKRY  728

Query  734  DKDLKVPKLLDILDKWFPVQSSKIKTYESQGFHRTDAINLAVGEIPTEPAVKINPILINF  793
            DK  ++PKL+D+L  +FP   S+I+ +E QGFHRTDAINLA+GEIP+EP +KINPIL  F
Sbjct  729  DKSFELPKLMDVLQNYFPAVESEIRQFEKQGFHRTDAINLAIGEIPSEPTIKINPILTRF  788

Query  794  VKLHLERQGITHQRGRNKIAKFIYQKTKQAENMILQSSLQQMYRY  838
            VK H+++ G+  QRGR  IA F+Y ++  A+  +L+S+L QMYRY
Sbjct  789  VKQHIDKAGVKFQRGRRSIAYFLYHQSASAQAALLKSTLNQMYRY  833
```

**Supplementary 2B**. BLASTP analysis of the IMNV ORF2-encoded putative RdRp protein against the NCBI ClusteredNR database, showing significant Totiviridae hits and pairwise sequence alignments with representative hits.

```
RID: 8UA75EJC014
Job Title:Protein Sequence
Program: BLASTP
Database: ClusteredNR clustered nr
Query #1:  Query ID: lcl|Query_4838030 Length: 838

Clusters producing significant alignments:
                                                                  #          #          Scientific      Cluster                    Max    Total Query   E    Per.   Acc.
Cluster Rep.                                                      Mem.       Taxa       Name            Ancestor        Taxid      Score  Score cover Value  Ident  Len        Accession
MAG: putative RNA-dependent RNA polymerase [Totiviridae sp.]      1          1          Totiviridae sp. viruses         1955172    1061   1061  98%   0.0    59.88  833        UHS72541.1
MAG: putative RNA-dependent RNA polymerase, partial [Totivirid... 1          1          Totiviridae sp. viruses         1955172    782    782   63%   0.0    69.81  534        UHS72568.1
MAG: putative RNA-dependent RNA polymerase [Totiviridae sp.]      1          1          Totiviridae sp. viruses         1955172    702    702   96%   0.0    44.83  911        UHS72444.1
MAG: putative RNA-dependent RNA polymerase [Totiviridae sp.]      2          1          Totiviridae sp. viruses         1955172    641    641   89%   0.0    44.05  760        UHS72493.1
MAG: putative RNA-dependent RNA polymerase [Totiviridae sp.]      1          1          Totiviridae sp. viruses         1955172    628    628   94%   0.0    42.19  807        UHS72478.1
MAG: putative RNA-dependent RNA polymerase, partial [Totivirid... 1          1          Totiviridae sp. viruses         1955172    626    626   88%   0.0    43.89  739        UHS72503.1
MAG: putative RNA-dependent RNA polymerase, partial [Totivirid... 2          1          Totiviridae sp. viruses         1955172    622    622   92%   0.0    42.31  851        UHS72552.1
MAG: putative RNA-dependent RNA polymerase [Totiviridae sp.]      1          1          Totiviridae sp. viruses         1955172    603    603   93%   0.0    41.80  799        UHS72448.1
MAG: putative RNA-dependent RNA polymerase, partial [Totivirid... 1          1          Totiviridae sp. viruses         1955172    598    598   85%   0.0    42.99  780        UHS72466.1
MAG: putative RNA-dependent RNA polymerase, partial [Totivirid... 1          1          Totiviridae sp. viruses         1955172    597    597   78%   0.0    46.04  687        UHS72514.1
MAG: putative RNA-dependent RNA polymerase, partial [Totivirid... 1          1          Totiviridae sp. viruses         1955172    580    580   80%   0.0    44.38  800        UHS72496.1
MAG: putative RNA-dependent RNA polymerase [Totiviridae sp.]      1          1          Totiviridae sp. viruses         1955172    566    566   100%  0.0    37.75  869        UHS72487.1
MAG: putative RNA-dependent RNA polymerase, partial [Totivirid... 1          1          Totiviridae sp. viruses         1955172    548    548   46%   0.0    68.32  403        UHS72509.1
MAG: putative RNA-dependent RNA polymerase, partial [Totivirid... 1          1          Totiviridae sp. viruses         1955172    538    538   42%   0.0    71.10  361        UHS71733.1
MAG: putative RNA-dependent RNA polymerase [Totiviridae sp.]      1          1          Totiviridae sp. viruses         1955172    537    537   87%   0.0    40.59  734        UHS72421.1
MAG: putative RNA-dependent RNA polymerase [Totiviridae sp.]      1          1          Totiviridae sp. viruses         1955172    516    516   87%   4e-174 40.29  738        UHS72429.1
MAG: putative RNA-dependent RNA polymerase [Totiviridae sp.]      1          1          Totiviridae sp. viruses         1955172    510    510   94%   4e-171 37.74  787        UHS72490.1
MAG: putative RNA-dependent RNA polymerase, partial [Totivirid... 1          1          Totiviridae sp. viruses         1955172    498    498   77%   3e-168 41.32  647        UHS72526.1
MAG: putative RNA-dependent RNA polymerase [Totiviridae sp.]      1          1          Totiviridae sp. viruses         1955172    492    492   92%   3e-164 37.21  772        UHS72476.1
MAG: putative RNA-dependent RNA polymerase, partial [Totivirid... 2          1          Totiviridae sp. viruses         1955172    457    457   59%   2e-154 45.45  497        UHS72534.1
MAG: putative RNA-dependent RNA polymerase, partial [Totivirid... 2          1          Totiviridae sp. viruses         1955172    438    438   64%   7e-146 42.91  583        UHS72482.1
MAG: putative RNA-dependent RNA polymerase, partial [Totivirid... 1          1          Totiviridae sp. viruses         1955172    416    416   65%   8e-138 41.80  531        UHS72491.1
MAG: putative RNA-dependent RNA polymerase, partial [Totivirid... 1          1          Totiviridae sp. viruses         1955172    407    407   47%   3e-136 50.00  403        UHS72480.1
MAG: putative RNA-dependent RNA polymerase, partial [Totivirid... 1          1          Totiviridae sp. viruses         1955172    400    400   62%   9e-132 40.23  549        UHS72532.1
MAG: putative RNA-dependent RNA polymerase, partial [Totivirid... 1          1          Totiviridae sp. viruses         1955172    391    391   45%   1e-130 52.51  371        UHS72560.1
MAG: putative RNA-dependent RNA polymerase, partial [Totivirid... 1          1          Totiviridae sp. viruses         1955172    368    368   49%   4e-121 45.30  416        UHS72546.1
MAG: putative RNA-dependent RNA polymerase, partial [Totivirid... 1          1          Totiviridae sp. viruses         1955172    367    367   51%   1e-120 44.29  433        UHS72488.1
MAG: putative RNA-dependent RNA polymerase, partial [Totivirid... 1          1          Totiviridae sp. viruses         1955172    343    343   40%   1e-112 49.10  333        UHS72468.1
MAG: putative RNA-dependent RNA polymerase, partial [Totivirid... 1          1          Totiviridae sp. viruses         1955172    343    343   44%   2e-112 47.41  364        UHS72564.1
MAG: putative RNA-dependent RNA polymerase, partial [Totivirid... 1          1          Totiviridae sp. viruses         1955172    347    347   56%   5e-112 41.09  508        UHS72558.1
MAG: putative RNA-dependent RNA polymerase, partial [Totivirid... 1          1          Totiviridae sp. viruses         1955172    334    334   39%   2e-109 50.15  340        UHS72525.1
MAG: putative RNA-dependent RNA polymerase, partial [Totivirid... 1          1          Totiviridae sp. viruses         1955172    334    334   45%   6e-108 44.39  417        UHS72522.1
MAG: putative RNA-dependent RNA polymerase, partial [Totivirid... 1          1          Totiviridae sp. viruses         1955172    337    337   56%   6e-108 39.75  494        UHS72417.1
MAG: putative RNA-dependent RNA polymerase, partial [Totivirid... 1          1          Totiviridae sp. viruses         1955172    326    326   50%   6e-105 40.98  426        UHS72432.1
MAG: putative RNA-dependent RNA polymerase, partial [Totivirid... 1          1          Totiviridae sp. viruses         1955172    302    302   56%   4e-95  37.95  478        UHS72538.1
MAG: putative RNA-dependent RNA polymerase, partial [Totivirid... 1          1          Totiviridae sp. viruses         1955172    285    285   50%   7e-89  38.26  465        UHS72415.1
MAG: putative RNA-dependent RNA polymerase, partial [Totivirid... 1          1          Totiviridae sp. viruses         1955172    278    278   36%   9e-88  46.00  352        UHS72416.1
MAG: putative RNA-dependent RNA polymerase, partial [Totivirid... 1          1          Totiviridae sp. viruses         1955172    272    272   45%   6e-85  39.11  378        UHS72413.1
MAG: putative RNA-dependent RNA polymerase, partial [Totivirid... 1          1          Totiviridae sp. viruses         1955172    262    262   39%   7e-82  41.90  330        UHS72556.1
MAG: putative RNA-dependent RNA polymerase, partial [Totivirid... 1          1          Totiviridae sp. viruses         1955172    259    259   41%   1e-80  42.07  346        UHS72537.1
MAG: putative RNA-dependent RNA polymerase, partial [Totivirid... 1          1          Totiviridae sp. viruses         1955172    254    254   40%   5e-79  40.65  337        UHS72524.1
MAG: putative RNA-dependent RNA polymerase, partial [Totivirid... 1          1          Totiviridae sp. viruses         1955172    218    218   37%   3e-65  37.30  350        UHS72528.1
MAG: RNA-dependent RNA polymerase [Totiviridae sp.]               4          1          Totiviridae sp. viruses         1955172    230    230   88%   7e-65  27.43  960        WAK77453.1
MAG: putative RNA-dependent RNA polymerase, partial [Totivirid... 1          1          Totiviridae sp. viruses         1955172    216    216   41%   3e-64  36.78  372        UHS72569.1
MAG: RNA-dependent RNA polymerase [Totiviridae sp.]               12         1          Totiviridae sp. viruses         1955172    225    225   75%   4e-63  28.06  959        WAK77419.1
MAG: putative RNA-dependent RNA polymerase, partial [Totivirid... 1          1          Totiviridae sp. viruses         1955172    206    206   28%   2e-61  42.80  295        UHS72475.1
MAG: RNA-dependent RNA polymerase [Totiviridae sp.]               1          1          Totiviridae sp. viruses         1955172    219    219   75%   4e-61  28.79  938        WAK77558.1
MAG: RNA-dependent RNA polymerase [Totiviridae sp.]               5          1          Totiviridae sp. viruses         1955172    216    216   76%   1e-60  27.62  833        WAK77448.1
MAG: RNA-dependent RNA polymerase [Totiviridae sp.]               5          1          Totiviridae sp. viruses         1955172    218    218   71%   2e-60  27.49  1011       WAK77483.1
MAG: putative RNA-dependent RNA polymerase, partial [Totivirid... 1          1          Totiviridae sp. viruses         1955172    202    202   34%   4e-60  38.44  316        UHS72455.1
MAG: RNA-dependent RNA polymerase [Totiviridae sp.]               14         2          unclassified... viruses         3415261    212    212   75%   7e-59  27.26  932        WAK77607.1
MAG: RNA-dependent RNA polymerase, partial [Totiviridae sp.]      1          1          Totiviridae sp. viruses         1955172    211    211   65%   8e-59  28.92  826        WAK77488.1
MAG: RNA-dependent RNA polymerase [Totiviridae sp.]               3          1          Totiviridae sp. viruses         1955172    212    212   81%   9e-59  27.00  938        WAK77258.1
MAG: putative RNA-dependent RNA polymerase, partial [Totivirid... 1          1          Totiviridae sp. viruses         1955172    199    199   26%   1e-58  42.73  351        UHS72457.1
MAG: RNA-dependent RNA polymerase [Totiviridae sp.]               3          1          Totiviridae sp. viruses         1955172    211    211   75%   2e-58  26.75  932        WAK77681.1
MAG: RNA-dependent RNA polymerase [Totiviridae sp.]               4          1          Totiviridae sp. viruses         1955172    208    208   75%   8e-58  26.24  830        WAK77316.1
MAG: RNA-dependent RNA polymerase [Totiviridae sp.]               1          1          Totiviridae sp. viruses         1955172    206    206   70%   3e-57  28.59  802        WAK77387.1
MAG: RNA-dependent RNA polymerase [Totiviridae sp.]               8          1          Totiviridae sp. viruses         1955172    205    205   75%   8e-57  26.17  844        WAK77262.1
MAG: RNA-dependent RNA polymerase [Totiviridae sp.]               7          1          Totiviridae sp. viruses         1955172    204    204   72%   3e-56  27.17  871        WAK77610.1
MAG: RNA-dependent RNA polymerase [Totiviridae sp.]               12         1          Totiviridae sp. viruses         1955172    203    203   71%   1e-55  27.27  1053       UUT42649.1
MAG: RNA-dependent RNA polymerase, partial [Totiviridae sp.]      5          2          unclassified... viruses         3415261    202    202   71%   3e-55  27.22  1048       WAK77747.1
MAG: RNA-dependent RNA polymerase, partial [Totiviridae sp.]      1          1          Totiviridae sp. viruses         1955172    202    202   70%   5e-55  27.49  1105       WAK77284.1
MAG: RNA-dependent RNA polymerase [Totiviridae sp.]               4          2          unclassified... viruses         3415261    199    199   75%   9e-55  26.40  854        WAK77254.1
MAG: RNA-dependent RNA polymerase [Totiviridae sp.]               26         1          Totiviridae sp. viruses         1955172    199    199   71%   2e-54  27.40  926        WAK77427.1
MAG: putative RNA-dependent RNA polymerase, partial [Totivirid... 1          1          Totiviridae sp. viruses         1955172    190    190   31%   3e-54  38.40  426        UHS72484.1
MAG: RNA-dependent RNA polymerase [Totiviridae sp.]               1          1          Totiviridae sp. viruses         1955172    198    198   71%   4e-54  26.29  952        WAK77278.1
MAG: RNA-dependent RNA polymerase [Totiviridae sp.]               2          1          Totiviridae sp. viruses         1955172    197    197   65%   5e-54  29.43  830        WAK77616.1
MAG: RNA-dependent RNA polymerase, partial [Totiviridae sp.]      1          1          Totiviridae sp. viruses         1955172    194    194   58%   6e-54  29.39  637        WAK77676.1
RNA-dependent RNA polymerase [Yanbian Totiv tick virus 1]         14         2          unclassified... viruses         3415261    197    197   71%   1e-53  26.97  945        XFO99082.1
MAG: RNA-dependent RNA polymerase [Totiviridae sp.]               9          2          unclassified... viruses         3415261    191    191   69%   5e-52  27.83  809        WAK77722.1
MAG: RNA-dependent RNA polymerase [Totiviridae sp.]               1          1          Totiviridae sp. viruses         1955172    191    191   71%   1e-51  25.67  942        WAK77292.1
RNA-dependent RNA polymerase, partial [Totiviridae sp.]           2          1          Totiviridae sp. viruses         1955172    187    187   75%   3e-51  24.59  708        XCG98880.1
MAG: RNA-dependent RNA polymerase [Totiviridae sp.]               2          1          Totiviridae sp. viruses         1955172    188    188   74%   7e-51  24.54  870        WAK77583.1
MAG: putative RNA-dependent RNA polymerase [Totiviridae sp.]      2          1          Totiviridae sp. viruses         1955172    187    187   75%   2e-50  24.59  943        XZG29191.1
MAG: RNA-dependent RNA polymerase [Totiviridae sp.]               107        3          unclassified... viruses         3415261    186    186   76%   3e-50  24.26  831        WAK77535.1
RNA-dependent RNA polymerase [Totiviridae sp.]                    73         2          unclassified... viruses         3415261    184    184   74%   9e-50  23.80  831        XCG98881.1
MAG: RNA-dependent RNA polymerase [Totiviridae sp.]               5          1          Totiviridae sp. viruses         1955172    182    182   70%   4e-49  25.04  803        WAK77658.1
MAG: RNA-dependent RNA polymerase [Totiviridae sp.]               1          1          Totiviridae sp. viruses         1955172    182    182   74%   3e-48  24.25  2832       WAK77245.1
MAG: RNA-dependent RNA polymerase, partial [Totiviridae sp.]      1          1          Totiviridae sp. viruses         1955172    172    172   55%   3e-47  26.92  535        WAK77504.1
MAG: RNA-dependent RNA polymerase, partial [Totiviridae sp.]      1          1          Totiviridae sp. viruses         1955172    172    172   50%   1e-46  28.99  629        WAK77481.1
MAG: RNA-dependent RNA polymerase [Totiviridae sp.]               14         2          unclassified... viruses         3415261    173    173   52%   1e-46  28.76  704        WAK77695.1
MAG: putative RNA dependent RNA polymerase, partial [Totivirid... 1          1          Totiviridae sp. viruses         1955172    156    156   38%   5e-43  31.83  380        WWV90225.1
MAG: RNA-dependent RNA polymerase, partial [Totiviridae sp.]      1          1          Totiviridae sp. viruses         1955172    154    154   49%   1e-40  28.80  617        WAK77298.1
MAG: RNA-dependent RNA polymerase, partial [Totiviridae sp.]      1          1          Totiviridae sp. viruses         1955172    152    152   47%   1e-40  28.50  526        WAK77652.1
MAG: RNA-dependent RNA polymerase, partial [Totiviridae sp.]      1          1          Totiviridae sp. viruses         1955172    123    123   37%   8e-32  28.79  365        WAK77755.1
MAG: putative RNA dependent RNA polymerase, partial [Totivirid... 1          1          Totiviridae sp. viruses         1955172    103    103   20%   5e-27  34.91  167        WWV90304.1
MAG: RNA-dependent polymerase [Totiviridae sp.]                   1          1          Totiviridae sp. viruses         1955172    102    102   17%   1e-25  35.33  235        WAK77683.1
MAG: nucleocapsid protein, partial [Totiviridae sp.]              1          1          Totiviridae sp. viruses         1955172    92.8   92.8  31%   3e-22  28.36  263        QYW06889.1
MAG: RNA-dependent RNA polymerase [Totiviridae sp.]               1          1          Totiviridae sp. viruses         1955172    59.3   59.3  13%   1e-11  33.91  159        WAK77592.1
RNA-dependent RNA polymerase [Totiviridae sp.]                    1          1          Totiviridae sp. viruses         1955172    46.6   46.6  45%   2e-06  18.48  784        BDC79619.1
RNA-dependent RNA polymerase, partial [Totiviridae sp.]           1          1          Totiviridae sp. viruses         1955172    38.9   38.9  38%   4e-04  18.69  300        BDG62461.1
MAG: RNA-dependent RNA polymerase [Totiviridae sp.]               1          1          Totiviridae sp. viruses         1955172    35.4   35.4  21%   0.006  26.18  1316       QKN88741.1


Alignments:

>MAG: putative RNA-dependent RNA polymerase [Totiviridae sp.]
Sequence ID: UHS72541.1 Length: 833
Range 1: 10 to 833

Score:1061 bits(2744), Expect:0.0,
Method:Compositional matrix adjust.,
Identities:494/825(60%), Positives:628/825(76%), Gaps:1/825(0%)

Query  14   QELTKGIYNASTLTPKIVDQIIKDEGSGTDKTKYVNVPKIITDKELQTFYVPRSNADLVI  73
            QEL+ G+ N  TLT K V  I K      D++KYV +P   TD +LQ  + PR+  DL +
Sbjct  10   QELSDGVINRKTLTWKSVIHIAKQSVGQNDQSKYVEIPSHTTDIQLQEIFKPRAKVDLSL  69

Query  74   RRIRLIDLWRNLKPDQMDEIRNYTHLDYIFVQNICIYMLVFGINTVKHFRQIGLFNERNE  133
            RR R++DL R+   +Q  E+  YTHLDYIFVQNI +YM+VFG+ T++  R  G+FN R
Sbjct  70   RRFRMVDLLRHSTYEQRLELGRYTHLDYIFVQNIFVYMIVFGVPTLRSLRATGMFNNREL  129

Query  134  FIEIAKQLSTKGKRFVDDVDNMKQKVCEIATIVGYMDPNVDKIDVMEEVNSLAAEGNEHG  193
            F+  AK+LST  KR+VDD DN KQKVCE+ATI+GYM+P +D+++V+EEV+SLAA GN HG
Sbjct  130  FLTAAKELSTLAKRYVDDRDNAKQKVCELATIIGYMNPAIDELNVLEEVHSLAAPGNPHG  189

Query  194  IDRDNWNDLFTKTCKEVMTWYKGHEFISFDDYIKEGMWLTSGSSSIGKVHWTKDGENGKF  253
            ++R  W + F    +E+M +   H+FI F+ Y+KEG WLTSGSSSIG V W  + E G F
Sbjct  190  VNRHCWQNEFDAQVREIMEYTPIHDFIPFELYVKEGKWLTSGSSSIGTVEWFANDEKGHF  249

Query  254  KARKNMLLQIYTPQELANIVYAWDGKLHSRVFIKNEMSKLRLAVASNIEAYIHESYMLFL  313
            KARKNML Q+YTP ++ N+   WDGKL SRVFIKNEMSKLRLAVASNIEAYIHESYML+L
Sbjct  250  KARKNMLTQLYTPDDIYNMCVHWDGKLKSRVFIKNEMSKLRLAVASNIEAYIHESYMLYL  309

Query  314  YGHGFKEYFGVTLDEKPDQQHQREIEMIEKLQAGYFGLPFDYASFDHQPTTFEVKTMVRR  373
            YGHGFK YFG+TLDE P  QH REIEM++ LQ G +G PFDY SFDHQPTT E++++++R
Sbjct  310  YGHGFKNYFGITLDESPTAQHIREIEMVKMLQDGAYGFPFDYKSFDHQPTTSEIQSIIKR  369

Query  374  VGEIVVSQVPKNYYYQTQLLVNKIVNAYDKSYLSGNIKNTKFENIKVKGGVPSGVRITSL  433
            V  I++ Q+P  YY +   L  +I+++Y  SYLSG I +  F +++VKGGVPSGVR TSL
Sbjct  370  VISIIMPQIPSRYYTECNHLFTRILDSYTNSYLSGEINDITFRDVRVKGGVPSGVRSTSL  429

Query  434  LGNMWNAIITKIAINNVIGIIGYDPISQISLRGDDVAILSKDPAALYLLRLSYAAINAIG  493
            +GN+WN+ +T  AI      IG  P   ISLRGDDVAILS +P ALY LR++YAAINAIG
Sbjct  430  IGNLWNSCVTHKAIEITESQIGQKPFKHISLRGDDVAILSHNPMALYALRVNYAAINAIG  489

Query  494  KDSKLGISPKVCEFLRNEISVTGVRGWTCRGIGGISQRKPWNPQPWSPNDEVETNASNIS  553
             DSKLGISPK+CEFLRNEI   G+RGWTCRGIGG+SQRKPWNP PWSPN EV TNA+NI
Sbjct  490  LDSKLGISPKICEFLRNEIRTDGIRGWTCRGIGGLSQRKPWNPSPWSPNAEVTTNAANIG  549

Query  554  LLERRAGIELQQLHHINKVKWSRHVRQSYKYLELPKRLGGFGIYRFQGWLPNGKLPLAKK  613
            LLERR G ++ QLH INK+KWS+H+RQSYK+LELP RLGGFG+Y FQGWLPN KLPLAKK
Sbjct  550  LLERRVGYQIPQLHQINKIKWSKHMRQSYKWLELPIRLGGFGLYPFQGWLPNCKLPLAKK  609

Query  614  PLVNVEDIHPSQELFLPLSEQQKKILAQVEMTNKMQTDDIPGTQKLFSKEWIQKVRAKKI  673
            PLV+V+++HPS   F+ L++QQ  +LA++EMTNKMQTDDIPGTQKLFSKEWI +V+A K
Sbjct  610  PLVDVQNVHPSFTPFITLNQQQTALLAKIEMTNKMQTDDIPGTQKLFSKEWINQVKATKC  669

Query  674  IWSRNQTIPIHTDHTVRIPKWDEKIKFPRYKSEYILNNKINLTMEQVLRQYNLLKEVERH  733
            +W++ Q         + IP+WD   +FP Y  +YI +++INLT EQ+LRQYNLLKEV+R+
Sbjct  670  LWNKTQIFFGRRKPPI-IPEWDCTKQFPSYGGKYITSSRINLTFEQLLRQYNLLKEVKRY  728

Query  734  DKDLKVPKLLDILDKWFPVQSSKIKTYESQGFHRTDAINLAVGEIPTEPAVKINPILINF  793
            DK  ++PKL+D+L  +FP   S+I+ +E QGFHRTDAINLA+GEIP+EP +KINPIL  F
Sbjct  729  DKSFELPKLMDVLQNYFPAVESEIRQFEKQGFHRTDAINLAIGEIPSEPTIKINPILTRF  788

Query  794  VKLHLERQGITHQRGRNKIAKFIYQKTKQAENMILQSSLQQMYRY  838
            VK H+++ G+  QRGR  IA F+Y ++  A+  +L+S+L QMYRY
Sbjct  789  VKQHIDKAGVKFQRGRRSIAYFLYHQSASAQAALLKSTLNQMYRY  833


>MAG: putative RNA-dependent RNA polymerase, partial [Totiviridae sp.]
Sequence ID: UHS72568.1 Length: 534
Range 1: 5 to 534

Score:782 bits(2019), Expect:0.0,
Method:Compositional matrix adjust.,
Identities:370/530(70%), Positives:440/530(83%), Gaps:0/530(0%)

Query  29   KIVDQIIKDEGSGTDKTKYVNVPKIITDKELQTFYVPRSNADLVIRRIRLIDLWRNLKPD  88
            ++V +II  E    + T +  VPK ITDKELQT+YVPRSN DLV+RRIRLID+WR    +
Sbjct  5    QMVKEIIIKERESPNNTSFKYVPKNITDKELQTYYVPRSNGDLVLRRIRLIDIWRFSTEE  64

Query  89   QMDEIRNYTHLDYIFVQNICIYMLVFGINTVKHFRQIGLFNERNEFIEIAKQLSTKGKRF  148
            Q  E+  YTHLDYIF QNI IYM+VFG  TVK  R  G+FN R++FI+I+K+LSTKGKRF
Sbjct  65   QRQEMMYYTHLDYIFAQNIFIYMIVFGTETVKMLRNWGIFNNRSDFIKISKELSTKGKRF  124

Query  149  VDDVDNMKQKVCEIATIVGYMDPNVDKIDVMEEVNSLAAEGNEHGIDRDNWNDLFTKTCK  208
            + D+DN KQK+CE+ATIVGYMDP VD+ID+MEEV SLA EGN+HGI++  WN LF +T +
Sbjct  125  IKDIDNWKQKICELATIVGYMDPAVDEIDIMEEVYSLAEEGNDHGINKTKWNKLFEQTVE  184

Query  209  EVMTWYKGHEFISFDDYIKEGMWLTSGSSSIGKVHWTKDGENGKFKARKNMLLQIYTPQE  268
             VMTWYKGHEF SF++Y+KEGMWLTSGSSSIGKV W+ D E G FKARKNML+QIYTP+E
Sbjct  185  NVMTWYKGHEFKSFEEYVKEGMWLTSGSSSIGKVEWSMDEEKGHFKARKNMLMQIYTPEE  244

Query  269  LANIVYAWDGKLHSRVFIKNEMSKLRLAVASNIEAYIHESYMLFLYGHGFKEYFGVTLDE  328
            + +I   WDG+L SRVFIKNEMSKLRLAVASNIEAYI+ESY+LFLYGHGFK YFGVTLDE
Sbjct  245  IYDIARKWDGELKSRVFIKNEMSKLRLAVASNIEAYINESYILFLYGHGFKHYFGVTLDE  304

Query  329  KPDQQHQREIEMIEKLQAGYFGLPFDYASFDHQPTTFEVKTMVRRVGEIVVSQVPKNYYY  388
             P +QH RE+E+I+ LQ G++G PFDYASFDHQPTTFEV+T+V+R+ +IV+SQVP    Y
Sbjct  305  SPTEQHLREVEIIKLLQNGHYGFPFDYASFDHQPTTFEVQTIVKRMTDIVLSQVPVIAKY  364

Query  389  QTQLLVNKIVNAYDKSYLSGNIKNTKFENIKVKGGVPSGVRITSLLGNMWNAIITKIAIN  448
            +T  + N I+N Y KSYLSGNI   +F+ IKV+GGVPSGVRITS LGNMWN++ITKIA +
Sbjct  365  ETSRICNLIINGYSKSYLSGNINGVEFDRIKVRGGVPSGVRITSFLGNMWNSVITKIATD  424

Query  449  NVIGIIGYDPISQISLRGDDVAILSKDPAALYLLRLSYAAINAIGKDSKLGISPKVCEFL  508
            NV  ++G +P S ISLRGDDVAILS + +ALYLLR+SYAAINAIGKD+KLGISPKV EFL
Sbjct  425  NVKSMLGINPFSHISLRGDDVAILSNNWSALYLLRVSYAAINAIGKDAKLGISPKVIEFL  484

Query  509  RNEISVTGVRGWTCRGIGGISQRKPWNPQPWSPNDEVETNASNISLLERR  558
            RNEIS  G+RGWTCRGIGG+SQRKPWNPQPWSPN EVET  +NI LLERR
Sbjct  485  RNEISTEGIRGWTCRGIGGVSQRKPWNPQPWSPNAEVETICNNIHLLERR  534


>MAG: putative RNA-dependent RNA polymerase [Totiviridae sp.]
Sequence ID: UHS72444.1 Length: 911
Range 1: 112 to 911

Score:702 bits(1811), Expect:0.0,
Method:Compositional matrix adjust.,
Identities:360/803(45%), Positives:520/803(64%), Gaps:5/803(0%)

Query  38   EGSGTDKTKYVNVPKIITDKELQTFYVPRSNADLVIRRIRLIDLWRNLKPDQMDEIRNYT  97
            E  G   T    +     D ++Q  + PR   D+ +RR+R+ DL R    D   E+  YT
Sbjct  112  ENVGEKDTTKFRLVDFTHDSKIQEVFKPRVKLDMSVRRMRMRDLVRVANDDLKYEMGYYT  171

Query  98   HLDYIFVQNICIYMLVFGINTVKHFRQIGLFNERNEFIEIAKQLSTKGKRFVDDVDNMKQ  157
            HLDYIF  NI +Y L+ G++ ++  RQ GLFN+ + F++ A ++ST  KR + D+ N+KQ
Sbjct  172  HLDYIFASNIMLYGLIHGVDVLRELRQKGLFNDFDTFVDKASRISTYMKRLMIDLGNVKQ  231

Query  158  KVCEIATIVGYMDPNVDKIDVMEEVNSLAAEGNEHGIDRDNWNDLFTKTCKEVMTWYKGH  217
            ++ EI +++GY+  +    + +EE+  LA  G +HG+  DNW+ +F +  K++ T  K
Sbjct  232  RLVEIGSLIGYLQNDTVNWNTVEELKGLAEGGKQHGLITDNWDLVFVEYMKKIQTPSKRV  291

Query  218  EFISFDDYIKEGMWLTSGSSSIGKVHWTKDGENGKFKARKNMLLQIYTPQELANIVYAWD  277
            EF++F+DY+K   W+TSGSSSIGKV W+ D E GKFKARKNM+L +YT  EL +IV +W
Sbjct  292  EFVTFEDYVKSDKWITSGSSSIGKVEWSYDDEKGKFKARKNMILDVYTGDELWDIVRSWT  351

Query  278  GKLHSRVFIKNEMSKLRLAVASNIEAYIHESYMLFLYGHGFKEYFGVTLDEKPDQQHQRE  337
            G L S+ FIK+E+ K RLAVAS IE Y++ESY+L+LYGHGFK + G+TLDE P  QH R
Sbjct  352  GYLESKAFIKDEVGKRRLAVASGIEPYLYESYILYLYGHGFKNWEGITLDESPVSQHNRV  411

Query  338  IEMIEKLQAGYFGLPFDYASFDHQPTTFEVKTMVRRVGEIVVSQVPKNYYYQTQLLVNKI  397
              ++E L++G + LPFD++ FDHQPTT E++TM+  + E   + VP  Y  + + +V+KI
Sbjct  412  SGVVESLKSGAYALPFDFSRFDHQPTTKEIQTMILNLAE--QNDVPIGYAQEYKKIVHKI  469

Query  398  VNAYDKSYLSGNIKNTKFENIKVKGGVPSGVRITSLLGNMWNAIITKIAINNVIGIIGYD  457
            V AY  S +   I + +    KV GG+PSGVR TSL+GN WN+I+T+ A + VI ++GYD
Sbjct  470  VGAYANSEIIMKIDD-RLVKEKVIGGIPSGVRSTSLIGNEWNSIMTRRAQDIVIQLLGYD  528

Query  458  PISQISLRGDDVAILSKDPAALYLLRLSYAAINAIGKDSKLGISPKVCEFLRNEISVTGV  517
            PI +  ++GDD  +L+     LYL RL+YA+INAIG ++K GIS + CEFLRNEISV GV
Sbjct  529  PILRKGIKGDDTYLLANTAVELYLFRLAYASINAIGVNAKFGISQRNCEFLRNEISVNGV  588

Query  518  RGWTCRGIGGISQRKPWNPQPWSPNDEVETNASNISLLERRAGIELQQLHHINKVKWSRH  577
            RGW+ R +  I QRKPWNPQPWSPN EV T A+NI LLERR   +L ++H  NK++W+
Sbjct  589  RGWSNRTLPTIIQRKPWNPQPWSPNAEVATMANNIYLLERRLDFKLPKIHLANKMRWAYF  648

Query  578  VRQSYKYLELPKRLGGFGIYRFQGWLPNGKLPLAKKPLVNVEDIHPSQEL-FLPLSEQQK  636
             RQSYK+LELP RLGGFG+YR+ GW  N KLPL  KP+++VE++     L ++ LSEQQ
Sbjct  649  TRQSYKWLELPVRLGGFGLYRYDGWEVNCKLPLTTKPVISVENLTKQSSLSWIDLSEQQL  708

Query  637  KILAQVEMTNKMQTDDIPGTQKLFSKEWIQKVRAKKIIWSRNQTIPIHTDHTVRIPKWDE  696
            +    V  TNK+ T DI G QKLF +E++ K+R  + +W +           +  P
Sbjct  709  ESYHLVSFTNKISTSDIVGPQKLFGREFLLKLRKLQPVWKKEDRKVQLLKPFLNDPGLTS  768

Query  697  KIKFPRYKSEYI-LNNKINLTMEQVLRQYNLLKEVERHDKDLKVPKLLDILDKWFPVQSS  755
              ++P+   +YI  NN      ++ +R+Y+LLK+  ++DK +K+  L + L   +P   +
Sbjct  769  NARWPKIFPKYIPSNNPKYPDFQKWIREYDLLKKASQYDKSIKILSLKEYLQVAYPSILT  828

Query  756  KIKTYESQGFHRTDAINLAVGEIPTEPAVKINPILINFVKLHLERQGITHQRGRNKIAKF  815
             +  YES G+HRTDAI LA G+ PTEP  +++PIL  FVK  ++  G+ + +GR  IA
Sbjct  829  SLIKYESNGWHRTDAITLATGDYPTEPVKELHPILTPFVKEAVKNAGVEYWKGRKNIAMK  888

Query  816  IYQKTKQAENMILQSSLQQMYRY  838
            +Y  TK     ++QS+  ++Y Y
Sbjct  889  LYSVTKAQVTALMQSTTSRLYMY  911


>MAG: putative RNA-dependent RNA polymerase [Totiviridae sp.]
Sequence ID: UHS72493.1 Length: 760
Range 1: 13 to 760

Score:641 bits(1653), Expect:0.0,
Method:Compositional matrix adjust.,
Identities:333/756(44%), Positives:483/756(63%), Gaps:15/756(1%)

Query  90   MDEIRNYTHLDYIFVQNICIYMLVFGINTVKHFRQIGLFNERNEFIEIAKQLSTKGKRFV  149
            +DE+  YTHLDYIFV NI +Y ++ G+   +  R  G+FN  + FIE+A ++S+  KR
Sbjct  13   LDEMMYYTHLDYIFVSNIMLYTIIHGVEITQQLRSFGMFNSFDIFIELAGEVSSYFKRSP  72

Query  150  DDVDNMKQKVCEIATIVGYMDPNVDKIDVMEEVNSLAAEGNEHGIDRDNWNDLFTKTCKE  209
               D+ KQ++CE+A++ GY+  + +K D  EE++ LA  G EHG+   +W   +
Sbjct  73   WCTDSTKQRLCELASLTGYLQNDTEKWDTKEELSGLADGGEEHGLVGSDWLATYEHAFTS  132

Query  210  VMTWYKGHE---FISFDDYIKEGMWLTSGSSSIGKVHWTKDGENGKFKARKNMLLQIYTP  266
            +      +    F+SF+DY+K   WLTSGSSSIG V W  D E GKFKA KNM+  IYT
Sbjct  133  IQHGKYPNVYPVFMSFEDYVKSNKWLTSGSSSIGDVTWHYDQEEGKFKAHKNMIHDIYTD  192

Query  267  QELANIVYAWDGKLHSRVFIKNEMSKLRLAVASNIEAYIHESYMLFLYGHGFKEYFGVTL  326
             EL +IV  WDG LHSR FIK+E+ K RLAVASNIEAY+HESY+L+LYGH FK + G+TL
Sbjct  193  DELWDIVSNWDGVLHSRAFIKDEVGKRRLAVASNIEAYLHESYILYLYGHNFKNWDGITL  252

Query  327  DEKPDQQHQREIEMIEKLQAGYFGLPFDYASFDHQPTTFEVKTMVRRVGEIVVSQVPKNY  386
            DE P +QH+R  + I  L+ G + LPFD+  FDHQP T E++  +  +   +   +P  Y
Sbjct  253  DESPQEQHRRNSKAINLLKKGAYALPFDFKRFDHQPQTKEIQITMSNIARQI--PIPSKY  310

Query  387  YYQTQLLVNKIVNAYDKSYLSGNIKNTKFENIK--VKGGVPSGVRITSLLGNMWNAIITK  444
                  +VNK++ +YD S +S  I +   EN+K  V+GG+PSGVR TSL GN WN  ITK
Sbjct  311  LGDWDKIVNKVIQSYDNSEISMQINS---ENVKHIVEGGLPSGVRSTSLTGNGWNGAITK  367

Query  445  IAINNVIGIIGYDPISQISLRGDDVAILSKDPAALYLLRLSYAAINAIGKDSKLGISPKV  504
            IAI+    ++    + Q  ++GDD  +     +  YL+R+ YAAINA+G D+K GIS  +
Sbjct  368  IAIDTAEKVLQVLTVLQKMIKGDDTGLFVNTVSEAYLIRMCYAAINAVGSDAKFGISQNI  427

Query  505  CEFLRNEISVTGVRGWTCRGIGGISQRKPWNPQPWSPNDEVETNASNISLLERRAGIELQ  564
            CEFLRNEIS +GVRGW+ R I  ++QRKPWN +PWSPN  + T + N  LLERR  IEL
Sbjct  428  CEFLRNEISTSGVRGWSNRTIPTLTQRKPWNSEPWSPNAMISTLSQNTYLLERRLDIELP  487

Query  565  QLHHINKVKWSRHVRQSYKYLELPKRLGGFGIYRFQGWLPNGKLPLAKKPLVNVEDIHPS  624
             +H+I K KWSR++ QS ++L+LPKRLGGFG+Y + G++P+GKLPL  +PL++V+++ P
Sbjct  488  IIHNICKQKWSRYMGQSTRWLDLPKRLGGFGLYPWLGFVPDGKLPLISRPLIHVDNLKPR  547

Query  625  QEL-FLPLSEQQKKILAQVEMTNKMQTDDIPGTQKLFSKEWIQKVRAKKIIWSRNQTIPI  683
              + + PL+E Q+ ++ +V  ++K+ T DI G QK FSK+++   R +K  W++   +
Sbjct  548  SIIDWTPLNETQQYMVHKVNFSSKIATGDIVGPQKYFSKDFVTSFRKRKYGWTKEDVL-F  606

Query  684  HTDHTVRIPKWDEKIKFPRYKSEYILNNKINLTMEQV-LRQYNLLKEVERHDKDLKVPKL  742
              D T   P  DE+I +P+ +++ I +    L   QV LR+Y LL+  +R+DK +KV  L
Sbjct  607  EVDKTT-YPGVDEEIHWPKNQTKTIRSRGGKLPQLQVFLREYTLLRNAQRYDKSIKVKTL  665

Query  743  LDILDKWFPVQSSKIKTYESQGFHRTDAINLAVGEIPTEPAVKINPILINFVKLHLERQG  802
             D L ++FP    ++   E++G+HRTDAIN+ +G++PTEP  +++P+L  FVK  +  +
Sbjct  666  KDYLREYFPSSYGRLVNLEAKGWHRTDAINIILGDMPTEPIKQLHPLLSAFVKNTISDK-  724

Query  803  ITHQRGRNKIAKFIYQKTKQAENMILQSSLQQMYRY  838
                +GR  IAK +Y  T+     I  S L  MY +
Sbjct  725  FGGWKGRKTIAKHLYAWTQATIERIRSSELMTMYNW  760


>MAG: putative RNA-dependent RNA polymerase [Totiviridae sp.]
Sequence ID: UHS72478.1 Length: 807
Range 1: 29 to 807

Score:628 bits(1619), Expect:0.0,
Method:Compositional matrix adjust.,
Identities:335/794(42%), Positives:491/794(61%), Gaps:21/794(2%)

Query  51   PKIITDKELQTFYVPRSNADLVIRRIRLIDLWRNLKPDQMDEI-RNYTHLDYIFVQNICI  109
            P IIT   L    + R+  DL I R+++ D+ R +  + ++    N  HLD I + N+ I
Sbjct  29   PPIITKGNLLELPL-RTKGDLAIARLKMKDVLRKIDNEIVEYFSNNLCHLDQIMLTNLII  87

Query  110  YMLVFGINTVKHFRQIGLFNERNEFIEIAKQLSTKGKRFVDDVDNMKQKVCEIATIVGYM  169
            +  ++G+  ++     GL N    F   A +LS+  KRF  D ++ KQ+  E  T+ GYM
Sbjct  88   WTQMWGLGLLRTIHSTGLLNSFEVFATKAAKLSSYVKRFPFDKNDCKQRFSEFNTLTGYM  147

Query  170  DPNVDKIDVMEEVNSLAAEGNEHGIDRDNWNDLFTKTCKEVMTWYKGHEFISFDDYIKEG  229
              N    D   E++ LA+ GNEH    + W   F      +M      +F+SF+ Y+KEG
Sbjct  148  QTNFGNFDYDHELSELASGGNEHD---NKWTTKFNDKLITLMNVQPLPQFVSFEKYVKEG  204

Query  230  MWLTSGSSSIGKVHWTKDGENGKFKARKNMLLQIYTPQELANIVYAWDGKLHSRVFIKNE  289
            +WLTSGSSSIG+VHW+ DGE GKFKARKNMLL +YT  EL  +   WDGKL ++VFIK+E
Sbjct  205  LWLTSGSSSIGQVHWSYDGEKGKFKARKNMLLDLYTGDELYKMALEWDGKLENKVFIKDE  264

Query  290  MSKLRLAVASNIEAYIHESYMLFLYGHGFKEYFGVTLDEKPDQQHQREIEMIEKLQAGYF  349
            ++K RLAV+SNIEAY+++ Y+L L+GHGFK Y  +TLDEKP +QH R  ++I+ L+ G F
Sbjct  265  LAKRRLAVSSNIEAYLNQGYLLHLFGHGFKNYKYITLDEKPSEQHVRNCKLIKLLRNGSF  324

Query  350  GLPFDYASFDHQPTTFEVKTMVRRVGEIVVSQVPKNYYYQTQLLVNKIVNAYDKSYLSGN  409
             LP+D+  FD QP T +++ +V R+G+ V   VP  +    + ++ K    Y  +YL
Sbjct  325  ALPWDFKGFDRQPRTADIEAIVDRIGKRVEPNVPIEHRSMFKKILQKNKICYYNNYLYSP  384

Query  410  IKNTKFENIKVKGGVPSGVRITSLLGNMWNAIITKIAINNVIGIIGYDPISQISLRGDDV  469
            I     + IK  GG+PSG+R TSL+GN++NA +T +A +    IIG+D I +I LRGDD
Sbjct  385  ITK---QTIKQTGGLPSGIRPTSLIGNIYNANVTDMARDVTKEIIGFDDIQEIGLRGDDT  441

Query  470  AILSKDPAALYLLRLSYAAINAIGKDSKLGISPKVCEFLRNEISVTGVRGWTCRGIGGIS  529
             I+SK+   L + R +Y ++N  G++SK GI   VCEFLR E+S  GVRGW+ R I  ++
Sbjct  442  YIISKNWFTLLIFRYAYLSLNVTGENSKFGIMQNVCEFLRTEVSTNGVRGWSNRAIPSVT  501

Query  530  QRKPWNPQPWSPNDEVETNASNISLLERRAGIELQQLHHINKVKWSRHVRQSYKYLELPK  589
            QRKPWNPQPW+ N+EVET A+NI LLERR+ ++L  LHH NK+KWSR+  QSY +L LPK
Sbjct  502  QRKPWNPQPWTANNEVETVANNIYLLERRSKLDLDWLHHCNKLKWSRYTHQSYLWLHLPK  561

Query  590  RLGGFGIYRFQGWLPNGKLPLAKKPLVNVEDIHPSQELFLP---LSEQQKKILAQVEMTN  646
             LGG G+Y ++GW P+GKLPL++ P   +E+   +  L L    L+E++K+    V M+
Sbjct  562  HLGGIGLYEWKGWSPDGKLPLSQPPTFAIEET-TTANLSLQWHNLNEEEKQKYQAVSMSM  620

Query  647  KMQTDDIPGTQKLFSKEWIQKVRAKKIIWSRNQTIPIHTDHTVRIPKWDEKIKFPRYKSE  706
            K+  DDIPG QK  S E+++K+R  +  WS+ QTIPI   + +  P +D  I +P+ K
Sbjct  621  KIAADDIPGPQKFMSIEYVKKLRTMRFNWSK-QTIPIVKTYKIEGPTFDN-ILWPKTKLH  678

Query  707  YILN-NKINL-TMEQVLRQYNLLKEVERHDKDLKVPKLLDILDKWFPVQSSKIKTYESQG  764
              L  N+I +    + +R++ + K  +     +K+P L+++  K++P     I+ YE++G
Sbjct  679  VDLKPNRIEMPNFIEFVREHQIAKRAK-----IKIPSLMELTLKYYPSVYKDIRYYEARG  733

Query  765  FHRTDAINLAVGEIPTEPAVKINPILINFVKLHLERQGITHQRGRNKIAKFIYQKTKQAE  824
            +HRTDAINLA G +PTEP   +NP+L  F+   L + G+ H +GR  IA  +Y  T  +
Sbjct  734  WHRTDAINLASGNVPTEPTKILNPLLTIFITTMLLKSGLKHWKGRKNIAILLYTITTSSV  793

Query  825  NMILQSSLQQMYRY  838
            + I       MY +
Sbjct  794  SWIQAEGGSTMYAF  807


>MAG: putative RNA-dependent RNA polymerase, partial [Totiviridae sp.]
Sequence ID: UHS72503.1 Length: 739
Range 1: 1 to 739

Score:626 bits(1614), Expect:0.0,
Method:Compositional matrix adjust.,
Identities:327/745(44%), Positives:479/745(64%), Gaps:11/745(1%)

Query  78   LIDLWRNLKPDQMDEIRNYTHLDYIFVQNICIYMLVFGINTVKHFRQIGLFNERNEFIEI  137
            ++D+ R    +  DEI  Y+HLDYIFV NI IY ++ G++T++  R +G+FN  + FIE+
Sbjct  1    MVDILRFCPKELKDEILYYSHLDYIFVSNILIYGVIHGVDTLRQLRGLGIFNSYDHFIEV  60

Query  138  AKQLSTKGKRFVDDVDNMKQKVCEIATIVGYMDPNVDKIDVMEEVNSLAAEGNEHGIDRD  197
            A  +S+  KR     D+ KQ++CE+A+I GY+  +  + D  EE   LA  GNEHG+
Sbjct  61   AGNISSYFKRSPYCTDDTKQRLCELASISGYLQNDTSEWDTKEEFKDLAEGGNEHGLIGT  120

Query  198  NWNDLFTKTCKEVMTW---YKGHEFISFDDYIKEGMWLTSGSSSIGKVHWTKDGENGKFK  254
            NW  LF +T +++      +K  E++SF+D+I+   WLTSGSSSIG V W+ D E+  FK
Sbjct  121  NWMQLFRETFEKIQITDQPFKRPEWLSFEDFIRSNKWLTSGSSSIGGVEWSYDEEHTTFK  180

Query  255  ARKNMLLQIYTPQELANIVYAWDGKLHSRVFIKNEMSKLRLAVASNIEAYIHESYMLFLY  314
            ARKNM+  IY P EL  I+ +WDG LHS+ FIK+E+ K RLAVASNIEAY+HESY+L+LY
Sbjct  181  ARKNMIHDIYNPDELIQIIKSWDGILHSKAFIKDEVGKRRLAVASNIEAYMHESYILYLY  240

Query  315  GHGFKEYFGVTLDEKPDQQHQREIEMIEKLQAGYFGLPFDYASFDHQPTTFEVKTMVRRV  374
            GH FK +  +TLDE P  QH R +E IE L+AG + LPFD+  FDHQPTT E++ M+  +
Sbjct  241  GHNFKNWPNITLDESPVAQHYRMVECIELLRAGKYALPFDFKRFDHQPTTDEIQLMIASL  300

Query  375  GEIVVSQVPKNYYYQTQLLVNKIVNAYDKSYLSGNIKNTKFENIKVKGGVPSGVRITSLL  434
               +V  VP++Y  + + +V+ +V +Y KS LS  I + K   I VKGG+PSGVR TSL
Sbjct  301  SAKIV--VPEHYRQEWESIVSIVVRSYAKSQLSMFI-DGKMHTIDVKGGLPSGVRATSLY  357

Query  435  GNMWNAIITKIAINNVIGIIGYDPISQISLRGDDVAILSKDPAALYLLRLSYAAINAIGK  494
            GN WN  +T IAI+    ++    +  + ++GDD  I +   A  Y++RL YAAINAIG
Sbjct  358  GNGWNDDMTTIAIDLAKVVLQALQVLLVMIKGDDTGIFTNTIAEAYIIRLCYAAINAIGS  417

Query  495  DSKLGISPKVCEFLRNEISVTGVRGWTCRGIGGISQRKPWNPQPWSPNDEVETNASNISL  554
            DSK GIS  +CEFLRNEIS TGVRGW+ R I  ++QRKPWN +PWSPN  + T + N+ L
Sbjct  418  DSKFGISQNICEFLRNEISSTGVRGWSNRTIPTLTQRKPWNSEPWSPNALISTLSQNVYL  477

Query  555  LERRAGIELQQLHHINKVKWSRHVRQSYKYLELPKRLGGFGIYRFQGWLPNGKLPLAKKP  614
            LERR   E+  +H + +VKWS+++ QS  +L+LPKRLGGFG+Y ++G +P+GKLPL  +P
Sbjct  478  LERRLSREVSSVHQMCRVKWSKYMGQSTNWLDLPKRLGGFGLYVWRGLIPDGKLPLISRP  537

Query  615  LVNVED-IHPSQELFLPLSEQQKKILAQVEMTNKMQTDDIPGTQKLFSKEWIQKVRAKKI  673
            L+++ + ++ +   ++  +  Q+K++ +V    K+   DI G QK +SK+++ K R  K
Sbjct  538  LIHIHNLVNRTIVGWVKATPNQEKVIHEVNFNAKIAAGDILGPQKYYSKDFVTKFRKNKY  597

Query  674  IWSRNQTIPIHTDHTVRIPKWDEKIKFPRYKSEYILNNKINLTMEQV-LRQYNLLKEVER  732
             W R  ++ I  D T       +  ++P   ++ I +    L   QV LR+Y L+K  +R
Sbjct  598  NW-RKISVDIEID-TTSYNAMSQTFRWPTSTTKTIRSVANQLPQLQVFLREYTLVKNAKR  655

Query  733  HDKDLKVPKLLDILDKWFPVQSSKIKTYESQGFHRTDAINLAVGEIPTEPAVKINPILIN  792
            +DK +++  L   L  ++P    ++   E +G+HRTDA+N+ +G+ PTEP  +I+PIL
Sbjct  656  YDKTIELAPLKQYLKDYYPSSYQQLLILEKKGWHRTDALNIIMGDYPTEPIKQIHPILAP  715

Query  793  FVKLHLERQGITHQRGRNKIAKFIY  817
            FVK  + R+     +GR  I K +Y
Sbjct  716  FVKDRISRE-FGKWKGRINIGKKLY  739


>MAG: putative RNA-dependent RNA polymerase, partial [Totiviridae sp.]
Sequence ID: UHS72552.1 Length: 851
Range 1: 87 to 851

Score:622 bits(1604), Expect:0.0,
Method:Compositional matrix adjust.,
Identities:330/780(42%), Positives:484/780(62%), Gaps:22/780(2%)

Query  66   RSNADLVIRRIRLIDLWRNLKPDQMDEIRN-YTHLDYIFVQNICIYMLVFGINTVKHFRQ  124
            R+  D+ I R+++ D+ R +  D ++   N   HLD I + N+ I+  ++G++ +K
Sbjct  87   RTKGDIAIARLKMKDILRKIDKDIINYFENNLCHLDQIMLTNLLIWTKMWGLDILKAIHM  146

Query  125  IGLFNERNEFIEIAKQLSTKGKRFVDDVDNMKQKVCEIATIVGYMDPNVDKIDVMEEVNS  184
             G+ N    F   A +LS+  KRF  D D+ KQ+  E  T+ GYM  +    D  +E +
Sbjct  147  TGMLNSFEIFAVKAAKLSSYVKRFPFDRDDCKQRFSEFNTLTGYMQNDFGNFDYDKEFDE  206

Query  185  LAAEGNEHGIDRDNWNDLFTKTCKEVMTWYKGHEFISFDDYIKEGMWLTSGSSSIGKVHW  244
            LA  GNEH    + W   F     E+M       FI+F+DY+K+G+WLTSGSSS+G+VHW
Sbjct  207  LAHGGNEHD---EEWTTKFEAKLDELMNVQSLPTFITFEDYVKKGLWLTSGSSSLGEVHW  263

Query  245  TKDGENGKFKARKNMLLQIYTPQELANIVYAWDGKLHSRVFIKNEMSKLRLAVASNIEAY  304
            + D E GKFKARKNMLL +YT  EL +I   WDGKL+++VF+K+E++K RLAV+SNIEAY
Sbjct  264  SYDNEKGKFKARKNMLLDLYTADELYDIALNWDGKLYNKVFVKDELAKRRLAVSSNIEAY  323

Query  305  IHESYMLFLYGHGFKEYFGVTLDEKPDQQHQREIEMIEKLQAGYFGLPFDYASFDHQPTT  364
            +++ Y+L+L+GHGFK Y  VTLDEKP +QH R  ++I+ L+ G F LP+D+  FD QP
Sbjct  324  LNQGYLLYLFGHGFKNYKYVTLDEKPCEQHARNAKLIKLLKNGSFALPWDFKGFDRQPRL  383

Query  365  FEVKTMVRRVGEIVVSQVPKNY--YYQTQLLVNKIVNAYDKSYLSGNIKNTKFENIKVKG  422
             +++ +V R+G+ V   VP  +   ++  LL NK    Y K+YL   I     + +   G
Sbjct  384  RDIEAIVERIGKRVEPNVPSEHKSLFRQILLKNK--TCYFKNYLYSPITK---QTVLQTG  438

Query  423  GVPSGVRITSLLGNMWNAIITKIAINNVIGIIGYDPISQISLRGDDVAILSKDPAALYLL  482
            G+PSG+R TSL+GN++NA +T +A +    IIG+D I +ISLRGDD  I+SK+   L +
Sbjct  439  GLPSGIRPTSLIGNIYNANVTDMARDVTKSIIGFDDIQEISLRGDDTYIISKNWFTLLIF  498

Query  483  RLSYAAINAIGKDSKLGISPKVCEFLRNEISVTGVRGWTCRGIGGISQRKPWNPQPWSPN  542
            R +Y ++N  G++SK GI    CEFLR EIS +GVRGW+ R I  ++QRKPWNPQPW+ N
Sbjct  499  RYAYQSLNITGENSKFGIMQNACEFLRTEISTSGVRGWSNRAIPSVTQRKPWNPQPWTAN  558

Query  543  DEVETNASNISLLERRAGIELQQLHHINKVKWSRHVRQSYKYLELPKRLGGFGIYRFQGW  602
             EVET A+NI LLERR+ ++L  LHH NK+KWS++  QSY +L LPK LGG G+Y ++GW
Sbjct  559  IEVETVANNIYLLERRSKMDLDWLHHCNKIKWSKYTHQSYLWLHLPKHLGGIGLYEWKGW  618

Query  603  LPNGKLPLAKKPLVNVEDIHPS--QELFLPLSEQQKKILAQVEMTNKMQTDDIPGTQKLF  660
             P+GKLPL++ P   ++D   +     +  L+EQ+K+    V M+ K+  DDIPG QK
Sbjct  619  SPDGKLPLSQPPTFTIQDTTTANINLDWYQLNEQEKQDYQAVSMSMKIAADDIPGPQKYM  678

Query  661  SKEWIQKVRAKKIIWSRNQTIPIHTDHTVRIPKWDEKIKFPRYKSEYILN-NKINL-TME  718
            S  ++ K+R  +  WS+ + IPI   + +  P ++  + +PR K    L  NKI +
Sbjct  679  SIGYVNKLRNMRFTWSKIE-IPIAKVYKIEGPHFENNL-WPRNKHFVDLKPNKITMPNYI  736

Query  719  QVLRQYNLLKEVERHDKDLKVPKLLDILDKWFPVQSSKIKTYESQGFHRTDAINLAVGEI  778
            + +R++ + K  +     +K+P L+++  K++P     I+ YES G+HRTDAINLA G I
Sbjct  737  EFVREHQIAKRAK-----IKIPSLMELTLKYYPSVYKDIRQYESNGWHRTDAINLATGGI  791

Query  779  PTEPAVKINPILINFVKLHLERQGITHQRGRNKIAKFIYQKTKQAENMILQSSLQQMYRY  838
            PTEP   +NP+L  F+   LE+ G+   +GR  IA  +Y  T  + N I       MY +
Sbjct  792  PTEPTKILNPLLTVFITTMLEKSGLRFWKGRTNIAILLYITTTASVNWIQAEGGSTMYAF  851


>MAG: putative RNA-dependent RNA polymerase [Totiviridae sp.]
Sequence ID: UHS72448.1 Length: 799
Range 1: 17 to 793

Score:603 bits(1555), Expect:0.0,
Method:Compositional matrix adjust.,
Identities:329/787(42%), Positives:477/787(60%), Gaps:17/787(2%)

Query  52   KIITDKELQTFYVPRSNADLVIRRIRLIDLWRNLKPDQMDEIRNY-THLDYIFVQNICIY  110
            KI   +E+ +    R   DL IRR+R  D+WR L  D++ E+  Y THLD I   NI ++
Sbjct  17   KINVTEEILSDLSARCKGDLAIRRLRAKDIWRTLD-DKVKEVMGYLTHLDTIMFTNIAVW  75

Query  111  MLVFGINTVKHFRQIGLFNERNEFIEIAKQLSTKGKRFVDDVDNMKQKVCEIATIVGYMD  170
              +FG + +    +  +F++ + F E A ++ST  KRF    D+ K  + E+  + GY+
Sbjct  76   GSIFGADKIMDLIKRNVFDDFDTFREAASKISTYIKRFPYCNDDTKIALGELNCLTGYLQ  135

Query  171  PNVDKIDVMEEVNSLAAEGNEHGIDRDNWNDLFTKTCKEVMTWYKGHEFISFDDYIKEGM  230
             N    D+ EE   LA  GNEHG+   +WN +F    + + T  +   F++  +YI  G
Sbjct  136  TNPRGFDLEEEFYKLANAGNEHGLVGSDWNRIFDDKVRSLFTREELPPFMTLREYIDSGK  195

Query  231  WLTSGSSSIGKVHWTKDGENGKFKARKNMLLQIYTPQELANIVYAWDGKLHSRVFIKNEM  290
            WLT+GSSSIGKV W+   E GKFKARKNML++++T  E+ N+   WDGK+ ++VF K+E+
Sbjct  196  WLTAGSSSIGKVEWSMADEKGKFKARKNMLVELFTADEIYNMANEWDGKIINKVFTKDEL  255

Query  291  SKLRLAVASNIEAYIHESYMLFLYGHGFKEYFGVTLDEKPDQQHQREIEMIEKLQAGYFG  350
             K RLAVASNIE+Y+ ESY+ +L GHGFK +  +TLDE P  QH R  + ++ L  G F
Sbjct  256  GKRRLAVASNIESYLVESYLSYLGGHGFKNWKYMTLDETPAAQHNRMRKAMKLLTDGSFA  315

Query  351  LPFDYASFDHQPTTFEVKTMVRRVGEIVVSQVPKNYYYQTQL--LVNKIVNAYDKSYLSG  408
            LP+D+A+FDHQ +  EVK+++   G +V+  VP  +YYQ++   L+NK + +YD + L
Sbjct  316  LPWDFAAFDHQSSISEVKSIISMSGSLVMDSVP--FYYQSEYRKLLNKSIASYDNNILLM  373

Query  409  NIKNTKFENIKVKGGVPSGVRITSLLGNMWNAIITKIAINNVIGIIGYDPISQISLRGDD  468
               +   E  +  GG+PSG+R TS +GN WN ++T I  + V  I+G D I  I ++GDD
Sbjct  374  KTNDRTLE-ARQTGGIPSGIRRTSYIGNCWNMVVTDIVRDYVKAIVGQDTIQTIRIKGDD  432

Query  469  VAILSKDPAALYLLRLSYAAINAIGKDSKLGISPKVCEFLRNEISVTGVRGWTCRGIGGI  528
              I+++DP  LY+ R  YAA+NAIG +SK GI+  +CEFLR E+     +GW  R I  I
Sbjct  433  TYIIAEDPVVLYIFRAVYAAVNAIGINSKFGIARNICEFLRVEMRPDACQGWVNRAIPSI  492

Query  529  SQRKPWNPQPWSPNDEVETNASNISLLERRAGIELQQLHHINKVKWSRHVRQSYKYLELP  588
            +QRKPWN QPWSP+ EV T   NI LLERRA   +  LH  NK+KWS++  QS  +L+LP
Sbjct  493  TQRKPWNSQPWSPSAEVGTVCKNIELLERRAMRLIPSLHRANKIKWSKYTSQSTHWLDLP  552

Query  589  KRLGGFGIYRFQGWLPNGKLPLAKKPLVNVED-IHPSQELFLPLSEQQKKILAQVEMTNK  647
             RLGGFG+YRFQGW+PN  LP   KPLV +E+ + P++  +  L+  + +   +    +K
Sbjct  553  TRLGGFGLYRFQGWVPNDTLPRISKPLVKIENPLVPAKLQWTNLTHMEAEAYTRESFNDK  612

Query  648  MQTDDIPGTQKLFSKEWIQKVRAKKIIWSRNQTIPIHTDHTVRIPKWDEKI--KFPRYKS  705
            + +DDIPG QK + K++I  +R +K  W R +T      HTV  P  D+ I  KF R  +
Sbjct  613  LASDDIPGPQKYYGKKFINDLRNRKYDW-RKETFMTGRYHTVSGPV-DDAIWPKFKRVAT  670

Query  706  EYILNNKINLTMEQVLRQYNLLKE-VERHDKDLKVPKLLDILDKWFPVQSSKIKTYESQG  764
            + +  N    T  + +R++  LK  +ER   D+K+  L+D   +++P    +IK YE  G
Sbjct  671  DSLSANFPRYT--EFVREHQTLKRALERL--DIKIRSLMDYTQQYYPGIYEQIKMYERNG  726

Query  765  FHRTDAINLAVGEIPTEPAVKINPILINFVKLHLERQGITHQRGRNKIAKFIYQKTKQAE  824
            +HRTDAI++AVGEIP EP   ++PIL  FVK  +E       +GR  IA  +Y  T QA
Sbjct  727  WHRTDAISIAVGEIPCEPTKILHPILTPFVKKAVEMNDFKFWKGRKNIAIRLYNITMQAV  786

Query  825  NMILQSS  831
              I  +S
Sbjct  787  EKIQLTS  793


>MAG: putative RNA-dependent RNA polymerase, partial [Totiviridae sp.]
Sequence ID: UHS72466.1 Length: 780
Range 1: 59 to 780

Score:598 bits(1542), Expect:0.0,
Method:Compositional matrix adjust.,
Identities:313/728(43%), Positives:449/728(61%), Gaps:20/728(2%)

Query  54   ITDKELQTFYVPRSNADLVIRRIRLIDLWRNLKPDQMDEIRNYTHLDYIFVQNICIYMLV  113
            +TD EL      RS  D  IRR +  D+W +   +   E+R Y HLD IF  NI I+  +
Sbjct  59   LTDAELLKRMPVRSRLDSSIRRFKWRDIWTSAPAELKHEMRYYQHLDLIFSTNIMIWGRL  118

Query  114  FGINTVKHFRQIGLFNERNEFIEIAKQLSTKGKRFVDDVDNMKQKVCEIATIVGYMDPNV  173
            FG++ ++  R +GLFN  + F++ A ++S   KRF  D D++KQK+ E++ +VGY+  +
Sbjct  119  FGVDYLRDLRLLGLFNGLDHFLDRASKISAYVKRFPYDPDDVKQKLAELSGLVGYLTTDT  178

Query  174  DKIDVMEEVNSLAAEGNEHGIDRDNWNDLFTKTCKEVMTWYKGHEFISFDDYIKEGMWLT  233
            +  +   E+ +LA  GN+HG+  D+W  LF +  + +       +FI+F++Y+K G   T
Sbjct  179  ESWNTAGELAALAEGGNQHGLITDDWRSLFPQLAELIKNGRSAPKFITFEEYVKSGQXTT  238

Query  234  SGSSSIGKVHWTKDGENGKFKARKNMLLQIYTPQELANIVYAWDGKLHSRVFIKNEMSKL  293
            SGS+SIGKV WT D E+G FKA+KNML +IYTP EL  +  AWDG L SR FIK+E  K
Sbjct  239  SGSASIGKVVWTHDLEHGSFKAKKNMLFEIYTPDELWALALAWDGTLRSRAFIKSEAGKR  298

Query  294  RLAVASNIEAYIHESYMLFLYGHGFKEYFGVTLDEKPDQQHQREIEMIEKLQAGYFGLPF  353
            RLAV+SN+E+Y+HESY+L LYGH FK +  +TLDE    QH R  + ++ L+AG + LPF
Sbjct  299  RLAVSSNLESYLHESYILRLYGHSFKNWDYITLDESAATQHNRSAKTVQLLKAGAWALPF  358

Query  354  DYASFDHQPTTFEVKTMVRRVGEIVVSQVPKNYYYQTQLLVNKIVNAYDKSYLSGNIKNT  413
            D+A FDHQPTT+E++TMV  + + V   VP  Y  Q  ++  K+V +Y +S +S  I
Sbjct  359  DFARFDHQPTTWEIQTMVSSIIKEV--SVPTTYAEQFSIIAAKVVGSYARSSISMVIDGK  416

Query  414  KFENIKVKGGVPSGVRITSLLGNMWNAIITKIAINNVIGIIGYDPISQISLRGDDVAILS  473
            +  +  V GG+PSGVRITSL+GN WN+I+T  A      ++GYDP+    ++GDD AI
Sbjct  417  EHVHT-VTGGIPSGVRITSLIGNQWNSIMTSRARALATSVLGYDPVLFGQIKGDDTAIFC  475

Query  474  KDPAALYLLRLSYAAINAIGKDSKLGISPKVCEFLRNEISVTGVRGWTCRGIGGISQRKP  533
            +     ++ RLSY AINAIG DSK GIS  VCEFLRNE S +G+RGW  R I  ++QRKP
Sbjct  476  RTAVECWVFRLSYQAINAIGLDSKFGISQNVCEFLRNEASPSGLRGWANRTIPTLTQRKP  535

Query  534  WNPQPWSPNDEVETNASNISLLERRAGIELQQLHHINKVKWSRHVRQSYKYLELPKRLGG  593
            W  QP SP+ +  T ASNI  LERR G+EL   H  N +KWSR +RQSY +L LP RLGG
Sbjct  536  WTAQPLSPSRDTATLASNIRTLERRLGVELPWAHRANAIKWSRLMRQSYFWLHLPIRLGG  595

Query  594  FGIYRFQGWLPNGKLPLAKKPLVNVE-----DIHPSQELFLPLSEQQKKILAQVEMTNKM  648
             G+Y F GW+P+ KLPL  KP+++ +          Q L LP S + K +  ++ M+ KM
Sbjct  596  LGVYSFNGWVPSRKLPLVTKPIIDTDVRTAVGTDGPQWLSLPNSARDKYL--KLSMSEKM  653

Query  649  QTDDIPGTQKLFSKEWIQKVRAKKIIWSRNQ-------TIPIHTDHT-VRIPKWDEKIKF  700
              DDIPG Q+ F +++I +VRA K+ WSR+        +  I  + T +  P  +    +
Sbjct  654  AFDDIPGPQQAFFRDYIDEVRALKVTWSRDSQLLKRYNSFKIAAEQTQILTPSLEPDNSW  713

Query  701  PRYKSEYILNNKINL-TMEQVLRQYNLLKEVERHDKDLKVPKLLDILDKWFPVQSSKIKT  759
            P+ +   +L++  ++    Q +R+Y  LK +        +PK +D+  K+FP  S +I+
Sbjct  714  PKLQMPNLLSSDSSVPPFPQWVREYMALKRLWEPQFG-TLPKFIDLASKYFPNISDRIQY  772

Query  760  YESQGFHR  767
            YE  G+HR
Sbjct  773  YERNGWHR  780


>MAG: putative RNA-dependent RNA polymerase, partial [Totiviridae sp.]
Sequence ID: UHS72514.1 Length: 687
Range 1: 21 to 652

Score:597 bits(1539), Expect:0.0,
Method:Compositional matrix adjust.,
Identities:302/656(46%), Positives:428/656(65%), Gaps:27/656(4%)

Query  56   DKELQTFYVPRSNADLVIRRIRLIDLWRNLKPDQMDEIRNYTHLDYIFVQNICIYMLVFG  115
            D+EL+ ++  R+  DL +RR++ +DL+R    D   EIR Y+HLD IF  N+ +Y ++ G
Sbjct  21   DRELEDYFSVRTKLDLSVRRLKFLDLYRLANSDLKSEIRYYSHLDIIFATNVLLYGMIHG  80

Query  116  INTVKHFRQIGLFNERNEFIEIAKQLSTKGKRFVDDVDNMKQKVCEIATIVGYMDPNVDK  175
            + T++  RQ+G+FN+   F+ +A  +ST  KR   D D+ KQK+CE+ +++GY+  + ++
Sbjct  81   VETLRDLRQLGVFNDFEHFVRVAGDISTYFKRVPFDEDDTKQKLCELGSLIGYLQNDTEE  140

Query  176  IDVMEEVNSLAAEGNEHGIDRDNWNDLFTKTCKEVMTWYKGH-EFISFDDYIKEGMWLTS  234
             D  EE++ LA  G++HG+  ++W   F +  +E+ +  +   +FI+F++YI    W+T
Sbjct  141  WDTEEELSGLAEGGDQHGLIDEDWMPEFRRLIREITSAQRTPPKFITFEEYITSSEWITP  200

Query  235  GSSSIGKVHWTKDGENGKFKARKNMLLQIYTPQELANIVYAWDGKLHSRVFIKNEMSKLR  294
            GSSSIGKV W+ D + GKFKARKNM+  I+T QEL +I+  WDG L SR FIK+E+ K R
Sbjct  201  GSSSIGKVEWSYDTDTGKFKARKNMIEDIFTKQELLDIILNWDGVLRSRAFIKDEVGKRR  260

Query  295  LAVASNIEAYIHESYMLFLYGHGFKEYFGVTLDEKPDQQHQREIEMIEKLQAGYFGLPFD  354
            LAVASNIEAY+HE+Y+L L+GH FK + G+TLDE P  QH R  E I +L  G + LPFD
Sbjct  261  LAVASNIEAYLHEAYLLRLFGHSFKNWDGITLDESPKAQHIRNAENIIQLSQGKYALPFD  320

Query  355  YASFDHQPTTFEVKTMVRRVGEIVVSQVPKNYYYQTQLLVNKIVNAYDKSYLSGNIKNTK  414
            +  FDHQPTT E++TMV  +   +   VP +Y +    +V+K++ +Y KS +S  I
Sbjct  321  FRRFDHQPTTLEIQTMVADLAHDIT--VPHSYQHTFNKIVSKVIESYAKSQISMQIGQKT  378

Query  415  FENIK-VKGGVPSGVRITSLLGNMWNAIITKIAINNVIGIIGYDPISQISLRGDDVAILS  473
               IK V GG+PSGVRITSL+GN WN+I+T+ A N    I+GYDP++Q  ++GDD  +L
Sbjct  379  I--IKDVTGGIPSGVRITSLIGNEWNSIMTRKAQNITTTILGYDPVTQKQIKGDDTGLLF  436

Query  474  KDPAALYLLRLSYAAINAIGKDSKLGISPKVCEFLRNEISVTGVRGWTCRGIGGISQRKP  533
            +  A  ++ RL+Y AINA+G DSK GIS  +CEFLRNEIS +GVRGW+ R I  I+QRKP
Sbjct  437  RTAAEAFIFRLAYQAINAVGLDSKFGISQNICEFLRNEISPSGVRGWSNRAIPTITQRKP  496

Query  534  WNPQPWSPNDEVETNASNISLLERRAGIELQQLHHINKVKWSRHVRQSYKYLELPKRLGG  593
            WNP+PWSP+  V T A NI LLERR   +L  +H  NKVKWS++  QSYK+LELP RLGG
Sbjct  497  WNPEPWSPSSAVGTIAQNIYLLERRLHFQLPHIHQANKVKWSKYTNQSYKWLELPVRLGG  556

Query  594  FGIYRFQGWLPNGKLPLAKKPLVNVEDIHPSQEL-FLPLSEQQKKILAQVEMTNKMQTDD  652
             GIY +QGW PN KLPL  KPL+ V+++ P     ++ L++ Q   +    +TNK+ T D
Sbjct  557  MGIYHWQGWEPNCKLPLVTKPLIKVDNLVPGFSYDWIALTQSQWASVHAQSVTNKISTSD  616

Query  653  IPGTQKLFSKEWIQKVRAKKIIWSRNQTIPIHTDHTVRIPKWDEKIKFPRYKSEYI  708
            I G QK FS+++I ++R     WS +                    K PR+ S YI
Sbjct  617  INGPQKHFSRDFINQIRKINPTWSLS--------------------KLPRFSSVYI  652


>MAG: putative RNA-dependent RNA polymerase, partial [Totiviridae sp.]
Sequence ID: UHS72496.1 Length: 800
Range 1: 132 to 800

Score:580 bits(1496), Expect:0.0,
Method:Compositional matrix adjust.,
Identities:300/676(44%), Positives:427/676(63%), Gaps:15/676(2%)

Query  64   VPRSNADLVIRRIRLIDLWRNLKPDQMDEIRNYTHLDYIFVQNICIYMLVFGINTVKHFR  123
            +PR+N DL IRR+R+ D+ R +K +++DE+R + HLD I   NIC++ +V G   ++  R
Sbjct  132  LPRTNGDLAIRRLRIKDIMRVMKKEEIDEMRFFAHLDNIMFANICVFGIVNGFGKLRDLR  191

Query  124  QIGLFNERNEFIEIAKQLSTKGKRFVDDVDNMKQKVCEIATIVGYMDPNVDKIDVMEEVN  183
            + G+FN  + F+E A ++S+  KR+   +D+ K  +CEI  + GY+  +    D   E +
Sbjct  192  ECGIFNSFDIFVEAASKISSHFKRYPICLDDTKMVLCEINCLTGYLQNDPPGWDFEREFD  251

Query  184  SLAAEGNEHGIDRDNWNDLFTKTCKEVMTWYKGHEFISFDDYIKEGMWLTSGSSSIGKVH  243
            +L   G EHG+   +W   F    K VMT    + ++S DDYI+ G+W+TSG+SSIGKV
Sbjct  252  ALGKGGYEHGLLNSDWKKDFVGALKRVMTRQSVNNWVSLDDYIRSGLWITSGASSIGKVE  311

Query  244  WTKDGENGKFKARKNMLLQIYTPQELANIVYAWDGKLHSRVFIKNEMSKLRLAVASNIEA  303
            W+  GE+GKFKARKNML ++Y+  EL +IV  WDG L SRVF K+E+SK RLAVASNIEA
Sbjct  312  WSYSGESGKFKARKNMLTELYSDDELISIVRKWDGVLRSRVFTKDELSKRRLAVASNIEA  371

Query  304  YIHESYMLFLYGHGFKEYFGVTLDEKPDQQHQREIEMIEKLQAGYFGLPFDYASFDHQPT  363
            Y+ ES++L ++GHGFK Y  +TLDE P +QHQR   ++  ++ G F LPFD+  FDHQP
Sbjct  372  YLTESWILHMFGHGFKNYDYITLDETPKEQHQRGCGVMRLIEGGAFCLPFDFKGFDHQPQ  431

Query  364  T-FEVKTMVRRVGEIVVSQVPKNYYYQTQLLVNKIVNAYDKSYLSGNIKNTK-FENIKVK  421
               EV+ ++  V E V   VP +       L +++V    KSY  G + N K   +I  +
Sbjct  432  IENEVQPILDMVVERVREIVPVDQLPDYDRLSSRMV----KSYAKGELINPKTMRSIIQE  487

Query  422  GGVPSGVRITSLLGNMWNAIITKIAINNVIGIIGYDPISQISLRGDDVAILSKDPAALYL  481
            GG+PSGVR TSL+GN+WNA +T  A      ++G D + +I ++GDD  I+S +P  L +
Sbjct  488  GGIPSGVRTTSLIGNIWNATMTSEARELTRLVMGEDLVKKIGIKGDDTYIISDNPICLVV  547

Query  482  LRLSYAAINAIGKDSKLGISPKVCEFLRNEISVTGVRGWTCRGIGGISQRKPWNPQPWSP  541
             RL+YAAINAIG DSK GIS +VCEFLR EIS+ G+RGWT R I  I+QRKPWN +PWSP
Sbjct  548  FRLAYAAINAIGSDSKFGISQRVCEFLRTEISMNGMRGWTNRAIPSITQRKPWNSKPWSP  607

Query  542  NDEVETNASNISLLERRAGIELQQLHHINKVKWSRHVRQSYKYLELPKRLGGFGIYRFQG  601
            + EV T A NI LLERRA   L  LH  NK+KWS+   QSY +L LP RLGG G+YRF+G
Sbjct  608  SSEVGTVADNIYLLERRAKKNLDILHLANKIKWSKFTNQSYHWLHLPVRLGGLGLYRFEG  667

Query  602  WLPNGKLPLAKKPLVNVEDIHPSQEL--FLPLSEQQKKILAQVEMTNKMQTDDIPGTQKL  659
            W PNG+LP   KP + V++I  S++   +  L+ QQ +  A   +   + +DDIPG Q+
Sbjct  668  WEPNGRLPPVTKPHIAVQNIKASRDYVKWAGLNRQQNEAFASEVLNMTIASDDIPGPQRF  727

Query  660  FSKEWIQKVRAKKIIWSRNQTIPIHTDHTVRIPKWDEKIKFPRYKSEYILNNKINLT---  716
            +SK++I ++R  K +W +  T     D  +  P   E + +P+   +   N+K  +
Sbjct  728  YSKDFINRLRQNKYVWQKVTTTITIFDPLIDCPPKSETVYWPK---KVKTNDKAIVDGMP  784

Query  717  -MEQVLRQYNLLKEVE  731
               Q +R++ +LK  E
Sbjct  785  RFAQFVREHQMLKNAE  800


>MAG: putative RNA-dependent RNA polymerase [Totiviridae sp.]
Sequence ID: UHS72487.1 Length: 869
Range 1: 27 to 869

Score:566 bits(1459), Expect:0.0,
Method:Compositional matrix adjust.,
Identities:322/853(38%), Positives:490/853(57%), Gaps:27/853(3%)

Query  3    RKAKLITAKKWQELTKGIYNASTLTPKIVDQIIKDE--GSGTDKTKYVNVPKIITDKELQ  60
            R+++ +  +  +E+TKG+++    T + +  I+K         K +Y    +   DK  +
Sbjct  27   RRSRRLPTRVEEEMTKGLFDGEVYTERDILAIVKGYVLRVKDSKEEYQWAERPRDDKACK  86

Query  61   TFYVPRSNADLVIRRIRLIDLWRNLKPDQMDEIRNYTHLDYIFVQNICIYMLVFGINTVK  120
              +  R  AD+ IRR++LIDL R L  ++ + IR    LDY+ V N+ IYM  +G
Sbjct  87   EIWPARVPADMRIRRLKLIDLIRELNDNEWNLIRRL-KLDYVLVSNLVIYMKTWGTRWFA  145

Query  121  HFRQIGLFNERNEFIEIAKQLSTKGKRFVDDVDNMKQKVCEIATIVGYM------DPNVD  174
                 G+      F ++++ +S++ KR   D +  KQ   E+  ++GY       D  +
Sbjct  146  RVYNTGILGSWRIFSQVSRLISSQVKRKKTDTEFTKQAFAELQALLGYQQLPFPGDGLIP  205

Query  175  KIDVMEEVNSLAAEGNEHGIDRDNWNDLFTKTCKEVM--TWYKGHEFISFDDYIKEGMWL  232
            + +   E+  LA  GNEHG+  D+W   F K  K++   T  +  ++++F++Y+KEG WL
Sbjct  206  EYNFFAELADLAKGGNEHGLIGDDWYSTFMKHLKKIKGKTPLEVVKYVTFEEYVKEGYWL  265

Query  233  TSGSSSIGKVHWTKDGENGKFKARKNMLLQIYTPQELANIVYAWDGKLHSRVFIKNEMSK  292
            TSGSSSIGKV WT +G+ GKFKARKNMLL +YT +EL  IV  WDG  H+   IKNE+ K
Sbjct  266  TSGSSSIGKVTWTFEGKEGKFKARKNMLLDVYTKEELWEIVSNWDGAQHTTAIIKNELGK  325

Query  293  LRLAVASNIEAYIHESYMLFLYGHGFKEYFGVTLDEKPDQQHQREIEMIEKLQAGYFGLP  352
            +RLAVASNIE YIHESY++   GH ++ + G+TLDE P    +R  +     ++G F LP
Sbjct  326  VRLAVASNIELYIHESYLMRQAGHTYRSWTGITLDESPTDNIRRVAKTSSLFRSGAFALP  385

Query  353  FDYASFDHQPTTFEVKTMVRRVGEIVVSQVPKNYYYQTQLLVNKIVNAYDKSYLSGNIKN  412
            FDYA FDHQ TT EV+ +V    +  +S VP++   +   ++ K V+AY    ++G   +
Sbjct  386  FDYAGFDHQVTTKEVQWIVDDYFKQGLSCVPQDRRAEFNKILVKTVDAYGVCDITGTT-D  444

Query  413  TKFENIKVKGGVPSGVRITSLLGNMWNAIITKIAINNVIGIIGYDPISQISLRGDDVAIL  472
             + +  KV GG+PSGVR+TSL+GN+WN ++T IA      I+GYDP   +SLRGDD  IL
Sbjct  445  GETKTYKVTGGLPSGVRVTSLVGNVWNGVLTSIAREVAKEILGYDPTLDVSLRGDDSLIL  504

Query  473  SKDPAALYLLRLSYAAINAIGKDSKLGISPKVCEFLRNEISVTGVRGWTCRGIGGISQRK  532
               P   Y +RL Y A+NAIG  +K GI     EFLR  I+  G  GWT R I  ++QRK
Sbjct  505  CHTPLECYAVRLGYQAVNAIGHSAKFGICQGEGEFLRTVITSNGNYGWTNRAIPSVTQRK  564

Query  533  PWNPQPWSPNDEVETNASNISLLERRAGIELQQLHHINKVKWSRHVRQSYKYLELPKRLG  592
            PWNP+PW PN +V+T ++NI LL RR+   ++ LH  N   W +      K+L LP RLG
Sbjct  565  PWNPEPWLPNHQVKTLSANIDLLGRRSQQSVEWLHRANTAAWCKITGLDKKWLHLPVRLG  624

Query  593  GFGIYRFQGWLPNGKLPLAKKPLVNVEDI--HPSQELFLPLSEQQKKILAQVEMTNKMQT  650
            G GIY F G  P G L   KKP + V +I  +P    +L L+++ +    Q  M NKM
Sbjct  625  GVGIYEFHGATPAGALHTGKKPAIQVRNIVDNPG-PTWLTLNDEDRSKYNQQVMINKMVA  683

Query  651  DDIPGTQKLFSKEWIQKVRAKKIIWSRNQTIPIHTDHTVRIPK--WDEKIKFP---RYKS  705
            DDIPG Q +FS+++ +K    +     ++ +  ++   + +PK   +  I +P   ++K
Sbjct  684  DDIPGVQAVFSRDFSRKNAGLRACQWHDEEMDTYSSVLISLPKPMTELNITWPSLDKHKF  743

Query  706  EYILNNKINLTMEQVLRQYNLLKEVERHDKDLKVPKLLDILDKWFPVQSSKIKTYESQGF  765
            E +     ++TM   +R++ ++++       ++ P L + +DK +P   + +K +E +G+
Sbjct  744  EPLDKTFPSVTM--FMREHTVVQQA-----GIECPTLREYMDKVYPKFWALVKGWEEKGW  796

Query  766  HRTDAINLAVGEIPTEPAVKINPILINFVKLHLERQGITHQRGRNKIAKFIYQKTKQAEN  825
            HRTDA+ LA+G IP EP  KINP+L  +VK +L   GI   RGR  I + I Q T+   +
Sbjct  797  HRTDAVELALGNIPCEPCPKINPLLTGYVKNYLVANGIYRWRGRRTIGEKISQFTRAGCD  856

Query  826  MILQSSLQQMYRY  838
            ++  S + + +RY
Sbjct  857  LLSTSEVGRYFRY  869


>MAG: putative RNA-dependent RNA polymerase, partial [Totiviridae sp.]
Sequence ID: UHS72509.1 Length: 403
Range 1: 19 to 400

Score:548 bits(1413), Expect:0.0,
Method:Compositional matrix adjust.,
Identities:261/382(68%), Positives:314/382(82%), Gaps:0/382(0%)

Query  9    TAKKWQELTKGIYNASTLTPKIVDQIIKDEGSGTDKTKYVNVPKIITDKELQTFYVPRSN  68
            T K+ +E+T GIYN+STLT   V  II  E    + T +  VPK ITDKELQT+YVPRSN
Sbjct  19   TNKEQKEVTNGIYNSSTLTVNKVKDIIMKERESPNNTNFKFVPKNITDKELQTYYVPRSN  78

Query  69   ADLVIRRIRLIDLWRNLKPDQMDEIRNYTHLDYIFVQNICIYMLVFGINTVKHFRQIGLF  128
             DLV+RRIRLID+WR    +Q  E+  YTHLDYIF QNI IY++VFGI TVK  R  G+F
Sbjct  79   GDLVLRRIRLIDIWRFSTEEQRQEMMYYTHLDYIFAQNIFIYIIVFGIETVKMLRNWGVF  138

Query  129  NERNEFIEIAKQLSTKGKRFVDDVDNMKQKVCEIATIVGYMDPNVDKIDVMEEVNSLAAE  188
            N RN+FI+IAK+LSTKGKRF+ D+DN KQK+CE+ATIVGYMDP VD+ID+MEEV SLA E
Sbjct  139  NNRNDFIKIAKELSTKGKRFIKDIDNWKQKICELATIVGYMDPAVDEIDIMEEVYSLAEE  198

Query  189  GNEHGIDRDNWNDLFTKTCKEVMTWYKGHEFISFDDYIKEGMWLTSGSSSIGKVHWTKDG  248
            GN HGID+  WN LF +T +EVMTWY+GHEF SF++Y+KEGMWLTSGSSSIGKV W+ D
Sbjct  199  GNSHGIDKTGWNKLFEQTVEEVMTWYRGHEFKSFEEYVKEGMWLTSGSSSIGKVEWSMDN  258

Query  249  ENGKFKARKNMLLQIYTPQELANIVYAWDGKLHSRVFIKNEMSKLRLAVASNIEAYIHES  308
            E+G FKARKNML+QIYTP+E+ +I   WDG+L SRVFIKNEMSKLRLAVASNIEAYI+ES
Sbjct  259  ESGHFKARKNMLMQIYTPEEIYDIARKWDGELKSRVFIKNEMSKLRLAVASNIEAYINES  318

Query  309  YMLFLYGHGFKEYFGVTLDEKPDQQHQREIEMIEKLQAGYFGLPFDYASFDHQPTTFEVK  368
            Y+LFLYGHGFK YFGVTLDE P +QH RE+E+I+ LQ+G++G PFDYASFDHQPTTFEV+
Sbjct  319  YILFLYGHGFKHYFGVTLDESPTEQHIREVEIIKLLQSGHYGFPFDYASFDHQPTTFEVQ  378

Query  369  TMVRRVGEIVVSQVPKNYYYQT  390
            T+V+R+ +IV+SQVP    Y+T
Sbjct  379  TIVKRMTDIVLSQVPVIAKYET  400


>MAG: putative RNA-dependent RNA polymerase, partial [Totiviridae sp.]
Sequence ID: UHS71733.1 Length: 361
Range 1: 4 to 356

Score:538 bits(1387), Expect:0.0,
Method:Compositional matrix adjust.,
Identities:251/353(71%), Positives:298/353(84%), Gaps:0/353(0%)

Query  96   YTHLDYIFVQNICIYMLVFGINTVKHFRQIGLFNERNEFIEIAKQLSTKGKRFVDDVDNM  155
            YTHLDYIF QNI IYM+VFG  TVK  R  G+FN R++FI+I+K+LSTKGKRF+ D+DN
Sbjct  4    YTHLDYIFAQNIFIYMIVFGTETVKMLRNWGIFNNRSDFIKISKELSTKGKRFIKDIDNW  63

Query  156  KQKVCEIATIVGYMDPNVDKIDVMEEVNSLAAEGNEHGIDRDNWNDLFTKTCKEVMTWYK  215
            KQK+CE+ATIVGYMDP VD+ID+MEEV SLA EGN+HGID+  WN LF +T + VMTWYK
Sbjct  64   KQKICELATIVGYMDPAVDEIDIMEEVYSLAEEGNDHGIDKTKWNKLFEQTVENVMTWYK  123

Query  216  GHEFISFDDYIKEGMWLTSGSSSIGKVHWTKDGENGKFKARKNMLLQIYTPQELANIVYA  275
            GHEF SF++Y+KEGMWLTSGSSSIGKV W+ D E G FKARKNML+QIYTP+E+ +I
Sbjct  124  GHEFKSFEEYVKEGMWLTSGSSSIGKVEWSMDEEKGHFKARKNMLMQIYTPEEIYDIARK  183

Query  276  WDGKLHSRVFIKNEMSKLRLAVASNIEAYIHESYMLFLYGHGFKEYFGVTLDEKPDQQHQ  335
            WDG+L SRVFIKNEMSKLRLAVASNIEAYI+ESY+LFLYGHGFK YFGVTLDE P +QH
Sbjct  184  WDGELKSRVFIKNEMSKLRLAVASNIEAYINESYILFLYGHGFKHYFGVTLDESPTEQHL  243

Query  336  REIEMIEKLQAGYFGLPFDYASFDHQPTTFEVKTMVRRVGEIVVSQVPKNYYYQTQLLVN  395
            RE+E+I+ LQ G++G PFDYASFDHQPTTFEV+T+V+R+ +IV+SQVP    Y+T  + N
Sbjct  244  REVEIIKLLQNGHYGFPFDYASFDHQPTTFEVQTIVKRMTDIVLSQVPVIAKYETSRICN  303

Query  396  KIVNAYDKSYLSGNIKNTKFENIKVKGGVPSGVRITSLLGNMWNAIITKIAIN  448
             I+N Y KSYLSGNI   +F+ IKV+GGVPSGVRITS LGNMWNA+ITKIA N
Sbjct  304  LIINGYSKSYLSGNINGVEFDRIKVRGGVPSGVRITSFLGNMWNAVITKIATN  356


>MAG: putative RNA-dependent RNA polymerase [Totiviridae sp.]
Sequence ID: UHS72421.1 Length: 734
Range 1: 1 to 734

Score:537 bits(1384), Expect:0.0,
Method:Compositional matrix adjust.,
Identities:302/744(41%), Positives:456/744(61%), Gaps:26/744(3%)

Query  111  MLVFGINTVKHFRQIGLFNERNEFIEIAKQLSTKGKRFVDDVDNMKQKVCEIATIVGYMD  170
            ML FG++  K   + G+    N F  +++++S K K++  + + +KQ   E+++++GY
Sbjct  1    MLSFGVDWFKKLARTGIMKNWNIFSAVSREVSVKVKKYKCESEMVKQHFGELSSLLGYQQ  60

Query  171  P-------NVDKIDVMEEVNSLAAEGNEHGIDRDNWNDLFTKTCKEVM------TWYKGH  217
                    +    D+ EE+N LA  G EHG++ +NWN    KTC  ++      T
Sbjct  61   LPFPGDTYSTKPYDLKEEMNELAKGGEEHGLEGENWN----KTCNTILDELIANTIVTKI  116

Query  218  EFISFDDYIKEGMWLTSGSSSIGKVHWTKDGENGKFKARKNMLLQIYTPQELANIVYAWD  277
            ++ISF+D++KEG WLT+GSSSIG+V W +  E+G FKARKNMLL++YT +EL ++V  WD
Sbjct  117  DYISFEDFVKEGKWLTNGSSSIGEVFWERGEESGHFKARKNMLLELYTAEELIDLVNKWD  176

Query  278  GKLHSRVFIKNEMSKLRLAVASNIEAYIHESYMLFLYGHGFKEYFGVTLDEKPDQQHQRE  337
             KL ++  IKNE+ K+RLAVAS IE Y+ ESY+ +L GH +K++ G+TLDE P ++ +R
Sbjct  177  CKLKNKTVIKNELGKVRLAVASPIEDYLIESYLFYLTGHLYKQWQGITLDETPSEELRRT  236

Query  338  IEMIEKLQAGYFGLPFDYASFDHQPTTFEVKTMVRRVGEIVVSQVPKNYYYQTQLLVNKI  397
            +E+   L   Y+ LPFDYA FD Q TT EV  +V++   + +  VP     +    +NKI
Sbjct  237  LEIQSYLAKHYYCLPFDYAGFDRQVTTDEVTIIVKKYFSLGLRNVPFEERTKYNNFINKI  296

Query  398  VNAYDKSYLSGNIKNTKFENIKVKGGVPSGVRITSLLGNMWNAIITKIAINNVIGIIGYD  457
            +  Y    L+G++ + ++  I V+GG+PSGVRITSL+GN+WN I+TK A+     ++ Y+
Sbjct  297  IMGYRNQTLTGSVGDKEY-TINVEGGLPSGVRITSLIGNIWNCIMTKRALEVAESLLDYN  355

Query  458  PISQISLRGDDVAILSKDPAALYLLRLSYAAINAIGKDSKLGISPKVCEFLRNEISVTGV  517
            P   ISLRGDD  I+ +     Y++RL Y +INAIG  +K GI+    EFLR   +
Sbjct  356  PAIHISLRGDDSLIVCRTALECYIVRLGYQSINAIGNSAKFGITQGEGEFLRTVYTAESR  415

Query  518  RGWTCRGIGGISQRKPWNPQPWSPNDEVETNASNISLLERRAGIELQQLHHINKVKWSRH  577
             GW  R I  I+QRKPWNP+ W PN  V+T  +NI LLERR    + +LH  NK++WS+
Sbjct  416  YGWVNRSIPSITQRKPWNPEVWEPNQNVKTIKNNIDLLERRLDHSVDKLHLANKIQWSKW  475

Query  578  VRQSYKYLELPKRLGGFGIYRFQGWLPNGKLPLAKKPLVNVEDIHPSQE--LFLPLSEQQ  635
            V QSYK+LELPKRLGG G+YRF+GW+ + KL L+ KP + V+++  +Q+  L++ LS+++
Sbjct  476  VHQSYKWLELPKRLGGVGLYRFEGWITDAKLSLSLKPSIFVQNLKGTQQKILWINLSKEE  535

Query  636  KKILAQVEMTNKMQTDDIPGTQKLFSKEWIQKVRAKKIIWSRNQTIPIHTDHTVRIPK-W  694
            ++   Q++   K+ + DIPG QK +++E + K +  K +W R   IPI     V++P+ W
Sbjct  536  EQKYNQLKFEKKIISSDIPGMQKYYTREVVGKYKKLKTVW-RKTEIPI----PVKMPQTW  590

Query  695  DEKIKFPRYKSEYILNNKINLTMEQVLRQYNLLKEVERHDKDLKVPKLLDILDKWFPVQS  754
             E  K  + K   I  +  +   EQ       L+E     +  K+  L + L+K +P
Sbjct  591  PEIEKTSKPKINKIKMDIYSPIDEQFPVLNAFLQEYSLVSRIKKINTLREYLEKEYPEFY  650

Query  755  SKIKTYESQGFHRTDAINLAVGEIPTEPAVKINPILINFVKLHLERQGITHQRGRNKIAK  814
             K++ YE QGFHRTDAI+LA G +P E   KI+PIL  F++  +E +GI + RGR  IA
Sbjct  651  VKMRAYERQGFHRTDAIDLARGLVPIENTTKIHPILTPFIQRSVEEKGIRYIRGRKNIAL  710

Query  815  FIYQKTKQAENMILQSSLQQMYRY  838
             +Y  T+ A   +  S L ++YRY
Sbjct  711  KLYNWTRSACIALSTSRLSRLYRY  734


>MAG: putative RNA-dependent RNA polymerase [Totiviridae sp.]
Sequence ID: UHS72429.1 Length: 738
Range 1: 3 to 738

Score:516 bits(1330), Expect:4e-174,
Method:Compositional matrix adjust.,
Identities:301/747(40%), Positives:448/747(59%), Gaps:25/747(3%)

Query  106  NICIYMLVFGINTVKHFRQIGLFNERNEFIEIAKQLSTKGKRFVDDVDNMKQKVCEIATI  165
            N+  Y++VFG+      +  G+F+    F  +++++ +K K F++    +KQK  E   +
Sbjct  3    NLITYLIVFGLEWFNKIKSWGIFSNYITFSVLSRKIGSKIK-FLNTSLELKQKFAEFNCL  61

Query  166  VGYM------DPNVDK-IDVMEEVNSLAAEGNEHGIDRDNWNDLFTKTCKEVMT--WYKG  216
            +GY       D  V K  D   E+ +LA  GN+HG+   NW + F ++ KEV     Y+
Sbjct  62   LGYQQLPFEGDGIVAKPFDYFSEIKNLANGGNKHGLRNTNWENTFKESLKEVSKSKLYEE  121

Query  217  HEFISFDDYIKEGMWLTSGSSSIGKVHWTKDGENGKFKARKNMLLQIYTPQELANIVYAW  276
             E+I F++Y+K+G WLTSGSSSIG V W  DG+ GKFKARKNM+  +YT  EL  +V  W
Sbjct  122  REYIEFEEYVKKGKWLTSGSSSIGHVEWEFDGDKGKFKARKNMIENVYTENELWQLVKNW  181

Query  277  DGKLHSRVFIKNEMSKLRLAVASNIEAYIHESYMLFLYGHGFKEYFGVTLDEKPDQQHQR  336
            DG + SR  IK EMSK+RLAVASNIE YI+ESY+++L GH +K ++G+TLD+  ++
Sbjct  182  DGTIKSRAIIKGEMSKIRLAVASNIEMYIYESYLMYLSGHVYKNWYGITLDQNTNEALND  241

Query  337  EIEMIEKLQAGYFGLPFDYASFDHQPTTFEVKTMVRRVGEIVVSQVPKNYYYQTQLLVNK  396
             I +    + G + LPFDYA+FDHQ TT+EV+ +     ++ +  VP   +   Q L+ K
Sbjct  242  NILIRTNCKEGNYLLPFDYAAFDHQVTTWEVQQITEFYYKLGLKNVPDEKFIYVQNLITK  301

Query  397  IVNAYDKSYLSGNIKNTKFENIKVKGGVPSGVRITSLLGNMWNAIITKIAINNVIGIIGY  456
             V AY KS +SG  K  KFE + V GG+PSG+R TSL+GN+WN+I+T I  N V+ ++GY
Sbjct  302  TVKAYSKSTISGEYKG-KFEKLDVIGGLPSGIRTTSLIGNIWNSIVTNIMKNLVVKLLGY  360

Query  457  DPISQISLRGDDVAILSKDPAALYLLRLSYAAINAIGKDSKLGISPKVCEFLRNEISVTG  516
            +PI  +SLRGDD  I+ + P   YL+R+ Y +INAIG  +K  I     EFLRN ++
Sbjct  361  NPIVHLSLRGDDSLIICRKPQECYLIRVCYLSINAIGHSNKFAIMKGTGEFLRNYLTKEN  420

Query  517  VRGWTCRGIGGISQRKPWNPQPWSPNDEVETNASNISLLERRAGIELQQLHHINKVKWSR  576
            V GW  R I  ++QRKPWNP+PW  N EV T  +NI LLERR+  +L++LH  NK+ WS+
Sbjct  421  VIGWVNRAIPSLTQRKPWNPEPWEDNHEVTTIKNNIDLLERRSSQDLEKLHLANKISWSK  480

Query  577  HVRQSYKYLELPKRLGGFGIYRFQGWLPNGKLPLAKKPLVNVEDIHPSQELF--LPLSEQ  634
              + S K+LELP+RLGG GIY F+G++P+ KLP   K  ++ E+I+     F  + L++
Sbjct  481  ITKLSTKWLELPRRLGGVGIYNFKGYIPDKKLPKISKYKIDFENINIKYPKFSWIELTDD  540

Query  635  QKKILAQVEMTNKMQTDDIPGTQKLFSKEWIQKVRAKKIIWSRNQ--TIPIHTDHT-VRI  691
            Q   + ++++  K+  DDIPG Q  F +  + K +  K+ W + +   I    D+T +
Sbjct  541  QLTKVNKIDLQTKIVADDIPGFQSKFHRNLMDKYKQLKLNWQKTELDLISPKIDYTYLPT  600

Query  692  PKWDEKIKFPRYKSEYILNNKINLTMEQVLRQYNLLKEVERHDKDLKVPKLLDILDKWFP  751
             K+   IK  R K+ Y+      + + + LRQY L+++V     D  V  + + L +++P
Sbjct  601  CKYPTIIKRIRPKN-YLYK---EMPIWEFLRQYGLVRQV-----DETVKPMTEYLKEFYP  651

Query  752  VQSSKIKTYESQGFHRTDAINLAVGEIPTEPAVKINPILINFVKLHLERQGITHQRGRNK  811
                 +K +E  G+HRTDAINLA+GE+  EP   IN  L + VKL + +  I    GR K
Sbjct  652  QFYKDMKKWERIGWHRTDAINLAMGELVIEPTDLINSQLTDIVKLLVWKTDIKKTFGRKK  711

Query  812  IAKFIYQKTKQAENMILQSSLQQMYRY  838
            IA  + + TK    ++ Q  +   +RY
Sbjct  712  IAIKLSRYTKYMSKVVSQDIVSTYFRY  738


>MAG: putative RNA-dependent RNA polymerase [Totiviridae sp.]
Sequence ID: UHS72490.1 Length: 787
Range 1: 18 to 787

Score:510 bits(1314), Expect:4e-171,
Method:Compositional matrix adjust.,
Identities:300/795(38%), Positives:457/795(57%), Gaps:33/795(4%)

Query  52   KIITDKELQTFYVPRSNADLVIRRIRLIDLWRNLKPDQMDEIRNYTHLDYIFVQNICIYM  111
            K+  D E  +F   R  AD+  RR++L +L     P   + I    HLD I V N+ I+
Sbjct  18   KVNVDVETISF---RCKADISTRRLKLSNL--TTLPHFQEVIGWTHHLDQILVANLVIWT  72

Query  112  LVFGINTVKHFRQIGLFNERNEFIEIAKQLSTKGKRFVDDVDNMKQKVCEIATIVGYMDP  171
             + GI+     +++G  N   +FI I+  +S+  KRF  D D  KQ   EI ++ GY+
Sbjct  73   YINGIDKFHDLKRLGYLNSITDFINISF-ISSYVKRFPTD-DGSKQIFAEINSLTGYLQN  130

Query  172  NVDKIDVMEEVNSLAAEGNEHGIDRDN----WNDLFTKTCKEVMTWYKGHEFISFDDYIK  227
            +++++D  E+++ LA  G EHG+  ++    + D+    C       + H FI F DYI+
Sbjct  131  DIEEVDWHEKLSELANGGAEHGLPGEDTIKTFTDILNLICPPTT---RPHLFIGFYDYIE  187

Query  228  EGMWLTSGSSSIGKVHWTKDGENGKFKARKNMLLQIYTPQELANIVYAWDGKLHSRVFIK  287
             G+WLT+GSSSIGKV+W    E G FKARKNM+  +YT  EL ++   WDG+L SR F K
Sbjct  188  SGLWLTAGSSSIGKVYWETTTEKGHFKARKNMVTYLYTTDELYDLCKRWDGRLISRAFTK  247

Query  288  NEMSKLRLAVASNIEAYIHESYMLFLYGHGFKEYFGVTLDEKPDQQHQREIEMIEKLQAG  347
            +E+SK R+AVASNIE+Y+ ESY+L+   H +K +  +TLDE P  +H R IE+ + L++G
Sbjct  248  DELSKRRIAVASNIESYLCESYLLYNMTHVYKSWKYITLDESPAVEHNRTIEVSKLLKSG  307

Query  348  YFGLPFDYASFDHQPTTFEVKTMVRRVGEIVVSQVPKNYYYQTQLLVNKIVNAYDKSYLS  407
             + LPFD+ +FDHQPTT E++T++    ++    VPK Y  +   +  K++N+Y  + +S
Sbjct  308  KWALPFDFKNFDHQPTTEEIQTIMSH--DLNGVDVPKQYLQEWNNIKYKVINSYSNNTMS  365

Query  408  GNIKNTKFENIKVKGGVPSGVRITSLLGNMWNAIITKIAINNVIGIIGYDPISQISLRGD  467
             +I N K E + VKGG+PSGVR TSL+GN WN ++++ A N    I+GYDP+  + ++GD
Sbjct  366  MSINNKKVE-LDVKGGLPSGVRWTSLIGNQWNMVMSQWARNITTKILGYDPVLTMGIKGD  424

Query  468  DVAILSKDPAALYLLRLSYAAINAIGKDSKLGISPKVCEFLRNEISVTGVRGWTCRGIGG  527
            D  I++     L + R +YA INAIG +SK GI  +VCEFLR EIS    RGWT R I
Sbjct  425  DTYIIASRAVDLCIFREAYAMINAIGLNSKFGIQKRVCEFLRVEISGDRARGWTNRSIPT  484

Query  528  ISQRKPWNPQPWSPNDEVETNASNISLLERRAGIELQQLHHINKVKWSRHVRQSYKYLEL  587
            I+QRKPWN +PW+    VE  A +I+  ERR    L +LH INK +WS+H   S ++LEL
Sbjct  485  ITQRKPWNAEPWTIGSGVEAVAQSINTTERRCYKSLTELHTINKTRWSKHFGLSTRWLEL  544

Query  588  PKRLGGFGIYRFQGWLPNGKLPLA-KKPLVNVEDIHPSQEL-FLPLSEQQKKILAQVEMT  645
            P+  GGFG+Y +  + P+  L +  ++P V  +D+ P +EL ++ L+E ++K +A   +T
Sbjct  545  PRIFGGFGVYPWNYYKPSEPLRIKNERPKVKFKDVVPYKELTWIQLTEDERKTMAINALT  604

Query  646  NKMQTDDIPGTQKLFSKEWIQKVRAKKIIWSRNQTIPIHTDHTVRIPKWDEKIKFPRYKS  705
                TDDI         + ++  R  K+ W + Q          RI  W   ++ P
Sbjct  605  AATHTDDIRPIYSQTKHDILKTARKTKVRWYKYQP-------AFRI-SWFGGLETPDLTG  656

Query  706  EYILNNKINLTMEQVLRQYNLLKEVERHDKDLK--VPKLLDILDKWFPVQSSKIKTYESQ  763
             +    K     EQ      ++ +       +K  +P L+  L  + P  +  +K YES+
Sbjct  657  YW---PKFAGGFEQQKEALEIITDYSYLPNHIKQEIP-LIKALQDYAPAFAQGVKQYESR  712

Query  764  GFHRTDAINLAVGEIPTEPAVKINPILINFVKLHLERQGITHQRGRNKIAKFIYQKTKQA  823
            G+HRTDAIN+ +G +PT    K++P +I FV+  L    I +Q GR +I   I   T+ +
Sbjct  713  GWHRTDAINIGLGIVPTSTTFKLHPKMIPFVRSSLINTRILYQVGRKQILDHIDTTTQAS  772

Query  824  ENMILQSSLQQMYRY  838
             + I QS+  ++Y+Y
Sbjct  773  NHAIQQSAGYKLYQY  787


>MAG: putative RNA-dependent RNA polymerase, partial [Totiviridae sp.]
Sequence ID: UHS72526.1 Length: 647
Range 1: 5 to 642

Score:498 bits(1282), Expect:3e-168,
Method:Compositional matrix adjust.,
Identities:269/651(41%), Positives:396/651(60%), Gaps:17/651(2%)

Query  190  NEHGIDRDNWNDLFTKTCKEVMTWYKGHEFISFDDYIKEGMWLTSGSSSIGKVHWTKDGE  249
            NEHG+   +W+  F +            + ++  +YI+   WLT+GSSSIG+V WT D +
Sbjct  5    NEHGLVNSDWDSDFEEALVSTTGLVPKVKQMTLREYIESKRWLTAGSSSIGEVEWTFDEK  64

Query  250  NGKFKARKNMLLQIYTPQELANIVYAWDGKLHSRVFIKNEMSKLRLAVASNIEAYIHESY  309
            +GKFKARKNML  ++T  EL ++VY W+G+L S+ F K+E+SK R+AVASNIEAY+ ESY
Sbjct  65   SGKFKARKNMLTYLFTVDELVDMVYKWNGRLMSKAFTKDELSKRRIAVASNIEAYLSESY  124

Query  310  MLFLYGHGFKEYFGVTLDEKPDQQHQREIEMIEKLQAGYFGLPFDYASFDHQPTTFEVKT  369
            +L+  GH +K +   TLDE P +QH R  +++  LQ G + LPFD+ +FDHQPTT E++
Sbjct  125  VLYHLGHPYKNWEYFTLDESPGKQHARNADIMGLLQRGEWALPFDFKNFDHQPTTKEIQM  184

Query  370  MVRRVGEIVVSQVPKNYYYQTQLLVNKIVNAYDKSYLSGNIKNTKFENIKVKGGVPSGVR  429
            +V +  E++      N     +L+  ++V +Y+ S +  + KN K     V GG+PSGVR
Sbjct  185  IVNKYIELI--NAGNNMSSDFELISRRVVASYENSVIEMS-KNGKTWKETVTGGLPSGVR  241

Query  430  ITSLLGNMWNAIITKIAINNVIGIIGYDPISQISLRGDDVAILSKDPAALYLLRLSYAAI  489
            +TSLLGN WNA +T I       + G D  S I ++GDD  I+S  P  L   RL Y AI
Sbjct  242  LTSLLGNQWNAAMTHIVRGMASRLCGLDLTSTIGIKGDDTYIISSSPGFLACFRLCYEAI  301

Query  490  NAIGKDSKLGISPKVCEFLRNEISVTGVRGWTCRGIGGISQRKPWNPQPWSPNDEVETNA  549
            NAIG D+K  I P  CEFLRN IS +G+RGW+ R I  ++QRKPW+PQPW P+  V T A
Sbjct  302  NAIGLDAKFAIMPTECEFLRNAISKSGIRGWSNRAIPTLTQRKPWSPQPWKPDAAVATVA  361

Query  550  SNISLLERRAGIELQQLHHINKVKWSRHVRQSYKYLELPKRLGGFGIYRFQGWLPNGKLP  609
              I  LERR G  + +LH +N+ KWS+   QS  +L LP R+GG+GIY F G +PN  LP
Sbjct  362  DGIWTLERRLGYYIPRLHMVNRKKWSKFTGQSDNWLRLPIRMGGYGIYPFTGKVPNNTLP  421

Query  610  LAKKPLVNVE-DIHPS--QELFLPLSEQQKKILAQVEMTNKMQTDDIPGTQKLFSKEWIQ  666
              + P +N     H +  Q  ++ L+E+Q     QV +T  M TDDIPGTQ +F ++++
Sbjct  422  TIELPKINTNIPKHNAIEQPKWIKLNEEQFTAYQQVGLTQMMATDDIPGTQSVFGRKFLN  481

Query  667  KVRAKKIIWSRNQTIP-IHTDHTVRIPKWDEKIKFPRYKSEYILNNKINLTMEQVLRQYN  725
             +R  ++ W+ +  +P       VR P       +P  K +   N+++ +++ Q LR+
Sbjct  482  NIRNLRVEWADSPLVPDPKLWKKVRGPSQKLVGPWPSDKVKVDYNDELEMSVTQFLREAK  541

Query  726  LLKEVERHDKDLKVPKLLDILDKWFPVQSSKIKTYESQGFHRTDAINLAVGEIPTEPAVK  785
            L+++V      + +P + ++L +++P    ++K +E++G+HR DAI+LA G  PTE   +
Sbjct  542  LVQKV------MDIP-IAELLKQFYPYTYIQMKAFETRGWHRADAIDLAQGITPTERVTR  594

Query  786  INPILINFVKLHLERQGITHQRGRNKIAKFIYQKTKQAENMILQSSLQQMY  836
            INPIL +FVK  ++  G+   RGR  IA  +YQ T   EN +LQ S Q +Y
Sbjct  595  INPILTSFVKEAVQNTGVRFWRGRKNIASKLYQVT---ENAVLQLSNQNIY  642


>MAG: putative RNA-dependent RNA polymerase [Totiviridae sp.]
Sequence ID: UHS72476.1 Length: 772
Range 1: 14 to 772

Score:492 bits(1267), Expect:3e-164,
Method:Compositional matrix adjust.,
Identities:291/782(37%), Positives:450/782(57%), Gaps:32/782(4%)

Query  66   RSNADLVIRRIRLIDLWRNLKP-DQMDEIRNYTH-LDYIFVQNICIYMLVFGINTVKHFR  123
            R  AD+   RI+L     NLK     +E+ ++TH LD + + N+ I+  + G+      +
Sbjct  14   RCKADISTLRIKL----NNLKTLPCYNEVISWTHHLDPLLISNLIIWTHINGMKAFLDIK  69

Query  124  QIGLFNERNEFIEIAKQLSTKGKRFVDDVDNMKQKVCEIATIVGYMDPNVDKIDVMEEVN  183
            Q+G  N   +F++++  +S+  KRF  D D  KQ   EI ++ GY+  ++++ D  E+++
Sbjct  70   QLGYLNSIQDFVDMS-FISSYVKRFPSD-DGSKQIFAEINSLTGYLQNDIEETDWHEKMS  127

Query  184  SLAAEGNEHGIDRDN----WNDLFTKTCKEVMTWYKGHEFISFDDYIKEGMWLTSGSSSI  239
             LA  G EHG+   +    +++L    C       K H F++  DYI  G+WLT+GSSSI
Sbjct  128  ELANGGAEHGLPGQDTIKTFSELLDLVCPPTN---KPHLFLNLYDYIDSGIWLTAGSSSI  184

Query  240  GKVHWTKDGENGKFKARKNMLLQIYTPQELANIVYAWDGKLHSRVFIKNEMSKLRLAVAS  299
            GKV+W    + G FKARKNM+  +YT  EL ++   WDG+L SR F K+E+SK R+AVAS
Sbjct  185  GKVYWETGTDKGHFKARKNMITYLYTTSELYDLCKRWDGRLISRAFTKDELSKRRIAVAS  244

Query  300  NIEAYIHESYMLFLYGHGFKEYFGVTLDEKPDQQHQREIEMIEKLQAGYFGLPFDYASFD  359
            NIE+Y+ ESY+L+   H +K +  +TLDE P  +H R IE+ + L+ G + LPFD+ +FD
Sbjct  245  NIESYLCESYLLYNMTHIYKSWKYITLDETPAVEHNRTIELSKLLKDGKWALPFDFKNFD  304

Query  360  HQPTTFEVKTMVRRVGEIVVSQVPKNYYYQTQLLVNKIVNAYDKSYLSGNIKNTKFENIK  419
            HQPTT E++T++R   ++   +VPK +  +      K++N+Y  + +S NI N K + I
Sbjct  305  HQPTTEEIQTIMRH--DLNGVKVPKQHLDEWNKTKYKVINSYSNNTMSMNIDNKKVK-ID  361

Query  420  VKGGVPSGVRITSLLGNMWNAIITKIAINNVIGIIGYDPISQISLRGDDVAILSKDPAAL  479
            VKGG+PSGVR TSL+GN WN I+++ A N    I+GYDP+  + ++GDD  I++     L
Sbjct  362  VKGGLPSGVRWTSLIGNQWNMIMSQWARNITTKILGYDPVLTMGIKGDDTYIIANRAVDL  421

Query  480  YLLRLSYAAINAIGKDSKLGISPKVCEFLRNEISVTGVRGWTCRGIGGISQRKPWNPQPW  539
             + R +YA INA+G +SK GI  +VCEFLR EIS +  RGWT R I  I+QRKPWN +PW
Sbjct  422  CIFREAYAMINAVGLNSKFGIQKRVCEFLRVEISGSNARGWTNRSIPTITQRKPWNAEPW  481

Query  540  SPNDEVETNASNISLLERRAGIELQQLHHINKVKWSRHVRQSYKYLELPKRLGGFGIYRF  599
            S    V   A  IS  ERR   +L  LH IN+ +WSRH   S ++LELP+  GGFGIY +
Sbjct  482  SIEAGVGAVAQAISTTERRCFKDLNDLHDINRKRWSRHFGLSTRWLELPRLYGGFGIYPW  541

Query  600  QGWLPNGKLPLA-KKPLVNVEDIHPSQEL-FLPLSEQQKKILAQVEMTNKMQTDDIPGTQ  657
            + + P   L +   KP +  +DI P +EL ++ L+++++  +A   M+    TDDI
Sbjct  542  KYYKPTDPLKIKNNKPTIKYKDIVPYKELSWINLTDEERTTMAVNAMSAATHTDDIRPIY  601

Query  658  KLFSKEWIQKVRAKKIIWSRNQTIPIHTDHTVRIPKWDEKIKFPRYKSEYILNNKINLTM  717
                ++ ++  R  K  W + +        + RI  W   ++ P +   +    K
Sbjct  602  TQTKQDIVKATRKVKTRWYKYEP-------SFRI-GWYGGLETPDFTGYW---PKFMGGF  650

Query  718  EQVLRQYNLLKEVERHDKDLKVP-KLLDILDKWFPVQSSKIKTYESQGFHRTDAINLAVG  776
            EQ      ++ +      D+K    L++ L +  P  +  +K YES+G+HRTDAIN+ +G
Sbjct  651  EQQKEALEIISDYSYLPNDVKQRITLMEALQQNAPSFAQGVKQYESRGWHRTDAINIGLG  710

Query  777  EIPTEPAVKINPILINFVKLHLERQGITHQRGRNKIAKFIYQKTKQAENMILQSSLQQMY  836
             +PT  A  ++P +  F++  L    I  Q GR +I   I+  T+ +   I QS+  ++Y
Sbjct  711  IVPTSTAFGLHPKMTPFIRSSLVNSRILFQSGRKQILDHIHTTTQASTQAIKQSAGYKLY  770

Query  837  RY  838
            +Y
Sbjct  771  QY  772


>MAG: putative RNA-dependent RNA polymerase, partial [Totiviridae sp.]
Sequence ID: UHS72534.1 Length: 497
Range 1: 1 to 497

Score:457 bits(1176), Expect:2e-154,
Method:Compositional matrix adjust.,
Identities:230/506(45%), Positives:327/506(64%), Gaps:18/506(3%)

Query  59   LQTFYVPRSNADLVIRRIRLIDLWRNLKPDQMDEIRNYTHLDYIFVQNICIYMLVFGINT  118
            ++  + PR+ ADL IRR+++ D+ +    D ++E+  YTHLDYIFV NI +Y ++ G+ T
Sbjct  1    MKDIFKPRNKADLSIRRLKMDDISKLCPEDILNEMMYYTHLDYIFVCNIMLYTIIHGVET  60

Query  119  VKHFRQIGLFNERNEFIEIAKQLSTKGKRFVDDVDNMKQKVCEIATIVGYMDPNVDKIDV  178
             +  R  G+FN  + FI++A ++S+  KR     D+ KQ++CE+A++ GY+  + +K +
Sbjct  61   TQQLRSFGIFNSFDTFIDLAGEISSYFKRSPWCTDDTKQRLCELASLTGYLQNDTEKWNT  120

Query  179  MEEVNSLAAEGNEHGIDRDNWNDLFTKTCKEVMTWYKGHE-------FISFDDYIKEGMW  231
             +E+  LA  G EHG+   +W      T KE     +  +       F +F++Y+K   W
Sbjct  121  KDELAGLADGGEEHGLVGTDW----LTTYKEAFVSMQHGKYPNVYPTFTTFEEYVKSNKW  176

Query  232  LTSGSSSIGKVHWTKDGENGKFKARKNMLLQIYTPQELANIVYAWDGKLHSRVFIKNEMS  291
            LTSGS SIG+V W  D E GKFKA KNM+  IYT +EL +IV  WDG LHSR FIK+E+
Sbjct  177  LTSGSCSIGEVTWHYDQEEGKFKAHKNMIHDIYTDEELWDIVSKWDGILHSRAFIKDEVG  236

Query  292  KLRLAVASNIEAYIHESYMLFLYGHGFKEYFGVTLDEKPDQQHQREIEMIEKLQAGYFGL  351
            K RLAVASNIEAY+HESY+L+LYGH FK + G+TLDE P +QH+R+ + I  L+ G + L
Sbjct  237  KRRLAVASNIEAYLHESYILYLYGHNFKNWDGITLDESPQEQHRRKSKAINLLKRGAYAL  296

Query  352  PFDYASFDHQPTTFEVKTMVRRVGEIVVSQVPKNYYYQTQLLVNKIVNAYDKSYLSGNIK  411
            PFD+  FDHQP T E++  +  + + +   +P  Y    + +VNK++ +YD S +S  I
Sbjct  297  PFDFKRFDHQPQTKEIQITMSNIADQI--PIPSKYLRDWRKIVNKVIQSYDNSEISMQIG  354

Query  412  NTKFENIK--VKGGVPSGVRITSLLGNMWNAIITKIAINNVIGIIGYDPISQISLRGDDV  469
            +   EN+K  V+GG+PSGVR TSL GN WN  ITKIAIN    ++    + Q  ++GDD
Sbjct  355  S---ENVKHMVEGGLPSGVRSTSLTGNGWNGAITKIAINTAEKVLQVLTVLQKMIKGDDT  411

Query  470  AILSKDPAALYLLRLSYAAINAIGKDSKLGISPKVCEFLRNEISVTGVRGWTCRGIGGIS  529
             +     +  YL+R+ YAAINA+G D+K GIS  +CEFLRNEIS TGVRGW+ R I  ++
Sbjct  412  GLFVNTVSEAYLIRMCYAAINAVGSDAKFGISQNICEFLRNEISTTGVRGWSNRTIPTLT  471

Query  530  QRKPWNPQPWSPNDEVETNASNISLL  555
            QRKPWN +PWSPN  + T + N  LL
Sbjct  472  QRKPWNSEPWSPNAMISTLSQNTYLL  497


>MAG: putative RNA-dependent RNA polymerase, partial [Totiviridae sp.]
Sequence ID: UHS72482.1 Length: 583
Range 1: 46 to 583

Score:438 bits(1127), Expect:7e-146,
Method:Compositional matrix adjust.,
Identities:233/543(43%), Positives:325/543(59%), Gaps:9/543(1%)

Query  66   RSNADLVIRRIRLIDLWRNLKPDQMDEIRNYT-HLDYIFVQNICIYMLVFGINTVKHFRQ  124
            R+  D  IRRI+  DL   +  D + +   Y  H+D I+  N+ I+  + GI +    +
Sbjct  46   RTKLDSSIRRIKARDL-AMVCQDTISKCVPYMGHMDQIWATNLIIWADIHGIESFMQLKH  104

Query  125  IGLFNERNEFIEIAKQLSTKGKRFVDDVDNMKQKVCEIATIVGYMDPNVDKIDVMEEVNS  184
            +G+ N    F E + +LS+  KRF  +    K+K  E+  + GY++ +++ +D  E+
Sbjct  105  LGMLNSLTHFTEQSSRLSSWVKRFSTNETINKEKYAELNGLSGYLNNDLEPVDWFEKTRE  164

Query  185  LAAEGNEHGIDRDNWNDLFTKTCKEV---MTWYKGHEFISFDDYIKEGMWLTSGSSSIGK  241
            LA  GN HG+   +W   F    K++    +     EFI+F D+IK G W+TSGSS+ G
Sbjct  165  LAEGGNPHGLINYDWETEFKNANKQIKLKASHDSKPEFITFYDFIKTGDWITSGSSAQGH  224

Query  242  VHWTKDGENGKFKARKNMLLQIYTPQELANIVYAWDGKLHSRVFIKNEMSKLRLAVASNI  301
            + W  +GE+GKFKARKNML  IYTP+EL ++   WDG   +  F K+E+ K RLAVAS++
Sbjct  225  IEWEMEGESGKFKARKNMLEYIYTPEELYDLCVKWDGVNENVAFTKDELGKRRLAVASSL  284

Query  302  EAYIHESYMLFLYGHGFKEYFGVTLDEKPDQQHQREIEMIEKLQAGYFGLPFDYASFDHQ  361
            E+Y+ +SY L L+GH +K +  VTLDE  ++ H R   M + L  GY+ LPFD+ +FDHQ
Sbjct  285  ESYLLDSYFLRLFGHPYKNWDYVTLDESAEETHNRTATMSQLLSRGYYALPFDFKNFDHQ  344

Query  362  PTTFEVKTMVRRVGEIVVSQVPKNYYYQTQLLVNKIVNAYDKSYLSGNIKNTKFENIKVK  421
                E+  M+     +    +P  Y  + + +  KI+++Y  S ++ + +  K    KV
Sbjct  345  AQQAEIVWMME--SNLSSISIPVGYKCEWKWIKEKIMHSYTHSTITFSDQKEK-RVYKVT  401

Query  422  GGVPSGVRITSLLGNMWNAIITKIAINNVIGIIGYDPISQISLRGDDVAILSKDPAALYL  481
            GG+PSGVR TSLLGN WNA IT+I  N V  + G  P   I +RGDD  I+ + P  L +
Sbjct  402  GGLPSGVRTTSLLGNEWNATITRIVTNLVTKLTGKSPC-LIGVRGDDTYIIDRSPILLAI  460

Query  482  LRLSYAAINAIGKDSKLGISPKVCEFLRNEISVTGVRGWTCRGIGGISQRKPWNPQPWSP  541
             R  Y AINAIG D K GI+   CEFLRNEI+  G RGW  R I  I+QRKPWNP PW
Sbjct  461  TREMYKAINAIGHDEKFGITLGACEFLRNEITSEGSRGWANRTIPTITQRKPWNPSPWGI  520

Query  542  NDEVETNASNISLLERRAGIELQQLHHINKVKWSRHVRQSYKYLELPKRLGGFGIYRFQG  601
              +V+T A NI  LERR G E  +LH +NK+KWSR+ +QSYK+LELP R GGFGIY  QG
Sbjct  521  KQQVQTTADNIRTLERRLGTEAPRLHQVNKIKWSRYFKQSYKWLELPTREGGFGIYPDQG  580

Query  602  WLP  604
            W P
Sbjct  581  WRP  583


>MAG: putative RNA-dependent RNA polymerase, partial [Totiviridae sp.]
Sequence ID: UHS72491.1 Length: 531
Range 1: 5 to 528

Score:416 bits(1068), Expect:8e-138,
Method:Compositional matrix adjust.,
Identities:227/543(42%), Positives:328/543(60%), Gaps:19/543(3%)

Query  16   LTKGIYNASTLTPKIVDQIIKDEGSGTDKTKYVNVPKIITDKELQTFYVPRSNADLVIRR  75
            +TKG+     L+  +   +++  GS  D T Y        D ++ T +  R+ ADL  RR
Sbjct  5    VTKGLIYDEPLSHSMFLNLVRLYGS-QDTTTY-------NDYDIHTTFEQRTQADLSTRR  56

Query  76   IRLIDLWRNLKPDQMDEIRNYTHLDYIFVQNICIYMLVFGINTVKHFRQIGLFNERNEFI  135
            ++L DL  +L P     +   THLD + V NI ++  V G +     ++ G FN    FI
Sbjct  57   LKLADLKCDLTP-----LNWLTHLDLLLVTNILLWANVHGTDYFYLLKENGYFNSIESFI  111

Query  136  EIAKQLSTKGKRFVDDVDNMKQKVCEIATIVGYMDPNVDKIDVMEEVNSLAAEGNEHGID  195
            + A +L +  KR +   D+ KQ + E+  + GY+  ++  +    E+++LA  G+EHG
Sbjct  112  DQASKLGSHIKRPMGG-DDYKQILAELNVLTGYLQNDITPVQWSYELDALAKGGSEHGFG  170

Query  196  RDNWNDLFTKTCKEVMTWYKGHEFISFDDYIKEGMWLTSGSSSIGKVHWTKDGENGKFKA  255
              +W  LFTKT ++         FISF D+I    W T GSSSIG V W+ D ++G+FKA
Sbjct  171  --DWPSLFTKTARQNSFIQSQPNFISFKDFIYSKEWTTPGSSSIGHVRWSYDDDDGRFKA  228

Query  256  RKNMLLQIYTPQELANIVYAWDGKLHSRVFIKNEMSKLRLAVASNIEAYIHESYMLFLYG  315
            RKNML +IY  + + ++   W+G+L S+ F K+E+SK R+AVASN+EAY++ESY+L + G
Sbjct  229  RKNMLPEIYPMETIYDLALNWNGELRSKAFTKDELSKRRIAVASNLEAYLNESYILKIIG  288

Query  316  HGFKEYFGVTLDEKPDQQHQREIEMIEKLQAGYFGLPFDYASFDHQPTTFEVKTMVRRVG  375
            H +K +  +TLDE P +QH R  ++++ L  G + LPFD+ +FDHQPTT E++T+V  +
Sbjct  289  HAYKSWNFITLDETPAEQHARNQKLMKLLSQGQWALPFDFKAFDHQPTTEEIQTLVTVIC  348

Query  376  EIVVSQVPKNYYYQTQLLVNKIVNAYDKSYLSGNIKNTKFENIKVKGGVPSGVRITSLLG  435
            ++   Q+P  Y  + +++  + + +Y  S +   I N + +  KV GG+PSGVRITSL+G
Sbjct  349  DL--PQIPPPYREEWKMITTRTIQSYANSTIEMTI-NGQNKKTKVTGGLPSGVRITSLIG  405

Query  436  NMWNAIITKIAINNVIGIIGYDPISQISLRGDDVAILSKDPAALYLLRLSYAAINAIGKD  495
            N WN +IT IA      I+GY PI  I+LRGDD  ILS  P  L + RL+Y+AINA G D
Sbjct  406  NFWNQVITSIAQTVTNNILGYKPIFTIALRGDDTYILSSRPVELVVFRLAYSAINAKGLD  465

Query  496  SKLGISPKVCEFLRNEISVTGVRGWTCRGIGGISQRKPWNPQPWSPNDEVETNASNISLL  555
            SK GI    CEFLRN  +   VRGWT R I  I+QRKPWNPQPW+P  +V T A NI  L
Sbjct  466  SKFGICKFNCEFLRNIATDKVVRGWTNRAIPSITQRKPWNPQPWTPGSDVMTVADNIRTL  525

Query  556  ERR  558
            ERR
Sbjct  526  ERR  528


>MAG: putative RNA-dependent RNA polymerase, partial [Totiviridae sp.]
Sequence ID: UHS72480.1 Length: 403
Range 1: 7 to 402

Score:407 bits(1045), Expect:3e-136,
Method:Compositional matrix adjust.,
Identities:200/400(50%), Positives:277/400(69%), Gaps:8/400(2%)

Query  219  FISFDDYIKEGMWLTSGSSSIGKVHWTKDGENGKFKARKNMLLQIYTPQELANIVYAWDG  278
            FI+F++Y+K G W+TSGSSSIG V W+KD + G F+ARKNM+L +YT +EL  IV  WDG
Sbjct  7    FITFEEYVKSGYWITSGSSSIGLVEWSKDEDTGHFRARKNMILDLYTKEELWQIVKDWDG  66

Query  279  KLHSRVFIKNEMSKLRLAVASNIEAYIHESYMLFLYGHGFKEYFGVTLDEKPDQQHQREI  338
             + +R  IKNE+ K+RLAVA  IE Y+ ESY+  + GH +K + G+TLDE P+++  R +
Sbjct  67   NIENRAIIKNELGKVRLAVACPIEMYLCESYLFEMTGHLYKTWDGITLDETPEEELNRTL  126

Query  339  EMIEKLQAGYFGLPFDYASFDHQPTTFEVKTMVR---RVGEIVVSQVPKNYYYQTQLLVN  395
            E+   L+ G + LPFDYA FDHQ TT EV+ +V    + GEI    +P ++    + + +
Sbjct  127  EIQRLLKQGSYCLPFDYAGFDHQVTTKEVEWIVDYYFQKGEI---NIPPDHLLNFRKIRS  183

Query  396  KIVNAYDKSYLSGNIKNTKFENIKVKGGVPSGVRITSLLGNMWNAIITKIAINNVIGIIG  455
            K+V AY KS ++    N K  +  ++GG+PSGVRITSL+GN+WN+++T+I+ N V+ +I
Sbjct  184  KVVKAYSKSKITTTQDNKKI-SFDIEGGLPSGVRITSLIGNIWNSVMTRISRNLVVKLIN  242

Query  456  YDPISQISLRGDDVAILSKDPAALYLLRLSYAAINAIGKDSKLGISPKVCEFLRNEISVT  515
            YDP+ Q+SLRGDD  I+ +     Y+ RLSY AINAIG  +K GI+ +  EFLR  I+ T
Sbjct  243  YDPVQQLSLRGDDSLIICRSIVECYIFRLSYQAINAIGNSNKFGITAQEGEFLRTVITKT  302

Query  516  GVRGWTCRGIGGISQRKPWNPQPWSPNDEVETNASNISLLERR-AGIELQQLHHINKVKW  574
               GW CR I  I+QRKPWNP+PW PN  V T   NI LLERR     + +LH  NK++W
Sbjct  303  DKFGWVCRAIPSITQRKPWNPEPWEPNHMVITIKQNIDLLERRLQNNNVVKLHQANKLQW  362

Query  575  SRHVRQSYKYLELPKRLGGFGIYRFQGWLPNGKLPLAKKP  614
            S+ VRQSYK+LELP+RLGG GIYRF+GW+ + +L  ++KP
Sbjct  363  SKWVRQSYKWLELPRRLGGVGIYRFEGWVTDTRLSPSQKP  402


>MAG: putative RNA-dependent RNA polymerase, partial [Totiviridae sp.]
Sequence ID: UHS72532.1 Length: 549
Range 1: 41 to 549

Score:400 bits(1029), Expect:9e-132,
Method:Compositional matrix adjust.,
Identities:208/517(40%), Positives:306/517(59%), Gaps:9/517(1%)

Query  65   PRSNADLVIRRIRLIDLWRNLKPDQMDEIR-NYTHLDYIFVQNICIYMLVFGINTVKHFR  123
            PR   DL IRR+R+ DL R+   D       N    D I V N+  + +V+G+   K
Sbjct  41   PRVKGDLAIRRMRIQDLKRSCPVDVWTYFEENMCMHDVITVTNLMAWTMVWGLQKFKDLH  100

Query  124  QIGLFNERNEFIEIAKQLSTKGKRFVDDVDNMKQKVCEIATIVGYMDPNVDKIDVMEEVN  183
            + G+ N+      +   LS+  KRF +DV N+K+ + E   + GY+  +    +  ++
Sbjct  101  ETGMLNDLETLRTVGSSLSSYVKRFPNDVGNVKKMLAEFNCLTGYLQNDPADFNFADDFE  160

Query  184  SLAAEGNEHGIDRDNWNDLFTKTCKEVMTWYKGHEFISFDDYIKEGMWLTSGSSSIGKVH  243
            +LA  GN++  D++     FT     +MT     E++ F+DYIK+GMW+TSG+SSIGKV+
Sbjct  161  ALATGGNKND-DKET----FTIYMNRIMTPQPMPEYVGFEDYIKQGMWITSGASSIGKVN  215

Query  244  WTKDGENGKFKARKNMLLQIYTPQELANIVYAWDGKLHSRVFIKNEMSKLRLAVASNIEA  303
            W  DG++GKFKARKNM+L +YT  E+  +   WDGKL + VF K+E+SK RLAVASN+ A
Sbjct  216  WEYDGDSGKFKARKNMVLDLYTADEIYELAVQWDGKLENSVFTKDELSKRRLAVASNLAA  275

Query  304  YIHESYMLFLYGHGFKEYFGVTLDEKPDQQHQREIEMIEKLQAGYFGLPFDYASFDHQPT  363
            Y+++S+++ L GHGFK +  VTLDE+P++   R  ++   L++G F LP+DY  FD QP
Sbjct  276  YLNQSWIMRLLGHGFKNWNYVTLDERPNEGFDRTCKIQNLLRSGSFALPWDYKGFDRQPN  335

Query  364  TFEVKTMVRRVGEIVVSQVPKNYYYQTQLLVNKIVNAYDKSYLSGNIKNTKFENIKVKGG  423
            T+EV+ +  +VG +    VP    +   ++  K+  +Y+ +YL    ++ +    K  GG
Sbjct  336  TWEVQLISEKVGMLCRGVVPTADKHMFDVVNAKVNASYEHNYLFAKEQDLR---AKQTGG  392

Query  424  VPSGVRITSLLGNMWNAIITKIAINNVIGIIGYDPISQISLRGDDVAILSKDPAALYLLR  483
            +PSG+R TS +GN+WNAI T+  ++    + G + I  + LRGDD  I+SK   AL   R
Sbjct  393  IPSGIRPTSFIGNIWNAIKTEQVVDMCETVFGKNIIQTVGLRGDDTYIVSKSAVALLFFR  452

Query  484  LSYAAINAIGKDSKLGISPKVCEFLRNEISVTGVRGWTCRGIGGISQRKPWNPQPWSPND  543
              YA+++ IG +SK GI    CEFLR  I+   V GW  R I  + QRK WN QPW P
Sbjct  453  YGYASLDVIGNNSKFGIMQNCCEFLRQNITKDEVHGWPNRAIPSVFQRKSWNLQPWKPGS  512

Query  544  EVETNASNISLLERRAGIELQQLHHINKVKWSRHVRQ  580
            +V T  +NI LLERR G     +H  NK+KWS+ V Q
Sbjct  513  DVTTTTNNIYLLERRLGFACPDIHKANKLKWSKFVGQ  549


>MAG: putative RNA-dependent RNA polymerase, partial [Totiviridae sp.]
Sequence ID: UHS72560.1 Length: 371
Range 1: 1 to 370

Score:391 bits(1004), Expect:1e-130,
Method:Compositional matrix adjust.,
Identities:199/379(53%), Positives:258/379(68%), Gaps:15/379(3%)

Query  249  ENGKFKARKNMLLQIYTPQELANIVYAWDGKLHSRVFIKNEMSKLRLAVASNIEAYIHES  308
            E+GKFKARKNML ++Y+  EL +IV  WDG L SRVF K+E+SK RLAVASNIEAY+ ES
Sbjct  1    ESGKFKARKNMLTELYSDDELISIVRKWDGVLRSRVFTKDELSKRRLAVASNIEAYLTES  60

Query  309  YMLFLYGHGFKEYFGVTLDEKPDQQHQREIEMIEKLQAGYFGLPFDYASFDHQPT----T  364
            ++L ++GHGFK Y  +TLDE P +QHQR   ++  ++ G F LPFD+  FDHQP
Sbjct  61   WILHMFGHGFKNYDYITLDETPKEQHQRSCGVMRLIEGGAFCLPFDFKGFDHQPQIENGV  120

Query  365  FEVKTMVRRVGEIV-VSQVPKNYYYQTQLLVNKIVNAYDKSYLSGNIKNTK-FENIKVKG  422
              +  +V RV EIV V Q+P      ++++         KSY  G + N K   +I  +G
Sbjct  121  QPMDMVVDRVREIVPVDQLPDYDRLSSRMV---------KSYAKGELINPKTMRSIIQEG  171

Query  423  GVPSGVRITSLLGNMWNAIITKIAINNVIGIIGYDPISQISLRGDDVAILSKDPAALYLL  482
            G+PSGVR TSL+GN+WNA +T   +N    ++G D + +I ++GDD  I+S +P  L +
Sbjct  172  GIPSGVRTTSLIGNIWNATMTSKLVNLTRLVMGEDLVKKIGIKGDDTYIISDNPICLVVF  231

Query  483  RLSYAAINAIGKDSKLGISPKVCEFLRNEISVTGVRGWTCRGIGGISQRKPWNPQPWSPN  542
            RL+YAAINAIG DSK GIS +VCEFLR EIS+ G+RGWT R I  I+QRKPWN +PWSP+
Sbjct  232  RLAYAAINAIGSDSKFGISQRVCEFLRTEISMNGMRGWTNRAIPSITQRKPWNSKPWSPS  291

Query  543  DEVETNASNISLLERRAGIELQQLHHINKVKWSRHVRQSYKYLELPKRLGGFGIYRFQGW  602
             EV T A NI LLERRA   L  LH  NK+KWS+   QSY +L LP RLGG G+YRF+GW
Sbjct  292  SEVGTVADNIYLLERRAKKNLDILHLANKIKWSKFTNQSYHWLHLPVRLGGLGLYRFEGW  351

Query  603  LPNGKLPLAKKPLVNVEDI  621
             PNG+LP   KP + V++I
Sbjct  352  EPNGRLPPVTKPHIVVQNI  370


>MAG: putative RNA-dependent RNA polymerase, partial [Totiviridae sp.]
Sequence ID: UHS72546.1 Length: 416
Range 1: 1 to 412

Score:368 bits(945), Expect:4e-121,
Method:Compositional matrix adjust.,
Identities:188/415(45%), Positives:270/415(65%), Gaps:5/415(1%)

Query  241  KVHWTKDGENGKFKARKNMLLQIYTPQELANIVYAWDGKLHSRVFIKNEMSKLRLAVASN  300
            KV+W    E+G FKARKNM+  +YT  EL  +   WDG+L SR F K+E+SK R+AVASN
Sbjct  1    KVYWETGTEHGHFKARKNMVTYLYTTDELYELCKRWDGRLVSRAFTKDELSKRRIAVASN  60

Query  301  IEAYIHESYMLFLYGHGFKEYFGVTLDEKPDQQHQREIEMIEKLQAGYFGLPFDYASFDH  360
            IE+Y+ ESY+L+   H +K +  +TLDE P  +H R IE+ + L++G + LPFD+ +FDH
Sbjct  61   IESYLCESYLLYNMTHVYKSWNYITLDETPAVEHNRTIELSKLLKSGKWALPFDFKNFDH  120

Query  361  QPTTFEVKTMVRRVGEIVVSQVPKNYYYQTQLLVNKIVNAYDKSYLSGNIKNTKFENIKV  420
            QPTT E++T++R   ++   +VP NY  +   +  K++N+Y  + +S NI N + E + V
Sbjct  121  QPTTEEIQTIMRH--DLNGVKVPNNYLNEWNSIKYKVINSYSNNTMSMNIDNKRIE-MDV  177

Query  421  KGGVPSGVRITSLLGNMWNAIITKIAINNVIGIIGYDPISQISLRGDDVAILSKDPAALY  480
            KGG+PSGVR TSL+GN WN ++++ A N    I+GYDP+  + ++GDD  I++     L
Sbjct  178  KGGLPSGVRWTSLIGNQWNMVMSQWAKNITTKILGYDPVLSMGIKGDDTYIIANRAVDLC  237

Query  481  LLRLSYAAINAIGKDSKLGISPKVCEFLRNEISVTGVRGWTCRGIGGISQRKPWNPQPWS  540
            + R +YA INA+G +SK GI  +VCEFLR EIS  G RGWT R I  I+QRKPWN +PWS
Sbjct  238  IFREAYAMINAVGLNSKFGIQKRVCEFLRVEISGDGARGWTNRSIPTITQRKPWNAEPWS  297

Query  541  PNDEVETNASNISLLERRAGIELQQLHHINKVKWSRHVRQSYKYLELPKRLGGFGIYRFQ  600
             +  V   A  I   ERR    L +LH INK +W+RH   S ++LELP+  GGFGIY ++
Sbjct  298  IDSGVSAVAQAICTTERRCFKPLDELHEINKKRWTRHFGLSTRWLELPRLYGGFGIYPWK  357

Query  601  GWLPNGKLPLAK-KPLVNVEDIHPSQEL-FLPLSEQQKKILAQVEMTNKMQTDDI  653
             + P+  L +   +P V  +DI P +EL ++ L+E+++  +A   MT    TDDI
Sbjct  358  YYKPSEPLRIKNDRPSVKYKDIVPYKELTWIKLTEEERHTMAINAMTAATHTDDI  412


>MAG: putative RNA-dependent RNA polymerase, partial [Totiviridae sp.]
Sequence ID: UHS72488.1 Length: 433
Range 1: 1 to 425

Score:367 bits(943), Expect:1e-120,
Method:Compositional matrix adjust.,
Identities:190/429(44%), Positives:267/429(62%), Gaps:8/429(1%)

Query  242  VHWTKDGENGKFKARKNMLLQIYTPQELANIVYAWDGKLHSRVFIKNEMSKLRLAVASNI  301
            V+W  + + GKFKARKNMLL IYT  E+ +IV +WDGK  +   IKNE+ K+RLAVASNI
Sbjct  1    VNWEFERKKGKFKARKNMLLDIYTKDEMWDIVRSWDGKQRTTAIIKNELGKVRLAVASNI  60

Query  302  EAYIHESYMLFLYGHGFKEYFGVTLDEKPDQQHQREIEMIEKLQAGYFGLPFDYASFDHQ  361
            E YI+ESY+L   GHG+  + G+TLDE P    +R     +  + G + LPFDYA FDHQ
Sbjct  61   ELYIYESYLLRHSGHGYTSWTGITLDESPQDNIRRVATTAKFFRRGSYALPFDYAGFDHQ  120

Query  362  PTTFEVKTMVRRVGEIVVSQVPKNYYYQTQLLVNKIVNAYDKSYLSGNIKNTKFENIKVK  421
             TT EV+ +V+   +     VP         ++ K V AY K  ++G+ +    E   V
Sbjct  121  VTTQEVQWIVKDFFDRGKVTVPAYEIPVYNEIIAKTVEAYAKCTITGS-EGGVTETFDVT  179

Query  422  GGVPSGVRITSLLGNMWNAIITKIAINNVIGIIGYDPISQISLRGDDVAILSKDPAALYL  481
            GG+PSGVR+TSL+GN+WN ++T+ A      I+GYDPI Q++LRGDD  I+   P   +
Sbjct  180  GGLPSGVRVTSLVGNIWNGVMTQKAREIAESILGYDPIQQVALRGDDSLIVCAQPLECFA  239

Query  482  LRLSYAAINAIGKDSKLGISPKVCEFLRNEISVTGVRGWTCRGIGGISQRKPWNPQPWSP  541
            +R+ Y AINAIG  +K GI     EFLR  I+     GW  R I  +SQRKPWNP+PWSP
Sbjct  240  VRVGYQAINAIGHSAKFGICQGEGEFLRTVITGRRNYGWVNRAIPSVSQRKPWNPEPWSP  299

Query  542  NDEVETNASNISLLERRAGIELQQLHHINKVKWSRHVRQSYKYLELPKRLGGFGIYRFQG  601
            N +V+T  SNI+LLERR+ +EL++LH  N   WS     S  +L LP R+GG GIY+F+G
Sbjct  300  NHQVKTIWSNINLLERRSMVELERLHRANTKAWSAVTHLSTHWLSLPTRIGGIGIYKFEG  359

Query  602  WLPNGKLPLAKKPLVNVEDI----HPSQELFLPLSEQQKKILAQVEMTNKMQTDDIPGTQ  657
            W+P+  +    KP + VE +     PS   ++ LS  Q +   ++++  KM TDD+PG Q
Sbjct  360  WVPSSAMKNVNKPNIRVEGLVTVPGPS---WIKLSVTQAEEFHRIQVVKKMITDDVPGVQ  416

Query  658  KLFSKEWIQ  666
             ++++ +++
Sbjct  417  AVYNRAFLE  425


>MAG: putative RNA-dependent RNA polymerase, partial [Totiviridae sp.]
Sequence ID: UHS72468.1 Length: 333
Range 1: 1 to 333

Score:343 bits(879), Expect:1e-112,
Method:Compositional matrix adjust.,
Identities:164/334(49%), Positives:230/334(68%), Gaps:1/334(0%)

Query  199  WNDLFTKTCKEVMTWYKGHEFISFDDYIKEGMWLTSGSSSIGKVHWTKDGENGKFKARKN  258
            W   F +    + T      +ISF+DYIK  +W+TSGSSSIGKV W+ D E+GKFKARKN
Sbjct  1    WKKRFAELMIMIKTPVTHKTYISFEDYIKSPIWITSGSSSIGKVEWSYDKESGKFKARKN  60

Query  259  MLLQIYTPQELANIVYAWDGKLHSRVFIKNEMSKLRLAVASNIEAYIHESYMLFLYGHGF  318
            ML  IYTP+EL  ++  W+G L SR FIK+EM K RLAV+S++++Y+HESY+L+L+GHGF
Sbjct  61   MLYDIYTPEELWLVIKDWNGHLESRAFIKDEMGKRRLAVSSSLQSYLHESYILYLFGHGF  120

Query  319  KEYFGVTLDEKPDQQHQREIEMIEKLQAGYFGLPFDYASFDHQPTTFEVKTMVRRVGEIV  378
            K++ G+TLDE P     R +++++ L  G + LPFDYA FDHQP T EV +++  + E V
Sbjct  121  KDWKGITLDETPRMNQLRNLKLMDLLHEGAYALPFDYARFDHQPQTEEVASIIDNLAEEV  180

Query  379  VSQVPKNYYYQTQLLVNKIVNAYDKSYLSGNIKNTKFENIKVKGGVPSGVRITSLLGNMW  438
             S VP         +VNK++ +Y  S +S ++K  + +   V GG+PSGVR+TSL+GN+W
Sbjct  181  RSIVPGRDMDSYNSIVNKVLKSYHNSTISMDVKGKRVKK-TVTGGIPSGVRVTSLVGNVW  239

Query  439  NAIITKIAINNVIGIIGYDPISQISLRGDDVAILSKDPAALYLLRLSYAAINAIGKDSKL  498
            NAI+T+ A      I+GY+PI  + ++GDD   +++    LY +RLSYAA+ AIGK++K
Sbjct  240  NAIMTEYATRITTSILGYNPILTVGIKGDDTYTVARTAVELYFVRLSYAAVKAIGKNAKF  299

Query  499  GISPKVCEFLRNEISVTGVRGWTCRGIGGISQRK  532
            GI+  VCEFLRNEI+ T V GWT R I  ++QRK
Sbjct  300  GITQHVCEFLRNEITSTTVTGWTNRAIPSLTQRK  333


>MAG: putative RNA-dependent RNA polymerase, partial [Totiviridae sp.]
Sequence ID: UHS72564.1 Length: 364
Range 1: 1 to 364

Score:343 bits(880), Expect:2e-112,
Method:Compositional matrix adjust.,
Identities:174/367(47%), Positives:237/367(64%), Gaps:3/367(0%)

Query  153  DNMKQKVCEIATIVGYMDPNVDKIDVMEEVNSLAAEGNEHGIDRDNWNDLFTKTCKEVMT  212
            D+ K ++CEI  + GY+  ++   D   E  SLA  G EHG   +NW   F +   +VMT
Sbjct  1    DDTKIRLCEINCLTGYLQNDMPGWDFAAEFKSLAEGGYEHGWIDENWERTFKRLVDKVMT  60

Query  213  WYKGHEFISFDDYIKEGMWLTSGSSSIGKVHWTKDGENGKFKARKNMLLQIYTPQELANI  272
                 +F++F++YI EG+WLT+GSSSIGKV W K  E G FKARKNML + YT  EL  +
Sbjct  61   KEPLPDFLTFEEYIAEGLWLTAGSSSIGKVAWEKGEEKGTFKARKNMLTEFYTDIELVAL  120

Query  273  VYAWDGKLHSRVFIKNEMSKLRLAVASNIEAYIHESYMLFLYGHGFKEYFGVTLDEKPDQ  332
            V AWDG L S+VF K+E+SK RLAVASNIEAY+ ES++L+L+GHGFK +  +TLDE P
Sbjct  121  VDAWDGILLSKVFTKDELSKRRLAVASNIEAYLSESWILYLFGHGFKNHAYITLDETPKA  180

Query  333  QHQREIEMIEKLQAGYFGLPFDYASFDHQPTTFEVKTMVRRVGEIVVSQVPKNYYYQTQL  392
            QH+R   +I+ L  G F LPFD+  FDHQPT  EV+T++  +     S+ P  Y  + +
Sbjct  181  QHERTSRLIQHLANGSFCLPFDFKGFDHQPTIPEVQTIISNITNTASSKCPPQYLPRFKK  240

Query  393  LVNKIVNAYDKSYLSGNIKNTKFENIKVKGGVPSGVRITSLLGNMWNAIITKIAINNVIG  452
            +  ++VN+Y    +   I     E +   GG+PSGVR TSL+GN WNAI+T
Sbjct  241  MAARMVNSYAHGII---INPMTKERLHQTGGIPSGVRPTSLIGNEWNAIMTTRCREITRL  297

Query  453  IIGYDPISQISLRGDDVAILSKDPAALYLLRLSYAAINAIGKDSKLGISPKVCEFLRNEI  512
            I+  D I +I ++GDD  ++S++P AL + RL+YA+INAIG DSK GIS  +CEFLRNEI
Sbjct  298  IMKTDMIQEIGIKGDDTYVVSRNPIALIVFRLAYASINAIGLDSKFGISQNICEFLRNEI  357

Query  513  SVTGVRG  519
            +  G +G
Sbjct  358  TPKGSQG  364


>MAG: putative RNA-dependent RNA polymerase, partial [Totiviridae sp.]
Sequence ID: UHS72558.1 Length: 508
Range 1: 33 to 504

Score:347 bits(891), Expect:5e-112,
Method:Compositional matrix adjust.,
Identities:196/477(41%), Positives:282/477(59%), Gaps:10/477(2%)

Query  54   ITDKELQTFYVP-RSNADLVIRRIRLIDLWRNLKPDQMDEIRNYT-HLDYIFVQNICIYM  111
            + D  +    +P R+  D  IRRI+  DL   +  D M++   Y  H+D I+  N+ I+
Sbjct  33   VYDSNVNILSLPFRTKLDSAIRRIKARDL-AMVCQDTMNKCAPYMGHMDQIWATNVIIWA  91

Query  112  LVFGINTVKHFRQIGLFNERNEFIEIAKQLSTKGKRFVDDVDNMKQKVCEIATIVGYMDP  171
             + GI +    +++G+ N    F E + +LS+  KRF  +    K+K  E+  + GY++
Sbjct  92   EIHGIESFMQLKRVGMLNSLTHFTEQSSRLSSWVKRFNTNETANKEKYAELNGLSGYLNN  151

Query  172  NVDKIDVMEEVNSLAAEGNEHGIDRDNWNDLFTKTCKEV-MTWYKGH--EFISFDDYIKE  228
            +++ ID  E+   LA  GN HG+   +W   F    K++ +  Y     EFI+F D+IK
Sbjct  152  DIEPIDWFEKTRELAEGGNPHGLINYDWETEFKDANKQIKLKAYHDTKPEFITFYDFIKS  211

Query  229  GMWLTSGSSSIGKVHWTKDGENGKFKARKNMLLQIYTPQELANIVYAWDGKLHSRVFIKN  288
            G W+TSGSS+ G + W  DGE+GKFKARKNML  IYTP+EL N+   W G   +  F K+
Sbjct  212  GDWITSGSSAKGHIEWEMDGESGKFKARKNMLEYIYTPEELYNLCVKWSGVNENVAFTKD  271

Query  289  EMSKLRLAVASNIEAYIHESYMLFLYGHGFKEYFGVTLDEKPDQQHQREIEMIEKLQAGY  348
            E+SK RLAVAS++E+Y+ +SY L L+GH +K +  VTLDE  ++ H R  +M + L  GY
Sbjct  272  ELSKRRLAVASSLESYLLDSYFLRLFGHPYKNWDYVTLDESAEEAHNRTAKMSQLLSHGY  331

Query  349  FGLPFDYASFDHQPTTFEVKTMVRRVGEIVVSQVPKNYYYQTQLLVNKIVNAYDKSYLSG  408
            + LPFD+ +FDHQ    E+  MV    + +   VP +Y  +   + +KI+++Y  S ++
Sbjct  332  YALPFDFKNFDHQAQQAEIVWMVESNLDSI--SVPISYQNEWTWIKDKIMHSYTHSTITF  389

Query  409  NIKNTKFENIKVKGGVPSGVRITSLLGNMWNAIITKIAINNVIGIIGYDPISQISLRGDD  468
            + +N K    KV GG+PSGVR TSLLGN WNA IT+I  N V  ++G  P S I +RGDD
Sbjct  390  SDQNEK-RVYKVTGGLPSGVRTTSLLGNEWNATITRIVSNLVTKLVGKSP-SLIGVRGDD  447

Query  469  VAILSKDPAALYLLRLSYAAINAIGKDSKLGISPKVCEFLRNEISVTGVRGWTCRGI  525
              I+ K P  + + R  Y AINAIG D K GI+   CEFLRNEI+  G RGW  R I
Sbjct  448  TYIIDKSPILVAITREMYKAINAIGHDEKFGITLGACEFLRNEITSKGSRGWANRTI  504


>MAG: putative RNA-dependent RNA polymerase, partial [Totiviridae sp.]
Sequence ID: UHS72525.1 Length: 340
Range 1: 14 to 339

Score:334 bits(857), Expect:2e-109,
Method:Compositional matrix adjust.,
Identities:166/331(50%), Positives:231/331(69%), Gaps:9/331(2%)

Query  219  FISFDDYIKEGMWLTSGSSSIGKVHWTKDGENGKFKARKNMLLQIYTPQELANIVYAWDG  278
            F++ ++YI  G WLTSGSSSIG V W+   + GKFKARKNM+ ++YTP+EL  +   WDG
Sbjct  14   FMTLEEYIASGKWLTSGSSSIGSVEWSYGDDKGKFKARKNMINELYTPEELIKMAVNWDG  73

Query  279  KLHSRVFIKNEMSKLRLAVASNIEAYIHESYMLFLYGHGFKEYFGVTLDEKPDQQHQREI  338
             + ++VF K+E+SK RLAVASNIE+Y++ESY+ +L GHGFK +  +TLDE P  QH R
Sbjct  74   VIINKVFTKDELSKRRLAVASNIESYLNESYLSYLAGHGFKNWKYMTLDETPAAQHNRMY  133

Query  339  EMIEKLQAGYFGLPFDYASFDHQPTTFEVKTMVRRVGEIVVSQVPKNYYYQTQLLVNKIV  398
            ++ + L+ G F LP+DYA+FDHQPT  EV++++R  GE +  ++P +Y  +   ++ K V
Sbjct  134  KVTQLLKEGSFALPWDYAAFDHQPTRDEVQSIIRTSGEKIAHKIPASYRSRYLAILEKSV  193

Query  399  NAYDKSYLSGNIKNTKFENIKVK--GGVPSGVRITSLLGNMWNAIITKIAINNVIG--II  454
             +Y+ ++L   I + K + IK K  GG+PSG+R T+ +GN+ N  +TK  IN  I   I
Sbjct  194  RSYEHNFL---ILDNKQKIIKEKQTGGIPSGIRRTTYIGNIDN--MTKTDINRDITKIIT  248

Query  455  GYDPISQISLRGDDVAILSKDPAALYLLRLSYAAINAIGKDSKLGISPKVCEFLRNEISV  514
              D I  I ++GDD  I+S+DP ALY+ R+ YAA+N IG DSK GISP +CEFLR E+
Sbjct  249  KTDLIQTIKVKGDDTYIISRDPIALYIFRVVYAALNVIGLDSKFGISPNICEFLRVEMRP  308

Query  515  TGVRGWTCRGIGGISQRKPWNPQPWSPNDEV  545
               +GWTCR I GI+QRKPWNPQPWSP+ +V
Sbjct  309  DRCQGWTCRSIPGITQRKPWNPQPWSPSADV  339


>MAG: putative RNA-dependent RNA polymerase, partial [Totiviridae sp.]
Sequence ID: UHS72522.1 Length: 417
Range 1: 27 to 402

Score:334 bits(856), Expect:6e-108,
Method:Compositional matrix adjust.,
Identities:170/383(44%), Positives:248/383(64%), Gaps:9/383(2%)

Query  66   RSNADLVIRRIRLIDLWRNLKPDQMDEIRN-YTHLDYIFVQNICIYMLVFGINTVKHFRQ  124
            R+  DL I R+++ ++      + ++ + N   HLD I V N+ I+  ++G+  +K
Sbjct  27   RTKGDLAIARLKIKNVIIKTDNNIVEYLNNNLCHLDQILVTNLMIWTQMWGLEILKAIVA  86

Query  125  IGLFNERNEFIEIAKQLSTKGKRFVDDVDNMKQKVCEIATIVGYMDPNVDKIDVMEEVNS  184
             GL N    FI+ A +LS+  KRF  D D+ KQ+  E   + GY+  ++D  D  +E +
Sbjct  87   TGLLNSFETFIKYASKLSSYVKRFPLDKDDCKQRFSEFNCLSGYLQNDIDTFDYDKEFDD  146

Query  185  LAAEGNEHGIDRDNWNDLFTKTCKEVMTWYKGHEFISFDDYIKEGMWLTSGSSSIGKVHW  244
            LA  G  H    D+W  LF+    E+MT      F++F++Y+K+G+WLTSGSSS+GKV+W
Sbjct  147  LATGGRLHD---DDWEYLFSSKVYEIMTKQDKPNFVTFEEYVKKGLWLTSGSSSLGKVNW  203

Query  245  TKDGENGKFKARKNMLLQIYTPQELANIVYAWDGKLHSRVFIKNEMSKLRLAVASNIEAY  304
            + D + GKFKARKNML+ +YTP E+  +   WDG L +RVFIK+E+SK RLAVASNIEAY
Sbjct  204  SYDNDAGKFKARKNMLMDLYTPDEIYRLALDWDGTLQNRVFIKDELSKRRLAVASNIEAY  263

Query  305  IHESYMLFLYGHGFKEYFGVTLDEKPDQQHQREIEMIEKLQAGYFGLPFDYASFDHQPTT  364
            +++ Y+L+L+GHGFK Y  +TLDEKP++QH+R IE I  LQAGYF LPFD+  FD QPT
Sbjct  264  LNQGYILYLFGHGFKNYKYITLDEKPNEQHKRNIETIALLQAGYFALPFDFKGFDRQPTL  323

Query  365  FEVKTMVRRVGEIVVSQVPKNYYYQTQLLVNKIVNAYDKSYL-SGNIKNTKFENIKVKGG  423
             E+K ++ R+G++ +  +P +       L  K+VN++D  +L S   K TK    K  GG
Sbjct  324  EEIKLIMSRLGDLTLLDIPPDQSSFVSALSRKVVNSFDNCFLYSPLTKQTK----KQSGG  379

Query  424  VPSGVRITSLLGNMWNAIITKIA  446
            + SG+R TSL+GN+WN+I+T IA
Sbjct  380  LSSGIRPTSLIGNIWNSIVTDIA  402


>MAG: putative RNA-dependent RNA polymerase, partial [Totiviridae sp.]
Sequence ID: UHS72417.1 Length: 494
Range 1: 22 to 494

Score:337 bits(863), Expect:6e-108,
Method:Compositional matrix adjust.,
Identities:192/483(40%), Positives:281/483(58%), Gaps:20/483(4%)

Query  51   PKIITDKELQTFYVP-RSNADLVIRRIRLIDLWRNLKPDQMDEIRNYT----HLDYIFVQ  105
            P + T +      +P R+  D  IRR++    +R+++P    E+++      H D I+
Sbjct  22   PDLTTCQPTTIHDLPFRTKMDAAIRRLK----YRDIEPFLSAEVKSIIPWLHHTDQIWAT  77

Query  106  NICIYMLVFGINTVKHFRQIGLFNERNEFIEIAKQLSTKGKRFVDDVDNMKQKVCEIATI  165
            NI I+  + G+ +    ++ G FN    F++ +  LS   KRF    D  KQK  E+ ++
Sbjct  78   NILIWTQIHGVPSFMELKRYGFFNSLETFLDQSSILSAWVKRFGVQEDTNKQKYAELNSL  137

Query  166  VGYMDPNVDKIDVMEEVNSLAAEGNEHGIDRDNWNDLFTKTCKEV---MTWYKGHEFISF  222
             GYM  ++   D + E+ SLA  G  HG+   +W   F  + + +       +  +FISF
Sbjct  138  SGYMTSDLVDTDWLAELKSLAHGGKPHGLIGSDWKRDFITSNQHIKLPAIHDRRPKFISF  197

Query  223  DDYIKEGMWLTSGSSSIGKVHWTKDGENGKFKARKNMLLQIYTPQELANIVYAWDGKLHS  282
             DY++ G WLT+GSSSIG VHW    ENG F+ARKNML  ++T +EL ++V +WDG   +
Sbjct  198  QDYVESGQWLTAGSSSIGIVHWEAGEENGTFRARKNMLKYLFTAKELIDLVLSWDGVSRN  257

Query  283  RVFIKNEMSKLRLAVASNIEAYIHESYMLFLYGHGFKEYFGVTLDEKPDQQHQREIEMIE  342
            + F K+E+ K RLAVASNIE Y+ ESY L LYGH +K +  VTLDE P Q H R  ++
Sbjct  258  KAFTKDELGKRRLAVASNIEGYLCESYFLALYGHPYKNWKYVTLDETPGQSHNRTSDISN  317

Query  343  KLQAGYFGLPFDYASFDHQPTTFEVKTMVRR-VGEIVVSQVPKNYYYQTQLLVNKIVNAY  401
             L+ G + LPFD+ +FDHQP+ +E+  MV+  + EI    VP  +  Q  ++  KI+ +Y
Sbjct  318  HLKNGAYALPFDFKNFDHQPSQWEISYMVQSNLDEI---SVPCGHRLQWDIIKQKILKSY  374

Query  402  DKSYLSGNIKN-TKFENIKVKGGVPSGVRITSLLGNMWNAIITKIAINNVIGIIGYDPIS  460
                ++  I N T  E I   GG+PSGVR+TSL+GN WNAI+T + +     I    P++
Sbjct  375  AHGQITMKIGNGTATETI--TGGLPSGVRMTSLIGNQWNAIVTNLVLTYTQEITDSVPLA  432

Query  461  QISLRGDDVAILSKDPAALYLLRLSYAAINAIGKDSKLGISPKVCEFLRNEISVTGVRGW  520
             + +RGDD  I+ + P  L ++R  Y ++NAIG D+K GI   V EFLRNEIS TG RGW
Sbjct  433  -VGVRGDDTYIVHESPIYLAIVREVYKSVNAIGIDAKFGICHGVXEFLRNEISPTGQRGW  491

Query  521  TCR  523
            T R
Sbjct  492  TNR  494


>MAG: putative RNA-dependent RNA polymerase, partial [Totiviridae sp.]
Sequence ID: UHS72432.1 Length: 426
Range 1: 9 to 426

Score:326 bits(836), Expect:6e-105,
Method:Compositional matrix adjust.,
Identities:175/427(41%), Positives:254/427(59%), Gaps:15/427(3%)

Query  418  IKVKGGVPSGVRITSLLGNMWNAIITKIAINNVIGIIGYDPISQISLRGDDVAILSKDPA  477
            +K  GG+PSG+R TSL+GN+WN I T IA +    I+G D I +I+L+GDD  I++ +
Sbjct  9    VKQTGGLPSGIRPTSLIGNLWNMIATDIARDVTKDILGQDYIQEIALKGDDTYIVAPNLF  68

Query  478  ALYLLRLSYAAINAIGKDSKLGISPKVCEFLRNEISVTGVRGWTCRGIGGISQRKPWNPQ  537
               + R +Y +INA+G++SK GI    CEFLR EIS TGVRGWT R I  ++QRKPWNP+
Sbjct  69   VCLVFRYAYQSINAVGENSKFGIMQNACEFLRTEISSTGVRGWTNRAIPSVTQRKPWNPE  128

Query  538  PWSPNDEVETNASNISLLERRAGIELQQLHHINKVKWSRHVRQSYKYLELPKRLGGFGIY  597
            PW+ N +V+T A+NI LLERR   +   LH  NK+KWS+ + QSY +L LPK  GGFGIY
Sbjct  129  PWTENQQVQTIANNIYLLERRCKQDCSFLHMANKIKWSKMMHQSYLWLHLPKHHGGFGIY  188

Query  598  RFQGWLPNGKLPLAKKPLVNVEDIHPSQE--LFLPLSEQQKKILAQVEMTNKMQTDDIPG  655
             + GWLPN KLPL + P+ +V +++PS     +  LS+ +K+   +VE ++K+  +DIPG
Sbjct  189  EWSGWLPNCKLPLTQPPVFDVANLNPSSVDLSWFSLSDSEKRAYQKVEFSSKIAANDIPG  248

Query  656  TQKLFSKEWIQKVRAKKIIWSRN----QTIPIHTDHTVRIPKWDEKIKFPRYKSEYILNN  711
              K   + +I  +RA K  WS+     + IP+ +  T   P W      PR K++   N
Sbjct  249  PAKHVMRSYIVALRACKPEWSKTLVNFKPIPLISGPTYTNPFWPH----PRIKTDTTSNT  304

Query  712  KINLTMEQVLRQYNLLKEVERHDKDLKVPKLLDILDKWFPVQSSKIKTYESQGFHRTDAI  771
                 + + +RQ+ + K  +     + VP L D++ K +P     +   E QG+HRTD+I
Sbjct  305  NGYPQLSEFVRQHQIAKRAK-----IPVPPLRDMIAKHYPSAYRVMNRCELQGWHRTDSI  359

Query  772  NLAVGEIPTEPAVKINPILINFVKLHLERQGITHQRGRNKIAKFIYQKTKQAENMILQSS  831
            N+A G+IPTEP   +NPIL  +V+  +   G+    GR  IA  +Y  T  A + I
Sbjct  360  NIACGKIPTEPLKILNPILAAWVQQIVLNAGLLKWSGRKNIANRLYAATTLAVHHIQCEG  419

Query  832  LQQMYRY  838
               MY +
Sbjct  420  GASMYAF  426


>MAG: putative RNA-dependent RNA polymerase, partial [Totiviridae sp.]
Sequence ID: UHS72538.1 Length: 478
Range 1: 18 to 478

Score:302 bits(773), Expect:4e-95,
Method:Compositional matrix adjust.,
Identities:181/477(38%), Positives:276/477(57%), Gaps:23/477(4%)

Query  52   KIITDKELQTFYVPRSNADLVIRRIRLIDLWRNLKPDQMDEIRNYTHLDYIFVQNICIYM  111
            K+  D E  +F   R  AD+  RR++L +L     P   + I    HLD I V N+ I+
Sbjct  18   KVNVDVETISF---RCKADISTRRLKLSNL--TTLPHFQEVIGWTHHLDQILVANLVIWT  72

Query  112  LVFGINTVKHFRQIGLFNERNEFIEIAKQLSTKGKRFVDDVDNMKQKVCEIATIVGYMDP  171
             + GI+     +++G  N   +FI I+  +S+  KRF  D D  KQ   EI ++ GY+
Sbjct  73   YINGIDKFHDLKRLGYLNSITDFINISF-ISSYVKRFPTD-DGSKQIFAEINSLTGYLQN  130

Query  172  NVDKIDVMEEVNSLAAEGNEHGIDRDN----WNDLFTKTCKEVMTWYKGHEFISFDDYIK  227
            +++++D  E+++ LA  G EHG+  ++    + D+    C       + H FI F DYI+
Sbjct  131  DIEEVDWHEKLSELANGGAEHGLPGEDTIKTFTDILNLICPPTT---RPHLFIGFYDYIE  187

Query  228  EGMWLTSGSSSIGKVHWTKDGENGKFKARKN---MLLQIYTPQELANIVYAWDGKLHSRV  284
             G+WLT+GSSSIGKV+W    +    K  K      +Q+      AN+      + H +
Sbjct  188  SGLWLTAGSSSIGKVYWETTQKRVTSKQGKTWSPTCIQLMNYMICANVGMGGSYREHLQ-  246

Query  285  FIKNEMSKLRLAVASNIEAYIHESYMLFLYGHGFKEYFGVTLDEKPDQQHQREIEMIEKL  344
              +  +SK R+AVASNIE+Y+ ESY+L+   H +K +  +TLDE P  +H R IE+ + L
Sbjct  247  --RTNLSKRRIAVASNIESYLCESYLLYNMTHVYKSWKYITLDESPAVEHNRTIEVSKLL  304

Query  345  QAGYFGLPFDYASFDHQPTTFEVKTMVRRVGEIVVSQVPKNYYYQTQLLVNKIVNAYDKS  404
            ++G + LPFD+ +FDHQPTT E++T++    ++    VPK Y  +   +  K++N+Y  +
Sbjct  305  KSGKWALPFDFKNFDHQPTTEEIQTIMSH--DLNGVDVPKQYLQEWNNIKYKVINSYSNN  362

Query  405  YLSGNIKNTKFENIKVKGGVPSGVRITSLLGNMWNAIITKIAINNVIGIIGYDPISQISL  464
             +S +I N K E + VKGG+PSGVR TSL+GN WN ++++ A N    I+GYDP+  + +
Sbjct  363  TMSMSINNKKVE-LDVKGGLPSGVRWTSLIGNQWNMVMSQWARNITTKILGYDPVLTMGI  421

Query  465  RGDDVAILSKDPAALYLLRLSYAAINAIGKDSKLGISPKVCEFLRNEISVTGVRGWT  521
            +GDD  I++     L + R +YA INAIG +SK GI  +VCEFLR EIS    RGWT
Sbjct  422  KGDDTYIIASRAVDLCIFREAYAMINAIGLNSKFGIQKRVCEFLRVEISGDRARGWT  478


>MAG: putative RNA-dependent RNA polymerase, partial [Totiviridae sp.]
Sequence ID: UHS72415.1 Length: 465
Range 1: 46 to 465

Score:285 bits(729), Expect:7e-89,
Method:Compositional matrix adjust.,
Identities:163/426(38%), Positives:245/426(57%), Gaps:11/426(2%)

Query  66   RSNADLVIRRIRLIDLWRNLKPDQMDEIRNYT-HLDYIFVQNICIYMLVFGINTVKHFRQ  124
            R+  D  IRRI+  DL   +  D M +   Y  H+D ++  N+ I+  + G  +    ++
Sbjct  46   RTKLDSAIRRIKARDL-NMVCRDTMAKCAPYMGHMDQVWATNLVIWTEIHGFESFMTLKR  104

Query  125  IGLFNERNEFIEIAKQLSTKGKRFVDDVDNMKQKVCEIATIVGYMDPNVDKIDVMEEVNS  184
            +G+ N    F E + +LS+  KRF  +    K+K  E+  + GY++ +++ +D  E+
Sbjct  105  LGMLNSLTHFTEQSSRLSSWIKRFGTNEIANKEKYAELNGLSGYLNNDLEPVDWFEKTRE  164

Query  185  LAAEGNEHGIDRDNWNDLFTKTCKEVMTWYKGHE----FISFDDYIKEGMWLTSGSSSIG  240
            LA  GN HG+   +W   F    K++      H+    FI+  DYIK G W+TSGSS+ G
Sbjct  165  LAEGGNPHGLIGCDWKTEFMNANKQIKL-RASHDTKPDFITLFDYIKSGDWITSGSSAEG  223

Query  241  KVHWTKDGENGKFKARKNMLLQIYTPQELANIVYAWDGKLHSRVFIKNEMSKLRLAVASN  300
             + W  +G++GKFKARKNML  IYTP+EL N+   W+G   +  F K+E+ K RLAVAS+
Sbjct  224  HIEWEIEGQSGKFKARKNMLELIYTPEELYNLCVKWNGVNDNNAFTKDELGKRRLAVASS  283

Query  301  IEAYIHESYMLFLYGHGFKEYFGVTLDEKPDQQHQREIEMIEKLQAGYFGLPFDYASFDH  360
            +E+Y+ +SY L L+GH +K +  VTLDE  +Q H R  +M + L  GY+ LPFD+ +FDH
Sbjct  284  LESYLMDSYFLRLFGHPYKNWDYVTLDETTEQTHNRTAKMSQLLSDGYYALPFDFKNFDH  343

Query  361  QPTTFEVKTMVRRVGEIVVSQVPKNYYYQTQLLVNKIVNAYDKSYLSGNIKNTKFENIKV  420
            Q    E+  M++   + +   VP  Y  +   + NKI+ +Y  S ++ + +N +    KV
Sbjct  344  QAQQDEIVWMIQSNLDTI--SVPAGYQKEWTWIKNKIMRSYTHSTITYSNQN-ETRVFKV  400

Query  421  KGGVPSGVRITSLLGNMWNAIITKIAINNVIGIIGYDPISQISLRGDDVAILSKDPAALY  480
             GG+PSGVR TSLLGN WNA IT I  N V  ++G  P + I +RGDD  I+ K P  L
Sbjct  401  TGGLPSGVRTTSLLGNEWNATITCIVSNTVARLVGKTP-TMIGVRGDDTYIIDKSPVLLA  459

Query  481  LLRLSY  486
            + R  Y
Sbjct  460  ITREIY  465


>MAG: putative RNA-dependent RNA polymerase, partial [Totiviridae sp.]
Sequence ID: UHS72416.1 Length: 352
Range 1: 46 to 344

Score:278 bits(712), Expect:9e-88,
Method:Compositional matrix adjust.,
Identities:138/300(46%), Positives:195/300(65%), Gaps:2/300(0%)

Query  64   VPRSNADLVIRRIRLIDLWRNLKPDQMDEIRNYTHLDYIFVQNICIYMLVFGINTVKHFR  123
            +PR+N DL IRR+R+ DL R LK +++DE+R + HLD I   NIC++ +V G   ++  R
Sbjct  46   LPRTNGDLAIRRLRIKDLMRVLKKEEIDEMRFFAHLDNIMFANICVFGIVNGFGKLRDLR  105

Query  124  QIGLFNERNEFIEIAKQLSTKGKRFVDDVDNMKQKVCEIATIVGYMDPNVDKIDVMEEVN  183
            + G+FN  + F+E A ++S+  KR+   +D+ K  +CEI  + GY+  +    D   E +
Sbjct  106  ECGIFNSFDIFVEAASRISSHFKRYPVCLDDTKMMLCEINCLTGYLQNDPPGWDFEREFD  165

Query  184  SLAAEGNEHGIDRDNWNDLFTKTCKEVMTWYKGHEFISFDDYIKEGMWLTSGSSSIGKVH  243
            +L   G EHG+   +W   F    K VMT    + ++S DDYI+ G+W+T+G+SSIGKV
Sbjct  166  ALGKGGYEHGLLNSDWKKDFVGALKRVMTRQSINNWVSLDDYIRSGLWITAGASSIGKVE  225

Query  244  WTKDGENGKFKARKNMLLQIYTPQELANIVYAWDGKLHSRVFIKNEMSKLRLAVASNIEA  303
            W+  GE+GKFKARKNML ++Y+  EL +IV  WDG L SRVF K+E+SK RLAVASNIEA
Sbjct  226  WSYSGESGKFKARKNMLTELYSDDELISIVRKWDGVLRSRVFTKDELSKRRLAVASNIEA  285

Query  304  YIHESYMLFLYGHGFKEYFGVTLDEKPDQQHQREIEMIEKLQAGYFGLPFDY-ASFDHQP  362
            Y+ ES++L ++GHGFK Y  +TLDE P +QHQR     E  + G     F +   FDHQP
Sbjct  286  YLTESWILHMFGHGFKNYDYITLDETPKEQHQRSCGN-ETDRGGSILFTFRFQGVFDHQP  344


>MAG: putative RNA-dependent RNA polymerase, partial [Totiviridae sp.]
Sequence ID: UHS72413.1 Length: 378
Range 1: 7 to 378

Score:272 bits(695), Expect:6e-85,
Method:Compositional matrix adjust.,
Identities:149/381(39%), Positives:219/381(57%), Gaps:16/381(4%)

Query  396  KIVNAYDKSYLSGNIKNTKFENIKVKGGVPSGVRITSLLGNMWNAIITKIAINNVIGIIG  455
            +++ +Y  S +  NIK+   +  +V GG+PSGVR+TSLLGN WN  IT+I  N    ++G
Sbjct  7    RVLRSYANSEVLMNIKDKTVKQ-EVTGGLPSGVRLTSLLGNQWNVAITQIVRNLATELVG  65

Query  456  YDPISQISLRGDDVAILSKDPAALYLLRLSYAAINAIGKDSKLGISPKVCEFLRNEISVT  515
            YD    I ++GDD  ++S  P  LY  RL Y A+NA+G D+K  I+P  CEFLRNE+S T
Sbjct  66   YDMTETIGIKGDDTYVISHSPGFLYAFRLCYEAVNAVGIDTKFAITPTNCEFLRNEVSST  125

Query  516  GVRGWTCRGIGGISQRKPWNPQPWSPNDEVETNASNISLLERRAGIELQQLHHINKVKWS  575
             V+GW+ R I  +SQRKPWNP PW P  +V T A NI  +ERR G  L +LH  NK+KWS
Sbjct  126  SVKGWSNRSIPSLSQRKPWNPAPWRPERDVTTIAENIWTIERRFGSPLPRLHSANKIKWS  185

Query  576  RHVRQSYKYLELPKRLGGFGIYRFQGWLPNGKLPLAKKPLVNVEDIHPSQE------LFL  629
            ++  Q  K+L LP RLGG+GIY ++GW+   KLP    P +   DI P          ++
Sbjct  186  KYTNQDQKWLHLPIRLGGYGIYPWKGWITTTKLPKLTHPTIRF-DIPPDDNNAVKYPKWI  244

Query  630  PLSEQQKKILAQVEMTNKMQTDDIPGTQKLFSKEWIQKVRAKKIIWSR-NQTIPIHTDHT  688
             ++ Q+K   +Q  M+  + +DD+PG Q  FS ++I+++R  +  W   +    + T
Sbjct  245  DMTPQEKDDYSQALMSAMVASDDVPGLQSEFSGQYIKELRKLRPKWELISIRWSLKTLSR  304

Query  689  VRIPKWDEKIKFPRYKSEYILNNKINLTMEQVLRQYNLLKEVERHDKDLKVPKLLDILDK  748
            +  P  D    +P+       NN ++L + + L Q N+++        LK       L+K
Sbjct  305  LPYPTKDYSKIWPKDHVILERNNTLDLNVWEFLHQVNIVQR-------LKSISTAVYLEK  357

Query  749  WFPVQSSKIKTYESQGFHRTD  769
            +FP   S++K YE  G+HRTD
Sbjct  358  FFPSTYSQMKAYEKSGWHRTD  378


>MAG: putative RNA-dependent RNA polymerase, partial [Totiviridae sp.]
Sequence ID: UHS72556.1 Length: 330
Range 1: 12 to 330

Score:262 bits(669), Expect:7e-82,
Method:Compositional matrix adjust.,
Identities:137/327(42%), Positives:201/327(61%), Gaps:8/327(2%)

Query  42   TDKTKYVNVPKIITDKELQTFYVPRSNADLVIRRIRLIDLWRNLKPDQMDEIRNYTHLDY  101
             D TKY  V     D+     + PR NADL +RR++L D++  L  D +  I   THLD
Sbjct  12   ADGTKYSEVSFSTLDE----VFTPRCNADLAVRRLKLKDMYNYLDIDCLHWI---THLDV  64

Query  102  IFVQNICIYMLVFGINTVKHFRQIGLFNERNEFIEIAKQLSTKGKRFVDDVDNMKQKVCE  161
            I   NI I+  ++G +     R+ G+FN      E   +LS+  KRF  D D  K    E
Sbjct  65   ITATNILIWSNLWGYDVFTRVRKTGIFNNLINLTEFGSKLSSYVKRFPAD-DGSKAMFAE  123

Query  162  IATIVGYMDPNVDKIDVMEEVNSLAAEGNEHGIDRDNWNDLFTKTCKEVMTWYKGHEFIS  221
            + ++ GY+  ++  +D  +E   L+  GNEHG+  +N  + F +         + H ++S
Sbjct  124  LGSLTGYLQSDITPVDWKKEFVDLSEGGNEHGLIGENIGNTFQRITNIYCPVKQLHPYMS  183

Query  222  FDDYIKEGMWLTSGSSSIGKVHWTKDGENGKFKARKNMLLQIYTPQELANIVYAWDGKLH  281
              +YI+ G WLTSGSSSIGKV W++D  +G FKARKNML  +YTP+EL  +V  W+GKL
Sbjct  184  MYEYIESGFWLTSGSSSIGKVEWSQDASSGHFKARKNMLTYLYTPEELYKLVKLWNGKLT  243

Query  282  SRVFIKNEMSKLRLAVASNIEAYIHESYMLFLYGHGFKEYFGVTLDEKPDQQHQREIEMI  341
            SRVF K+E+SK RLAV+SNIE+Y+ ES+ L + GH +K++  +TLDE P +QH R +E+
Sbjct  244  SRVFTKDELSKRRLAVSSNIESYLCESWTLAILGHFYKKWNYLTLDESPSRQHYRTLEVA  303

Query  342  EKLQAGYFGLPFDYASFDHQPTTFEVK  368
            ++L++G + LPFD+ +FDHQPT  E++
Sbjct  304  QQLKSGSWALPFDFKAFDHQPTLAEIE  330


>MAG: putative RNA-dependent RNA polymerase, partial [Totiviridae sp.]
Sequence ID: UHS72537.1 Length: 346
Range 1: 5 to 346

Score:259 bits(661), Expect:1e-80,
Method:Compositional matrix adjust.,
Identities:146/347(42%), Positives:207/347(59%), Gaps:9/347(2%)

Query  146  KRFVDDVDNMKQKVCEIATIVGYMDPNVDKIDVMEEVNSLAAEGNEHGIDRDNWNDLFTK  205
            KRF  +    K+K  E+  + GY++ +++ ID  E+   LA  GN HG+   +W   F
Sbjct  5    KRFNTNETANKEKYAELNGLSGYLNNDIEPIDWFEKTRELAEGGNPHGLINCDWETEFKD  64

Query  206  TCKEVMTWYKGH----EFISFDDYIKEGMWLTSGSSSIGKVHWTKDGENGKFKARKNMLL  261
              K++      H    EF +F D+IK G W+TSGSS+ G + W  +GE+GKFKARKNML
Sbjct  65   ANKQIKL-KASHDTKPEFTTFYDFIKSGDWITSGSSAKGHIEWEMEGESGKFKARKNMLE  123

Query  262  QIYTPQELANIVYAWDGKLHSRVFIKNEMSKLRLAVASNIEAYIHESYMLFLYGHGFKEY  321
             IYTP+EL N+   WDG   +  F K+E+SK RLAVAS++E+Y+ +SY L L+GH +K +
Sbjct  124  YIYTPEELYNLCVKWDGVNKNIAFTKDELSKRRLAVASSLESYLLDSYFLRLFGHPYKNW  183

Query  322  FGVTLDEKPDQQHQREIEMIEKLQAGYFGLPFDYASFDHQPTTFEVKTMVRRVGEIVVSQ  381
              VTLDE  ++ H R   M + L  GY+ LPFD+ +FDHQ    E+  MV     +
Sbjct  184  DYVTLDESAEETHNRTTTMSQLLSHGYYALPFDFKNFDHQAQQAEIVWMVE--SNLNSIS  241

Query  382  VPKNYYYQTQLLVNKIVNAYDKSYLSGNIKNTKFENIKVKGGVPSGVRITSLLGNMWNAI  441
            VP  Y  + + + +KI+++Y  S ++ + +N K    KV GG+PSGVR TSLLGN WNA
Sbjct  242  VPIGYQNEWKWIKDKIMHSYTHSTIAFSDQNEK-RVYKVTGGLPSGVRTTSLLGNEWNAT  300

Query  442  ITKIAINNVIGIIGYDPISQISLRGDDVAILSKDPAALYLLRLSYAA  488
            IT+I  N V  ++G  P S I +RGD+  I+ K P  L + R  Y A
Sbjct  301  ITRIVSNLVTKLVGKSP-SLIGVRGDETYIIDKSPILLAITREMYKA  346


>MAG: putative RNA-dependent RNA polymerase, partial [Totiviridae sp.]
Sequence ID: UHS72524.1 Length: 337
Range 1: 4 to 331

Score:254 bits(650), Expect:5e-79,
Method:Compositional matrix adjust.,
Identities:137/337(41%), Positives:211/337(62%), Gaps:9/337(2%)

Query  211  MTWYKGHEFISFDDYIKEGMWLTSGSSSIGKVHWTKDGENGKFKARKNMLLQIYTPQELA  270
            ++WY      +  DYI  G W+TSGSSS+GKV WT   E G+FKARKN +  IYT +EL
Sbjct  4    VSWY------TLYDYIDSGKWITSGSSSVGKVEWTFGDEKGRFKARKNFIPYIYTTEELK  57

Query  271  NIVYAWDGKLHSRVFIKNEMSKLRLAVASNIEAYIHESYMLFLYGHGFKEYFGVTLDEKP  330
             +      K  ++V +KNE  K+RLAVA +I  Y+ ++++ +L GH +K + G TLDE
Sbjct  58   ELCEQSLLKQENKVLVKNEFGKVRLAVAGDIGTYLVQAWLFYLTGHAYKTWKGGTLDETT  117

Query  331  DQQHQREIEMIEKLQAGYFGLPFDYASFDHQPTTFEVKTMVRRVGEIVVSQVPKNYYYQT  390
             +Q +R+ +M +K++  Y  LPFDYA+FDHQ  T+E+K +V    E+  + V      +
Sbjct  118  FEQIRRQEKMRDKMKKAYV-LPFDYAAFDHQVNTWEIKEIVHAYFELGRANVLLEDQREW  176

Query  391  QLLVNKIVNAYDKSYLSGNIKNTKFENIKVKGGVPSGVRITSLLGNMWNAIITKIAINNV  450
            +  ++  V+A+D +YL+ N++  K    KV GG+ SG+RITSL+GN WN ++T+IA + V
Sbjct  177  KRWLDGTVSAFDNAYLTSNLEG-KPVTYKVTGGLMSGLRITSLVGNCWNLVMTRIAESLV  235

Query  451  IGIIGYDPISQISLRGDDVAILSKDPAALYLLRLSYAAINAIGKDSKLGISPKVCEFLRN  510
            +    ++  +  ++RGDD  +++ +  A+ L R +Y AINAIG D+K GI  +  EFLRN
Sbjct  236  VETGLFNAFTN-AIRGDDSVVINDNYIAVLLTRFAYGAINAIGNDAKYGIHYEQAEFLRN  294

Query  511  EISVTGVRGWTCRGIGGISQRKPWNPQPWSPNDEVET  547
             +S   + G   R I G++QRKPW+  PWSP   +++
Sbjct  295  WLSTNAIYGLPNRNIPGLTQRKPWSNDPWSPESVLQS  331


>MAG: putative RNA-dependent RNA polymerase, partial [Totiviridae sp.]
Sequence ID: UHS72528.1 Length: 350
Range 1: 33 to 350

Score:218 bits(554), Expect:3e-65,
Method:Compositional matrix adjust.,
Identities:119/319(37%), Positives:186/319(58%), Gaps:6/319(1%)

Query  54   ITDKELQTFYVP-RSNADLVIRRIRLIDLWRNLKPDQMDEIRNYT-HLDYIFVQNICIYM  111
            I D  +    +P R+  D  IRRI+  DL   +  D +++   Y  H+D I+  N+ I+
Sbjct  33   IYDSSVNILNLPFRTKLDSAIRRIKARDL-AMICQDTINKCAPYMGHMDQIWATNLIIWA  91

Query  112  LVFGINTVKHFRQIGLFNERNEFIEIAKQLSTKGKRFVDDVDNMKQKVCEIATIVGYMDP  171
             + GI +    +++G+ N    F E + +LS+  KRF  +    K+K  E+  + GY++
Sbjct  92   DIHGIESFMQLKRLGMLNSLTHFTEQSSRLSSWVKRFGTNETLNKEKYAELNGLSGYLNN  151

Query  172  NVDKIDVMEEVNSLAAEGNEHGIDRDNWNDLFTKTCKEV---MTWYKGHEFISFDDYIKE  228
            +++ +D  E+   LA  GN HG+   +W   F    K++    +     EFI+F D+IK
Sbjct  152  DLEPVDWFEKTRELAEGGNPHGLINYDWETEFKNANKQIKLEASHDTKPEFITFYDFIKT  211

Query  229  GMWLTSGSSSIGKVHWTKDGENGKFKARKNMLLQIYTPQELANIVYAWDGKLHSRVFIKN  288
            G W+TSGSS+ G + W  +G++GKFKARKNML  IYTP EL ++   W+G   +  F K+
Sbjct  212  GDWITSGSSAQGNIEWEMEGKSGKFKARKNMLEYIYTPDELYDLCVKWNGINENVAFTKD  271

Query  289  EMSKLRLAVASNIEAYIHESYMLFLYGHGFKEYFGVTLDEKPDQQHQREIEMIEKLQAGY  348
            E+ K RLAVAS++E+Y+ +SY L L+GH +K +  VTLDE  ++ H R  +M + L  GY
Sbjct  272  ELGKRRLAVASSLESYLLDSYFLRLFGHPYKNWDYVTLDESAEETHNRTTKMSQLLSQGY  331

Query  349  FGLPFDYASFDHQPTTFEV  367
            + LPFD+ +FDHQ    E+
Sbjct  332  YALPFDFKNFDHQAQQAEI  350


>MAG: RNA-dependent RNA polymerase [Totiviridae sp.]
Sequence ID: WAK77453.1 Length: 960
Range 1: 136 to 912

Score:230 bits(586), Expect:7e-65,
Method:Compositional matrix adjust.,
Identities:220/802(27%), Positives:364/802(45%), Gaps:86/802(10%)

Query  56   DKELQTFYVPRSNADLVIRRIRLIDL--WRNLKPDQMDE---IRNYTHLDYIFVQNICIY  110
            D      + PR+ ADL +R +RL DL  +    P+  D    +R     DY+ V NI +
Sbjct  136  DSLALALFPPRNRADLGVRNVRLADLLAFYRCHPEYGDTPTLLRQLKFFDYVCVCNILLA  195

Query  111  MLVF-GINTVKHFRQIGLFNERNEFIEIA-KQLS--TKGKRFVDDVDNMKQKVCEIATIV  166
             +VF G    +     GL     E   +A K LS   K +R +   D ++  + E+A++
Sbjct  196  RVVFYGTGWWEIVEGSGLLAAGAEHFRLAGKALSDLVKKERVLIPAD-LRTSLYEVASLY  254

Query  167  GYMDPNVDKIDVMEEVNSLAAEG-NEHGI---DRDNWNDLFTKTCK-EVMTWYKGHEFIS  221
            G M P V   D +EE  +LA  G + HG+   +R   + +     +   +      +  S
Sbjct  255  GAMTPPVPGWDPVEETEALAKSGQSRHGLMGFERYGQSAILEAITRLGDIPGPPARKAES  314

Query  222  FDDYIKEGMWLTSGSSSIGKVHW----TKDGENGKFKARKNMLLQIYTPQELANIVYAWD  277
            F  +++  +W  SGSSSIG V +     ++ E G F+ARKN++L I TP+EL  +V   D
Sbjct  315  FRSWLESAVWERSGSSSIGNVEYELSDGRESERGHFRARKNLILDIKTPEELEKLVLDCD  374

Query  278  GKLHSRVFIKNEMSKLRLAVASNIEAYIHESYMLFLYGHGFKEYFGVTLDEKPDQQHQRE  337
             K  +   IK E SK+R+AV+S +  Y+ +S+   L G  +K + G TL+E   Q+  R
Sbjct  375  -KQDNNALIKAEFSKIRIAVSSPLAVYLGQSWFYDLAGTFYKNWPGNTLEETAGQEAFRT  433

Query  338  IEMIEKLQAGYFGLPFDYASFDHQPTTFEV----KTMVRRVGEIVVSQVPKNYYYQTQLL  393
                 KLQ G F LP+D+  FDHQP   E+      +VR+    + +  P +     +
Sbjct  434  ELTFSKLQRGEFALPYDFNRFDHQPRLEEILAFHDVLVRKA---LAAAAPID-----RPT  485

Query  394  VNKIVNAYDKSYLSGNIKNTKF----ENIKVKGGVPSGVRITSLLGNMWNAIITKIAINN  449
            V  I     K +L+  +++       +   V GG+ SG+R TS +G+ WN ++  +A
Sbjct  486  VLHIAEMQRKGFLAATLRSPPGLGDRQTFTVTGGLMSGLRSTSCVGSGWNLVLGTMA---  542

Query  450  VIGIIG-YDPISQ-----ISLRGDDVAILSKDPAALYLLRLSYAAINAIGKDSKLGISPK  503
              GI+G +   +      I +RGDD  +LS +   +  ++L Y A+ A   +SK  +
Sbjct  543  -QGIVGEFRSCASAVRTCIYVRGDDTQVLSPNYHDVLGVKLCYDALGAEANESKFSLRQG  601

Query  504  VCEFLRNEISVTGVRGWTCRGIGGISQRKPWNPQPWSPNDEVETNASNISLLERRAG---  560
              EFLR E+S    RG+ CR +  ++QR+PWN +P  P   V   +  +S L RR
Sbjct  602  RSEFLRIEVS-DRARGYPCRTVPLVTQRRPWNARPVGPEAVVGHVSKVLSTLGRRTANPQ  660

Query  561  -IELQQLHHINKVKWSRHVRQSYKYLELPKRLGGFGIYRFQG-WL----PNGKLP----L  610
             ++  +  HI++V     +    K  ++P   GGFG+  + G WL     +  LP    L
Sbjct  661  HVDAYRRFHIDRVMGLLGLDPRLK--DVPVGAGGFGLEPWSGRWLVTRWRSSLLPPVHVL  718

Query  611  AKKPLVNVEDIHPSQELFLPLSEQQKKILAQVEMTNKMQTDDIPGT-------------Q  657
                  +V+     Q L LP+       + Q   T KM  DDIP               +
Sbjct  719  NATDFRHVQVTRELQRLRLPVRPNIVASIVQARRTGKMALDDIPKLGGVARRLMRAELRE  778

Query  658  KLFSKEWIQKVRAKKIIWSRNQTIPIHTDHTVRIPKWDEKIKFPRYKSEYILNNKINL--  715
            + F    I +  A + + +   T+ +    T +   +   I       E  +  +  L
Sbjct  779  RQFVAADIDRDLAGQGLIAMCPTLAVVHGVTPKPGAYQSLIS----DLEATVGGEAALWG  834

Query  716  TMEQVLRQYNLLKEVERHDKDLKVPKLLDILDKWFPVQSSKIKTYESQ-GFHRTDAINLA  774
            + ++ +   +LL E  RH K      L  +L ++ P+  +K+   E + G  R  A+++
Sbjct  835  SCKREVASISLLAEYARHSKH----SLGQVLRRYRPMIWNKLLATERRYGVRRNVALDIL  890

Query  775  VGEIPTEPAVKINPILINFVKL  796
             G +    +  +NP+L    +L
Sbjct  891  TGSLAVSGSESMNPLLPRVARL  912


>MAG: putative RNA-dependent RNA polymerase, partial [Totiviridae sp.]
Sequence ID: UHS72569.1 Length: 372
Range 1: 29 to 362

Score:216 bits(549), Expect:3e-64,
Method:Compositional matrix adjust.,
Identities:128/348(37%), Positives:197/348(56%), Gaps:17/348(4%)

Query  66   RSNADLVIRRIRLIDLWRNLKPDQMDEIRNYTHLDYIFVQNICIYMLVFGINTVKHFRQI  125
            R NAD   RRI+L +L R   P+        +HLD + + N+ I+  + GI+  +  R+
Sbjct  29   RCNADKSSRRIKLQNLKR--LPEYEHVTSYLSHLDPLLIANLVIWAHINGIDAFEDLRKF  86

Query  126  GLFNERNEFIEIAKQLSTKGKRFVDDVDNMKQKVCEIATIVGYMDPNVDKIDVMEEVNSL  185
            G FN  N+F+E++  +S+  KRF  D D  KQ   EI  + GY+  ++++++  E++  L
Sbjct  87   GFFNSVNDFVELS-SISSFVKRFPSD-DGSKQIFAEIQNLTGYLQSDLEEVNWKEKLEDL  144

Query  186  AAEGNEHGIDRDNWNDLFTKTCKEVMTWYKGHEFISFDDYIKEGMWLTSGSSSIGKVHWT  245
               G +   +   + D+ +  C +  +     E +   DYI  G+WLT+GSSSIGKV+W+
Sbjct  145  VHGGQKSRTNITEFQDVLSLMCPKATSM---REHMGLFDYIDSGLWLTAGSSSIGKVYWS  201

Query  246  KDGENGKFKARKNMLLQIYTPQELANIVYAWDGKLHSRVFIKNEMSKLRLAVASNIEAYI  305
               E G FKARKNM+  +YT   L ++   W+GKL S+ F K+E++K R+AVASNIE+YI
Sbjct  202  LGDEKGHFKARKNMIAYLYTSDYLYDLCKRWNGKLVSKAFTKDELAKRRIAVASNIESYI  261

Query  306  HESYMLFLYGHGFKEYFGVTLDEKPDQQHQREIEMIEKLQAGYFGLPFDYAS--FDHQPT  363
             ES++L+  GH +K +  +TLDE P  +H R +     L    + LPFD+    FDHQP+
Sbjct  262  CESFLLYNMGHVYKSWDYITLDESPGVEHSRTLNAARILGENAWALPFDFKKKLFDHQPS  321

Query  364  TFEVKTMVRR-VGEIVVSQVPKNYYYQTQLLVNKIVNAYDKSYLSGNI  410
             +E+  MV+  + EI    VP  +  Q   +  KI+    KSY  G I
Sbjct  322  QWEISLMVQSNLDEI---SVPPGHRLQWDCIRQKIL----KSYTHGQI  362


>MAG: RNA-dependent RNA polymerase [Totiviridae sp.]
Sequence ID: WAK77419.1 Length: 959
Range 1: 136 to 788

Score:225 bits(573), Expect:4e-63,
Method:Compositional matrix adjust.,
Identities:188/670(28%), Positives:323/670(48%), Gaps:60/670(8%)

Query  56   DKELQTFYVPRSNADLVIRRIRLID-----LWRNLKPDQMDEIRNYTHLDYIFVQNICIY  110
            D   Q  + PR+ AD+ IR +R+ D     L  + +    D +R     DYI   N+ I
Sbjct  136  DTLAQELFPPRNEADMGIRNLRMADMLTYCLATDRRAAVTDVLRLCRGFDYILTCNLLIA  195

Query  111  MLVFGINTVKHFRQIGLFNERNE-FIEIAKQLSTKGKRFVDDVDNMKQKVC--EIATIVG  167
            M  FG       R+ G+F+  +E +    K LS   K+  + + +   +VC  E A + G
Sbjct  196  MFCFGDLWWTGLREAGVFDGDSEHYRTSCKALSDTVKKTRNTLSD-DDRVCFYECAALFG  254

Query  168  YMDPNVDKIDVMEEVNSLAAEG-NEHG--IDRDNWNDLFTKTCKEVMTWYKGH--EFISF  222
             M P V   D +EE   LA+ G  +HG  +  +   D      + ++ + +G   E  +F
Sbjct  255  AMCPPVPGWDPLEETRDLASGGQTDHGLVVGDELGPDAILGRIQALVCFPEGKRCEERTF  314

Query  223  DDYIKEGMWLTSGSSSIGKVHWTKDGEN----GKFKARKNMLLQIYTPQELANIVYAWDG  278
            +++++   W  SGSSSIG+V +  + ++    G FKARKN++L +   QEL N V + D
Sbjct  315  EEWLRSCEWERSGSSSIGRVEYEVEVDDERRRGHFKARKNLVLDVVPFQELVNSVRSHDQ  374

Query  279  KLHSRVFIKNEMSKLRLAVASNIEAYIHESYMLFLYGHGFKEYFGVTLDEKPDQQHQREI  338
            +  ++  +K+E  K+RLAV++ +E Y+ + Y+  + G  + ++ G TL+E   ++ +R
Sbjct  375  Q-ENKALVKSEFGKVRLAVSAPLEVYLQQGYLYGVSGSAYLQWPGNTLEESIGEEMRRNE  433

Query  339  EMIEKLQAGYFGLPFDYASFDHQPTTFEVKTMVRRVGEIVVSQVPKNYYYQTQLLVNKIV  398
                ++  G F LP+D+A FDHQPTT EV T      +  +     +      L    +
Sbjct  434  LTYTRMAGGGFALPYDFARFDHQPTTEEVITFQTITFDRALLNAGPSQRSDVVLFEQLLE  493

Query  399  NAYDKSYLSGNIKNTKFENIKVKGGVPSGVRITSLLGNMWNAIITKIAINNVIGIIGYD-  457
            + +  + L+      +     V GG+ SG+R TS +G+ WN+++ + A +       Y
Sbjct  494  HGFRHATLTTPPGICEPHTFPVTGGLMSGLRSTSAVGSGWNSVLGETARDMAGRFRTYAR  553

Query  458  PIS--QISLRGDDVAILSKDPAALYLLRLSYAAINAIGKDSKLGISPKVCEFLRNEISVT  515
            PI   QI +RGDD  ++S     +  ++L Y A+ A   +SK  +     EFLR E   T
Sbjct  554  PIDTWQI-VRGDDTQVVSDHYLDVLAVKLGYDALGAEANESKFTLRRGRTEFLRVE---T  609

Query  516  G--VRGWTCRGIGGISQRKPWNPQPWSPNDEVETNASNISLLERRAGIELQQLHHINKVK  573
            G   R + CR I  + QRKPW+ +  S +  +       S+L RR       +   + V
Sbjct  610  GDRARAYPCRTIPLLGQRKPWSARSPSADASLSRIDKVFSVLARRLPDPSDLMGFADHVM  669

Query  574  WS--RHVRQSYKYLELPKRLGGFGIYRFQG------WLPNGKLPLAKKPLVNVEDIHPSQ  625
            +     ++  ++ + +P  LGG G+  + G      W  N ++P+  + ++N  D  P+Q
Sbjct  670  YRMMSKMKLDFRLIRIPTGLGGLGLRPWDGRYHVSVW--NSRVPVPVR-VLNQTDFRPAQ  726

Query  626  ------ELFLPLSEQQKKILAQVEMTNKMQTDD---IPGTQKLFSKEWIQKVR----AKK  672
                  +L + +S+ + + LA  E+  KM TDD   + GT        I++VR    A++
Sbjct  727  QRAKYAQLGVRISDGESRALADHEVREKMATDDMVELAGT--------IRRVRRAELARR  778

Query  673  IIWSRNQTIP  682
            +I S   + P
Sbjct  779  VIVSSGCSGP  788


>MAG: putative RNA-dependent RNA polymerase, partial [Totiviridae sp.]
Sequence ID: UHS72475.1 Length: 295
Range 1: 59 to 294

Score:206 bits(523), Expect:2e-61,
Method:Compositional matrix adjust.,
Identities:101/236(43%), Positives:149/236(63%), Gaps:0/236(0%)

Query  54   ITDKELQTFYVPRSNADLVIRRIRLIDLWRNLKPDQMDEIRNYTHLDYIFVQNICIYMLV  113
            +TD EL      RS  D  IRR +  D+W +   +   E+R Y HLD IF  NI I+  +
Sbjct  59   LTDTELLERMPVRSKLDSSIRRFKWRDIWSSAPVELKYEMRYYQHLDIIFSTNIMIWGRL  118

Query  114  FGINTVKHFRQIGLFNERNEFIEIAKQLSTKGKRFVDDVDNMKQKVCEIATIVGYMDPNV  173
            FG + ++  R +GLFN  + F++ A ++S   KRF  D D++KQ++ E++ +VGY+  +
Sbjct  119  FGADALRDLRLLGLFNGLDHFLDRASKISAYVKRFPYDPDDIKQRLAELSGLVGYLTTDT  178

Query  174  DKIDVMEEVNSLAAEGNEHGIDRDNWNDLFTKTCKEVMTWYKGHEFISFDDYIKEGMWLT  233
            +  +  EE+ +LA  GN+HG+  D+W+ LF +  + +       +FI+F++Y+K G W T
Sbjct  179  ESWNTAEELAALAEGGNQHGLITDDWHRLFPQLAELIKNGRSAPKFITFEEYVKSGQWTT  238

Query  234  SGSSSIGKVHWTKDGENGKFKARKNMLLQIYTPQELANIVYAWDGKLHSRVFIKNE  289
            SGS+SIGKV WT D E+G FKA+KNML +IYTP EL  +  AWDG L SR FIK+E
Sbjct  239  SGSASIGKVVWTHDLEHGSFKAKKNMLFEIYTPDELWALATAWDGTLKSRAFIKSE  294


>MAG: RNA-dependent RNA polymerase [Totiviridae sp.]
Sequence ID: WAK77558.1 Length: 938
Range 1: 115 to 767

Score:219 bits(557), Expect:4e-61,
Method:Compositional matrix adjust.,
Identities:192/667(29%), Positives:316/667(47%), Gaps:54/667(8%)

Query  56   DKELQTFYVPRSNADLVIRRIRLIDLWRNLKP----DQMDEIRNYTH-LDYIFVQNICIY  110
            D   Q  + PR+  D+ IR +R+ D +   K      +M E+       DYI   N+ I
Sbjct  115  DSLAQQLFPPRNEEDIGIRNLRMADAFSYAKATGRVQRMREVLVLCKGFDYILTNNLLIA  174

Query  111  MLVFGINTVKHFRQIGLFNERNEFIEIA-KQLSTKGKRFVDDVDNMKQKVC--EIATIVG  167
            +  FG       ++ G+F+  +E   +A K LS K K+  +DVD  K +VC  E A++ G
Sbjct  175  VFCFGEWWWDVLKRAGVFDGDSEHYRLACKALSDKVKKTRNDVDADK-RVCFYECASLYG  233

Query  168  YMDPNVDKIDVMEEVNSLAAEGN-EHGIDRDNW---NDLFTKTCKEV-MTWYKGHEFISF  222
             M P V   D ++E   LA+ G   HG+ R +     D+  +    V  T  K  +  +F
Sbjct  234  AMCPPVPGWDPVQETRDLASGGKVPHGLTRGSVLRPADVLARIQDLVRFTAGKKCDRRTF  293

Query  223  DDYIKEGMWLTSGSSSIGKVHWTKDGEN----GKFKARKNMLLQIYTPQELANIVYAWDG  278
            ++++  G W  SG+SSIG+V +  D E     G FKARKN++L +    +L   V   D
Sbjct  294  EEWLASGDWERSGASSIGRVEYRIDQEGEERKGHFKARKNLVLDVVPVPDLLERVRCHDT  353

Query  279  KLHSRVFIKNEMSKLRLAVASNIEAYIHESYMLFLYGHGFKEYFGVTLDEKPDQQHQREI  338
            +  ++  +K+E  K+RLAV++ +E Y+ + ++  + G  +  + G TL+E   ++  R
Sbjct  354  Q-ENKALVKSEFGKVRLAVSAPLEVYLQQGFLYGVSGSAYLSWPGNTLEESIVEEMSRNE  412

Query  339  EMIEKLQAGYFGLPFDYASFDHQPTTFEVKTMVRRVGEIVVSQVPKNYYYQTQLLVNKIV  398
                +++ G F LP+D+A FDHQPTT EV        E  +     N     +L    +
Sbjct  413  RTFCRMRDGAFALPYDFARFDHQPTTEEVIAFQTITFERAIVNASPNQRADVRLFEQLLE  472

Query  399  NAYDKSYLSGNIKNTKFENIKVKGGVPSGVRITSLLGNMWNAIITKIAINNVIGII-GYD  457
              + ++ L+      +     V GG+ SG+R TS +G+ WN+++ + A  ++ G+   YD
Sbjct  473  RGFRRATLATPPGICEPHVFPVTGGLMSGLRSTSAVGSGWNSVLGETA-RDLAGVFRRYD  531

Query  458  -PIS--QISLRGDDVAILSKDPAALYLLRLSYAAINAIGKDSKLGISPKVCEFLRNEISV  514
             PI   QI +RGDD  ++S     +  +++ Y A+ A   +SK  +     EFLR E
Sbjct  532  RPIETWQI-VRGDDTQVVSDYYLDVLAVKIGYDALGAEANESKFTLRRGRTEFLRVE---  587

Query  515  TG--VRGWTCRGIGGISQRKPWNPQPWSPNDEVETNASNISLLERRAGIELQQLHHINKV  572
            TG   R + CR +  + QRKPW+ +P + +  V       S+L RR       L   + V
Sbjct  588  TGDRARCYPCRTLPLLGQRKPWSARPAAADSGVHRVDKVFSVLARRLPDPADLLGFADHV  647

Query  573  KWSRHVRQSY--KYLELPKRLGGFGIYRFQG------WLPNGKLPLAKKPLVNVEDIHPS  624
                  + S   + L +P  LGG G+  + G      W  N ++P+    + N  D
Sbjct  648  LQRMMAKMSLDIRLLAIPTALGGLGLRDWNGKWHVRTWRSNLRVPVT---VTNQTDFRAG  704

Query  625  QEL------FLPLSEQQKKILAQVEMTNKMQTDD---IPGTQKLFSKEWIQKVRAKKIIW  675
            QE+       + L   +   LA   +  K+  DD   + GT +  SK  +Q   +++I+
Sbjct  705  QEMQRYQEYGIRLRPSEAAALADRSVREKIAVDDLVELAGTIRRVSKAEMQ---SREIVL  761

Query  676  SRNQTIP  682
            S N T P
Sbjct  762  S-NCTKP  767


>MAG: RNA-dependent RNA polymerase [Totiviridae sp.]
Sequence ID: WAK77448.1 Length: 833
Range 1: 3 to 663

Score:216 bits(551), Expect:1e-60,
Method:Compositional matrix adjust.,
Identities:187/677(28%), Positives:318/677(46%), Gaps:59/677(8%)

Query  49   NVPKIITDKELQTFYVPRSNADLVIRRIRLIDL--------WRNLKPDQMDEIRNYTHLD  100
            ++P+ +T +   +   PR+  D+ IR +R+ D+         RN   + +   R +   D
Sbjct  3    SLPRQMTTRWPNSSTPPRNEEDIGIRNLRMADMISYGLATGRRNRLHETLRLCRGF---D  59

Query  101  YIFVQNICIYMLVFGINTVKHFRQIGLFNERNEFIEIA-KQLSTKGKRFVDDVDNMKQKV  159
            YI   N+ I +  FG       R+ G+F   ++   ++ K LS K K+  +D++   ++V
Sbjct  60   YILTSNLLIAIYCFGDWWWDSLRRAGVFEGDSDHYRVSCKALSDKVKKTKNDIEP-DRRV  118

Query  160  C--EIATIVGYMDPNVDKIDVMEEVNSLAAEGN-EHGIDRDNW---NDLFTKTCKEVMTW  213
            C  E   + G M P V   D ++E   LA+ G   HG+ R +     D+  +  ++++ +
Sbjct  119  CFYECVALFGAMCPPVAGWDPVQETRDLASGGKIAHGLMRGDEMRPCDVVERI-RDLVRF  177

Query  214  YKGHEF--ISFDDYIKEGMWLTSGSSSIGKVHWTKDGEN----GKFKARKNMLLQIYTPQ  267
              G +    +F+D+++   W  SG+SSIG+V +  D E     G FKARKN++L +
Sbjct  178  KAGKKCDGRTFEDWLRSSDWERSGASSIGRVEYRIDQEGEERRGHFKARKNLVLDVVPLA  237

Query  268  ELANIVYAWDGKLHSRVFIKNEMSKLRLAVASNIEAYIHESYMLFLYGHGFKEYFGVTLD  327
             +   V   D +  ++  +K+E  K+RLAV++ +E Y+ + Y+  + G  +  + G TL+
Sbjct  238  TVLKSVREHDSQ-DNKALVKSEFGKIRLAVSAPLEVYLQQGYLYGVSGSAYLSWPGNTLE  296

Query  328  EKPDQQHQREIEMIEKLQAGYFGLPFDYASFDHQPTTFEVKTMVRRVGEIVVSQVPKNYY  387
            E   Q+  R       +QAG F LP+D+A FDHQPTT EV    R   E  V     N
Sbjct  297  ESVVQEMSRNERTFCNMQAGAFALPYDFARFDHQPTTEEVVAFQRITFERAVVNAGPNQK  356

Query  388  YQTQLLVNKIVNAYDKSYLSGNIKNTKFENIKVKGGVPSGVRITSLLGNMWNAIITKIAI  447
               +L  + + + +  + L+      +     V GG+ SG+R TS +G+ WN+++ + A
Sbjct  357  DDVKLFEDLLEHGFRCATLATPPGICEPHVFPVTGGLMSGLRSTSAVGSGWNSVLGETAR  416

Query  448  NNVIGIIGYD-PIS--QISLRGDDVAILSKDPAALYLLRLSYAAINAIGKDSKLGISPKV  504
            +       +D PI   QI +RGDD  ++S     +  +++ Y A+ A   +SK  +
Sbjct  417  DLAGMFRKFDRPIQTWQI-VRGDDTQVVSDHYLDVLAVKIGYDALGAEANESKFTLRRGR  475

Query  505  CEFLRNEISVTG--VRGWTCRGIGGISQRKPWNPQPWSPNDEVETNASNISLLERRAGIE  562
             EFLR E   TG   R + CR I  + QRKPW+ +P + +  V       ++L RR
Sbjct  476  TEFLRVE---TGDRARCYPCRTIPLLGQRKPWSARPAAADSGVHRVDKVFNVLARRLPDP  532

Query  563  LQQLHHINKVKWSRHVRQS--YKYLELPKRLGGFGIYRFQG------WLPNGKLPLAKKP  614
               L   + V      +     + L +P  LGG G+  + G      W PN ++P+
Sbjct  533  EDLLRFSDHVISRMMCKMGLDMRLLSIPTALGGLGLRDWSGLWHVRTWRPNLRVPVT---  589

Query  615  LVNVEDIHPSQEL------FLPLSEQQKKILAQVEMTNKMQTDD---IPGTQKLFSKEWI  665
            + N  D    QE+       + L   +   LA   +  K+  DD   + GT +  SK  +
Sbjct  590  VTNQTDFRAGQEMQRYQEYGIRLRPSEAAALADRSVREKIAVDDLVELAGTIRRVSKAEM  649

Query  666  QKVRAKKIIWSRNQTIP  682
            Q   +++II S     P
Sbjct  650  Q---SREIIASNCSRPP  663


>MAG: RNA-dependent RNA polymerase [Totiviridae sp.]
Sequence ID: WAK77483.1 Length: 1011
Range 1: 185 to 813

Score:218 bits(555), Expect:2e-60,
Method:Compositional matrix adjust.,
Identities:174/633(27%), Positives:299/633(47%), Gaps:38/633(6%)

Query  56   DKELQTFYVPRSNADLVIRRIRLIDLW----RNLKPDQMDEI-RNYTHLDYIFVQNICIY  110
            D+ +   +  RS+ DL IR +R  DLW     N   +++ ++ R   + DYI + N+ +
Sbjct  185  DRLILAAFPARSSQDLGIRYVRAPDLWVFYMENRSYEELHQVLRQLKYFDYICMVNLLLA  244

Query  111  MLVF-GINTVKHFRQIGLFNERNEFIEIA-KQLSTKGKRFVDDVD-NMKQKVC--EIATI  165
             +VF          + G F+  +E   +A KQLS   K+  ++V     ++VC  E A++
Sbjct  245  KIVFTNPQWWDTLARAGCFDGGSEHFRLACKQLSDWIKK--ENVQLPAAERVCYYECASL  302

Query  166  VGYMDPNVDKIDVMEEVNSLAAEG-NEHGI---DRDNWN-DLFTKTCKEVMTW--YKGHE  218
             G M P V   D + E   LA  G +EHG+    RD +  + F    +++         +
Sbjct  303  YGAMCPPVPGWDPILETRELAQGGQDEHGLLLSSRDGYAPEDFCAAIEDLAALPPRDSPD  362

Query  219  FISFDDYIKEGMWLTSGSSSIGKVHWTKDGEN----GKFKARKNMLLQIYTPQELANIVY  274
              +F ++++   W  +G+SSIG V +T + E     G+FKARKN LL +    +L   V
Sbjct  363  VRTFREFLESEEWARAGASSIGTVEFTLEAEGTRKTGRFKARKNFLLDVVELDDLERRVR  422

Query  275  AWDGKLHSRVFIKNEMSKLRLAVASNIEAYIHESYMLFLYGHGFKEYFGVTLDEKPDQQH  334
                +  +   IK E+SK+RLAV+S +E Y+ ++++  L G+ +  +   TL+E   ++
Sbjct  423  ECREQ-RNVALIKTELSKIRLAVSSPVEVYLPQTWLFLLSGNAYSSWPSNTLEEPLWKEQ  481

Query  335  QREIEMIEKLQAGYFGLPFDYASFDHQPTTFEVKTMVRRVGEIVVSQVPKNYYYQTQLLV  394
             R     E+LQAG + LP+D+A FDHQPTT E++   R   E     V  +     + L
Sbjct  482  ARHETTWERLQAGEYSLPYDFARFDHQPTTAEIQAFQRATNECARQMVQDHQRRDFEELT  541

Query  395  NKIVNAYDKSYLSGNIKNTKFENIKVKGGVPSGVRITSLLGNMWNAIITKIAINNVIGII  454
              ++  +  + L+           KV GG+ SG+R TS +G+ WNAI  + A   +  +
Sbjct  542  ANVLQGFSNATLTSPPGLGDQLTFKVTGGLMSGLRSTSCVGSGWNAIFGQFATRLIYDVR  601

Query  455  GYDPISQI--SLRGDDVAILSKDPAALYLLRLSYAAINAIGKDSKLGISPKVCEFLRNEI  512
            G      +   +RGDD  ++ +    +  ++L Y A+ AI  +SK  +     EFLR E
Sbjct  602  GGQAAPHVWQLVRGDDTQVVGRSYHDVLGVKLGYDALGAIANESKFTLRRGRTEFLRVET  661

Query  513  SVTGVRGWTCRGIGGISQRKPWNPQPWSPNDEVETNASNISLLERRA--GIELQQLHHIN  570
                +RG+ CR I  I QR+PWN +P      ++  A   S + RR      L
Sbjct  662  E-DRLRGYPCRTIPLIVQRRPWNARPVRTEAGLDHVAKAASTVRRRIPDPTGLDAFVGFF  720

Query  571  KVKWSRHVRQSYKYLELPKRLGGFGIYRFQGWLPNGKLPLAKKPLVNVEDIHPSQE----  626
              +    +    + L +P  +GG G+  ++G          + P V + +    +E
Sbjct  721  MTRTCGMLGLDERLLSIPAAVGGLGLRPWRGEWAVQCWSSVRAPPVTITNRTSYRETRVR  780

Query  627  -----LFLPLSEQQKKILAQVEMTNKMQTDDIP  654
                 + +P+  ++   +A+  + +K+ +DDIP
Sbjct  781  QDFDKIGVPVFHEEVARMAEERIRSKIASDDIP  813


>MAG: putative RNA-dependent RNA polymerase, partial [Totiviridae sp.]
Sequence ID: UHS72455.1 Length: 316
Range 1: 22 to 311

Score:202 bits(515), Expect:4e-60,
Method:Compositional matrix adjust.,
Identities:113/294(38%), Positives:167/294(56%), Gaps:12/294(4%)

Query  51   PKIITDKELQTFYVP-RSNADLVIRRIRLIDLWRNLKPDQMDEIRNYT----HLDYIFVQ  105
            P + T +      +P R+  D  IRR++    +R+++P    E+++      H D I+
Sbjct  22   PDLTTCQPTTIHDLPFRTKMDAAIRRLK----YRDIEPFLSAEVKSIIPWLHHTDQIWAT  77

Query  106  NICIYMLVFGINTVKHFRQIGLFNERNEFIEIAKQLSTKGKRFVDDVDNMKQKVCEIATI  165
            NI I+  + G+ +    ++ G FN    F++ +  LS   KRF    D  KQK  E+ ++
Sbjct  78   NILIWTQIHGVPSFMELKRYGFFNSLETFLDQSSILSAWVKRFGVQEDTNKQKYAELNSL  137

Query  166  VGYMDPNVDKIDVMEEVNSLAAEGNEHGIDRDNWNDLFTKTCKEV---MTWYKGHEFISF  222
             GYM  ++   D + E+ SLA  G  HG+   +W   F  + + +       +  +FISF
Sbjct  138  SGYMTSDLVDTDWLAELKSLAHGGKPHGLIGSDWKRDFITSNQHIKLPAIHDRRPKFISF  197

Query  223  DDYIKEGMWLTSGSSSIGKVHWTKDGENGKFKARKNMLLQIYTPQELANIVYAWDGKLHS  282
             DY++ G WLT+GSSSIG VHW    ENG F+ARKNML  ++T +EL ++V +WDG   +
Sbjct  198  QDYVESGQWLTAGSSSIGIVHWEAGEENGTFRARKNMLKYLFTAKELIDLVLSWDGVSRN  257

Query  283  RVFIKNEMSKLRLAVASNIEAYIHESYMLFLYGHGFKEYFGVTLDEKPDQQHQR  336
            + F K+E+ K RLAVASNIE Y+ ESY L LYGH +K +  VTLDE P Q H R
Sbjct  258  KAFTKDELGKRRLAVASNIEGYLCESYFLALYGHPYKNWKYVTLDETPGQSHNR  311


>MAG: RNA-dependent RNA polymerase [Totiviridae sp.]
Sequence ID: WAK77607.1 Length: 932
Range 1: 106 to 765

Score:212 bits(540), Expect:7e-59,
Method:Compositional matrix adjust.,
Identities:184/675(27%), Positives:318/675(47%), Gaps:60/675(8%)

Query  56   DKELQTFYVPRSNADLVIRRIRLIDLWR-----NLKPDQMDEIRNYTHLDYIFVQNICIY  110
            D+     +  R+N DL IR  R  D+W          +  D +R   + DYI + N+ I
Sbjct  106  DRYALAAFPARTNQDLGIRYARAADVWSFYIETGRSQELRDTLRLLKYFDYITMVNVLIA  165

Query  111  MLVFGINTV-KHFRQIGLFNERNEFIEIA-KQLS--TKGKRFVDDVDNMKQKVCEIATIV  166
             +VF          + G F    E   +A + LS   K +    D D+ +    E A++
Sbjct  166  KVVFTRAVWWDTLVRAGCFVAGAEHFRLAARALSDYVKKENVALDPDD-RVSFYECASLY  224

Query  167  GYMDPNVDKIDVMEEVNSLAAEG-NEHGIDRDNWNDLFTKTCKEVMTWYKGHEFI-----  220
            G M P V   D +EE  +LA  G +EHG+  D      ++   E +   +   F+
Sbjct  225  GAMCPPVPGWDPVEETQALAEGGSDEHGLLLDCSPGYSSEDFLEAV---RALAFVKPRDD  281

Query  221  ----SFDDYIKEGMWLTSGSSSIGKVHWT----KDGENGKFKARKNMLLQIYTPQELANI  272
                 F D++ +  W  SG+SSIG+V +T    ++  +GKFKARKN++L +   +EL
Sbjct  282  PDPRPFHDWLWQEEWARSGASSIGQVRYTLTLGEESMSGKFKARKNLVLDVVPLEELEER  341

Query  273  VYAWDGKLHSRVFIKNEMSKLRLAVASNIEAYIHESYMLFLYGHGFKEYFGVTLDEKPDQ  332
               +  +  +   +K E++K+RLAV+  IE Y+ ++++  + G+ +  +   TL+E
Sbjct  342  ARTYT-RQDNVALVKTELAKIRLAVSCPIETYLAQAWIYLVTGNSYSNWPANTLEEPLHV  400

Query  333  QHQREIEMIEKLQAGYFGLPFDYASFDHQPTTFEVKTMVRRVGEIVVSQVPKNYYYQTQL  392
            +  R  E  EKLQ G + LP+D+A FDHQPTT EV        +   +Q    Y    Q+
Sbjct  401  EVARHEETWEKLQDGQYSLPYDFARFDHQPTTAEVTAF-----QHATNQCGLRYARPEQM  455

Query  393  -----LVNKIVNAYDKSYLSGNIKNTKFENIKVKGGVPSGVRITSLLGNMWNAIITKIAI  447
                 L + +V  Y  + L+      K +  +V GG+ SG+R TS +G+ WNAI  + A
Sbjct  456  DDYLQLSSCVVEGYSHATLTSPPGLGKQQTFRVTGGLMSGLRSTSCVGSGWNAIFGEFA-  514

Query  448  NNVIGII--GYDPISQISL-RGDDVAILSKDPAALYLLRLSYAAINAIGKDSKLGISPKV  504
              +IG +    DP S   L RGDD  +       +  +++ Y A+ A+  +SK  +
Sbjct  515  KRMIGEMRDPDDPASVWQLVRGDDTQVTGVSYHDVLGIKIGYDAMGAVANESKFTLRRGR  574

Query  505  CEFLRNEISVTGVRGWTCRGIGGISQRKPWNPQPWSPNDEVETNASNISLLERR----AG  560
             EFLR E     +RG+ CR I  ++QR+PWN +P      +E     +S L  R    A
Sbjct  575  TEFLRIETE-DRLRGYPCRSIPLLTQRRPWNARPVMQEAGLEHVLKTMSTLRMRLPDPAA  633

Query  561  IELQQLHHINKVKWSRHVRQSYKYLELPKRLGGFGIYRFQG-WLPNGKLPLAKKPLVNVE  619
            ++  + H++ +    R +    +   +P  +GG G+  + G W      P+ + P V +E
Sbjct  634  LDEYREHYMRRC--FRLLGLDGRLRRIPVAMGGLGLDPWDGQWTVKTWRPI-RPPPVKIE  690

Query  620  DIHPSQE---------LFLPLSEQQKKILAQVEMTNKMQTDDIPGTQKLFSKEWIQKVRA  670
            +    +E         + + ++ ++ + +A+V +  K+  DD+P    +  K   +++RA
Sbjct  691  NRTSYREERVAKEFADIAVAITPEEARQMAEVRVQAKVAADDVPEFAGVLRKAGRRELRA  750

Query  671  KKIIWSRNQTIPIHT  685
            ++   ++ +   IH+
Sbjct  751  REYTPAQVRKERIHS  765


>MAG: RNA-dependent RNA polymerase, partial [Totiviridae sp.]
Sequence ID: WAK77488.1 Length: 826
Range 1: 212 to 778

Score:211 bits(537), Expect:8e-59,
Method:Compositional matrix adjust.,
Identities:166/574(29%), Positives:279/574(48%), Gaps:35/574(6%)

Query  56   DKELQTFYVPRSNADLVIRRIRLIDLW----RNLKPDQMDEI-RNYTHLDYIFVQNICIY  110
            D+ +   +  RS  DL IR +R  DLW     N   +++ ++ +     DYI + N+ +
Sbjct  212  DRLILAAFPARSPQDLGIRYVRAPDLWVFFMENRSYEELRQVLQQLKFFDYICMVNLLLA  271

Query  111  MLVF-GINTVKHFRQIGLFNERNEFIEIA-KQLSTKGKRFVDDVD-NMKQKVC--EIATI  165
             +VF          + G F+  +E   +A KQLS   K+  ++V     ++VC  E A++
Sbjct  272  KIVFTNPQWWDTLTRAGCFDGGSEHFRLACKQLSDWVKK--ENVQLPAAERVCYYECASL  329

Query  166  VGYMDPNVDKIDVMEEVNSLAAEG-NEHGI---DRDNWN-DLFTKTCKEVMTW--YKGHE  218
             G M P V   D + E   LA  G +EHG+    RD +  + F    +++ +       +
Sbjct  330  YGAMCPPVPGWDPILETRELAQGGQDEHGLLLSSRDGYAPEDFCAAIEDLASLPPRDSPD  389

Query  219  FISFDDYIKEGMWLTSGSSSIGKVHWTKDGEN----GKFKARKNMLLQIYTPQELANIVY  274
              +F ++++   W  +G+SSIG V +T + E     G+FKARKN LL +    +L   V
Sbjct  390  VRTFREFLESEEWARAGASSIGTVEFTLEAEGTRKTGRFKARKNFLLDVVELDDLERRVR  449

Query  275  AWDGKLHSRV-FIKNEMSKLRLAVASNIEAYIHESYMLFLYGHGFKEYFGVTLDEKPDQQ  333
              + K    V  IK E+SK+RLAV+S +E Y+ ++++  L G+ +  +   TL+E   ++
Sbjct  450  --ECKEQKNVALIKTELSKIRLAVSSPVEVYLPQTWLFLLSGNAYSSWPSNTLEEPLWKE  507

Query  334  HQREIEMIEKLQAGYFGLPFDYASFDHQPTTFEVKTMVRRVGEIVVSQVPKNYYYQTQLL  393
              R     E+LQAG + LP+D+A FDHQPTT E++   R   E     V  +  +  + L
Sbjct  508  QARHETTWERLQAGEYSLPYDFARFDHQPTTAEIQAFQRATNECARQMVQDHQRHDFEEL  567

Query  394  VNKIVNAYDKSYLSGNIKNTKFENIKVKGGVPSGVRITSLLGNMWNAIITKIAINNVIGI  453
               ++  +  + L+           KV GG+ SG+R TS +G+ WNAI  + A   +  +
Sbjct  568  TANVLQGFSNATLTSPPGLGDQLTFKVTGGLMSGLRSTSCVGSGWNAIFGQFATRLIYDV  627

Query  454  IGYDPISQI--SLRGDDVAILSKDPAALYLLRLSYAAINAIGKDSKLGISPKVCEFLRNE  511
             G      +   +RGDD  ++ +    +  ++L Y A+ AI  +SK  +     EFLR E
Sbjct  628  RGGQAAPNVWQLVRGDDTQVVGRSYHDVLGVKLGYDALGAIANESKFTLRRGRTEFLRVE  687

Query  512  ISVTGVRGWTCRGIGGISQRKPWNPQPWSPNDEVETNASNISLLERR----AGIELQQLH  567
                 +RG+ CR I  I QR+PWN +P      ++  A   S + RR    AG++
Sbjct  688  TE-DRLRGYPCRTIPLIVQRRPWNARPVRTEAGLDHVAKAASTVRRRIPDPAGLDAFVGF  746

Query  568  HINKVKWSRHVRQSYKYLELPKRLGGFGIYRFQG  601
             + +      +    + L +P  +GG G+  ++G
Sbjct  747  FMTRT--CGMLGLDERLLGIPVAVGGLGLRPWRG  778


>MAG: RNA-dependent RNA polymerase [Totiviridae sp.]
Sequence ID: WAK77258.1 Length: 938
Range 1: 115 to 817

Score:212 bits(540), Expect:9e-59,
Method:Compositional matrix adjust.,
Identities:196/726(27%), Positives:334/726(46%), Gaps:70/726(9%)

Query  56   DKELQTFYVPRSNADLVIRRIRLIDL----WRNLKPDQMDE-IRNYTHLDYIFVQNICIY  110
            D   Q  Y PR+  D+ IR +R+ D+        + D++ E +R     DYI   N+ I
Sbjct  115  DTLAQQLYPPRNEEDIGIRNLRMADMISYGLATGRRDRLHETLRLCRGFDYILTSNLLIA  174

Query  111  MLVFGINTVKHFRQIGLFNERNEFIEIA-KQLSTKGKRFVDDVDNMKQKVC--EIATIVG  167
            +  FG       R+ G+F   ++   ++ K LS K K+  +D++  K +VC  E   + G
Sbjct  175  IYCFGDWWWDSLRRAGVFEGDSDHYRVSCKALSDKVKKTKNDIEPDK-RVCFYECVALFG  233

Query  168  YMDPNVDKIDVMEEVNSLAAEGN-EHGIDRDNW---NDLFTKTCKEVMTWYKGHEF--IS  221
             M P V   D ++E   LA+ G   HG+ R +     D+  +  ++++ +  G +    +
Sbjct  234  AMCPPVAGWDPVQETRDLASGGKIAHGLMRGDEMRPCDVVERI-RDLVRFKAGKKCDGRT  292

Query  222  FDDYIKEGMWLTSGSSSIGKVHWTKDGEN----GKFKARKNMLLQIYTPQELANIVYAWD  277
            F+D+++   W  SG+SSIG+V +  D E     G FKARKN++L +     +   V   D
Sbjct  293  FEDWLRSSDWERSGASSIGRVEYRIDQEGEERKGHFKARKNLVLDVVPLATVLKSVREHD  352

Query  278  GKLHSRVFIKNEMSKLRLAVASNIEAYIHESYMLFLYGHGFKEYFGVTLDEKPDQQHQRE  337
             +  ++  +K+E  K+RLAV++ +E Y+ + Y+  + G  +  + G TL+E   ++  R
Sbjct  353  SQ-DNKALVKSEFGKIRLAVSAPLEVYLQQGYLYGVSGSAYLSWPGNTLEESIVEEMSRN  411

Query  338  IEMIEKLQAGYFGLPFDYASFDHQPTTFEVKTMVRRVGEIVVSQVPKNYYYQTQLLVNKI  397
                 +++ G F LP+D+A FDHQPTT EV        E  +     N     +L    +
Sbjct  412  ERTFCRMRDGAFALPYDFARFDHQPTTEEVIAFQTITFERAIVNASPNQRADVRLFEQLL  471

Query  398  VNAYDKSYLSGNIKNTKFENIKVKGGVPSGVRITSLLGNMWNAIITKIAINNVIGII--G  455
               + ++ L+      +     V GG+ SG+R TS +G+ WN+++ + A  ++ G+
Sbjct  472  ERGFRRATLATPPGICEPHVFPVTGGLMSGLRSTSAVGSGWNSVLGETA-RDLAGVFRRC  530

Query  456  YDPIS--QISLRGDDVAILSKDPAALYLLRLSYAAINAIGKDSKLGISPKVCEFLRNEIS  513
              PI   QI +RGDD  ++S     +  +++ Y A+ A   +SK  +     EFLR E
Sbjct  531  DRPIETWQI-VRGDDTQVVSDYYLDVLAVKIGYDALGAEANESKFTLRRGRTEFLRVE--  587

Query  514  VTG--VRGWTCRGIGGISQRKPWNPQPWSPNDEVETNASNISLLERRAGIELQQLHHINK  571
             TG   R + CR +  + QRKPW+ +P + +  V       S+L RR       L   +
Sbjct  588  -TGDRARCYPCRTLPLLGQRKPWSARPAAADSGVHRVDKVFSVLARRLPDPADLLGFADH  646

Query  572  VKWSRHVRQSY--KYLELPKRLGGFGIYRFQG------WLPNGKLPLAKKPLVNVEDIHP  623
            V      + S   + L +P  LGG G+  + G      W  N ++P+    + N  D
Sbjct  647  VLRRMMAKMSLDIRLLAIPTALGGLGLRDWNGKWHVRTWRSNLRVPVT---VTNQTDFRA  703

Query  624  SQEL------FLPLSEQQKKILAQVEMTNKMQTDD---IPGTQKLFSKEWIQKVRAKKII  674
             QE+       + L   +   LA   +  K+  DD   + GT +  SK  +Q   +++II
Sbjct  704  GQEMQRYQEYGIRLRPSEAAALADRSVREKIAVDDLVELAGTIRRVSKAEMQ---SREII  760

Query  675  WSRNQTIP------IHTDHTVRIPKWDEKIKFPRYKSEYILNNKINLTMEQVLRQYNLLK  728
             S     P       ++   +R    D  +    Y S   L+N +   ++ V R Y
Sbjct  761  ASNCSRPPPVSLLRCYSHLLLRARSLD--VAEGSYAS---LDNDVGRQLQDVARLYG---  812

Query  729  EVERHD  734
              ERH+
Sbjct  813  -SERHN  817


>MAG: putative RNA-dependent RNA polymerase, partial [Totiviridae sp.]
Sequence ID: UHS72457.1 Length: 351
Range 1: 132 to 351

Score:199 bits(507), Expect:1e-58,
Method:Compositional matrix adjust.,
Identities:94/220(43%), Positives:142/220(64%), Gaps:0/220(0%)

Query  64   VPRSNADLVIRRIRLIDLWRNLKPDQMDEIRNYTHLDYIFVQNICIYMLVFGINTVKHFR  123
            +PR+N DL IRR+R+ D+ R LK +++DE+R + HLD I   NIC++ +V G   ++  R
Sbjct  132  LPRTNGDLAIRRLRIKDIMRVLKKEEIDEMRFFAHLDNIMFANICVFGIVNGFGKLRDLR  191

Query  124  QIGLFNERNEFIEIAKQLSTKGKRFVDDVDNMKQKVCEIATIVGYMDPNVDKIDVMEEVN  183
            + G+FN  + F+E A ++S+  KR+   +D+ K  +CEI  + GY+  +    D  +E +
Sbjct  192  ECGIFNSFDIFVEAASRISSHFKRYPVCLDDTKMMLCEINCLTGYLQSDPPGWDFEKEFD  251

Query  184  SLAAEGNEHGIDRDNWNDLFTKTCKEVMTWYKGHEFISFDDYIKEGMWLTSGSSSIGKVH  243
            +L   G EHG+   +W   F    K VMT    + ++S DDYI+ G+W+T+G+SSIGKV
Sbjct  252  ALGKGGYEHGLLNSDWKKDFVGALKRVMTRQSVNNWVSLDDYIRSGLWITAGASSIGKVE  311

Query  244  WTKDGENGKFKARKNMLLQIYTPQELANIVYAWDGKLHSR  283
            W+  GE+GKFKARKNML ++Y+  EL +IV  WDG L SR
Sbjct  312  WSYSGESGKFKARKNMLTELYSDDELISIVRKWDGVLRSR  351


>MAG: RNA-dependent RNA polymerase [Totiviridae sp.]
Sequence ID: WAK77681.1 Length: 932
Range 1: 106 to 765

Score:211 bits(536), Expect:2e-58,
Method:Compositional matrix adjust.,
Identities:183/684(27%), Positives:320/684(46%), Gaps:78/684(11%)

Query  56   DKELQTFYVPRSNADLVIRRIRLIDLWRN-LKPDQMDEIRNYTHL----DYIFVQNICIY  110
            D+     +  R++ DL IR  R  D+W   +   +  E+R   HL    DYI + N+
Sbjct  106  DRYALAAFPARTSQDLGIRYARAADIWSFFISRGRQQELREDLHLLKAFDYITMVNVLSA  165

Query  111  MLVFGINTV-KHFRQIGLFNERNEFIEIA-KQLS--TKGKRFVDDVDNMKQKVCEIATIV  166
             +VF  +      ++ G F+   E   +A + LS   K +    D D+ +    E+A++
Sbjct  166  KIVFTRDLWWATLKRAGCFSGGAEHFRLAARSLSDIVKKENVSLDPDD-RVSFYEVASLY  224

Query  167  GYMDPNVDKIDVMEEVNSLAAEG-NEHGIDRDNWNDLFTKTCKEVMTWYKGHEFIS----  221
            G M P V   D +EE  +LA  G ++HG+           +C      Y   +FI+
Sbjct  225  GTMCPPVPGWDPVEETQALAQGGQDQHGL---------LLSCAPG---YGSQDFIAAVRA  272

Query  222  --------------FDDYIKEGMWLTSGSSSIGKVHWTKD-GE---NGKFKARKNMLLQI  263
                          F D++ +  W  SG+SS+G+V +  + GE   +GKFKARKN++L +
Sbjct  273  LAYVPPRDAPDGRPFRDWLWDEEWARSGASSLGRVEYALELGEEVKSGKFKARKNLVLDV  332

Query  264  YTPQELANIVYAWDGKLHSRVFIKNEMSKLRLAVASNIEAYIHESYMLFLYGHGFKEYFG  323
                EL      +  +  +   IK E++K+RLAV+  IE Y+ ++++  + G+ +  +
Sbjct  333  IPIDELEQRTRCYTQQ-ENVALIKTELAKIRLAVSCPIETYLAQAWIYLVSGNAYTNWPA  391

Query  324  VTLDEKPDQQHQREIEMIEKLQAGYFGLPFDYASFDHQPTTFEVKTMVRRVGEIVVSQVP  383
             TL+E    +  R  E  E+LQ G + LP+D+A FDHQPTT EVK       ++  +
Sbjct  392  NTLEEPLPVEVARHEETWERLQKGEYSLPYDFARFDHQPTTSEVKAF-----QVATNICG  446

Query  384  KNYYYQTQL-----LVNKIVNAYDKSYLSGNIKNTKFENIKVKGGVPSGVRITSLLGNMW  438
            + Y    QL     L   I   YD + L+        +  +V GG+ SG+R TS +G+ W
Sbjct  447  RRYAVPEQLADYDQLAYNITVGYDHATLTSPPGLGDQQTFRVTGGLMSGLRSTSCVGSGW  506

Query  439  NAIITKIAINNVIGIIGYDPISQI--SLRGDDVAILSKDPAALYLLRLSYAAINAIGKDS  496
            NAI  + A   +  I   D  +++   +RGDD  +  +    +  +++ Y A+ AI  +S
Sbjct  507  NAIFGEFARKMINDIRNPDRPARVWQLVRGDDTQVTGESYHDVLAIKIGYDAMGAIANES  566

Query  497  KLGISPKVCEFLRNEISVTG--VRGWTCRGIGGISQRKPWNPQPWSPNDEVETNASNISL  554
            K  +     EFLR E   TG  +RG+ CR I  ++QR+PWN +P      +E     ++
Sbjct  567  KFTLRQGRTEFLRIE---TGDRLRGYPCRTIPLLTQRRPWNARPVVQEAGLEHVLKTMAT  623

Query  555  LERR----AGIELQQLHHINKVKWSRHVRQSYKYLELPKRLGGFGIYRFQG-WLPNGKLP  609
            L  R    +G++  + H+I +    R +    +   +P  LGG G+  + G W
Sbjct  624  LRLRLPDPSGLDAYREHYIQRCM--RLLSLDARLRRIPVALGGLGLDPWDGRWTVTTWRA  681

Query  610  LAKKP--LVNVEDIHPS------QELFLPLSEQQKKILAQVEMTNKMQTDDIPGTQKLFS  661
            +   P  ++N  D          +E+ +P+++ +   +A+  +  K+  DD+P    +
Sbjct  682  VRAPPVKILNQTDYRQRRVAREFEEIGVPVTDAEVASMAETRIQAKVAADDVPEFAGVLR  741

Query  662  KEWIQKVRAKKIIWSRNQTIPIHT  685
            +   ++++A+K   ++ +   IH
Sbjct  742  RASRRELKARKYAPAQVRADRIHA  765


>MAG: RNA-dependent RNA polymerase [Totiviridae sp.]
Sequence ID: WAK77316.1 Length: 830
Range 1: 7 to 664

Score:208 bits(529), Expect:8e-58,
Method:Compositional matrix adjust.,
Identities:174/663(26%), Positives:302/663(45%), Gaps:37/663(5%)

Query  56   DKELQTFYVPRSNADLVIRRIRLIDLWRNLKPDQMDE-----IRNYTHLDYIFVQNICIY  110
            D+  ++ + PR+  DL IR+ R+ D+       Q        +R     DY    N+ +
Sbjct  7    DELAKSLFPPRTKEDLTIRKTRIGDVIEYAHATQSTAEILRILRLCRGYDYALTCNLLVA  66

Query  111  MLVFGINTVKHFRQIGLFNERNE-FIEIAKQLS-TKGKRFVDDVDNMKQKVCEIATIVGY  168
             L             G F+   E +   AK LS T  KR  +  D+ +  + E A + G
Sbjct  67   GLAMDELWWHILEGSGCFDGDGEHYRASAKVLSDTVKKRKNELSDHDRVSLYECAALFGA  126

Query  169  MDPNVDKIDVMEEVNSLAAEGN-EHGIDRDNW---NDLFTKTCKEV-MTWYKGHEFISFD  223
            + P V   D ++E   LA  G   HG+   +W   N +  K          K H+  +FD
Sbjct  127  LCPPVPGWDPVQETRDLATGGQVAHGLLSGDWLRANHILDKIAGLAQFPEAKPHDVRNFD  186

Query  224  DYIKEGMWLTSGSSSIGKVHWTKDGE----NGKFKARKNMLLQIYTPQELANIVYAWDGK  279
            ++++   W  SGSSSIG+V +  D       G FKARKN+++ +  P E  N
Sbjct  187  EWLRSCEWERSGSSSIGRVRYEVDDRLGRRTGHFKARKNLVMDV-VPYEEVNQRVREHNT  245

Query  280  LHSRVFIKNEMSKLRLAVASNIEAYIHESYMLFLYGHGFKEYFGVTLDEKPDQQHQREIE  339
              +   IK+E  K+RLAVA+ +E Y+ ++Y+  + G  +  + G TL+E  +++  R
Sbjct  246  QDNHALIKSEFGKIRLAVAAPLETYLQQAYLYSVSGCAYLRWPGNTLEESIEEEMHRNER  305

Query  340  MIEKLQAGYFGLPFDYASFDHQPTTFEVKTMVRRVGEIVVSQVPKNYYYQTQLLVNKIVN  399
             +  ++AG++ LP+D+A FDHQPTT EV    R   +        + +   ++    +
Sbjct  306  TVVSMRAGHYALPYDFARFDHQPTTSEVVAFQRVTFDRAFLNAGYHQFDDVRMFEELLEC  365

Query  400  AYDKSYLSGNIKNTKFENIKVKGGVPSGVRITSLLGNMWNAIITKIAINNVIGIIGYD-P  458
             +  + L+      +     V+GG+ SG+R TS +G+ WNA++ + A   V    G+  P
Sbjct  366  GFASATLTTPPGVCEPRTFPVQGGLMSGLRSTSSVGSGWNAVLGECAREMVSRFRGFSRP  425

Query  459  ISQ-ISLRGDDVAILSKDPAALYLLRLSYAAINAIGKDSKLGISPKVCEFLRNEISVTGV  517
            +     +RGDD  ++S     +  +++ Y A+ A   +SK  +     EFLR E     +
Sbjct  426  VETWQQVRGDDTQVVSPFYLDVLGVKIGYDALGAEANESKFTLRRGRTEFLRVETGQFSM  485

Query  518  RGWTCRGIGGISQRKPWNPQPWSPNDEVETNASNISLLERRAGIELQQLHHIN--KVKWS  575
              + CR I  ++QRKPWN +P + +  V T      +L RR     + L  I     K +
Sbjct  486  -CYPCRTIPLVNQRKPWNVRPPTGDGSVSTIVKVGHVLSRRLKNAARVLGFIVFLSYKMA  544

Query  576  RHVRQSYKYLELPKRLGGFGIYRFQG------WLPNGKLPLAKKPLVNVEDIHPSQE---  626
            R +    + + +P  LGG G+  ++G      W P+   P A   ++N  D    QE
Sbjct  545  RMMGIDARLIAIPVALGGLGLIPWKGKWCVKNWHPD---PPAPVRILNRTDFRAKQEQTR  601

Query  627  ---LFLPLSEQQKKILAQVEMTNKMQTDDIPGTQKLFSKEWIQKVRAKKIIWSRNQTIPI  683
               L + ++E +   LA   + +K+  DD+     + +     ++R ++I+    +  P+
Sbjct  602  YADLGVRITEHEADTLADRSVRDKIAADDLVDVAGVIAFARRGELRGREIVMCDARLAPL  661

Query  684  HTD  686
            + +
Sbjct  662  NLE  664


>MAG: RNA-dependent RNA polymerase [Totiviridae sp.]
Sequence ID: WAK77387.1 Length: 802
Range 1: 27 to 631

Score:206 bits(524), Expect:3e-57,
Method:Compositional matrix adjust.,
Identities:177/619(29%), Positives:293/619(47%), Gaps:49/619(7%)

Query  99   LDYIFVQNICIYMLVFGINTVKHFRQIGLFNERNEFIEIA-KQLSTKGKRFVDDVDNMKQ  157
             DYI   N+ I +  FG       ++ G+F+  +E   +A K LS K K+  +DVD  K
Sbjct  27   FDYILTNNLLIAVFCFGEWWWDVLKRAGVFDGDSEHYRLACKALSDKVKKTRNDVDADK-  85

Query  158  KVC--EIATIVGYMDPNVDKIDVMEEVNSLAAEGN-EHGIDRDNW---NDLFTKTCKEV-  210
            +VC  E A++ G M P V   D ++E   LA+ G   HG+ R       D+  +    V
Sbjct  86   RVCFYECASLYGAMCPPVPGWDPIQETRDLASGGKVPHGLTRGGVLRPADVLARIQDLVR  145

Query  211  MTWYKGHEFISFDDYIKEGMWLTSGSSSIGKVHWTKDGEN----GKFKARKNMLLQIYTP  266
             T  K  +  +F++++  G W  SG+SSIG+V +  D E     G FKARKN++L +
Sbjct  146  FTAGKKCDRRTFEEWLASGDWERSGASSIGRVEYRIDQEGEERKGHFKARKNLVLDVVPV  205

Query  267  QELANIVYAWDGKLHSRVFIKNEMSKLRLAVASNIEAYIHESYMLFLYGHGFKEYFGVTL  326
             +L   V   D +  ++  +K+E  K+RLAV++ +E Y+ + ++  + G  +  + G TL
Sbjct  206  PDLLERVRCHDTQ-ENKALVKSEFGKVRLAVSAPLEVYLQQGFLYGVSGSAYLSWPGNTL  264

Query  327  DEKPDQQHQREIEMIEKLQAGYFGLPFDYASFDHQPTTFEVKTMVRRVGEIVVSQVPKNY  386
            +E   ++  R      +++ G F LP+D+A FDHQPTT EV        E  +     N
Sbjct  265  EESIVEEMSRNERTFCRMRDGAFALPYDFARFDHQPTTEEVIAFQTITFERAIVNASPNQ  324

Query  387  YYQTQLLVNKIVNAYDKSYLSGNIKNTKFENIKVKGGVPSGVRITSLLGNMWNAIITKIA  446
                +L    +   + ++ L+      +     V GG+ SG+R TS +G+ WN+++ + A
Sbjct  325  RADVRLFEQLLERGFRRATLATPPGICEPHVFPVTGGLMSGLRSTSAVGSGWNSVLGETA  384

Query  447  INNVIGIIGY--DPIS--QISLRGDDVAILSKDPAALYLLRLSYAAINAIGKDSKLGISP  502
              ++ G+      PI   QI +RGDD  ++S     +  +++ Y A+ A   +SK  +
Sbjct  385  -RDLAGVFRRCDRPIETWQI-VRGDDTQVVSDYYLDVLAVKIGYDALGAEANESKFTLRR  442

Query  503  KVCEFLRNEISVTG--VRGWTCRGIGGISQRKPWNPQPWSPNDEVETNASNISLLERRAG  560
               EFLR E   TG   R + CR +  + QRKPW+ +P + +  V       S+L RR
Sbjct  443  GRTEFLRVE---TGDRARCYPCRTLPLLGQRKPWSARPAAADSGVHRVDKVFSVLARRLP  499

Query  561  IELQQLHHINKVKWSRHVRQSY--KYLELPKRLGGFGIYRFQG------WLPNGKLPLAK  612
                 L   + V      + S   + L +P  LGG G+  + G      W  N ++P+
Sbjct  500  DPADLLGFADHVLRRMMSKMSLDIRLLAIPTALGGLGLRDWNGKWHVRTWRSNLRVPVT-  558

Query  613  KPLVNVEDIHPSQEL------FLPLSEQQKKILAQVEMTNKMQTDD---IPGTQKLFSKE  663
              + N  D    QE+       + L   +   LA   +  K+  DD   + GT +  S+
Sbjct  559  --VTNQTDFRAGQEMQRYQEYGIRLRPSEATALADRSVREKIAVDDLVELAGTIRRVSRA  616

Query  664  WIQKVRAKKIIWSRNQTIP  682
             +Q   +++I+ S N T P
Sbjct  617  EMQ---SREIVLS-NCTKP  631


>MAG: RNA-dependent RNA polymerase [Totiviridae sp.]
Sequence ID: WAK77262.1 Length: 844
Range 1: 21 to 678

Score:205 bits(522), Expect:8e-57,
Method:Compositional matrix adjust.,
Identities:173/661(26%), Positives:308/661(46%), Gaps:33/661(4%)

Query  56   DKELQTFYVPRSNADLVIRRIRLIDL--WRNLKPDQMDEIRNYTHL----DYIFVQNICI  109
            D+  +  + PR+  DL IR+ R+ D+  + +L    +DE+R    L    DY    N+ +
Sbjct  21   DEIAKNLFPPRTKEDLTIRKTRIGDVIEYAHLT-GCVDEMRRILRLCRGYDYALTCNLLV  79

Query  110  YMLVFGINTVKHFRQIGLFNERNE-FIEIAKQLS-TKGKRFVDDVDNMKQKVCEIATIVG  167
              L           + G F+   E +   AK LS T  KR  +  D+ +  + E A + G
Sbjct  80   AGLAMDELWWTILEESGCFDGDGEHYRATAKILSDTVKKRKNELSDHDRVSLYECAALYG  139

Query  168  YMDPNVDKIDVMEEVNSLAAEGN-EHGIDRDNW--NDLFTKTCKEVMTW--YKGHEFISF  222
             + P V   D + E   LA  G   HG+   +W  +D        +  +   K H+   F
Sbjct  140  ALCPPVPGWDPVRETRDLATGGQVAHGLLSGDWLRSDHILDMIAGLAQFPEAKPHDVRGF  199

Query  223  DDYIKEGMWLTSGSSSIGKVHWTKDGENGK----FKARKNMLLQIYTPQELANIVYAWDG  278
            D++++   W  SGSSSIG+V +  D   GK    FKARKN+++ +   +E+ + V +
Sbjct  200  DEWLQSCEWERSGSSSIGRVRYEIDDRLGKRTGHFKARKNLVMDVVPFEEVRDRVRS-HA  258

Query  279  KLHSRVFIKNEMSKLRLAVASNIEAYIHESYMLFLYGHGFKEYFGVTLDEKPDQQHQREI  338
               +R  IK+E  K+RLAVA+ +E Y+ ++Y+  + G  +  + G TL+E  D++  R
Sbjct  259  TQDNRALIKSEFGKIRLAVAAPLETYLQQAYLYAVSGCAYLRWPGNTLEESIDEEMCRNE  318

Query  339  EMIEKLQAGYFGLPFDYASFDHQPTTFEVKTMVRRVGEIVVSQVPKNYYYQTQLLVNKIV  398
              +  ++   + LP+D+A FDHQPTT EV    R   +        + Y   +   + +
Sbjct  319  RTVVCMREEQYALPYDFARFDHQPTTSEVVAFQRVTFDRAFLNAAHHQYDDVRSFEDLLE  378

Query  399  NAYDKSYLSGNIKNTKFENIKVKGGVPSGVRITSLLGNMWNAIITKIAINNVIGIIGYD-  457
              +  + L+      + +  +V GG+ SG+R TS +G+ WNA++ + A   V     +
Sbjct  379  RGFAAATLTTPPGVCEPQTFRVTGGLMSGLRSTSSVGSGWNAVLGECAREMVSRFRSFSR  438

Query  458  PISQ-ISLRGDDVAILSKDPAALYLLRLSYAAINAIGKDSKLGISPKVCEFLRNEISVTG  516
            P+     +RGDD  ++S     +  +++ Y A+ A   +SK  +     EFLR E
Sbjct  439  PVETWQQVRGDDTQVVSPFYLDVLGVKIGYDALGAEANESKFTLRRGRTEFLRVETGRFS  498

Query  517  VRGWTCRGIGGISQRKPWNPQPWSPNDEVETNASNISLLERRAGIELQQLHHIN--KVKW  574
            +  + CR +  I+QRKPWN +P + +  V T      +L RR     + +  I     K
Sbjct  499  M-CYPCRTVPLINQRKPWNVRPPTGDGSVSTIVKVGHVLSRRLKNAQRVMGFITFLSYKM  557

Query  575  SRHVRQSYKYLELPKRLGGFGIYRFQG------WLPNGKLP---LAKKPLVNVEDIHPSQ  625
            +R +    + + +P  LGG G+  + G      W P+  +P   L K      ++
Sbjct  558  ARMMGIDPRLVSIPVALGGLGLLPWAGKWCVRNWHPDPPIPVRILNKTTFRADQESRQFA  617

Query  626  ELFLPLSEQQKKILAQVEMTNKMQTDDIPGTQKLFSKEWIQKVRAKKIIWSRNQTIPIHT  685
            EL + +S  + ++LA   + +K+  DD+     + +     ++R ++I+    +  P+ +
Sbjct  618  ELGVRISRHEAEVLADRRVKDKVAADDLVDVAGVVAYTRRNELRRREIVTCDARLAPLSS  677

Query  686  D  686
            +
Sbjct  678  N  678


>MAG: RNA-dependent RNA polymerase [Totiviridae sp.]
Sequence ID: WAK77610.1 Length: 871
Range 1: 41 to 673

Score:204 bits(519), Expect:3e-56,
Method:Compositional matrix adjust.,
Identities:175/644(27%), Positives:297/644(46%), Gaps:52/644(8%)

Query  52   KIITDKELQTFYVPRSNADLVIRRIRLIDLW-----RNLKPDQMDEIRNYTHLDYIFVQN  106
            ++  D+     +  RS+ DL IR  R  D+W        + + +D +    H DYI + N
Sbjct  41   RVEDDRYALEAFPARSSQDLGIRYARAADVWTYYLDHGRRQELVDTLHQLKHFDYITMVN  100

Query  107  ICIYMLVFGINTV--KHFRQIGLFNERNEFIEIA-KQLST--KGKRFVDDVDNMKQKVCE  161
            +    LVF +N V      + G      E   +A + LS   K +R     D+ +    E
Sbjct  101  LLTAKLVF-VNPVWWAALERAGCLRAGAEHFRLAGRALSDLLKKERVALPPDD-RVAFYE  158

Query  162  IATIVGYMDPNVDKIDVMEEVNSLAAEG-NEHGI--------DRDNWNDLFTKTCKEVMT  212
            +A++ G M P V   D ++E  +LA  G ++HG+           ++++   +
Sbjct  159  VASLYGAMCPPVPGWDPIDETQALAQGGQDQHGLLLNAQPGYSSADFHNAIRRLAAARPR  218

Query  213  WYKGHEFISFDDYIKEGMWLTSGSSSIGKVHWT----KDGENGKFKARKNMLLQIYTPQE  268
                 +   F  ++ +  W  SG+SS+G+VH+T     +   GKFKARKN++L +    E
Sbjct  219  --DDPDPRPFRTWLWDEEWARSGASSLGRVHYTLSVGDEVRAGKFKARKNLVLDVIPIDE  276

Query  269  LANIVYAWDGKLHSRVFIKNEMSKLRLAVASNIEAYIHESYMLFLYGHGFKEYFGVTLDE  328
            L      +  +  +   IK E++K+RLAV+S IE Y+ ++++  + G+ +  +   TL+E
Sbjct  277  LEERTRHYTIQ-ENVALIKTELAKIRLAVSSPIETYLAQAWIFLVTGNAYANWPANTLEE  335

Query  329  KPDQQHQREIEMIEKLQAGYFGLPFDYASFDHQPTTFEV---KTMVRRVGEIVVSQVPKN  385
                +  R  E   +L+AG F LP+D+A FDHQPTT EV   +T     G    +
Sbjct  336  PLHAELARLEETWLRLRAGQFSLPYDFARFDHQPTTSEVTAFQTATNMCGRQFATPEQSE  395

Query  386  YYYQTQLLVNKIVNAYDKSYLSGNIKNTKFENIKVKGGVPSGVRITSLLGNMWNAIITKI  445
             Y Q   L   +   Y ++ L+           +V GG+ SG+R TS +G+ WNAI  +
Sbjct  396  DYDQ---LTRNVTLGYSQATLTSPPGLGDQRTFRVTGGLMSGLRSTSCVGSGWNAIFGEF  452

Query  446  AINNVIGIIGYDPISQI--SLRGDDVAILSKDPAALYLLRLSYAAINAIGKDSKLGISPK  503
            A   +  I   D    +   +RGDD  +       +  ++L Y A+ AI  +SK  +
Sbjct  453  ARQLIFDIRHPDSPPNVWQLVRGDDTQVCGASYHDVLAIKLGYDALGAIANESKFTLRRG  512

Query  504  VCEFLRNEISVTGVRGWTCRGIGGISQRKPWNPQPWSPNDEVETNASNISLLERR----A  559
              EFLR E     +RG+ CR I  +SQR+PWN +P      +E     +S L  R
Sbjct  513  RTEFLRVETE-DRLRGYPCRTIPLLSQRRPWNARPVLQEAGLEHVLKTMSTLRLRIADPT  571

Query  560  GIELQQLHHINKVKWSRHVRQSYKYLELPKRLGGFGIYRFQG-WLPNGKLPLAKKP--LV  616
            G++  + H++ +    R +    +   +P  +GG G+  + G W       +   P  +V
Sbjct  572  GLDSYREHYVQRC--FRLLGFDARLRRIPAAMGGLGLDPWDGRWAVATWRSVNAPPVKIV  629

Query  617  NVEDIHP---SQELF---LPLSEQQKKILAQVEMTNKMQTDDIP  654
            N  D      +QE     +P++ Q+ +++A+  +  K+  DD+P
Sbjct  630  NRTDYRRQRVTQEFAKAGIPVTVQETEVMAEKRIQAKVAADDVP  673


>MAG: RNA-dependent RNA polymerase [Totiviridae sp.]
Sequence ID: UUT42649.1 Length: 1053
Range 1: 230 to 854

Score:203 bits(517), Expect:1e-55,
Method:Compositional matrix adjust.,
Identities:174/638(27%), Positives:306/638(47%), Gaps:53/638(8%)

Query  56   DKELQTFYVPRSNADLVIRRIRLID-----LWRNLKPDQMDEIRNYTHLDYIFVQNICIY  110
            D   QT Y PR+ AD+ IR +R+ D     L  N +    + +R     DYI   N+
Sbjct  230  DTLAQTLYPPRNEADMGIRNLRMADMLTYCLATNRRARAAEVLRLCQGFDYILTSNVLTA  289

Query  111  MLVFGINTVKHFRQIGLFNERNE-FIEIAKQLSTKGKRFVDDVDNMKQKVC--EIATIVG  167
            +  FG    K  +  G+F+  +E +    K LS   K+  +++ +   +VC  E A++ G
Sbjct  290  IFCFGDLWWKGLQDAGVFDGDSEHYRTCCKALSDTIKKTKNELSD-DDRVCFYECASLFG  348

Query  168  YMDPNVDKIDVMEEVNSLAAEG-NEHGIDRDNWNDLFTKTC-----KEVMTWYKGHEFIS  221
             M P V   D ++E   LA+ G + HG+      D  T          ++ + +G +  +
Sbjct  349  AMCPPVPGWDPIQETRELASGGQHPHGL---LAGDELTPAVILGRIHGLVRYREGKKCDT  405

Query  222  --FDDYIKEGMWLTSGSSSIGKVHWTKDG----ENGKFKARKNMLLQIYTPQELANIVYA  275
              F++++    W  SGSSSIG+V +  +       G FKARKN++L +   ++L   V +
Sbjct  406  RPFEEWLASCEWERSGSSSIGRVEYEVEVDEEKRRGHFKARKNLVLDVVPFEDLVTSVRS  465

Query  276  WDGKLHSRVFIKNEMSKLRLAVASNIEAYIHESYMLFLYGHGFKEYFGVTLDEKPDQQHQ  335
             D +  ++  +K+E  K+RLAV++ +E Y+ + Y+  + G  + ++ G TL+E   ++ +
Sbjct  466  HDTQ-DNKALVKSEFGKVRLAVSAPLEVYLQQGYLYGVSGSAYLQWPGNTLEESISEEMR  524

Query  336  REIEMIEKLQAGYFGLPFDYASFDHQPTTFEVKTM-VRRVGEIVVSQVPKNYYYQTQLLV  394
            R      ++  G F LP+D+A FDHQPTT EV           +V+ +P       +L
Sbjct  525  RNELTYTRMARGGFALPYDFARFDHQPTTDEVVAFQTITFDRALVNALPPQ-RSDIELFE  583

Query  395  NKIVNAYDKSYLSGNIKNTKFENIKVKGGVPSGVRITSLLGNMWNAIITKIAINNVIGII  454
              + + +  + L+      +     V GG+ SG+R TS +G+ WN+++ + A +
Sbjct  584  RLLEHGFRHATLATPPGICEPHVFPVTGGLMSGLRSTSAVGSGWNSVLGESARDMAGRFR  643

Query  455  GYD-PIS--QISLRGDDVAILSKDPAALYLLRLSYAAINAIGKDSKLGISPKVCEFLRNE  511
             +  P+   QI +RGDD  ++S     +  ++L Y A+ A   +SK  +     EFLR E
Sbjct  644  TFSRPMETWQI-VRGDDTQVVSDHYLDVLAVKLGYDALGAEANESKFTLRQGRTEFLRVE  702

Query  512  ISVTG--VRGWTCRGIGGISQRKPWNPQPWSPNDEVETNASNISLLERRA--GIELQQLH  567
               TG   R + CR I  + QRKPW+ +  S +  +        +L RR     +LQ
Sbjct  703  ---TGDRARAYPCRTIPLLGQRKPWSARSPSADASLTRIDKVFCVLARRLPDPSDLQGFA  759

Query  568  HINKVKWSRHVRQSYKYLELPKRLGGFGIYRFQG------WLPNGKLPLAKKPLVNVEDI  621
                 +    ++  ++ + +P  LGG G+  + G      W  N +LP+  + ++N  D
Sbjct  760  DHVMHRMMSKMKLDFRLIRIPVGLGGLGLRPWDGRHHVAVW--NSRLPVPVR-VLNQTDF  816

Query  622  HPSQE------LFLPLSEQQKKILAQVEMTNKMQTDDI  653
             P+Q+      + + ++E + + LA  E+  KM TDD+
Sbjct  817  RPAQQQKKYAGIGVAITEGESRALADREVREKMATDDM  854


>MAG: RNA-dependent RNA polymerase, partial [Totiviridae sp.]
Sequence ID: WAK77747.1 Length: 1048
Range 1: 230 to 854

Score:202 bits(514), Expect:3e-55,
Method:Compositional matrix adjust.,
Identities:172/632(27%), Positives:293/632(46%), Gaps:41/632(6%)

Query  56   DKELQTFYVPRSNADLVIRRIRLID-----LWRNLKPDQMDEIRNYTHLDYIFVQNICIY  110
            D   Q  + PR+ AD+ IR +R+ D     L  + +    D +R     DYI   N+ I
Sbjct  230  DTLAQELFPPRNEADMGIRNLRMADMLTYCLATDRRAAVTDVLRLCRGFDYILTCNLLIA  289

Query  111  MLVFGINTVKHFRQIGLFNERNE-FIEIAKQLSTKGKRFVDDVDNMKQKVC--EIATIVG  167
            M  FG       R+ G+F+  +E +    K LS   K+  + + +   +VC  E A + G
Sbjct  290  MFCFGDLWWTGLREAGVFDGDSEHYRTSCKALSDTVKKTRNTLSD-DDRVCFYECAALFG  348

Query  168  YMDPNVDKIDVMEEVNSLAAEG-NEHG--IDRDNWNDLFTKTCKEVMTWYKGH--EFISF  222
             M P V   D +EE   LA+ G  +HG  +  +   D      + ++ + +G   E  +F
Sbjct  349  AMCPPVPGWDPLEETRDLASGGQTDHGLVVGDELGPDAILGRIQALVCFPEGKRCEERTF  408

Query  223  DDYIKEGMWLTSGSSSIGKVHWTKDG----ENGKFKARKNMLLQIYTPQELANIVYAWDG  278
            +++++   W  SGSSS+G+V +  +       G FKARKN++L +    +L     + +
Sbjct  409  EEWLRSCEWERSGSSSVGRVEYEVEVEGERRKGHFKARKNLVLDVVPFDDLVKSTRSHNT  468

Query  279  KLHSRVFIKNEMSKLRLAVASNIEAYIHESYMLFLYGHGFKEYFGVTLDEKPDQQHQREI  338
            + + +  +K+E  K+RLAV++ +E Y+ + Y+  + G  +  + G TL+E   ++ +R
Sbjct  469  QTN-KALVKSEFGKIRLAVSAPLEVYLQQGYLYGVSGSAYLRWPGNTLEESVSEEMRRNE  527

Query  339  EMIEKLQAGYFGLPFDYASFDHQPTTFEVKTMVRRVGEIVVSQVPKNYYYQTQLLVNKIV  398
                ++  G F LP+D+A FDHQPTT EV    R   +  +           +L  N +
Sbjct  528  RTYTRMARGGFALPYDFARFDHQPTTEEVVAFQRITFDRALVNAKPTQRADVELFENLLE  587

Query  399  NAYDKSYLSGNIKNTKFENIKVKGGVPSGVRITSLLGNMWNAIITKIAINNVIGIIGYD-  457
              +  + L+      +     V GG+ SG+R TS +G+ WN+++ + A +       Y
Sbjct  588  QGFRTATLTTPPGICQPTTFPVTGGLMSGLRSTSAVGSGWNSVLGESARDMADRFRTYTR  647

Query  458  PIS--QISLRGDDVAILSKDPAALYLLRLSYAAINAIGKDSKLGISPKVCEFLRNEISVT  515
            P+   QI +RGDD  ++S     +  +++ Y A+ A   +SK  +     EFLR E S
Sbjct  648  PVDTWQI-VRGDDTQVVSDHYLDVLAIKVGYDALGAEANESKFTLRRGRTEFLRVETS-D  705

Query  516  GVRGWTCRGIGGISQRKPWNPQPWSPNDEVETNASNISLLERRAGIELQQLHHINKV--K  573
              R + CR I  + QRKPW+ +P S +  +       S+L RR           + V  +
Sbjct  706  RARAYPCRTIPLLEQRKPWSARPPSGDASLVRVDKVFSVLARRLPDPRDLRGFADHVIRR  765

Query  574  WSRHVRQSYKYLELPKRLGGFGIYRFQG------WLPNGKLPLAKKPLVNVEDIHPSQEL  627
                +R   + + +P  LGG G+  ++G      W     +P+A   + N  D    QEL
Sbjct  766  MMVKMRLDMRLVSIPTALGGLGLRPWRGAWHVSVWSSQLPVPIA---VTNQTDFRAGQEL  822

Query  628  ------FLPLSEQQKKILAQVEMTNKMQTDDI  653
                   + +S  +   LA   +  KM TDD+
Sbjct  823  DRYAGMGVKISGGEATALADRSVREKMATDDM  854


>MAG: RNA-dependent RNA polymerase, partial [Totiviridae sp.]
Sequence ID: WAK77284.1 Length: 1105
Range 1: 472 to 1090

Score:202 bits(513), Expect:5e-55,
Method:Compositional matrix adjust.,
Identities:174/633(27%), Positives:291/633(45%), Gaps:58/633(9%)

Query  66    RSNADLVIRRIRLIDLW-----RNLKPDQMDEIRNYTHLDYIFVQNICIYMLVFGINTV-  119
             RS+ DL IR  R  D+W        + + +D +    H DYI + N+    LVF +N V
Sbjct  472   RSSQDLGIRYARAADVWTYYLDHGRRQELIDTLHQLKHFDYITMVNLLTAKLVF-VNPVW  530

Query  120   -KHFRQIGLFNERNEFIEIA-KQLST--KGKRFVDDVDNMKQKVCEIATIVGYMDPNVDK  175
                  + G      E   +A + LS   K +R     D+ +    E+A++ G M P V
Sbjct  531   WAALERAGCLRAGAEHFRLAGRALSDLLKKERVALPPDD-RVAFYEVASLYGAMCPPVPG  589

Query  176   IDVMEEVNSLAAEG-NEHGI--------DRDNWNDLFTKTCKEVMTWYKGHEFISFDDYI  226
              D ++E  +LA  G ++HG+           ++++   +            +   F  ++
Sbjct  590   WDPIDETQALAQGGQDQHGLLLNAQPGYSSADFHNAIRRLAAARPR--DDPDPRPFRTWL  647

Query  227   KEGMWLTSGSSSIGKVHWT----KDGENGKFKARKNMLLQIYTPQELANIVYAWDGKLHS  282
              +  W  SG+SS+G+VH+T     +   GKFKARKN++L +    EL      +  +  +
Sbjct  648   WDEEWARSGASSLGRVHYTLSVGDEVRAGKFKARKNLVLDVIPIDELEERTRRYTIQ-EN  706

Query  283   RVFIKNEMSKLRLAVASNIEAYIHESYMLFLYGHGFKEYFGVTLDEKPDQQHQREIEMIE  342
                +K E++K+RLAV+S IE Y+ ++++  + G+ +  +   TL+E    +  R  E
Sbjct  707   VALVKTELAKIRLAVSSPIETYLAQAWIFLITGNAYANWPANTLEEPLHAELARLEETWL  766

Query  343   KLQAGYFGLPFDYASFDHQPTTFEV---KTMVRRVGEIVVSQVPKNYYYQTQLLVNKIVN  399
             +L+AG F LP+D+A FDHQPTT EV   +T     G    +      Y Q   L   +
Sbjct  767   RLRAGQFSLPYDFARFDHQPTTSEVTAFQTATNMCGRRFATPEQSEDYDQ---LTRNVTL  823

Query  400   AYDKSYLSGNIKNTKFENIKVKGGVPSGVRITSLLGNMWNAIITKIAINNVIGIIGYDPI  459
              Y  + L+           +V GG+ SG+R TS +G+ WNAI  + A   +  I   D
Sbjct  824   GYSHATLTSPPGLGDQRTFRVTGGLMSGLRSTSCVGSGWNAIFGEFARQLIFDIRHPDSP  883

Query  460   SQI--SLRGDDVAILSKDPAALYLLRLSYAAINAIGKDSKLGISPKVCEFLRNEISVTGV  517
               +   +RGDD  +       +  ++L Y A+ AI  +SK  +     EFLR E     +
Sbjct  884   PNVWQLVRGDDTQVCGASYHDVLAIKLGYDALGAIANESKFTLRRGRTEFLRVETE-DRL  942

Query  518   RGWTCRGIGGISQRKPWNPQPWSPNDEVETNASNISLLERR----AGIELQQLHHINKVK  573
             RG+ CR I  +SQR+PWN +P      +E     +S L  R     G++  + H+I +
Sbjct  943   RGYPCRTIPLLSQRRPWNARPVLQEAGLEHVLKTMSTLRLRIADPTGLDSYREHYIQRC-  1001

Query  574   WSRHVRQSYKYLELPKRLGGFGIYRFQG------WLPNGKLPLAKKPLVNVEDIHPSQ--  625
               R +    +   +P  +GG G+  + G      W      P+    +VN  D    +
Sbjct  1002  -FRLLGFDARLRRIPAAMGGLGLDPWDGRWAVATWRSVNAPPVK---IVNRTDYRRQRVT  1057

Query  626   ELF----LPLSEQQKKILAQVEMTNKMQTDDIP  654
             E F    +P++ Q+ +++A+  +  K+  DD+P
Sbjct  1058  EEFAKAGIPVTVQETEVMAEKRIQAKVAADDVP  1090


>MAG: RNA-dependent RNA polymerase [Totiviridae sp.]
Sequence ID: WAK77254.1 Length: 854
Range 1: 31 to 684

Score:199 bits(507), Expect:9e-55,
Method:Compositional matrix adjust.,
Identities:175/663(26%), Positives:304/663(45%), Gaps:45/663(6%)

Query  56   DKELQTFYVPRSNADLVIRRIRLID-LWRNLKPDQMDEIRNYTHL----DYIFVQNICIY  110
            DK  Q  + PR+  D+ IR IR+ D L       + D  R    L    DYI  QN+
Sbjct  31   DKLAQELFPPRTKTDMGIRNIRMADALTYCAVTGRSDAARRVLKLCKGFDYITTQNLLTA  90

Query  111  MLVFGINTVKHFRQIGLFNERNEFIEIAKQLSTKGKRFVDDVDNMKQKVC--EIATIVGY  168
            +  F  +  +     G+F+  +E   ++ ++ +   +   +     ++VC  E A + G
Sbjct  91   LFCFNEDWWRTLSNAGVFDGDSEHYRLSCKVLSDLVKKTKNCLPSDERVCYYECAALFGA  150

Query  169  MDPNVDKIDVMEEVNSLAAEG-NEHGI--DRDNWNDLFTKTCKEVMTWY--KGHEFISFD  223
            M P V   D ++E   LA+ G  EHG+    +       K  KE+  +   K  +   F+
Sbjct  151  MCPPVAGWDPVQETKELASGGQTEHGLLFGEEFGPGSILKRIKELARFEGEKPSDGRGFE  210

Query  224  DYIKEGMWLTSGSSSIGKVHWT--KDGEN--GKFKARKNMLLQIYTPQELANIVYAWDGK  279
            D+++   W  SG+SS+G+V +    DGE   G FKARKN++L +   Q++ +   +   +
Sbjct  211  DWLRSCEWERSGASSVGRVEYEVEADGEQRKGSFKARKNLVLDVVPFQQIVDSTRSHASQ  270

Query  280  LHSRVFIKNEMSKLRLAVASNIEAYIHESYMLFLYGHGFKEYFGVTLDEKPDQQHQREIE  339
              ++  IK+E  K+RLAV++ +E Y+ + ++  + G  +  + G TL+E    + QR
Sbjct  271  -DNKALIKSEFGKVRLAVSAPLEVYLQQGFLYAVSGSAYLRWPGNTLEEAVADEMQRNER  329

Query  340  MIEKLQAGYFGLPFDYASFDHQPTTFEVKTMVRRVGEIVVSQVPKNYYYQTQLLVNKIVN  399
             +  +  G+F LP+D+A FDHQPTT EV        +  ++Q          L  + +
Sbjct  330  TLLAMSRGHFALPYDFARFDHQPTTDEVVAFQSITFDRALTQARPFQRDDVILFEDLLEQ  389

Query  400  AYDKSYLSGNIKNTKFENIKVKGGVPSGVRITSLLGNMWNAIITKIA--INNVIGIIGYD  457
             +  + L+        +   V GG+ SG+R TS +G+ WN+++ + A  I +     G
Sbjct  390  GFRSATLTTPPGVCAPQTFPVTGGLMSGLRSTSAVGSGWNSVLGESARDIASRFRSRGRP  449

Query  458  PISQISLRGDDVAILSKDPAALYLLRLSYAAINAIGKDSKLGISPKVCEFLRNEISVTGV  517
              +   +RGDD  ++S +   +  +++ Y  + A   +SK  +     EFLR EI
Sbjct  450  VDTWQIVRGDDTQVISPNYLDVLAVKIGYDVLGAEANESKFTLRRGRTEFLRVEIE-DRA  508

Query  518  RGWTCRGIGGISQRKPWNPQPWSPNDEVETNASNISLLERRAGIELQQLHHINKVKWSRH  577
            R + CR +  + Q KPW+ +  + +  +       S+L RR       L   + V  SR
Sbjct  509  RCYPCRTVPLVGQSKPWSARAPTADASLTRLDKVFSVLARRLPDPRDLLAFADHV-LSRM  567

Query  578  V---RQSYKYLELPKRLGGFGIYRFQG------WLPNGKLPLAKKPLVNVEDIHPSQE--  626
            V   R   + + +P  LGG G+  + G      W   G+LP+  K ++N       Q+
Sbjct  568  VSMMRLDVRLISIPTALGGLGLRPWGGKWHVSVW--AGRLPIPVK-VLNQTTFRTDQQYE  624

Query  627  ----LFLPLSEQQKKILAQVEMTNKMQTDD---IPGTQKLFSKEWIQKVRAKKIIWSRNQ  679
                + + LS  + + LA  E+  K+ TDD   I GT +   +  + +   +KI+ S
Sbjct  625  KYRSIGVNLSGVEARTLADRELREKIATDDMIEIAGTVRRVRRAELAR---RKIVSSGTA  681

Query  680  TIP  682
              P
Sbjct  682  GAP  684


>MAG: RNA-dependent RNA polymerase [Totiviridae sp.]
Sequence ID: WAK77427.1 Length: 926
Range 1: 103 to 727

Score:199 bits(505), Expect:2e-54,
Method:Compositional matrix adjust.,
Identities:174/635(27%), Positives:293/635(46%), Gaps:47/635(7%)

Query  56   DKELQTFYVPRSNADLVIRRIRLID-LWRNLKPDQMDEIRNYTHL----DYIFVQNICIY  110
            D   Q  Y PR+ AD+ IR +R+ D L   L   +    R    L    DYI   N+
Sbjct  103  DSLAQALYPPRNEADMGIRNLRMADMLTYCLATSRRARAREVLALCRGFDYILTSNVLTA  162

Query  111  MLVFGINTVKHFRQIGLFNERNE-FIEIAKQLSTKGKRFVDDVDNMKQKVC--EIATIVG  167
            +  FG    +     G+F+  +E +    K LS   K+  + + +   +VC  E A++ G
Sbjct  163  VFCFGDLWWQGLTNAGVFDGDSEHYRTCCKTLSDTIKKTKNTLSD-DDRVCFYECASLFG  221

Query  168  YMDPNVDKIDVMEEVNSLAAEG-NEHGIDRDNWNDLFTKT-----CKEVMTWYKGHEFIS  221
             M P V   D ++E   LA+ G + HG+    W +  T +      + ++ + +G +  +
Sbjct  222  AMCPPVPGWDPVQETRDLASGGQHPHGL---LWGEELTPSDILGRIQGLVRYKEGKKCDT  278

Query  222  --FDDYIKEGMWLTSGSSSIGKVHWTK--DGEN--GKFKARKNMLLQIYTPQELANIVYA  275
              F++++    W  SGSSSIG+V +    DGE   G FKARKN++L +    +L     +
Sbjct  279  RPFEEWLSSCEWERSGSSSIGRVEYEVEVDGERRKGHFKARKNLVLDVVPFDDLVRSTRS  338

Query  276  WDGKLHSRVFIKNEMSKLRLAVASNIEAYIHESYMLFLYGHGFKEYFGVTLDEKPDQQHQ  335
             + + + +  +K+E  K+RLAV++ +E Y+ + Y+  + G  +  + G TL+E   ++ +
Sbjct  339  HNTQTN-KALVKSEFGKIRLAVSAPLEVYLQQGYLYGVSGSAYLRWPGNTLEESVAEEMR  397

Query  336  REIEMIEKLQAGYFGLPFDYASFDHQPTTFEVKTMVRRVGEIVVSQVPKNYYYQTQLLVN  395
            R      ++  G F LP+D+A FDHQPTT EV    R   +  +            L  N
Sbjct  398  RNERTYTRMARGGFALPYDFARFDHQPTTEEVVAFQRITFDRALVNAKPTQRADVVLFEN  457

Query  396  KIVNAYDKSYLSGNIKNTKFENIKVKGGVPSGVRITSLLGNMWNAIITKIAINNVIGIIG  455
             +   +  + L+      +     V GG+ SG+R TS +G+ WN+++ + A +
Sbjct  458  LLEQGFRTATLTTPPGICQPTTFPVTGGLMSGLRSTSAVGSGWNSVLGESARDMADRFRT  517

Query  456  YD-PIS--QISLRGDDVAILSKDPAALYLLRLSYAAINAIGKDSKLGISPKVCEFLRNEI  512
            Y  P+   QI +RGDD  ++S     +  +++ Y A+ A   +SK  +     EFLR E
Sbjct  518  YTRPVDTWQI-VRGDDTQVVSDYYLDVLAVKVGYDALGAEANESKFTLRRGRTEFLRVET  576

Query  513  SVTGVRGWTCRGIGGISQRKPWNPQPWSPNDEVETNASNISLLERRAGIELQQLHHINKV  572
            S    R + CR I  + QRKPW+ +P S +  +       S+L RR           + V
Sbjct  577  S-DRARAYPCRTIPLLEQRKPWSARPPSGDASLVRVDKVFSVLARRLPDPRDLRGFADHV  635

Query  573  KWSRHVRQ--SYKYLELPKRLGGFGIYRFQG------WLPNGKLPLAKKPLVNVEDIHPS  624
                 V+     + + +P  LGG G+  ++G      W     +P+A   + N  D   +
Sbjct  636  IRRMMVKMQLDMRLISIPTALGGLGLRPWRGAWHVSVWSSQLPVPIA---VTNQTDFRAN  692

Query  625  QEL------FLPLSEQQKKILAQVEMTNKMQTDDI  653
            QEL       + +S  +   LA   +  KM TDD+
Sbjct  693  QELDRYAGMGVKISGGEAIALADRGVREKMATDDM  727


>MAG: putative RNA-dependent RNA polymerase, partial [Totiviridae sp.]
Sequence ID: UHS72484.1 Length: 426
Range 1: 164 to 425

Score:190 bits(482), Expect:3e-54,
Method:Compositional matrix adjust.,
Identities:101/263(38%), Positives:157/263(59%), Gaps:1/263(0%)

Query  16   LTKGIYNASTLTPKIVDQIIKDEGSGTDKTKYVNVPKIITDKELQTFYVPRSNADLVIRR  75
            LTKG++ A  +        + +     D T + ++     D +LQ  + PR   D+ +RR
Sbjct  164  LTKGLFRADEMDWFDALVWVGENVGEKDSTTHKDI-DFSHDSKLQDVFRPRIKLDMSVRR  222

Query  76   IRLIDLWRNLKPDQMDEIRNYTHLDYIFVQNICIYMLVFGINTVKHFRQIGLFNERNEFI  135
            +R+ DL R    D   E+  YTHLDYIF  NI +Y +V G++ +   R  GLFN+ + F+
Sbjct  223  MRMRDLARVASDDLKFEMGYYTHLDYIFASNIMLYGIVHGVDVLVDLRLRGLFNDFDSFM  282

Query  136  EIAKQLSTKGKRFVDDVDNMKQKVCEIATIVGYMDPNVDKIDVMEEVNSLAAEGNEHGID  195
              A ++ST  KR V D+ ++KQ++ EI  ++GY+  +V+  +  +E+  LAA GN HG+
Sbjct  283  LKANKISTFMKRLVVDLGSVKQRLVEIGGLIGYLQNDVESWNTEDELKGLAAGGNLHGLI  342

Query  196  RDNWNDLFTKTCKEVMTWYKGHEFISFDDYIKEGMWLTSGSSSIGKVHWTKDGENGKFKA  255
             D+W  +F +  +++ T     +F++F+DY+K   W+TSGSSSIGKV W+ D + GKFKA
Sbjct  343  SDDWKVVFQEYMRKIQTVSDRSKFLTFEDYVKSDKWITSGSSSIGKVIWSYDDDTGKFKA  402

Query  256  RKNMLLQIYTPQELANIVYAWDG  278
            RKNM+  +Y+  EL  IV  WDG
Sbjct  403  RKNMVQDLYSKDELWRIVSGWDG  425


>MAG: RNA-dependent RNA polymerase [Totiviridae sp.]
Sequence ID: WAK77278.1 Length: 952
Range 1: 129 to 753

Score:198 bits(504), Expect:4e-54,
Method:Compositional matrix adjust.,
Identities:168/639(26%), Positives:294/639(46%), Gaps:55/639(8%)

Query  56   DKELQTFYVPRSNADLVIRRIRLIDLW--------RNLKPDQMDEIRNYTHLDYIFVQNI  107
            D   QT + PR+ AD+ IR +R+ D+         RN   D +   R Y   DYI   N+
Sbjct  129  DTLAQTLFPPRNEADMGIRNLRMADMLTFGLVTGRRNRLLDILRLCRGY---DYIITCNL  185

Query  108  CIYMLVFGINTVKHFRQIGLFNERNEFIEIAKQLSTKGKRFVDDVDNMKQKVC--EIATI  165
             I +  F     +  +  G+F+   E   ++ +  +   +   +V +   +VC  E A++
Sbjct  186  LIALFCFDDLWWQILQDSGVFDGDAEHFRVSCKCLSDTVKKTKNVLSDDDRVCLYECASL  245

Query  166  VGYMDPNVDKIDVMEEVNSLAAEGN-EHGI--DRDNWNDLFTKTCKEVMTWYKGHEF--I  220
             G M P V   D + E   LA+ G  +HG+    +  +D   +    ++ + +G +
Sbjct  246  YGAMCPPVPGWDPLSETRDLASGGQVDHGLLLGDEFSSDEILRRIHALVRFPEGKKCDDR  305

Query  221  SFDDYIKEGMWLTSGSSSIGKVHWTK--DGEN--GKFKARKNMLLQIYTPQELANIVYAW  276
             F+++++   W  SGSSS+G+V +    DGE   G FKARKN++L +    EL   V
Sbjct  306  GFEEWLRSCDWERSGSSSLGRVEYEVEVDGERRKGHFKARKNLVLDVVPYDELLTSVRTH  365

Query  277  DGKLHSRVFIKNEMSKLRLAVASNIEAYIHESYMLFLYGHGFKEYFGVTLDEKPDQQHQR  336
            D +  ++  +K+E  K+RLAV++ +E Y+ + Y+  + G  +  + G TL+E   ++ +R
Sbjct  366  DTQ-SNKALVKSEFGKVRLAVSAPLEVYLQQGYLYSVSGSAYLHWPGNTLEESVGEEMRR  424

Query  337  EIEMIEKLQAGYFGLPFDYASFDHQPTTFEVKTMVRRVGEIVVSQVPKNYYYQTQLLVNK  396
              +   ++  G F LP+D+A FDHQPTT EV T  R   +  +           +L  +
Sbjct  425  NEQTFMRMAKGEFALPYDFARFDHQPTTEEVVTFQRITFDRALVNSRPVQRADVELFESL  484

Query  397  IVNAYDKSYLSGNIKNTKFENIKVKGGVPSGVRITSLLGNMWNAIITKIAINNVIGIIGY  456
            +   +  + L+            V GG+ SG+R TS +G+ WN+++ + A +       +
Sbjct  485  LEKGFRTATLTTPPGICTPTTFPVTGGLMSGLRSTSAVGSGWNSVLGESARDMAGRFRAH  544

Query  457  DPISQI--SLRGDDVAILSKDPAALYLLRLSYAAINAIGKDSKLGISPKVCEFLRNEISV  514
                +    +RGDD  ++S     +  ++L Y A+ A   +SK  +     EFLR E
Sbjct  545  TRAVETWQIVRGDDTQVVSDHYLDVLAVKLGYDALGAEANESKFTLRRGRTEFLRVETGD  604

Query  515  TGVRGWTCRGIGGISQRKPWNPQPWSPNDEVETNASNISLLERRAGIELQQLHHINKVKW  574
               R + CR +  + QRKPW  +P S +  +       S+L RR        H  + +++
Sbjct  605  RS-RAYPCRTVPLLEQRKPWTARPPSGDASLVRIDKVFSVLSRRLP------HPEDIMRF  657

Query  575  SRHVRQ--------SYKYLELPKRLGGFGIYRFQG------WLPNGKLPLAKKPLVNVED  620
            + HV +          + L +P  LGG G   + G      W  + +LP+  + ++N
Sbjct  658  ADHVLRRMMAKMGLDLRLLSIPTALGGLGRRPWGGRWHVSVW--SSRLPVPVR-VLNRTG  714

Query  621  IHPSQE------LFLPLSEQQKKILAQVEMTNKMQTDDI  653
                QE      L + +S  +   LA   +  K+ TDD+
Sbjct  715  FRTEQERLRYQALGVSISTGEADSLADRSVREKLATDDM  753


>MAG: RNA-dependent RNA polymerase [Totiviridae sp.]
Sequence ID: WAK77616.1 Length: 830
Range 1: 7 to 570

Score:197 bits(500), Expect:5e-54,
Method:Compositional matrix adjust.,
Identities:171/581(29%), Positives:278/581(47%), Gaps:52/581(8%)

Query  56   DKELQTFYVPRSNADLVIRRIRLID-LWRNLKPDQMDEIRNYTH----LDYIFVQNICIY  110
            D   +T Y PR+  D+ IR  R+ D L    K  Q +E+R         DY+   N+ +
Sbjct  7    DALAKTLYPPRTREDMAIRVCRIGDVLGFAKKVGQAEEVRAVLRACRGFDYVSTNNLLVA  66

Query  111  MLVFGINTVKHFRQIGLFN-ERNEFIEIAKQLS-TKGKRFVDDVDNMKQKVCEIATIVGY  168
             L F           GL + +   F    + LS T  KR     D  +  + E A++ G
Sbjct  67   RLCFKDTWWDLVSGSGLLDSDATHFRAAGRTLSDTVKKRDTVLGDYDRVSLYECASLYGA  126

Query  169  MDPNVDKIDVMEEVNSLAAEG-NEHGI-----DRDNWNDLFTKTCKEVMTWYKGHEFISF  222
            M P V   D ++E   LA+ G +EHG+      R N + L            K H+  S
Sbjct  127  MCPPVPGWDPVQETRDLASGGQDEHGLLWGPKYRPN-SVLLAIADLARYPEAKPHDTRSL  185

Query  223  DDYIKEGMWLTSGSSSIGKVHW-TKDGEN---GKFKARKNMLLQIYTPQELANIVYAWDG  278
            + ++    W  SGSSS+G+V +   DG+    G+FKARKN++L I  P EL   V   D
Sbjct  186  EAWLLACDWERSGSSSMGRVEYEVFDGDTWRRGRFKARKNLVLDIIEPSELLEAVRTHDT  245

Query  279  KLHSRVFIKNEMSKLRLAVASNIEAYIHESYMLFLYGHGFKEYFGVTLDEKPDQQHQREI  338
            +  +R  IK+E  K+RLAV++ +  Y+ ++Y+  + G  +  + G TL+E  +Q+  R
Sbjct  246  Q-DNRALIKSEFGKIRLAVSAPLPTYLQQAYLYSVAGCPYLNWPGNTLEESLEQEMLRNE  304

Query  339  EMIEKLQAGYFGLPFDYASFDHQPTT-----FEVKTMVRRVGEIVVSQVPKNYYYQTQLL  393
              +  +++G+F LP+D+A FDHQPTT     F+  T  R +      Q+      +  L
Sbjct  305  RTLCLMRSGFFALPYDFARFDHQPTTAQVVEFQKLTFTRALLNANPWQIEDVRRIELLLE  364

Query  394  VNKIVNAYDKSYLSGNIKNTKFENIKVKGGVPSGVRITSLLGNMWNAIITKIA---INNV  450
            V     A+D + L+      +    KV GG+ SG+R TS +G+ WN+++ +++    N++
Sbjct  365  V-----AFDNATLTTPPGVCEPMTFKVTGGLMSGLRSTSAVGSGWNSVLGEMSRSIANDL  419

Query  451  IGIIGYDPISQISLRGDDVAILSKDPAALYLLRLSYAAINAIGKDSKLGISPKVCEFLRN  510
             G        QI +RGDD  ++S+    +  +++ Y A+ A   +SK  +     EFLR
Sbjct  420  RGPGAKLETWQI-VRGDDTQVISRSYLDVLAVKIGYDALGAEANESKFTLRRGRTEFLRV  478

Query  511  EISVTG--VRGWTCRGIGGISQRKPWNPQPWSPNDEVETNASNISLLERRAGIELQQLHH  568
            E   TG   R + CR +  ++QRKPW+ +P S +         I++L RR    ++
Sbjct  479  E---TGDVARCYPCRTVPLVNQRKPWSGRPDSDDASAARVVKVINVLSRR----VEDPRT  531

Query  569  INKVKWSRHVRQ--------SYKYLELPKRLGGFGIYRFQG  601
            I  + + R+V +          + L +P  LGG G+Y + G
Sbjct  532  I--LDFGRYVAEKMVAKKGVDVRLLSIPTSLGGLGLYPWDG  570


>MAG: RNA-dependent RNA polymerase, partial [Totiviridae sp.]
Sequence ID: WAK77676.1 Length: 637
Range 1: 106 to 607

Score:194 bits(492), Expect:6e-54,
Method:Compositional matrix adjust.,
Identities:154/524(29%), Positives:254/524(48%), Gaps:63/524(12%)

Query  56   DKELQTFYVPRSNADLVIRRIRLIDLWRN-LKPDQMDEIRNYTHL----DYIFVQNICIY  110
            D+     +  R++ DL IR  R  D+W   +   +  E+R   HL    DYI + N+
Sbjct  106  DRYALAAFPARTSQDLGIRYARAADIWSFFISRGRQQELREDLHLLKAFDYITMVNVLSA  165

Query  111  MLVFGINTV-KHFRQIGLFNERNEFIEIA-KQLS--TKGKRFVDDVDNMKQKVCEIATIV  166
             +VF  +      ++ G F+   E   +A + LS   K +    D D+ +    E+A++
Sbjct  166  KIVFTRDLWWATLKRAGCFSGGAEHFRLAARSLSDIVKKENVSLDPDD-RVSFYEVASLY  224

Query  167  GYMDPNVDKIDVMEEVNSLAAEG-NEHGIDRDNWNDLFTKTCKEVMTWYKGHEFIS----  221
            G M P V   D +EE  +LA  G ++HG+           +C      Y   +F++
Sbjct  225  GTMCPPVPGWDPIEETQALAQGGQDQHGL---------LLSCAPG---YSSQDFVAAVRA  272

Query  222  --------------FDDYIKEGMWLTSGSSSIGKVHWTKD-GE---NGKFKARKNMLLQI  263
                          F D++ +  W  SG+SS+G+V +  + GE   +GKFKARKN++L +
Sbjct  273  LAYVPPRDAPDGRPFRDWLWDEEWARSGASSLGRVEYALELGEEVKSGKFKARKNLVLDV  332

Query  264  YTPQELANIVYAWDGKLHSRVFIKNEMSKLRLAVASNIEAYIHESYMLFLYGHGFKEYFG  323
                EL     ++  +  +   IK E++K+RLAV+  IE Y+ ++++  + G+ +  +
Sbjct  333  IPIDELEQRTRSYTQQ-ENVALIKTELAKIRLAVSCPIETYLAQAWIYLVSGNAYTNWPA  391

Query  324  VTLDEKPDQQHQREIEMIEKLQAGYFGLPFDYASFDHQPTTFEVKTMVRRVGEIVVSQVP  383
             TL+E    +  R  E  E+LQ G + LP+D+A FDHQPTT EVK       ++  +
Sbjct  392  NTLEEPLPVEVARHEETWERLQKGEYSLPYDFARFDHQPTTSEVKAF-----QVATNICG  446

Query  384  KNYYYQTQL-----LVNKIVNAYDKSYLSGNIKNTKFENIKVKGGVPSGVRITSLLGNMW  438
            + Y    QL     L   I   YD + L+        +  +V GG+ SG+R TS +G+ W
Sbjct  447  RRYAVPEQLADYDQLAYNITVGYDHATLTSPPGLGDQQTFRVTGGLMSGLRSTSCVGSGW  506

Query  439  NAIITKIAINNVIGIIGYDPISQI--SLRGDDVAILSKDPAALYLLRLSYAAINAIGKDS  496
            NAI  + A   +  I   D  +++   +RGDD  +  +    +  +++ Y A+ AI  +S
Sbjct  507  NAIFGEFARKMINDIRNPDRPARVWQLVRGDDTQVTGESYHDVLAIKIGYDAMGAIANES  566

Query  497  KLGISPKVCEFLRNEISVTG--VRGWTCRGIGGISQRKPWNPQP  538
            K  +     EFLR E   TG  +RG+ CR I  ++QR+PWN +P
Sbjct  567  KFTLRQGRTEFLRIE---TGDRLRGYPCRTIPLLTQRRPWNARP  607


>RNA-dependent RNA polymerase [Yanbian Totiv tick virus 1]
Sequence ID: XFO99082.1 Length: 945
Range 1: 126 to 747

Score:197 bits(500), Expect:1e-53,
Method:Compositional matrix adjust.,
Identities:171/634(27%), Positives:290/634(45%), Gaps:54/634(8%)

Query  63   YVPRSNADLVIRRIRLIDLW-----RNLKPDQMDEIRNYTHLDYIFVQNICIYMLVFGIN  117
            + PR+N DL IR  R  D+W        + + +  +R   H DYI + N+    ++F
Sbjct  126  FPPRTNQDLGIRYARAADVWTFYMAHGRRAELVTTLRLLKHFDYITMVNVLTAKIMFTRP  185

Query  118  TV-KHFRQIGLFNERNEFIEIAKQL--STKGKRFVDDVDNMKQKVCEIATIVGYMDPNVD  174
                   + G F   +E   +A +    T  K  V    + +    E A++ G M P V
Sbjct  186  VWWMTMERAGCFAAGSEHFRLAARALSDTIKKEKVALSPDDRVAFYESASLYGAMCPPVP  245

Query  175  KIDVMEEVNSLAAEG-NEHGIDRDNWNDLFTKTCKEVMTWYKGHEFI---------SFDD  224
              D +EE  +LA  G ++HG+  D      T    E +   +   F+         +F
Sbjct  246  GWDPVEESRALAEGGQDQHGLLLDCCPGYSTLDFHEAV---RKLAFVQPRDTPDSRTFRK  302

Query  225  YIKEGMWLTSGSSSIGKVHWT----KDGENGKFKARKNMLLQIYTPQELANIVYAWDGKL  280
            ++ +  W  SG+SS+G+V +      +   GKFKARKN++L + +  EL      +  K
Sbjct  303  WLWDEEWARSGASSLGRVEYRLRMGDEVRTGKFKARKNLVLDVVSIDELERRTRTYT-KQ  361

Query  281  HSRVFIKNEMSKLRLAVASNIEAYIHESYMLFLYGHGFKEYFGVTLDEKPDQQHQREIEM  340
             +   IK E++K+RLAV+  IE Y+ ++++  + G+ +  +   TL+E    +  R  E
Sbjct  362  ENVALIKTELAKIRLAVSCPIETYLAQAWIYLVTGNAYSNWPANTLEEPLHVEIARHEET  421

Query  341  IEKLQAGYFGLPFDYASFDHQPTTFEVKTMVRRVGEIVVSQVPKNYYYQTQL-----LVN  395
             E+LQ   + LP+D+A FDHQPTT EVK       +I  +   + Y    Q+     L
Sbjct  422  WERLQTKEYSLPYDFARFDHQPTTSEVKAF-----QIATNICGRRYARPEQMADYEQLSA  476

Query  396  KIVNAYDKSYLSGNIKNTKFENIKVKGGVPSGVRITSLLGNMWNAIITKIAINNVIGIIG  455
             +   YD + L+      + +   V GG+ SG+R TS +G+ WNA   + A   +  +
Sbjct  477  CVTQGYDHATLTSPPGLGEQKTFHVTGGLMSGLRSTSCVGSGWNATFGEFARRMIDEMRH  536

Query  456  YDPISQI--SLRGDDVAILSKDPAALYLLRLSYAAINAIGKDSKLGISPKVCEFLRNEIS  513
             D   Q+   +RGDD  +  +    +  +++ Y A+ AI  +SK  +     EFLR E
Sbjct  537  PDHPPQVWQLVRGDDTQVTGRSYHDVLAIKIGYDAMGAIANESKFTLRQGRTEFLRIETE  596

Query  514  VTGVRGWTCRGIGGISQRKPWNPQPWSPNDEVETNASNISLLERR----AGIELQQLHHI  569
               +RG+ CR I  ++QR+PWN +P      +E     ++ L  R     G++  + H+I
Sbjct  597  -DRLRGYPCRTIPMMTQRRPWNARPVVQEAGLEHVLKTMATLRLRLPTCEGLDAYRDHYI  655

Query  570  NKVKWSRHVRQSYKYLELPKRLGGFGIYRFQG-WLPNGKLPLAKKP--LVNVEDIHPSQ-  625
             +    R +R   +   +P  LGG G+  + G W       +   P  +VN  D    +
Sbjct  656  RRC--FRLLRLDSRLRNIPAALGGLGVDPWDGRWSVQTWQSIRPPPVEIVNRTDYREQRV  713

Query  626  -ELF----LPLSEQQKKILAQVEMTNKMQTDDIP  654
             E F    +P+  ++  ++A   +  K+  DD+P
Sbjct  714  AEEFAAVDVPVDAKEVTLMASERIRAKVAADDVP  747


>MAG: RNA-dependent RNA polymerase [Totiviridae sp.]
Sequence ID: WAK77722.1 Length: 809
Range 1: 3 to 610

Score:191 bits(484), Expect:5e-52,
Method:Compositional matrix adjust.,
Identities:172/618(28%), Positives:291/618(47%), Gaps:47/618(7%)

Query  73   IRRIRLID-LWRNLKPDQMDEIRNYTHL----DYIFVQNICIYMLVFGINTVKHFRQIGL  127
            IR +R+ D L   L   +        HL    DYI   N+   +  FG    +   Q G+
Sbjct  3    IRNLRMADMLTYCLATGRRARAAEVLHLCRGFDYILTSNVLTAIFCFGDLWWQGLTQAGV  62

Query  128  FNERNE-FIEIAKQLSTKGKRFVDDVDNMKQKVC--EIATIVGYMDPNVDKIDVMEEVNS  184
            F+  +E F    K LS   K+  + + +   +VC  E A++ G M P V   D ++E
Sbjct  63   FDGDSEHFRTSCKTLSDTIKKTKNTLSD-DDRVCFYECASLFGAMCPPVPGWDPVQETED  121

Query  185  LAAEG-NEHGI---DRDNWNDLFTKTCKEVMTWYKGHEFIS--FDDYIKEGMWLTSGSSS  238
            LA+ G + HG+   D     D+  +    ++ + +G    +  F++++    W  SGSSS
Sbjct  122  LASGGQHPHGLMIGDELTPEDILGRI-HALVRYREGKRCDTRPFEEWLASCEWERSGSSS  180

Query  239  IGKVHWTKDGEN----GKFKARKNMLLQIYTPQELANIVYAWDGKLHSRVFIKNEMSKLR  294
            IG+V +  + E     G FKARKN++L +    EL   V   D K  ++  +K+E  K+R
Sbjct  181  IGRVEYEVEVEEERRRGHFKARKNLVLDVVPFDELVASVRTHD-KQENKALVKSEFGKVR  239

Query  295  LAVASNIEAYIHESYMLFLYGHGFKEYFGVTLDEKPDQQHQREIEMIEKLQAGYFGLPFD  354
            LAV++ +E Y+ + Y+  + G  + ++ G TL+E   ++ +R      ++  G F LP+D
Sbjct  240  LAVSAPLEVYLQQGYLYGVSGSAYLKWPGNTLEESIGEEMRRNELTYTRMAQGGFALPYD  299

Query  355  YASFDHQPTTFEVKTMVRRVGEIVVSQVPKNYYYQTQLLVNKIVNAYDKSYLSGNIKNTK  414
            +A FDHQPTT EV T      +  +           +L    + + +  + L+      +
Sbjct  300  FARFDHQPTTDEVVTFQTITFDRALVNAHPPQRPDVELFERLLEHGFRHATLTTPPGICE  359

Query  415  FENIKVKGGVPSGVRITSLLGNMWNAIITKIAINNVIGIIGYD---PISQISLRGDDVAI  471
                 V GG+ SG+R TS +G+ WN+++ + A +       Y       QI +RGDD  +
Sbjct  360  PRTFPVTGGLMSGLRSTSAVGSGWNSVLGESARDMADRFRTYTRSVDTWQI-VRGDDTQV  418

Query  472  LSKDPAALYLLRLSYAAINAIGKDSKLGISPKVCEFLRNEISVTG--VRGWTCRGIGGIS  529
            +S     +  ++L Y A+ A   +SK  +     EFLR E   TG   R + CR I  +
Sbjct  419  VSDYYLDVLAVKLGYDALGAEANESKFTLRRGRTEFLRVE---TGDRARAYPCRTIPLLG  475

Query  530  QRKPWNPQPWSPNDEVETNASNISLLERRAGIELQQLHHINKVKWS--RHVRQSYKYLEL  587
            QRKPW+ +P S +  +       S+L RR       L   + V +     ++  ++ +++
Sbjct  476  QRKPWSARPPSADASLTRVDKVFSVLARRLPDPTDLLGFADHVMYRMMSKMKLDFRLIKI  535

Query  588  PKRLGGFGIYRFQG------WLPNGKLPLAKKPLVNVEDIHPSQ------ELFLPLSEQQ  635
            P  LGG G+  + G      W  N +LP+  + ++N  D    Q      ++ + LS+ +
Sbjct  536  PTALGGLGLRPWDGRNHVSVW--NSRLPVPVR-VLNQTDFRSGQLSRKYAKMGVQLSDGE  592

Query  636  KKILAQVEMTNKMQTDDI  653
             + LA  E+  KM TDD+
Sbjct  593  SQALADREVREKMATDDM  610


>MAG: RNA-dependent RNA polymerase [Totiviridae sp.]
Sequence ID: WAK77292.1 Length: 942
Range 1: 123 to 744

Score:191 bits(484), Expect:1e-51,
Method:Compositional matrix adjust.,
Identities:163/635(26%), Positives:289/635(45%), Gaps:56/635(8%)

Query  63   YVPRSNADLVIRRIRLIDLW-----RNLKPDQMDEIRNYTHLDYIFVQNICIYMLVFGIN  117
            + PR++ DL IR  R  D+W     +  K + M  +      DYI + N+    ++F
Sbjct  123  FPPRTSQDLGIRYARAADVWSFFIAQGQKKELMAILHQLKFFDYITMVNVLTAKIMFTRP  182

Query  118  TV-KHFRQIGLFNERNEFIEIAKQLST---KGKRFVDDVDNMKQKVCEIATIVGYMDPNV  173
               K   + G F    E   +A +  +   K ++   + D+ +    E A++ G M P V
Sbjct  183  IWWKTMDRAGCFAAGAEHFRLAARALSDIIKKEKVALEPDD-RVAFYECASLYGAMCPPV  241

Query  174  DKIDVMEEVNSLAAEG-NEHGIDRDNWNDLFTKTCKEVMTWYKGHEFI---------SFD  223
               D +EE  +LA  G ++HG+     N     + ++     +   F+          F
Sbjct  242  PGWDPVEESRALAEGGKDQHGL---LLNCSPGYSVEDFHLAVRQLAFVQPRDLPDPRPFR  298

Query  224  DYIKEGMWLTSGSSSIGKVHWT----KDGENGKFKARKNMLLQIYTPQELANIVYAWDGK  279
             ++ +  W  SG+SS+G+V ++     + + GKFKARKN++L +    EL      +  +
Sbjct  299  KWLWDEEWARSGASSLGRVEYSLKIGDETKTGKFKARKNLVLDVVPIDELEERTRTYT-R  357

Query  280  LHSRVFIKNEMSKLRLAVASNIEAYIHESYMLFLYGHGFKEYFGVTLDEKPDQQHQREIE  339
              +   IK E++K+RLAV+  IE Y+ ++++  + G+ +  +   TL+E    +  R  E
Sbjct  358  QDNVALIKTELAKIRLAVSCPIETYLAQAWIYLVTGNAYSNWPANTLEEPLHVEVARHEE  417

Query  340  MIEKLQAGYFGLPFDYASFDHQPTTFEVKTMVRRVGEIVVSQVPKNYYYQTQL-----LV  394
               +LQ G + LP+D+A FDHQPTT EVK       +I  +   + +    Q+     L
Sbjct  418  TWSRLQTGDYSLPYDFARFDHQPTTSEVKAF-----QIATNICGRRFATIDQISDYDQLS  472

Query  395  NKIVNAYDKSYLSGNIKNTKFENIKVKGGVPSGVRITSLLGNMWNAIITKIAINNVIGII  454
              +   YD + L+            V GG+ SG+R TS +G+ WNA   + A   +  +
Sbjct  473  QCVTAGYDHATLTSPPGLGVQRTFHVTGGLMSGLRSTSCVGSGWNATFGEFAKKMIDEMR  532

Query  455  GYDPISQI--SLRGDDVAILSKDPAALYLLRLSYAAINAIGKDSKLGISPKVCEFLRNEI  512
              D   ++   +RGDD  + S     +  +++ Y A+ AI  +SK  +     EFLR E
Sbjct  533  HPDNPPRVWQLVRGDDTQVTSPSFHDVLAIKIGYDAMGAIANESKFTLRQGRTEFLRIET  592

Query  513  SVTGVRGWTCRGIGGISQRKPWNPQPWSPNDEVETNASNISLLERR----AGIELQQLHH  568
                +RG+ CR I  ++QR+PWN +P      +E     ++ L  R     G++  + H+
Sbjct  593  E-DRLRGYPCRTIPMMTQRRPWNARPVVQEAGLEHVLKTMATLRLRLPSPEGLDAYRTHY  651

Query  569  INKVKWSRHVRQSYKYLELPKRLGGFGIYRFQG------WLPNGKLPLAKKPLVNVEDIH  622
            I +    R ++  ++   +P  LGG GI  + G      W P    P+      +  +
Sbjct  652  IARSM--RLLKFDHRLRNIPAALGGLGIDPWDGRWSVLTWRPVRPPPVEITNRTDYRERR  709

Query  623  PSQELF---LPLSEQQKKILAQVEMTNKMQTDDIP  654
              +E     +P+   + + +A+  +  K+  DD+P
Sbjct  710  VEEEFTAVNIPVLPDEVRQMAEARIQAKVAADDVP  744


>RNA-dependent RNA polymerase, partial [Totiviridae sp.]
Sequence ID: XCG98880.1 Length: 708
Range 1: 13 to 656

Score:187 bits(475), Expect:3e-51,
Method:Compositional matrix adjust.,
Identities:165/671(25%), Positives:314/671(46%), Gaps:73/671(10%)

Query  62   FYVPRSNADLVIRRIRLIDLWRNLKPDQMDE-----IRNYTHLDYIFVQNICIYMLVFGI  116
             +  R+  DL IR +R+ D+   +K   M +     +R     DY+   N  +  L F
Sbjct  13   LFPARTKEDLGIRNLRMGDMLLVMKRKGMTDKAHHVLRLCRGFDYVLTSNCLLAALCF--  70

Query  117  NTVKHFRQI----GLF-NERNEFIEIAKQLSTKGKRFVDDVD-NMKQKVCEIATIVGYMD  170
             T  ++  +    G+F ++   F    K+LS   K+  +++  + +  + E A++ G M
Sbjct  71   -TQDYWWSVMVASGVFLSDSEHFRAACKELSDTVKKTNNELTPDERVSLYECASLYGAMC  129

Query  171  PNVDKIDVMEEVNSLAAEGN-EHGIDRDNW------NDLFTK--TCKEVMTWYKGHEFIS  221
            P V   D +E+   LA  G   HG+    W      ND       C E    +   E  +
Sbjct  130  PPVPGWDPVEQTRDLAEGGQVPHGLLAGRWGTQGRLNDRIRDLVRCPE----HTSPEVRT  185

Query  222  FDDYIKEGMWLTSGSSSIGKVHW--TKDGEN--GKFKARKNMLLQIYTPQELANIVYAWD  277
            F+D++++  W  SG++++G+V +    +GE   G F+ARKN++L +   +E+       D
Sbjct  186  FEDWLRDCEWERSGAATLGRVEYELVVNGEERRGSFRARKNLVLDVVPIEEIVARTRNHD  245

Query  278  GKLHSRVFIKNEMSKLRLAVASNIEAYIHESYMLFLYGHGFKEYFGVTLDEKPDQQHQRE  337
             +  ++  IK+E  K+RLAV++ +E Y+ ++++  + G+ +  + G TL+E   ++  R
Sbjct  246  TQ-ENKALIKSEFGKIRLAVSAPLEVYLQQAFLFAVSGNSYLSWPGNTLEETLTEEMTRN  304

Query  338  IEMIEKLQAGYFGLPFDYASFDHQPTTFEV-----KTMVRRVGEIVVSQVPKNYYYQTQL  392
                 +++ G   LP+D+  FDHQP T E+      T  R +G   V Q      ++  L
Sbjct  305  ERTFVQMRRGANALPYDFQKFDHQPRTSEILCFQNVTFARALGAASVRQRDDVALFEALL  364

Query  393  LVNKIVNAYDKSYLSGNIKNTKFENIKVKGGVPSGVRITSLLGNMWNAIITKIA--INNV  450
                    + ++ ++           +V+GG+ SG+R TS +G+ WN+++ + +  + N
Sbjct  365  -----ERGFQQATITSPPGLGDQRTFRVRGGLMSGLRSTSAVGSGWNSVLGETSRDLANK  419

Query  451  IGIIGYDPISQISLRGDDVAILSKDPAALYLLRLSYAAINAIGKDSKLGISPKVCEFLRN  510
                G D  +   +RGDD  ++S     +  ++L Y  + A   +SK  + P   EFLR
Sbjct  420  FRGPGRDLDTWQIVRGDDTQVVSDSYLDVLAVKLGYDLLGAEANESKFTLRPGRTEFLRV  479

Query  511  EISVTGVRGWTCRGIGGISQRKPWNPQPWSPNDEVETNASNISLLERR---AGIELQQLH  567
            E S    R + CR +  + QR+PW+ +P + +  +       S+L RR   A + ++ +
Sbjct  480  ETSDRS-RAYPCRTVPLLFQRRPWSTRPPTGDASLTRVTKVASVLARRLTDASLLMEFVE  538

Query  568  HINKVKWSRHVRQSYKYLELPKRLGGFGIYRFQG------WLPNGKLPLAKKPLVNVEDI  621
             + +   S+ +    +  ++P  LGG G++ + G      W P+  +P+    + N
Sbjct  539  FVVERMLSK-MSVDLRLAKIPISLGGLGLFPWDGKWHVREWSPSPPIPIN---VTNSTGF  594

Query  622  HPSQ------ELFLPLSEQQKKILAQVEMTNKMQTDDIPGTQKLFSKEWIQKVRAKKIIW  675
               Q      E+ +P++  +   LA+  +  K+  DD+     L     +++VR  ++
Sbjct  595  RTGQEARRYAEMGVPITGGEAARLAERALRTKIAMDDV-----LELTGVVRRVRRAEL--  647

Query  676  SRNQTIPIHTD  686
             RN+TI + TD
Sbjct  648  -RNRTI-VRTD  656


>MAG: RNA-dependent RNA polymerase [Totiviridae sp.]
Sequence ID: WAK77583.1 Length: 870
Range 1: 45 to 692

Score:188 bits(477), Expect:7e-51,
Method:Compositional matrix adjust.,
Identities:161/656(25%), Positives:305/656(46%), Gaps:45/656(6%)

Query  56   DKELQTFYVPRSNADLVIRRIRLIDLW----RNLKPDQMDE-IRNYTHLDYIFVQNICIY  110
            D+ +   + PR+  DL IR +R  D+W     + + D++ E +R     DY+ + N+ +
Sbjct  45   DRNVIAAFPPRTPQDLGIRHVRASDVWSFYVESGRRDELVECLRQLRAFDYVSMVNLLLA  104

Query  111  MLVFGINTV-KHFRQIGLFNERNEFIEI-AKQLSTKGKRFVDDVDNMKQKVCEIATIVGY  168
             ++ G     +   + G F    E   + A+ LS   K+    +    +   E+A++ G
Sbjct  105  QVLIGGQVWWRTLERAGCFLAGAEHFRLRARTLSDLMKKENVALSADDRVSYEVASLYGA  164

Query  169  MDPNVDKIDVMEEVNSLA-AEGNEHGIDRDNWNDL----FTKTCKEVMTWYKGHEFISFD  223
            M P V   D +E+   LA   G+ HG+   ++ +     F     E+      H
Sbjct  165  MCPPVPGWDPVEKTRELAHGGGSSHGLVLGHYPNFDSQQFLAAIGELAC-VPPHSTPDIR  223

Query  224  DYIK---EGMWLTSGSSSIGKVHWTKDGENG----KFKARKNMLLQIYTPQELANIVYAW  276
            D+++   +  W  SG++S+G V +  D E G    +FKARKN++L +    EL     ++
Sbjct  224  DFVQWLWDEEWARSGAASLGYVEYELDDEEGQKRGRFKARKNLILDVIPFDELVARALSY  283

Query  277  DGKLHSRVFIKNEMSKLRLAVASNIEAYIHESYMLFLYGHGFKEYFGVTLDEKPDQQHQR  336
              +  +   IK E+SK+RLAV+S +E Y+ ++++  + GH +  + G TL+E    +  R
Sbjct  284  R-RQDNVALIKTELSKIRLAVSSPVETYLGQAWLFAVCGHAYLGWPGNTLEEPKYVEMAR  342

Query  337  EIEMIEKLQAGYFGLPFDYASFDHQPTTFEVKTMVRRVGEIVVSQVPKNYYYQTQLLVNK  396
                  +L+ G + LP+D+A FDHQPT+ EV      +      Q   +       L +
Sbjct  343  LETTWLRLRGGEYSLPYDFAEFDHQPTSSEVLAFQDCINRGAQRQALPSQRPHMDTLTSC  402

Query  397  IVNAYDKSYLSGNIKNTKFENIKVKGGVPSGVRITSLLGNMWNAIITKIAINNVIGIIGY  456
            +++ +  + L+           KV GG+ SG+R TS +G+ WN++  + A      + G
Sbjct  403  LLDGFRAATLTSPPGLGPQATFKVTGGLMSGLRSTSCVGSGWNSLFGEFARKMAEMVRGS  462

Query  457  DPISQI--SLRGDDVAILSKDPAALYLLRLSYAAINAIGKDSKLGISPKVCEFLRNEISV  514
                 +   +RGDD  ++ +    + ++++ Y A+ A   +SK  +     EFLR E
Sbjct  463  REPCDVWQVVRGDDTQVVGQSYHDVLVIKVGYDALGAKANESKFTLRRGRTEFLRVETE-  521

Query  515  TGVRGWTCRGIGGISQRKPWNPQPWSPNDEVETNASNISLLERRAGIELQQLHHINKVKW  574
              +RG+ CR I  ++QR+PWN +P      +E     ++ L RR    L +   ++  +
Sbjct  522  DRLRGYPCRSIPLVTQRRPWNARPVRHEAGLEHVLKTLATLRRR----LPRTDGVDAFR-  576

Query  575  SRHVRQSYKYLE-------LPKRLGGFGIYRFQG-WLPNG--KLPLAKKPLVNVEDIHPS  624
               VR+S++ L        +P  LGG G+  + G W+ +   ++   +  + N  D
Sbjct  577  EHFVRRSFRLLRLDPRLRTIPLSLGGLGVDPWDGQWMVHSWRQIDPPRIRITNKTDFREQ  636

Query  625  Q--ELF----LPLSEQQKKILAQVEMTNKMQTDDIPGTQKLFSKEWIQKVRAKKII  674
            +  E F    + ++ ++   LA+  +  K+  DD+P    +  +    +++A++ +
Sbjct  637  KVAEEFAGAGVDVTPEEVSALAERRIRAKVAADDVPAFAGIVRRIGRTELQARRFV  692


>MAG: putative RNA-dependent RNA polymerase [Totiviridae sp.]
Sequence ID: XZG29191.1 Length: 943
Range 1: 125 to 768

Score:187 bits(474), Expect:2e-50,
Method:Compositional matrix adjust.,
Identities:165/671(25%), Positives:314/671(46%), Gaps:73/671(10%)

Query  62   FYVPRSNADLVIRRIRLIDLWRNLKPDQMDE-----IRNYTHLDYIFVQNICIYMLVFGI  116
             +  R+  DL IR +R+ D+   +K   M +     +R     DY+   N  +  L F
Sbjct  125  LFPARTKEDLGIRNLRMGDMLLFMKRXGMTDKAHHVLRLCRGFDYVLTSNCLLAALCF--  182

Query  117  NTVKHFRQI----GLF-NERNEFIEIAKQLSTKGKRFVDDVD-NMKQKVCEIATIVGYMD  170
             T  ++  +    G+F ++   F    K+LS   K+  +++  + +  + E A++ G M
Sbjct  183  -TQDYWWSVMVASGVFLSDSEHFRAACKELSDTVKKTNNELTPDERVSLYECASLYGAMC  241

Query  171  PNVDKIDVMEEVNSLAAEGN-EHGIDRDNW------NDLFTKT--CKEVMTWYKGHEFIS  221
            P V   D +E+   LA  G   HG+    W      ND       C E    +   E  +
Sbjct  242  PPVPGWDPVEQTRDLAEGGQVPHGLLAGRWGTQGRLNDRIRDLVRCPE----HTSPEVRT  297

Query  222  FDDYIKEGMWLTSGSSSIGKVHW--TKDGEN--GKFKARKNMLLQIYTPQELANIVYAWD  277
            F+D++++  W  SG++++G+V +    +GE   G F+ARKN++L +   +E+       D
Sbjct  298  FEDWLRDCEWERSGAATLGRVEYELVVNGEERRGSFRARKNLVLDVVPIEEIVARTRNHD  357

Query  278  GKLHSRVFIKNEMSKLRLAVASNIEAYIHESYMLFLYGHGFKEYFGVTLDEKPDQQHQRE  337
             +  ++  IK+E  K+RLAV++ +E Y+ ++++  + G+ +  + G TL+E   ++  R
Sbjct  358  TQ-ENKALIKSEFGKIRLAVSAPLEVYLQQAFLFAVSGNSYLSWPGNTLEETLTEEMTRN  416

Query  338  IEMIEKLQAGYFGLPFDYASFDHQPTTFEV-----KTMVRRVGEIVVSQVPKNYYYQTQL  392
                 +++ G   LP+D+  FDHQP T E+      T  R +G   V Q      ++  L
Sbjct  417  ERTFVQMRRGANALPYDFQKFDHQPRTSEILCFQNVTFARALGAASVRQRDDVALFEALL  476

Query  393  LVNKIVNAYDKSYLSGNIKNTKFENIKVKGGVPSGVRITSLLGNMWNAIITKIA--INNV  450
                    + ++ ++           +V+GG+ SG+R TS +G+ WN+++ + +  + N
Sbjct  477  -----ERGFQQATITSPPGLGDQRTFRVRGGLMSGLRSTSAVGSGWNSVLGETSRDLANK  531

Query  451  IGIIGYDPISQISLRGDDVAILSKDPAALYLLRLSYAAINAIGKDSKLGISPKVCEFLRN  510
                G D  +   +RGDD  ++S     +  ++L Y  + A   +SK  + P   EFLR
Sbjct  532  FRGPGRDLDTWQIVRGDDTQVVSDSYLDVLAVKLGYDLLGAEANESKFTLRPGRTEFLRV  591

Query  511  EISVTGVRGWTCRGIGGISQRKPWNPQPWSPNDEVETNASNISLLERR---AGIELQQLH  567
            E S    R + CR +  + QR+PW+ +P + +  +       S+L RR   A + ++ +
Sbjct  592  ETSDRS-RAYPCRTVPLLFQRRPWSTRPPTGDASLTRVTKVASVLARRLTDASLLMEFVE  650

Query  568  HINKVKWSRHVRQSYKYLELPKRLGGFGIYRFQG------WLPNGKLPLAKKPLVNVEDI  621
             + +   S+ +    +  ++P  LGG G++ + G      W P+  +P+    + N
Sbjct  651  FVVERMLSK-MSVDLRLAKIPISLGGLGLFPWDGKWHVREWSPSPPIPIN---VTNSTGF  706

Query  622  HPSQ------ELFLPLSEQQKKILAQVEMTNKMQTDDIPGTQKLFSKEWIQKVRAKKIIW  675
               Q      E+ +P++  +   LA+  +  K+  DD+     L     +++VR  ++
Sbjct  707  RTGQEARRYAEMGVPITGGEAARLAERALRTKIAMDDV-----LELTGVVRRVRRAEL--  759

Query  676  SRNQTIPIHTD  686
             RN+TI + TD
Sbjct  760  -RNRTI-VRTD  768


>MAG: RNA-dependent RNA polymerase [Totiviridae sp.]
Sequence ID: WAK77535.1 Length: 831
Range 1: 13 to 675

Score:186 bits(471), Expect:3e-50,
Method:Compositional matrix adjust.,
Identities:165/680(24%), Positives:306/680(45%), Gaps:58/680(8%)

Query  62   FYVPRSNADLVIRRIRLIDLW-----RNLKPDQMDEIRNYTHLDYIFVQNICIYMLVFGI  116
             Y PR+  DL IR +R+ D+      + L    +  ++     DY+   N  +  L F
Sbjct  13   LYPPRTKEDLGIRNLRMGDMLLFMRRKGLADRAVHILKLCRGFDYVLTSNCLLAALCF--  70

Query  117  NTVKHFRQIGLFNERNEFIEIAKQLSTKGKRFVDDVDNMKQKVC--------EIATIVGY  168
               +  +   +      F+  A       K   D V     ++         E A++ G
Sbjct  71   ---RDQKWWDIMVASGVFLRDASHYRLACKELSDTVKKTNNELTADERVSLYECASLYGA  127

Query  169  MDPNVDKIDVMEEVNSLAAEGNE-HGIDRDNW--NDLFTKTCKEVMTW--YKGHEFISFD  223
            M P V   D +EE   LA  G + HG+   +W       K  +E++    +   E  SFD
Sbjct  128  MCPPVPGWDPIEETKQLAEGGQKPHGLLAGDWMQQAQLNKRIRELVRHPPHSRPEVRSFD  187

Query  224  DYIKEGMWLTSGSSSIGKVHW--TKDGEN--GKFKARKNMLLQIYTPQELANIVYAWDGK  279
            +++    W  SGS+++G+V +  + DGE   G FKARKN++L +    +L       D +
Sbjct  188  EWVASCEWERSGSATMGRVEYELSIDGERRKGSFKARKNLVLDVVPLNDLLAKTRVHDTQ  247

Query  280  LHSRVFIKNEMSKLRLAVASNIEAYIHESYMLFLYGHGFKEYFGVTLDEKPDQQHQREIE  339
              +   +K+E  K+RLAV++ +E Y+ ++++  + G+ +  + G TL+E   ++  R  +
Sbjct  248  -DNHALVKSEFGKVRLAVSAPLEVYLQQAFLFAVSGNSYLSWPGNTLEESVGEEMARNEK  306

Query  340  MIEKLQAGYFGLPFDYASFDHQPTTFEVKTMVRRVGEIVVSQVPKNYYYQTQ--LLVNKI  397
               +++AG   LP+D+  FDHQP T EV   V   G      +    ++Q Q  LL  ++
Sbjct  307  TFRRMRAGDSALPYDFMRFDHQPKTDEV---VEFQGVTFDRALAAALWWQRQDVLLFERL  363

Query  398  V-NAYDKSYLSGNIKNTKFENIKVKGGVPSGVRITSLLGNMWNAIITKIA--INNVIGII  454
            +   + ++ ++        +  +V GG+ SG+R TS +G+ WNA++ + A  + N
Sbjct  364  LERGFRRATITSPPGLGAPQTFRVLGGLMSGLRSTSAVGSGWNAVLGETARDLANKFRAP  423

Query  455  GYDPISQISLRGDDVAILSKDPAALYLLRLSYAAINAIGKDSKLGISPKVCEFLRNEISV  514
            G +  +   +RGDD  ++S     +  ++L Y  + A   +SK  + P   EFLR E+S
Sbjct  424  GRELATWQIVRGDDTQVISSSYLDVLAVKLGYDVLGAEANESKFTLRPGRTEFLRVEMS-  482

Query  515  TGVRGWTCRGIGGISQRKPWNPQPWSPNDEVETNASNISLLERRAGIELQQLHHINKV--  572
               R + CR +  + QR+PW+ +P + +  +       S+L RR       L  +  V
Sbjct  483  DRARAYPCRTVPLLFQRRPWSSRPPTGDASLTRIVKVASVLARRLTDPSLMLEFVEFVIE  542

Query  573  KWSRHVRQSYKYLELPKRLGGFGIYRFQG------WLPNGKLPLAKKPLVNVEDIHPSQ-  625
            +    +    +   +P  LGG G+  + G      W     +P+  +   N  D    Q
Sbjct  543  RMMAKMGLDRRLARIPVSLGGLGLLPWDGRWHVRQWASFPPIPIDVR---NATDFREKQE  599

Query  626  -----ELFLPLSEQQKKILAQVEMTNKMQTDDIPGTQKLFSKEWIQKVRAKKIIWSRNQT  680
                 E+ + +++ +   LA+     K+  DD+     +  +    ++R ++II + N
Sbjct  600  AKRYSEMGVRVTDGEAARLAERARRTKLALDDVVELTGVVRRVRRSELRQREIIRTEN-P  658

Query  681  IPIHTDHTVRIPKWDEKIKF  700
            +P+  D    + ++ E ++F
Sbjct  659  LPVSCDG---VGQYSELVQF  675


>RNA-dependent RNA polymerase [Totiviridae sp.]
Sequence ID: XCG98881.1 Length: 831
Range 1: 13 to 660

Score:184 bits(467), Expect:9e-50,
Method:Compositional matrix adjust.,
Identities:159/668(24%), Positives:311/668(46%), Gaps:66/668(9%)

Query  62   FYVPRSNADLVIRRIRLIDLWRNLKPDQMDE-----IRNYTHLDYIFVQNICIYMLVFGI  116
             Y  R+  DL IR +R+ D+   +K   M +     +R     DY+   N  +  L F
Sbjct  13   LYPARTKEDLGIRNLRMGDMLLFMKRKGMTDKACHVLRLCRGFDYVLTSNCLLAALCF--  70

Query  117  NTVKHFRQI----GLF-NERNEFIEIAKQLSTKGKRFVDDVD-NMKQKVCEIATIVGYMD  170
             T  ++  +    G+F ++   F    K+LS   K+  +++  + +  + E A++ G M
Sbjct  71   -TQDYWWSVMVASGVFLSDSEHFRAACKELSDTVKKTNNELTPDERVSLYECASLYGAMC  129

Query  171  PNVDKIDVMEEVNSLAAEGN-EHGIDRDNW------NDLFTKT--CKEVMTWYKGHEFIS  221
            P V   D +E+   LA  G   HG+    W      ND       C E    +   E  +
Sbjct  130  PPVPGWDPVEQTRDLAEGGQVPHGLLAGRWGTQSRLNDRIRDLVRCPE----HTSPEVRT  185

Query  222  FDDYIKEGMWLTSGSSSIGKVHW--TKDGE--NGKFKARKNMLLQIYTPQELANIVYAWD  277
            F+D++++  W  SG++++G+V +  + +GE  +G F+ARKN++L +    ++     + D
Sbjct  186  FEDWLRDCEWERSGAATLGRVEYELSVNGEERSGSFRARKNLVLDVVPIGDILARTRSHD  245

Query  278  GKLHSRVFIKNEMSKLRLAVASNIEAYIHESYMLFLYGHGFKEYFGVTLDEKPDQQHQRE  337
             +  ++  IK+E  K+RLAV++ +E Y+ ++++  + G+ +  + G TL+E   ++  R
Sbjct  246  TQ-ENKALIKSEFGKIRLAVSAPLEVYLQQAFLFAVSGNSYLSWPGNTLEETLTEEMTRN  304

Query  338  IEMIEKLQAGYFGLPFDYASFDHQPTTFEV-----KTMVRRVGEIVVSQVPKNYYYQTQL  392
                  ++ G   LP+D+  FDHQP T E+      T  R +G   V Q      +++ L
Sbjct  305  ERTFVHMRRGAHALPYDFQKFDHQPRTSEILCFQNVTFERALGAASVQQRDDVALFESLL  364

Query  393  LVNKIVNAYDKSYLSGNIKNTKFENIKVKGGVPSGVRITSLLGNMWNAIITKIA--INNV  450
                    + ++ ++           +V+GG+ SG+R TS +G+ WN+++ + +  + N
Sbjct  365  -----EKGFQQATITSPPGLGDQRTFRVRGGLMSGLRSTSAVGSGWNSVLGETSRDLANK  419

Query  451  IGIIGYDPISQISLRGDDVAILSKDPAALYLLRLSYAAINAIGKDSKLGISPKVCEFLRN  510
                G    +   +RGDD  ++S     +  ++L Y  + A   +SK  + P   EFLR
Sbjct  420  FRGPGQGLDTWQIVRGDDTQVVSDSYLDVLAIKLGYDLLGAEANESKFALRPGRTEFLRV  479

Query  511  EISVTGVRGWTCRGIGGISQRKPWNPQPWSPNDEVETNASNISLLERR---AGIELQQLH  567
            E S    R + CR +  + QR+PW+ +P + +  +       S+L RR   A + ++ +
Sbjct  480  ETSDRS-RAYPCRTVPLLFQRRPWSTRPPTGDASLTRVTKVASVLARRLVDASLLMEFVE  538

Query  568  HINKVKWSRHVRQSYKYLELPKRLGGFGIYRFQG------WLPNGKLPLAKKPLVNVEDI  621
             + +   S+ +    +  ++P  LGG G++ + G      W P+  +P+    + N
Sbjct  539  FVVERMLSK-MSVDLRLAKIPISLGGLGLFPWDGKWHVREWSPSPPIPIN---VTNSTGF  594

Query  622  HPSQ------ELFLPLSEQQKKILAQVEMTNKMQTDDIPGTQKLFSKEWIQKVRAKKIIW  675
               Q      E+ +P++  +   LA+  +  K+  DD+     +  +    ++R + I+
Sbjct  595  RTEQEARRYAEMGVPITGGEATRLAERALRTKIAMDDVLELTGVVRRVRRSELRNRAIV-  653

Query  676  SRNQTIPI  683
             R +  P+
Sbjct  654  -RTEIAPV  660


>MAG: RNA-dependent RNA polymerase [Totiviridae sp.]
Sequence ID: WAK77658.1 Length: 803
Range 1: 21 to 631

Score:182 bits(462), Expect:4e-49,
Method:Compositional matrix adjust.,
Identities:157/627(25%), Positives:293/627(46%), Gaps:53/627(8%)

Query  93   IRNYTHLDYIFVQNICIYMLVFGINTVKHFRQI----GLF-NERNEFIEIAKQLSTKGKR  147
            +R     DY+   N  +  L F   T  ++  I    G+F ++   F    K+LS   K+
Sbjct  21   LRLCRGFDYVTTSNCLLAALCF---TQDYWWSIMISSGVFLSDSEHFRAACKELSDTVKK  77

Query  148  FVDDVD-NMKQKVCEIATIVGYMDPNVDKIDVMEEVNSLAAEGN-EHGIDRDNW---NDL  202
              +D+  + +  + E A++ G M P V   D +E+   LA  G   HG+   +W     L
Sbjct  78   TNNDLTPDERVSLYECASLYGAMCPPVPGWDPIEQTRDLADGGQIPHGLLTGSWMTQGQL  137

Query  203  FTKTCKEV-MTWYKGHEFISFDDYIKEGMWLTSGSSSIGKVHW--TKDGEN--GKFKARK  257
              +  + V    +   E  SF+D++K+  W  SG++++G+V +  + +GE   G F+ARK
Sbjct  138  IDRIQELVKCPAHSSPEVRSFEDWLKDCEWERSGAATLGRVEYELSVNGEERRGSFRARK  197

Query  258  NMLLQIYTPQELANIVYAWDGKLHSRVFIKNEMSKLRLAVASNIEAYIHESYMLFLYGHG  317
            N++L +   +EL     A D +  ++  IK+E  K+RLAV++ +E Y+ ++++  + G+
Sbjct  198  NLVLDVVPVEELIARTRAHDTQ-ENKALIKSEFGKIRLAVSAPLEVYLQQAFLFAVSGNS  256

Query  318  FKEYFGVTLDEKPDQQHQREIEMIEKLQAGYFGLPFDYASFDHQPTTFEV-----KTMVR  372
            +  + G TL+E   ++  R  +   ++  G   LP+D+  FDHQP T E+      T  R
Sbjct  257  YLSWPGNTLEETITEEMTRNEKTFVRMAQGASALPYDFQKFDHQPRTSEIVGFQNVTFSR  316

Query  373  RVGEIVVSQVPKNYYYQTQLLVNKIVNAYDKSYLSGNIKNTKFENIKVKGGVPSGVRITS  432
             +    V Q          L  + +V  ++ + ++           +VKGG+ SG+R TS
Sbjct  317  ALAAANVWQ-----RDDVNLFEHLLVRGFEHATVTSPPGLGDQRTFRVKGGLMSGLRSTS  371

Query  433  LLGNMWNAIITKIA--INNVIGIIGYDPISQISLRGDDVAILSKDPAALYLLRLSYAAIN  490
             +G+ WN+++ + +  + N     G    +   +RGDD  ++S     +  ++L Y  +
Sbjct  372  AVGSGWNSVLGETSRDLANKFRGPGRGLNTWQIVRGDDTQVVSDSYLDVLAVKLGYDLLG  431

Query  491  AIGKDSKLGISPKVCEFLRNEISVTGVRGWTCRGIGGISQRKPWNPQPWSPNDEVETNAS  550
            A   +SK  + P   EFLR E S    R + CR +  + QR+PW+ +P + +  +
Sbjct  432  AEANESKFTLRPGRTEFLRVETS-DRARAYPCRTVPLLFQRRPWSTRPPTGDASLTRITK  490

Query  551  NISLLERRAGIELQQLHHINKVKWSRHVRQ---SYKYLELPKRLGGFGIYRFQG------  601
              S+L RR   +   L         R + +    ++  ++P  +GG G++ + G
Sbjct  491  VGSVLARRL-TDASLLEEFVVFVVERMLSKMGVDHRLAKIPVNMGGLGLFPWDGRWHVRQ  549

Query  602  WLPNGKLPLAKKPLVNVEDIHPSQ------ELFLPLSEQQKKILAQVEMTNKMQTDDIPG  655
            W P+  +P+    + N       Q      E+ +P++E +   LA+  +  K+  DD+
Sbjct  550  WSPSPPIPVN---VTNSTGFRTHQEAKRYAEMGVPVTEGEAARLAERSLRTKLALDDVLE  606

Query  656  TQKLFSKEWIQKVRAKKIIWSRNQTIP  682
               +  +    ++RA+ I+  R  T P
Sbjct  607  LAGVVRRVRRSELRARTIV--RTDTAP  631


>MAG: RNA-dependent RNA polymerase [Totiviridae sp.]
Sequence ID: WAK77245.1 Length: 2832
Range 1: 2014 to 2661

Score:182 bits(461), Expect:3e-48,
Method:Composition-based stats.,
Identities:162/668(24%), Positives:311/668(46%), Gaps:66/668(9%)

Query  62    FYVPRSNADLVIRRIRLIDLWRNLKPDQMDE-----IRNYTHLDYIFVQNICIYMLVFGI  116
              +  R+  DL IR +R+ D+   +K   M +     +R     DY+   N  +  L F
Sbjct  2014  LFPARTKEDLGIRNLRMGDMLLFMKRKGMTDKAHHVLRLCRGFDYVLTSNCLLAALCF--  2071

Query  117   NTVKHFRQI----GLFNERNEFIEIA-KQLSTKGKRFVDDVD-NMKQKVCEIATIVGYMD  170
              T  ++  +    G+F   +E    A K+LS   K+  +++  + +  + E A++ G M
Sbjct  2072  -TQDYWWSVMVASGVFLSDSEHFRAACKELSDTVKKTNNELTPDERVSLYECASLYGAMC  2130

Query  171   PNVDKIDVMEEVNSLAAEGN-EHGIDRDNW------NDLFTK--TCKEVMTWYKGHEFIS  221
             P V   D +E+   LA  G   HG+    W      ND       C E    +   E  +
Sbjct  2131  PPVPGWDPVEQTRDLAEGGQVPHGLLAGRWGTQGRLNDRIRDLVRCPE----HTSPEVRT  2186

Query  222   FDDYIKEGMWLTSGSSSIGKVHW--TKDGEN--GKFKARKNMLLQIYTPQELANIVYAWD  277
             F+D++++  W  SG++++G+V +    +GE   G F+ARKN++L +   +++     + D
Sbjct  2187  FEDWLRDCEWERSGAATLGRVEYELVVNGEERRGSFRARKNLVLDVVPVEDILARTRSHD  2246

Query  278   GKLHSRVFIKNEMSKLRLAVASNIEAYIHESYMLFLYGHGFKEYFGVTLDEKPDQQHQRE  337
              +  ++  IK+E  K+RLAV++ +E Y+ ++++  + G+ +  + G TL+E   ++  R
Sbjct  2247  TQ-ENKALIKSEFGKIRLAVSAPLEVYLQQAFLFAVSGNSYLSWPGNTLEETLTEEMTRN  2305

Query  338   IEMIEKLQAGYFGLPFDYASFDHQPTTFEV-----KTMVRRVGEIVVSQVPKNYYYQTQL  392
                  +++ G   LP+D+  FDHQP T E+      T  R +G   V Q      ++T L
Sbjct  2306  ERTFVQMRRGANALPYDFQKFDHQPRTSEILCFQNVTFARALGAASVQQRDDVALFETLL  2365

Query  393   LVNKIVNAYDKSYLSGNIKNTKFENIKVKGGVPSGVRITSLLGNMWNAII--TKIAINNV  450
                     + ++ ++           +V+GG+ SG+R TS +G+ WN+++  T   + N
Sbjct  2366  -----ERGFQQATITSPPGLGDQRTFRVRGGLMSGLRSTSAVGSGWNSVLGETSRDLANK  2420

Query  451   IGIIGYDPISQISLRGDDVAILSKDPAALYLLRLSYAAINAIGKDSKLGISPKVCEFLRN  510
                 G D  +   +RGDD  ++S     +  ++L Y  + A   +SK  + P   EFLR
Sbjct  2421  FRGPGRDLDTWQIVRGDDTQVVSDSYLDVLAVKLGYDLLGAEANESKFTLRPGRTEFLRV  2480

Query  511   EISVTGVRGWTCRGIGGISQRKPWNPQPWSPNDEVETNASNISLLERR---AGIELQQLH  567
             E S    R + CR +  + QR+PW+ +P + +  +       S+L RR   A + ++ +
Sbjct  2481  ETSDRS-RAYPCRTVPLLFQRRPWSTRPPTGDASLTRVTKVASVLARRLTDASLLMEFVE  2539

Query  568   HINKVKWSRHVRQSYKYLELPKRLGGFGIYRFQG------WLPNGKLPLAKKPLVNVEDI  621
              + +   S+ +    +  ++P  LGG G++ + G      W P+  +P+    + N
Sbjct  2540  FVVERMLSK-MSVDLRLAKIPISLGGLGLFPWDGKWHVREWSPSPPIPIT---VTNSTGF  2595

Query  622   HPSQ------ELFLPLSEQQKKILAQVEMTNKMQTDDIPGTQKLFSKEWIQKVRAKKIIW  675
                Q      E+ +P++  +   LA+  +  K+  DD+     +  +    ++R + I+
Sbjct  2596  RTGQEARRYAEMGVPITGGEATKLAERALRTKIAMDDVLELTGVVRRVRRSELRNRTIV-  2654

Query  676   SRNQTIPI  683
              R +  P+
Sbjct  2655  -RTEIAPV  2661


>MAG: RNA-dependent RNA polymerase, partial [Totiviridae sp.]
Sequence ID: WAK77504.1 Length: 535
Range 1: 27 to 498

Score:172 bits(437), Expect:3e-47,
Method:Compositional matrix adjust.,
Identities:130/483(27%), Positives:228/483(47%), Gaps:34/483(7%)

Query  99   LDYIFVQNICIYMLVFGINTVKHFRQIGLFNERNEFIEIAKQLSTKGKRFVDDVDNMKQK  158
             DY+   N  +  L F     +  +   +      F+  A       K   D V     +
Sbjct  27   FDYVLTSNCLLAALCF-----RDQKWWDIMVASGVFLRDASHYRLACKELSDTVKKTNNE  81

Query  159  VC--------EIATIVGYMDPNVDKIDVMEEVNSLAAEGNE-HGIDRDNW--NDLFTKTC  207
            +         E A++ G M P V   D +EE   LA  G + HG+   +W       K
Sbjct  82   LTADERVSLYECASLYGAMCPPVPGWDPIEETKQLAEGGQKPHGLLAGDWMQQAQLNKRI  141

Query  208  KEVMTWYKGH---EFISFDDYIKEGMWLTSGSSSIGKVHW--TKDGEN--GKFKARKNML  260
            +E++  +  H   E  SFD+++    W  SGS+++G+V +    DGE   G FKARKN++
Sbjct  142  RELVR-HPSHSRPEVRSFDEWVASCEWERSGSATMGRVEYELAIDGERRKGSFKARKNLV  200

Query  261  LQIYTPQELANIVYAWDGKLHSRVFIKNEMSKLRLAVASNIEAYIHESYMLFLYGHGFKE  320
            L +    +L       D +  +   +K+E  K+RLAV++ +E Y+ ++++  + G+ +
Sbjct  201  LDVVPLNDLLAKTRVHDTQ-DNHALVKSEFGKVRLAVSAPLEVYLQQAFLFAVSGNSYLS  259

Query  321  YFGVTLDEKPDQQHQREIEMIEKLQAGYFGLPFDYASFDHQPTTFEVKTMVRRVGEIVVS  380
            + G TL+E   ++  R  +   +++AG   LP+D+  FDHQP T EV   V   G
Sbjct  260  WPGNTLEESVGEEMARNEKTFRRMRAGDSALPYDFMRFDHQPKTDEV---VEFQGVTFDR  316

Query  381  QVPKNYYYQTQ--LLVNKIVN-AYDKSYLSGNIKNTKFENIKVKGGVPSGVRITSLLGNM  437
             +    ++Q Q  LL  +++   + ++ ++        +  +V GG+ SG+R TS +G+
Sbjct  317  ALASALWWQRQDVLLFERLLERGFRRATITSPPGLGAPQTFRVLGGLMSGLRSTSAVGSG  376

Query  438  WNAIITKIA--INNVIGIIGYDPISQISLRGDDVAILSKDPAALYLLRLSYAAINAIGKD  495
            WNA++ + A  + N     G +  +   +RGDD  ++S     +  ++L Y  + A   +
Sbjct  377  WNAVLGETARDLANKFRAPGRELATWQIVRGDDTQVISSSYLDVLAVKLGYDVLGAEANE  436

Query  496  SKLGISPKVCEFLRNEISVTGVRGWTCRGIGGISQRKPWNPQPWSPNDEVETNASNISLL  555
            SK  + P   EFLR EIS    R + CR +  + QR+PW+ +P + +  +       S+L
Sbjct  437  SKFTLRPGRTEFLRVEIS-DRARAYPCRTVPLLFQRRPWSSRPPTGDASLTRIVKVASVL  495

Query  556  ERR  558
             RR
Sbjct  496  ARR  498


>MAG: RNA-dependent RNA polymerase, partial [Totiviridae sp.]
Sequence ID: WAK77481.1 Length: 629
Range 1: 185 to 623

Score:172 bits(436), Expect:1e-46,
Method:Compositional matrix adjust.,
Identities:129/445(29%), Positives:219/445(49%), Gaps:32/445(7%)

Query  56   DKELQTFYVPRSNADLVIRRIRLIDLW----RNLKPDQMDEI-RNYTHLDYIFVQNICIY  110
            D+ +   +  RS+ DL IR +R  DLW     N   +++ ++ R     DYI + N+ +
Sbjct  185  DRLILAAFPARSSQDLGIRYVRAPDLWVFFMENRSYEELRQVLRQLKFFDYICMVNLLLA  244

Query  111  MLVF-GINTVKHFRQIGLFNERNEFIEIA-KQLSTKGKRFVDDVDNMK----QKVC--EI  162
             +VF          + G F+  +E   +A KQLS   K+     +N++    ++VC  E
Sbjct  245  KVVFTNPQWWDTLARAGCFDGGSEHFRLACKQLSDWIKK-----ENVQLPAAERVCYYEC  299

Query  163  ATIVGYMDPNVDKIDVMEEVNSLAAEG-NEHGI---DRDNWN-DLFTKTCKEVMTW--YK  215
            A++ G M P V   D + E   LA  G +EHG+    RD +  + F    +++
Sbjct  300  ASLYGAMCPPVPGWDPILETRELAQGGQDEHGLLLSSRDGYAPEDFCAAIEDLAALPPRD  359

Query  216  GHEFISFDDYIKEGMWLTSGSSSIGKVHWTKDGE----NGKFKARKNMLLQIYTPQELAN  271
              +  +F ++++   W  +G+SSIG V +T + E     G+FKARKN LL +    +L
Sbjct  360  SPDVRTFREFLESEEWARAGASSIGTVEFTLEAEGTRKTGRFKARKNFLLDVVELDDLER  419

Query  272  IVYAWDGKLHSRVFIKNEMSKLRLAVASNIEAYIHESYMLFLYGHGFKEYFGVTLDEKPD  331
             V     +  +   IK E+SK+RLAV+S +E Y+ ++++  L G+ +  +   TL+E
Sbjct  420  RVRECREQ-RNVALIKTELSKIRLAVSSPVEVYLPQTWLFLLSGNAYSSWPSNTLEEPLW  478

Query  332  QQHQREIEMIEKLQAGYFGLPFDYASFDHQPTTFEVKTMVRRVGEIVVSQVPKNYYYQTQ  391
            ++  R     E+LQAG + LP+D+A FDHQPTT E++   R   E     V ++     +
Sbjct  479  KEQARHETTWERLQAGEYSLPYDFARFDHQPTTAEIQAFQRATNECARQMVQEHQRRDFE  538

Query  392  LLVNKIVNAYDKSYLSGNIKNTKFENIKVKGGVPSGVRITSLLGNMWNAIITKIAINNVI  451
             L   ++  +  + L+           KV GG+ SG+R TS +G+ WNAI  + A   +
Sbjct  539  ELTANVLQGFSNATLTSPPGLGDQLTFKVTGGLMSGLRSTSCVGSGWNAIFGQFATRLIY  598

Query  452  GIIGYDPISQI--SLRGDDVAILSK  474
             + G      +   +RGDD  ++ +
Sbjct  599  DVRGGQAAPHVWQLVRGDDTQVVGR  623


>MAG: RNA-dependent RNA polymerase [Totiviridae sp.]
Sequence ID: WAK77695.1 Length: 704
Range 1: 1 to 444

Score:173 bits(439), Expect:1e-46,
Method:Compositional matrix adjust.,
Identities:130/452(29%), Positives:226/452(50%), Gaps:27/452(5%)

Query  169  MDPNVDKIDVMEEVNSLAAEG-NEHGIDRDNWNDLF-----TKTCKEVMTW--YKGHEFI  220
            M P V   D ++E   LA+ G +EHG+    W   +      +   ++  +   K H+
Sbjct  1    MCPPVPGWDPVQETRDLASGGQDEHGL---LWGPKYRPSSVLRAIADLAQYPAAKPHDTR  57

Query  221  SFDDYIKEGMWLTSGSSSIGKVHW-TKDGEN---GKFKARKNMLLQIYTPQELANIVYAW  276
            S + ++    W  SGSSS+G++ +   DG++   G+FKARKN++L I  P EL + +
Sbjct  58   SLEAWLLACDWERSGSSSMGRIEYEVFDGDSWRRGRFKARKNLVLDIIEPSELLDAIRTH  117

Query  277  DGKLHSRVFIKNEMSKLRLAVASNIEAYIHESYMLFLYGHGFKEYFGVTLDEKPDQQHQR  336
            D +  +R  IK+E  K+RLAV++ +  Y+ ++Y+  + G  +  + G TL+E  + +  R
Sbjct  118  DTQ-ENRALIKSEFGKIRLAVSAPLPTYLQQAYLYSVAGCPYLNWPGNTLEESLEHEMMR  176

Query  337  EIEMIEKLQAGYFGLPFDYASFDHQPTTFEVKTMVRRVGEIVVSQVPKNYYYQTQLLVNK  396
                +  +++G+F LP+D+A FDHQPTT EV    +      +           + + +
Sbjct  177  NERTLCLMRSGFFALPYDFARFDHQPTTAEVVEFQKLTFTRALLNANPWQIEDIKRIEHL  236

Query  397  IVNAYDKSYLSGNIKNTKFENIKVKGGVPSGVRITSLLGNMWNAIITKIA---INNVIGI  453
            +  A+D + L+      +    KV GG+ SG+R TS +G+ WN+++ +++    NN+ G
Sbjct  237  LEIAFDNATLTTPPGVCEPMTFKVTGGLMSGLRSTSAVGSGWNSVLGEMSRSIANNLRGP  296

Query  454  IGYDPISQISLRGDDVAILSKDPAALYLLRLSYAAINAIGKDSKLGISPKVCEFLRNEIS  513
                   QI +RGDD  ++S+    +  +++ Y A+ A   +SK  +     EFLR E
Sbjct  297  GAKLDTWQI-VRGDDTQVISRSYFDVLAVKIGYDALGAEANESKFTLRRGRTEFLRVE--  353

Query  514  VTG--VRGWTCRGIGGISQRKPWNPQPWSPNDEVETNASNISLLERRAGIELQQLHHINK  571
             TG   R + CR +  ++QRKPW+ +P S +         I++L RR       L
Sbjct  354  -TGNVARCYPCRTVPLVNQRKPWSGRPDSDDASAARVVKVINVLSRRVDDPRTILDFGRY  412

Query  572  VKWSRHVRQS--YKYLELPKRLGGFGIYRFQG  601
            V      ++    + L +P  LGG G+Y + G
Sbjct  413  VAEKMVAKKGVDVRLLGIPTSLGGLGLYPWDG  444


>MAG: putative RNA dependent RNA polymerase, partial [Totiviridae sp.]
Sequence ID: WWV90225.1 Length: 380
Range 1: 1 to 324

Score:156 bits(395), Expect:5e-43,
Method:Compositional matrix adjust.,
Identities:106/333(32%), Positives:166/333(49%), Gaps:26/333(7%)

Query  466  GDDVAILSKDPAALYLLRLSYAAINAIGKDSKLGISPKVCEFLRNEISVTGVRGWTCRGI  525
            GDD  I +      Y++RL YA++ A+G D+K  I  +  EFLR EI+     GW  R I
Sbjct  1    GDDTYICTNTAYEAYMIRLCYASVWAVGNDAKFSIRQQSLEFLRTEITANQAYGWPNRSI  60

Query  526  GGISQRKPWNPQPWSPNDEVETNASNISLLERRAGIE--LQQLHHINKVKWSRHVRQSYK  583
              I+QRKPWN +P  P  EV T A NI+  ERR  I+     LH  N  +WSR  +QS
Sbjct  61   PSITQRKPWNDRPVDPALEVITIAENINTYERRNNIQPNTHWLHLANMRQWSRFTKQSID  120

Query  584  YLELPKRLGGFGIYRFQGWLPNGKLP--LAKKPLVNVEDIHPSQELFLPLSEQ------Q  635
            YL LP+RLGG G+Y+++G   + + P  +A KP ++       Q L +PL++
Sbjct  121  YLRLPRRLGGLGVYQYEGKRASKRFPPLIASKPNLD-------QTLHIPLTKHYLLPFVD  173

Query  636  KKILAQVEMTNKMQTDDIPGTQKLFSKEWIQKVRAKKIIWSRNQTIPIHTDHTVRIPKWD  695
            K  +AQ  + +K+   DIP        E +++VR  K  W R++   I   + + + + D
Sbjct  174  KDTIAQQTLLSKVVPSDIPSLDTATIDERVKQVRQHKTAWYRDKRFGIA--NPLLLNEID  231

Query  696  EKI--KFPRYKSEYILNNKINLTMEQVLRQY-NLLKEVERHDKDLKVPKLL----DILDK  748
            + I    P        NN   +        + N  K +  +   LK  K+      +
Sbjct  232  DMINKNLPTTPDYTFTNNLPVIDFASNDPNFPNFDKFISDYSPLLKSSKVTFSIGQMTKA  291

Query  749  WFPVQSSKIKTYESQGFHRTDAINLAVGEIPTE  781
            ++P    ++K  E+QGFHRTDA+++ + + P++
Sbjct  292  FYPKVYDELKRLENQGFHRTDALSILLRDCPSD  324


>MAG: RNA-dependent RNA polymerase, partial [Totiviridae sp.]
Sequence ID: WAK77298.1 Length: 617
Range 1: 188 to 617

Score:154 bits(389), Expect:1e-40,
Method:Compositional matrix adjust.,
Identities:125/434(29%), Positives:210/434(48%), Gaps:25/434(5%)

Query  56   DKELQTFYVPRSNADLVIRRIRLID-LWRNLKPDQMDEIRNYTHL----DYIFVQNICIY  110
            D   +  Y PR+  DL IR  R+ D L    +  + D +R    L    DYI V N+ +
Sbjct  188  DMLARELYPPRTREDLAIRVCRIGDVLDYAARTGRTDVVRRVLQLCRGFDYITVNNLLVA  247

Query  111  MLVFGINTVKHFRQIGLF-NERNEFIEIAKQLST---KGKRFVDDVDNMKQKVCEIATIV  166
             L FG          GLF  +   F   A+ LS    K    +DD D +    C  A +
Sbjct  248  RLCFGPVWWNLTEGSGLFEGDSCHFRLAARALSDTVKKSDTCLDDYDRVSLYEC--ACLY  305

Query  167  GYMDPNVDKIDVMEEVNSLAAEGNE-HGI---DRDNWNDLFTKTCKEVMTWYKG--HEFI  220
            G M P V   D ++E   LA+ G E HG+   +R     +  +  +++  + +G  H+
Sbjct  306  GAMCPPVPGWDPVQETYELASGGQEEHGLLAGERYRPKSVL-QAIRDLGRYPEGRPHDTR  364

Query  221  SFDDYIKEGMWLTSGSSSIGKVHW-TKDGEN---GKFKARKNMLLQIYTPQELANIVYAW  276
              + ++    W  SGSSS+G++ +   DGE    G FKARKN++L +  P  L   + +
Sbjct  365  PLEAWLLACEWERSGSSSMGRIDYEVLDGEEWRRGHFKARKNLVLDVLEPATLLEAIRSH  424

Query  277  DGKLHSRVFIKNEMSKLRLAVASNIEAYIHESYMLFLYGHGFKEYFGVTLDEKPDQQHQR  336
            D +  +R  IK+E  K+RLAV++ +  Y+ ++Y+  + G  +  + G TL+E  + +  R
Sbjct  425  DSQ-DNRALIKSEFGKIRLAVSAPLPTYLQQAYVYSVAGCPYLNWPGNTLEETLEDEMLR  483

Query  337  EIEMIEKLQAGYFGLPFDYASFDHQPTTFEVKTMVRRVGEIVVSQVPKNYYYQTQLLVNK  396
                I  ++AG+F LP+D+A FDHQPTT +V    +      +           +L+ +
Sbjct  484  NERTICLMRAGFFALPYDFARFDHQPTTAQVVEFQKLAFTRALLNANPWQEKDIRLIEHL  543

Query  397  IVNAYDKSYLSGNIKNTKFENIKVKGGVPSGVRITSLLGNMWNAIITKIAINNVIGIIGY  456
            +  ++  + L+      +    KV GG+ SG+R TS +G+ WN+++ +++ +    + G
Sbjct  544  LEISFSNATLTTPPGVCEPRTFKVTGGLMSGLRSTSAVGSGWNSVLGEMSRSIASDLRGL  603

Query  457  DPISQI--SLRGDD  468
            D   Q    +RGDD
Sbjct  604  DRDIQTWQIVRGDD  617


>MAG: RNA-dependent RNA polymerase, partial [Totiviridae sp.]
Sequence ID: WAK77652.1 Length: 526
Range 1: 106 to 514

Score:152 bits(385), Expect:1e-40,
Method:Compositional matrix adjust.,
Identities:118/414(29%), Positives:196/414(47%), Gaps:28/414(6%)

Query  56   DKELQTFYVPRSNADLVIRRIRLIDLWR-----NLKPDQMDEIRNYTHLDYIFVQNICIY  110
            D+     +  R+N DL IR  R  D+W          +  D +R   + DYI + N+ I
Sbjct  106  DRYALAAFPARTNQDLGIRYARAADIWSFYIETGRSQELRDTLRLLKYFDYITMVNVLIA  165

Query  111  MLVFG-INTVKHFRQIGLFNERNEFIEIA-KQLS--TKGKRFVDDVDNMKQKVCEIATIV  166
             +VF  +       + G F    E   +A + LS   K +    D D+ +    E A++
Sbjct  166  KIVFTRVVWWDTLVRAGCFVAGAEHFRLAARALSDYVKKENVALDPDD-RVSFYECASLY  224

Query  167  GYMDPNVDKIDVMEEVNSLAAEG-NEHGIDRDNWNDLFTKTCKEVMTWYKGHEFI-----  220
            G M P V   D +EE  +LA  G +EHG+  D        + ++ +   +   F+
Sbjct  225  GAMCPPVPGWDPVEETQALAEGGSDEHGLLLDCSPGY---SSEDFLDAVRALAFVKPRDD  281

Query  221  ----SFDDYIKEGMWLTSGSSSIGKVHWT----KDGENGKFKARKNMLLQIYTPQELANI  272
                 F +++ +  W  SG+SSIG+V +T    ++  +GKFKARKN++L +   +EL
Sbjct  282  PDPRPFHEWLWQEEWARSGASSIGQVRYTLTLGEERMSGKFKARKNLVLDVVPLEELEER  341

Query  273  VYAWDGKLHSRVFIKNEMSKLRLAVASNIEAYIHESYMLFLYGHGFKEYFGVTLDEKPDQ  332
               +  +  +   +K E++K+RLAV+  IE Y+ ++++  + G+ +  +   TL+E
Sbjct  342  ARTYT-RQDNVALVKTELAKIRLAVSCPIETYLAQAWIYLVTGNSYSNWPANTLEEPLHV  400

Query  333  QHQREIEMIEKLQAGYFGLPFDYASFDHQPTTFEVKTMVRRVGEIVVSQVPKNYYYQTQL  392
            +  R  E  EKLQ G + LP+D+A FDHQPTT EV           +
Sbjct  401  EVARHEETWEKLQDGQYSLPYDFARFDHQPTTAEVTAFQHATNLCGLRYARPEQMDDYLQ  460

Query  393  LVNKIVNAYDKSYLSGNIKNTKFENIKVKGGVPSGVRITSLLGNMWNAIITKIA  446
            L + ++  Y  + L+      K +  +V GG+ SG+R TS +G+ WNAI  + A
Sbjct  461  LSSCVIEGYSNATLTSPPGLGKQQTFRVSGGLMSGLRSTSCVGSGWNAIFGEFA  514


>MAG: RNA-dependent RNA polymerase, partial [Totiviridae sp.]
Sequence ID: WAK77755.1 Length: 365
Range 1: 7 to 333

Score:123 bits(309), Expect:8e-32,
Method:Compositional matrix adjust.,
Identities:95/330(29%), Positives:165/330(50%), Gaps:21/330(6%)

Query  56   DKELQTFYVPRSNADLVIRRIRLIDLWRNLK-PDQMDEIRNYTHL----DYIFVQNICIY  110
            D   +  Y PR+  DL IR  R+ D+    +   +  E+++   L    DYI   N+ +
Sbjct  7    DMLARQLYPPRTREDLAIRVCRIGDVLDYAQNTGRSSEVKSVLELCRGFDYITANNLLVA  66

Query  111  MLVFGINTVKHFRQIGLFN-ERNEFIEIAKQLSTKGKR---FVDDVDNMKQKVCEIATIV  166
             L FG    +  +  GLF+ + + F   A+ LS   K+    + D D +    C  A +
Sbjct  67   RLCFGSQWWELTQGSGLFDGDADHFRLSARALSDTVKKNDTCLTDYDRVSLYEC--ACLY  124

Query  167  GYMDPNVDKIDVMEEVNSLAAEGN-EHGI--DRDNWNDLFTKTCKEVMTW--YKGHEFIS  221
            G M P V   D ++E + LA+ G  EHG+        +   +  +++  +   + H+
Sbjct  125  GAMCPPVPGWDPVQETHDLASGGQQEHGLLFGSKYRPESVLQAIRDLGRYPEARPHDTRP  184

Query  222  FDDYIKEGMWLTSGSSSIGKVHW-TKDG---ENGKFKARKNMLLQIYTPQELANIVYAWD  277
             + ++    W  SGSSS+G++ +   DG     G FKARKN++L +  P  L   +   D
Sbjct  185  LEAWLLACEWERSGSSSMGRIEYEVLDGTEWRRGHFKARKNLVLDVIEPHVLLEAIRNHD  244

Query  278  GKLHSRVFIKNEMSKLRLAVASNIEAYIHESYMLFLYGHGFKEYFGVTLDEKPDQQHQRE  337
             +  ++  IK+E  K+RLAV++ +  Y+ ++Y+  + G  +  + G TL+E  + +  R
Sbjct  245  SQ-DNKALIKSEFGKIRLAVSAPLPTYLQQAYVYSVAGCPYLNWPGNTLEETLEDEMLRN  303

Query  338  IEMIEKLQAGYFGLPFDYASFDHQPTTFEV  367
               +  ++AG+F LP+D+A FDHQPTT +V
Sbjct  304  ERTLCLMRAGFFALPYDFARFDHQPTTPQV  333


>MAG: putative RNA dependent RNA polymerase, partial [Totiviridae sp.]
Sequence ID: WWV90304.1 Length: 167
Range 1: 6 to 167

Score:103 bits(258), Expect:5e-27,
Method:Compositional matrix adjust.,
Identities:59/169(35%), Positives:100/169(59%), Gaps:9/169(5%)

Query  642  VEMTNKMQTDDIPGTQKLFSKEWIQKVRAKKIIWSRNQTIPIHTDHTVRIPKWDEKIKFP  701
            V M+ K+  DDIPG QK  S E+++K+R  +  W++ Q IP+   + +  P +D  I +P
Sbjct  6    VSMSMKIAADDIPGPQKFMSIEYVKKLRTMRFNWNK-QIIPVTKMYKIEGPTFDN-ILWP  63

Query  702  RYKSEYILN-NKINL-TMEQVLRQYNLLKEVERHDKDLKVPKLLDILDKWFPVQSSKIKT  759
            + K    L  NKI +    + +R++ + K  +     +K+P L+++  +++P     I+
Sbjct  64   KTKLHVDLKPNKIEMPNFIEFVREHQIAKRAK-----IKIPSLMELTLRYYPSVYKDIRY  118

Query  760  YESQGFHRTDAINLAVGEIPTEPAVKINPILINFVKLHLERQGITHQRG  808
            YE++G+HRTDAINLA G +PTEP   +NP+L  F+   L + G+ H +G
Sbjct  119  YEARGWHRTDAINLASGSVPTEPTKILNPLLTVFITTMLLKSGLKHWKG  167


>MAG: RNA-dependent polymerase [Totiviridae sp.]
Sequence ID: WAK77683.1 Length: 235
Range 1: 61 to 209

Score:102 bits(253), Expect:1e-25,
Method:Composition-based stats.,
Identities:53/150(35%), Positives:88/150(58%), Gaps:5/150(3%)

Query  222  FDDYIKEGMWLTSGSSSIGKVHWT----KDGENGKFKARKNMLLQIYTPQELANIVYAWD  277
            F  ++ +  W+ SG+SS+G+VH+T     +   GKF ARKN++L +    +L      +
Sbjct  61   FRQWLWDEEWVRSGASSLGRVHYTLRIGDETRTGKFTARKNLVLDVIPINDLEQRTRTYT  120

Query  278  GKLHSRVFIKNEMSKLRLAVASNIEAYIHESYMLFLYGHGFKEYFGVTLDEKPDQQHQRE  337
             K  +   +K E++K+ LAV+  IE Y+ ++++  + G+ +  +   TL+E    +  R
Sbjct  121  -KQENVTLVKTELAKIHLAVSCPIETYLAQAWIYLVTGNAYSNWPANTLEEPLHVEVARH  179

Query  338  IEMIEKLQAGYFGLPFDYASFDHQPTTFEV  367
             E  E+LQAG + LP+D+A FDHQPTT EV
Sbjct  180  EETWERLQAGQYSLPYDFARFDHQPTTSEV  209


>MAG: nucleocapsid protein, partial [Totiviridae sp.]
Sequence ID: QYW06889.1 Length: 263
Range 1: 1 to 259

Score:92.8 bits(229), Expect:3e-22,
Method:Composition-based stats.,
Identities:76/268(28%), Positives:135/268(50%), Gaps:19/268(7%)

Query  301  IEAYIHESYMLFLYGHGFKEYFGVTLDEKPDQQHQREIEMIEKLQAGYFGLPFDYASFDH  360
            +  Y+ ++Y+  + G  +  + G TL+E  +++  R    I  ++ G+F LP+D++ FDH
Sbjct  1    LPVYLQQAYLYSVAGCPYLNWPGNTLEESLEEEMSRNERTIVLMRRGFFALPYDFSRFDH  60

Query  361  QPTTFEVKTMVRRVGEIVVSQVPKNYY-YQTQ------LLVNKIVNAYDKSYLSGNIKNT  413
            QPTT E+    R    I  ++   N   +Q+Q       L+    NA   +   G  + T
Sbjct  61   QPTTPEILEFQR----ITFTRALLNANPWQSQDIKLIEHLLELSFNAATLTTPPGICEPT  116

Query  414  KFENIKVKGGVPSGVRITSLLGNMWNAIITKIAIN---NVIGIIGYDPISQISLRGDDVA  470
             F   +V GG+ SG+R TS +G+ WN+++ +++ +      G  G     QI +RGDD
Sbjct  117  TF---RVTGGLMSGLRSTSAVGSGWNSVLGEMSRDIAAQFRGPGGRLLTWQI-VRGDDTQ  172

Query  471  ILSKDPAALYLLRLSYAAINAIGKDSKLGISPKVCEFLRNEISVTGVRGWTCRGIGGISQ  530
            ++S+    +  +++ Y A+ A   +SK  +     EFLR E      R + CR +  I+Q
Sbjct  173  VVSEKYLDVLAVKIGYDALGAEANESKFTLRQGRTEFLRVETGAVS-RCYPCRTVPLINQ  231

Query  531  RKPWNPQPWSPNDEVETNASNISLLERR  558
            +KPW+ +P S +         +++L RR
Sbjct  232  QKPWSGRPDSDDASAARVMKVVNVLSRR  259


>MAG: RNA-dependent RNA polymerase [Totiviridae sp.]
Sequence ID: WAK77592.1 Length: 159
Range 1: 2 to 115

Score:59.3 bits(142), Expect:1e-11,
Method:Composition-based stats.,
Identities:39/115(34%), Positives:60/115(52%), Gaps:3/115(2%)

Query  426  SGVRITSLLGNMWNAIITKIAINNVIGIIGYD-PISQISLR-GDDVAILSKDPAALYLLR  483
            SG+R TS +G+ WNAI  + A   +  +   D P S   L  G D  +       +  ++
Sbjct  2    SGLRSTSCVGSGWNAIFGEFARKMIDEMRSPDNPPSVWQLVCGYDTQVTGPSYHDVLAIK  61

Query  484  LSYAAINAIGKDSKLGISPKVCEFLRNEISVTGVRGWTCRGIGGISQRKPWNPQP  538
            +SY A+ A+  +SK  +     EFLR E     +RG+ CR I  ++QR+PWN +P
Sbjct  62   ISYDAMVAVANESKFTLRQGRTEFLRIETE-DQLRGYPCRTIPLLTQRRPWNARP  115


>RNA-dependent RNA polymerase [Totiviridae sp.]
Sequence ID: BDC79619.1 Length: 784
Range 1: 224 to 575

Score:46.6 bits(109), Expect:2e-06,
Method:Compositional matrix adjust.,
Identities:73/395(18%), Positives:155/395(39%), Gaps:63/395(15%)

Query  231  WLTSGSSSIGKVHWTKDGENGKFKARKNMLLQIYTPQELANIVYAWDGKLHSRVFIKNEM  290
            WL +G+S+ G +     G+   + A +  +  +    +  +     +    +RV  K E
Sbjct  224  WLVNGASTAGGMDGVLSGKYATYLAHRTRIAGVAAGSDSLD-----EYPGRARVIAKRER  278

Query  291  SKLRLAVASNIEAYIHESYMLFLYGHGFKEYFGVTLDEKPDQQHQREIEMIEKLQAGY--  348
             K+R  + +  + Y+ ++++    G   +E     +   P +  +  +   + L A
Sbjct  279  EKVRAVINAGFDLYVQQAFVFQGMGKLVRE-----VCRTPPEGGEPTLRFWDGLVADAKR  333

Query  349  -FGLPFDYASFDHQPTTFEVKTMVRRVGEIVVSQVPKNYYYQTQLLVNKIVNAYD--KSY  405
             + LP DY  FDH P+   +  ++  + +++  +              +   A++  ++
Sbjct  334  AYCLPLDYPKFDHIPSWGWISRVLDDMAQLLRGE---------GAAARERCRAWERARAT  384

Query  406  LSGNIKNTKFENIKVKGGVPSGVRITSLLGNMWNAII-TKIAINNVIGIIGYDPISQISL  464
            ++G       E +   GGV SG  +TS L  + NA +   + I   +     +  +  ++
Sbjct  385  MAGASLLVAGEEVPYHGGVLSGWYVTSDLDTLINAALEAGMRITQRL-----ETTAPSAV  439

Query  465  RGDDVAILSKDPAAL--------YLLRLSYAAINAIGKDSKLGISPKVCEFLRNEI--SV  514
            +GDD  + + + A           + RL  A     G+           EFLR  I   +
Sbjct  440  KGDDALVFAPNRAEARRWLSTFRRVFRLDLARFPTDGQRG---------EFLRYVIGGGL  490

Query  515  TGVRGWTCRGIGGISQRKPWNPQPWSPNDEVETNASNISLLERRAGIELQQLHHINKVKW  574
            TG RG+  R    +   + W        ++  + A   ++L  R G++ + +  +   +
Sbjct  491  TGRRGYPTRAAAALLWGQAWQG---GGLEDAGSIAGQAAVLVSR-GMDRESVERMLVARL  546

Query  575  SRHV----RQSYKYLELPKRLGGFGIYRFQGWLPN  605
            + H      ++ ++L  P  +GG       GWLP
Sbjct  547  ASHFGVVESEAARWLHTPTAVGG------AGWLPT  575


>RNA-dependent RNA polymerase, partial [Totiviridae sp.]
Sequence ID: BDG62461.1 Length: 300
Range 1: 1 to 299

Score:38.9 bits(89), Expect:4e-04,
Method:Compositional matrix adjust.,
Identities:63/337(19%), Positives:132/337(39%), Gaps:58/337(17%)

Query  289  EMSKLRLAVASNIEAYIHESYMLFLYGHGFKEYFGVTLDEKPDQQHQREIEMIEKLQAGY  348
            E  K+R  + +  + Y+ ++++    G   +E     +   P +  +  +   + L A
Sbjct  1    EREKVRAVINAGFDLYVQQAFVFQGMGKLVRE-----VCRTPPEGGEPTLRFWDGLVADA  55

Query  349  ---FGLPFDYASFDHQPTTFEVKTMVRRVGEIVVSQVPKNYYYQTQLLVNKIVNAYD--K  403
               + LP DY  FDH P+   +  ++  + +++  +              +   A++  +
Sbjct  56   KRAYCLPLDYPKFDHIPSWGWISRVLDDMAQLLRGE---------GAAARERCRAWERAR  106

Query  404  SYLSGNIKNTKFENIKVKGGVPSGVRITSLLGNMWNAII-TKIAINNVIGIIGYDPISQI  462
            + ++G       E +   GGV SG  +TS L  + NA +   + I   +     +  +
Sbjct  107  ATMAGASLLVAGEEVPYHGGVLSGWYVTSDLDTLINAALEAGMRITQRL-----ETTAPS  161

Query  463  SLRGDDVAILSKDPAAL--------YLLRLSYAAINAIGKDSKLGISPKVCEFLRNEI--  512
            +++GDD  + + + A           + RL  A     G+           EFLR  I
Sbjct  162  AVKGDDALVFAPNRAEARRWLSTFRRVFRLDLARFPTDGQRG---------EFLRYVIGG  212

Query  513  SVTGVRGWTCRGIGGISQRKPWNPQPWSPNDEVETNASNISLLERRAGIELQQLHHINKV  572
             +TG RG+  R    +   + W        ++  + A   ++L  R G++ + +  +
Sbjct  213  GLTGRRGYPTRAAAALLWGQAWQG---GGLEDAGSIAGQAAVLVSR-GMDRESVERMLVA  268

Query  573  KWSRHV----RQSYKYLELPKRLGGFGIYRFQGWLPN  605
            + + H      ++ ++L  P  +GG       GWLP
Sbjct  269  RLASHFGVVESEAARWLHTPTAVGG------AGWLPT  299


>MAG: RNA-dependent RNA polymerase [Totiviridae sp.]
Sequence ID: QKN88741.1 Length: 1316
Range 1: 824 to 1012

Score:35.4 bits(80), Expect:0.006,
Method:Compositional matrix adjust.,
Identities:50/191(26%), Positives:78/191(40%), Gaps:16/191(8%)

Query  420   VKGGVPSGVRITSLLGNMWNAIITKIA---INNVIGIIGYDPISQISLRGDDVAILSKDP  476
             VK G+ SG R T L+  + N    +IA   + + +G++  D +  +  +GDDV I S  P
Sbjct  824   VKQGMFSGTRSTDLINTLLNLAYFRIAKDYLESTMGVVSKD-LYHVH-QGDDVWISSASP  881

Query  477   AALYLLRLSYAAINAIGKDSKLGISPKVCEFLRNEISVTGVRGWTCRGIGGISQRKPWNP  536
                  +  S  ++  I + SK        E+LR   S  G  G+  R +     R   +
Sbjct  882   VWARGILYSLNSMGFIFQRSKQMFGQGRGEYLRVLYSGGGGYGYYGRALSNYLLRPLQSA  941

Query  537   QPWSPNDEVETNASNISLLERRA-GIELQQLHHINKVK-WSRH---------VRQSYKYL  585
                 P     T      LL RR    ++  + + N +  W+R          V    +YL
Sbjct  942   TTMDPVSWARTVRDGCCLLSRRGLSTQMANVVYNNGISFWARARAHPSDTSPVSLPPEYL  1001

Query  586   ELPKRLGGFGI  596
              LP+ LGG G
Sbjct  1002  SLPQVLGGLGC  1012
```
